# What needs to be standardized for reliable, reproducible, and robust tractography?

**DOI:** 10.1093/gigascience/giag034

**Published:** 2026-03-25

**Authors:** Jon Haitz Legarreta, Simona Schiavi, Wei Tang, Garrett Banks, Matthew Cieslak, Kurt Schilling, Alberto De Luca, Jacques-Donald Tournier, John Kruper, Francois Rheault, Stamatios N Sotiropoulos, Franco Pestilli, Jelle Veraart, Joseph Yuan-Mou Yang, Maxime Descoteaux, Sarah Heilbronner, Ariel Rokem

**Affiliations:** Department of Radiology, Brigham and Women’s Hospital, Mass General Brigham, Boston, MA, USA; Harvard Medical School, Boston, MA, USA; ASG Superconductors S.p.A., Genoa, Italy; Department of Computer Science, University of Verona, Verona, Italy; Department of Psychological and Brain Sciences, Indiana University, Bloomington, IN, USA; Department of Neurosurgery, Baylor College of Medicine, Houston, TX, USA; Lifespan Informatics and Neuroimaging Center (PennLINC), Department of Psychiatry, Perelman School of Medicine, University of Pennsylvania, Philadelphia, PA, USA; Penn/CHOP Lifespan Brain Institute, Perelman School of Medicine, Children’s Hospital of Philadelphia Research Institute, Philadelphia, PA, USA; Department of Psychiatry, Perelman School of Medicine, University of Pennsylvania, Philadelphia, PA, USA; Department of Radiology, Vanderbilt University Medical Center, Nashville, TN, USA; Image Sciences Institute, University Medical Center Utrecht, Utrecht, the Netherlands; Department of Biomedical Engineering, School of Biomedical Engineering and Imaging Sciences, King’s College London, King’s Health Partners, St. Thomas’ Hospital, London, UK; Centre for the Developing Brain, School of Biomedical Engineering and Imaging Sciences, King’s College London, King’s Health Partners, St. Thomas’ Hospital, London, UK; Department of Psychology, University of Washington, Seattle, WA, USA; Sherbrooke Connectivity Imaging Lab (SCIL), Department of Computer Science, Université de Sherbrooke, Sherbrooke, Québec, Canada; Sir Peter Mansfield Imaging Centre, School of Medicine, University of Nottingham, Nottingham, UK; NIHR Nottingham Biomedical Research Centre, School of Medicine, Queen’s Medical Centre, Nottingham, UK; Department of Psychology, The University of Texas at Austin, Austin, TX, USA; Department of Neuroscience, The University of Texas at Austin, Austin, TX, USA; School of Medicine, New York University, New York, NY, USA; Department of Neurosurgery, Neuroscience Advanced Clinical Imaging Service (NACIS), Royal Children’s Hospital, Melbourne, Victoria, Australia; Neuroscience Research, Murdoch Children’s Research Institute, Melbourne, Victoria, Australia; Department of Paediatrics, University of Melbourne, Melbourne, Victoria, Australia; Sherbrooke Connectivity Imaging Lab (SCIL), Department of Computer Science, Université de Sherbrooke, Sherbrooke, Québec, Canada; Department of Neurosurgery, Baylor College of Medicine, Houston, TX, USA; Department of Psychology, University of Washington, Seattle, WA, USA; The University of Washington eScience Institute, University of Washington, Seattle, WA, USA

**Keywords:** neuroanatomy, standardization, tractography, brain connectivity, white matter, computational neuroimaging

## Abstract

Tractography is a key component of efforts to map brain connectivity. As a rapidly evolving field of neuroscience, current tractography methods are diverse, often varying across research laboratories and different software pipelines. Therefore, it suffers from a lack of standardization, leading to inconsistencies in results, which can limit reproducibility and affect the robustness needed for research and clinical applications of these methods. Variability in data acquisition procedures, inconsistencies in spatial referencing schemes and implementations, and anatomical heterogeneity—at the individual level, across the lifespan, and across species—hinder comparative analyses. Additionally, the lack of consensus on best practices complicates the development of robust automated quality control pipelines and limits the clinical translation of tractography-based procedures. Establishing standardized protocols for acquisition, preprocessing, and tractography reconstruction is critical toward enabling reliable tract-specific analyses, facilitating cross-study harmonization, and supporting replicable large-scale population studies. The present article provides an overview of the current challenges in tractography standardization and identifies the key aspects that require standardization for reliable, reproducible, and robust tractography.

## Background

Understanding human brain anatomy across different organizational levels is a central goal of contemporary neuroscience. There is increasing evidence that structural connectivity across the white matter underlies many of the capacities of the living brain and that its physical properties are linked to human brain health [1–4]. This understanding has grown with the development of methods to measure brain connections, and it has also fueled a new generation of data collection and data analysis methods. Magnetic resonance imaging (MRI) measurements are noninvasive and can be used to study brain connectivity *in vivo* [5]. These data are complemented by other invasive techniques that highlight brain connections by using specific chemical tracers, histological stains, and molecular markers; by dissecting the tissue; and by direct optical observation [6–13] or using X-ray imaging methods [9, 14]. *Computational tractography* assesses the location, direction, and pattern of brain connections, estimating their trajectories (with an individual trajectory, often referred to as a *streamline*). However, as the methods evolve and the promises from their application grow, it is important to take stock of challenges that hinder leveraging their full potential. Other works have examined challenges related to the accurate delineation of brain connections [15–18] or challenges related to definitions of certain anatomical concepts in such data [19, 20]. The present article focuses specifically on the challenges related to standardizing representations of tractography-derived brain connections in digital formats.

These challenges have become increasingly pressing as the field has transitioned in recent years toward larger and larger datasets. This increase in data volume and complexity arises from large-scale data acquisition projects, with thousands of subjects, on the one hand. Examples of these include the Adolescent Brain Cognitive Development (ABCD) [21], Healthy Brain and Child Development (HBCD) [22], Healthy Brain Network [23, 24] studies; the UK Biobank [25]; and many others. On the other hand, it arises from the increase in the resolution as well as coverage of measurements. These larger data have led to the application of data-driven discovery methods, where consistent and comprehensive standards are necessary.

Establishing standards and best practices supports transparent, reproducible, and robust research through application of the FAIR (Findable, Accessible, Interoperable, Reusable) principles [26]. In addition, establishing usable standards can enable research that is otherwise difficult, making hard things easier and making the impossible practical. With the recent establishment of the International Society for Tractography (IST) [27] and its standardization unit (members of which are authors of this article), together with several consensus efforts in diffusion MRI (dMRI) acquisition and processing [18, 28, 29] led by the Diffusion Study Group [30] of the International Society for Magnetic Resonance in Medicine (ISMRM) [31], we see an opportunity to advance broadly applicable and widely accepted community standards that will pave the way toward future research of brain connectivity.

**Figure 1:**
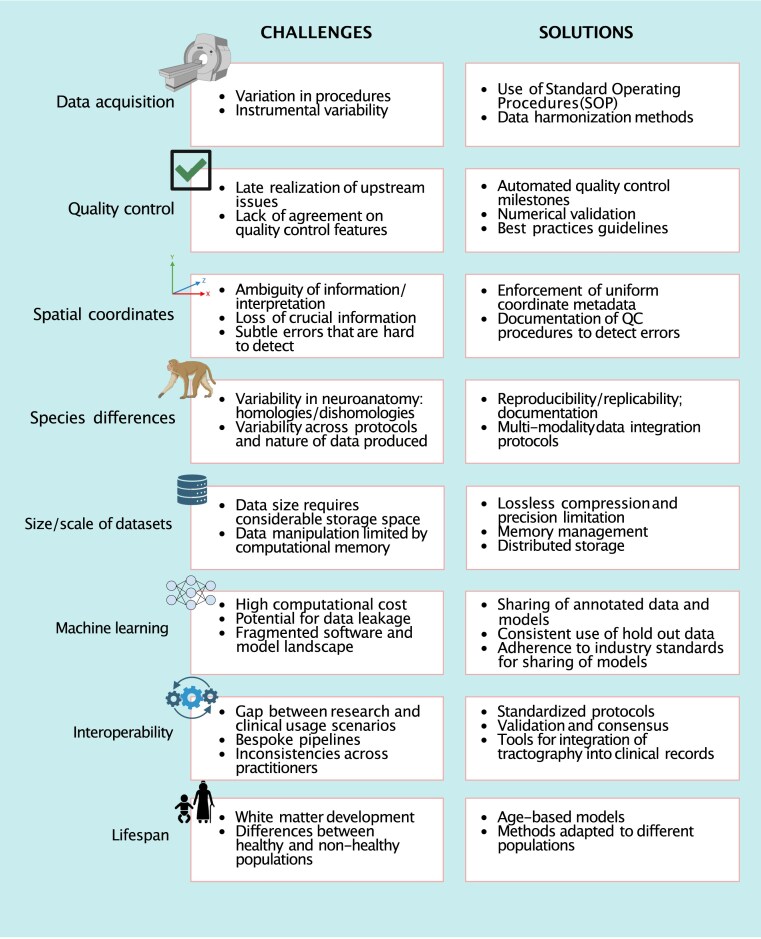
Summary of main challenges and suggested standardization solutions toward reliable, reproducible, and robust tractography.

This work introduces some of the current challenges that arise from gaps in standardization for tractography and offers some potential solutions to them. We provide a set of recommendations for the broader research community to pursue the solutions that we identify. The recommendations presented herein will improve the rigor and impact of work that uses brain tractography and will enable better understanding of brain connectivity. The article is organized by the different stages of the data life cycle and a range of domains in which standardization poses challenges (Fig. 1), concluding with a set of recommendations (section Summary and Recommendations).

## Challenges and Solutions

Reliability, reproducibility, and robustness in tractography encompass multiple methodological dimensions. They manifest across the full data life cycle of tractography, including acquisition, processing, and analysis. They also manifest in inferences applied across different species, different types of measurements, different ages, and across basic research and clinical applications. There are many definitions of these constructs [32–34]. We define *reliability* as the stability of tractography algorithms in the face of varying conditions (e.g., noise perturbations in test–retest, multisite or multivendor datasets). *Reproducibility* is defined as the ability to obtain the same results with the same data and same software used in the original study. Thus, reproducibility is mostly about the open and unencumbered availability of research products and, importantly, the compliance of openly available products with conventions and standards. *Replicability*, a closely related term, is the ability of another research team to produce findings that are consistent without using the materials used by the original research team, but while trying to emulate the methods as closely as possible. This will depend to a large degree on an adequate description of the methods used. This will also depend on the *robustness* of the findings, which we define as the ability to obtain the same expected anatomical observations using different data and/or different software or even methods that differ in their assumptions and implementation details—for example, the ability to delineate a certain brain structure in data obtained on different instruments, from different participants, and using different tractography algorithms. All 3 of these are necessary conditions for trustworthy, transparent research and an ability for research efforts to efficiently build on previous work. As we will demonstrate in addressing a range of practical and conceptual challenges, standardization is essential to achieve these goals. Therefore, as we survey a range of challenges and proposed solutions, we will highlight key factors influencing these aspects and propose ways to increase them.

### Data acquisition

The utility of standards starts with the moment that data are created. Different experimental methods produce tractography data, and considerations for standardization may be different for each one of these. Furthermore, the creation of some data is already governed by numerous existing standards. For example, in a large number of cases, MRI data are acquired following the Digital Imaging and Communications in Medicine (DICOM) standard [35]. Similarly, modern storage and sharing of reconstructed (raw or processed) research MRI data is standardized by the Brain Imaging Data Structure (BIDS) [36] specification [37], including several extensions for dMRI and brain connectivity (e.g., [38, 39]). These standards not only facilitate the structured storage of the data themselves but also prescribe necessary metadata. In principle, this should facilitate the standard processing in subsequent steps, but undocumented and poorly standardized procedures are typical of many different experimental techniques and a source of unwanted variability.

Differences across datasets can also arise due to a range of nonbiological variables, such as variation in acquisition protocols, scanner hardware, reconstruction pipelines, and tractography workflows, which are hard to reconcile, even given a full description of the data acquisition. These sources of variability can have a significant impact on downstream tractography results, particularly in multisite studies and large-scale datasets. For example, acquisition resolution, diffusion sampling schemes, and vendor-specific differences introduce variability in the spatial geometry and microstructural characteristics of reconstructed brain tracts that compromise reproducibility and robustness [40–44].

Statistical methods for *harmonization*, which aim to eliminate such differences at the level of the raw data [45], provide promising solutions to some of these challenges, but the intricacy of downstream tractography analysis workflows poses further challenges. These workflows involve subjective parameter choices, user-defined constraints, and possibly different priors or anatomical reference definitions that influence reproducibility and anatomical fidelity. Cross-scanner and cross-vendor effects propagate through these pipelines, leading to inconsistencies in reconstructed geometry, volume, and quantification of tract properties. Recent efforts to standardize processing pipelines and implement robust quality control protocols across sites have shown promise in reducing these inconsistencies, thus improving robustness [46–49].

Tractography is rarely the sole purpose of acquiring dMRI data. Quantitative characterization of tissue microstructure is often a concurrent goal, which imposes additional and occasionally conflicting constraints stemming from distinct acquisition requirements. As the sequences for probing the tissue microstructure get more sophisticated (e.g., using tensor-valued encoding), more variability can be expected to be present in the dMRI data, and more nuanced tractography reconstructions can be expected [50]. Thus, additional methodological development is needed to align such multidimensional acquisition protocols across sites, to manage scan time constraints while jointly supporting tissue microstructure modeling and tractography.

Despite the progress that has been achieved, further work is needed to provide an adequate basis for tractography. Using BIDS for neuroimaging data sharing offers a high level of consistency; however, its adoption across pipelines is not yet all-pervading, and its implementation is not uniform. Furthermore, although the diffusion extension proposal has been submitted for inclusion in the standard, it only covers scalar derivatives and tensors. The tractography extension has not been submitted yet for inclusion. Similarly, vendor-agnostic, standardized sequences have yet to be implemented across the variety of scanning hardware and protocols and validated extensively. Limited support and interest for regulatory clearance entail additional hindrance to their wide adoption [51]. Finally, harmonization is still a topic under active research or presents limitations (e.g., fixed, well-populated site numbers, homogeneous populations) that have yet to be overcome. Thus, significant methodological gaps remain in dMRI data acquisition that limit the development of automated and robust tractography downstream.

Standard operating procedures (SOPs) are necessary to remove variability at data collection and provide some level of reliability in tractography reconstruction from a given study. SOPs have been employed to describe the imaging and processing protocols for large-scale studies (e.g., the Human Connectome Project [HCP] [52], the ABCD study [53]); more recent studies (e.g., the HBCD study [54] or the Human Connectome PHantom Study (HCPh) [55]) have adopted contemporary web technologies to modernize data presentation. Smaller-scale, single-site studies would likewise benefit from this practice, contributing to improved transparency in reporting and reproducibility. Similarly, standardized data and metadata records and organization (e.g., through BIDS) are required to ensure reproducibility. This includes developing the extensions to derivatives (e.g., fiber orientation distributions) that are employed to generate tractography results. Finally, harmonization solutions (within and across scanners and sites, across acquisition upgrade packages, etc.) are required to resolve data source variability. Combined with the adoption of unambiguous, self-reporting data formats (see Spatial coordinates section), these measures would mitigate practical barriers and contribute to reducing overhead in multicenter studies with disparate settings.

### Quality control

There are many well-established pipelines for quality control (QC) of raw MRI data [56], including dMRI data [57, 58], as well as data that have undergone initial preprocessing (i.e., denoising, correction of motion and eddy current artifacts, and removal of other artifacts) [24, 59, 60]. Presumably, QC procedures applied at these early stages should catch many of the issues that would impact subsequent analysis steps. However, QC can and should be done at multiple different stages of the analysis, because errors can occur at each one (e.g., registration between different imaging modalities, separation of the image into different tissue types and regions of interest for tractography initiation). Oftentimes, many of these issues only become apparent when computational tractography is conducted. This means that QC of tractography results is still necessary. In practice, QC of computational tractography pipelines is often done through visual examination of whole-brain tractograms (the collection of all streamlines estimated in a single brain) or by their ability to identify the locations and trajectories of large, well-known tracts. However, this approach is challenged by high interrater variability and an apparent lack of consensus on the structure of these tracts, even in the same set of streamlines [19, 20].

Another approach for QC of tractography results is through numerical validation. In this approach, individual streamlines and sets of streamlines are subjected to statistical evaluation with respect to the measured data. In these methods [61–66], individual streamlines or sets of streamlines are given objective scores based on how well their trajectory conforms to the data used to generate the streamlines (e.g., in contrast to smoothness constraints, anatomical constraints, or randomness that is introduced in the process of tractography). Other methods use machine learning and deep learning techniques to filter streamlines based on their geometric properties or a representation of them [67, 68]. The benefit of these approaches is that they provide objective numerical values that can then be used as benchmarks for QC procedures. Overall, the field would benefit from more studies that demonstrate the utility and sensitivity of different QC procedures in relevant scientific inferences [69, 70], including validation by reference to known anatomical structure and via the detection of individual differences related to development, aging, or clinical conditions [71].

Taken together, progress has been limited in tractography QC standardization practice and adoption levels. Although there are many advanced tractography visualization tools and paradigms [72], and despite some pipelines offering automated reporting, their use for QC purposes has not been thoroughly studied. Similarly, validating numerical tractography QC methods is still impacted by the inability to have gold standards for real brain data and the lack of anatomical measures defining the success of a tractography method. As a result, best practices for excluding low-quality results based on either visual or numerical reports remain insufficiently established. Reliable tractography would benefit from clearly defined, shared criteria determining the quality of results, including standardized reporting. Additionally, universally shared phantom and synthetic data, as well as anonymized *in vivo* and *ex vivo* data from both healthy and diseased participants, are required to guarantee a common ground for quality control of a given tractography pipeline. This includes sharing data in software-agnostic repositories (e.g., OpenNeuro [73] or Zenodo [74]). Note that this extends beyond datasets used across international challenges that quantify tractography results only within a particular dataset (see, e.g., [16, 17, 75, 76]).

### Spatial coordinates

Representing spatial coordinates in an unequivocal frame is a known challenge in many fields of science that deal with spatial information. This is a well-recognized issue in anatomical studies of the human brain, where different types of information (e.g., structural, functional, physiological) need to be integrated. Given the variety of information sources that are involved, with each acquisition modality having particular spatial properties, the potential for errors is high and the consequences detrimental to the study of brain connectivity [77].

**Figure 2:**
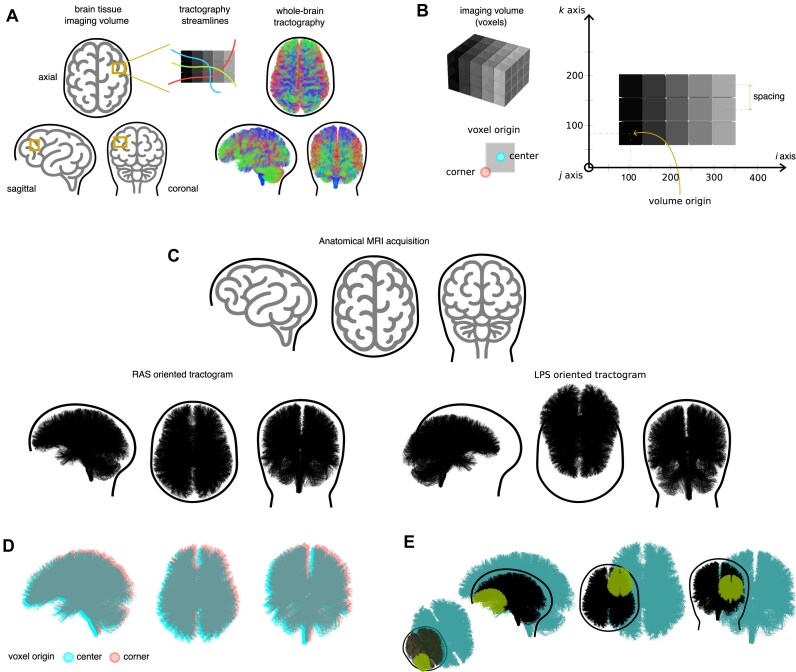
Tractography data representation issues. (A) Schematic view of the process of generating tractography streamline data from an imaged brain tissue volume. (B) Miscellaneous concepts related to tractography data. (C) Illustration of the spatial mismatch between a tractography file natively serialized following the *LPS+* convention but the structural data (e.g., a T1-weighted MRI acquisition) being arranged following the *RAS+* convention. (D) Effect of the voxel origin convention visualized as a half-voxel shift between the *corner* and *center* conventions on a whole-brain tractogram. (E) Illustration of tractography data representation issues arising from mismatches between the spatial coordinate transformation matrix employed when reconstructing streamlines versus the one used when serializing the tractography data, from storing the data in voxel space versus real-world space coordinates, or from inadvertently applying spatial transformations multiple times. In all cases, the tractogram in black is at the correct location, and the tractograms in green and turquoise do not lie at the correct anatomical location.

Tractography algorithms typically output data as a set of 3-dimensional (3D) coordinates that represent the trajectory of particular brain white matter tracts (see Fig. 2A, B). In reconstructing and interpreting these structures, several coordinate frames need to be considered. One coordinate frame refers only to the individual participant’s brain, allowing one to locate a structure relative to a particular anatomical landmark. Another coordinate frame is the position of the individual’s brain within the scanner, that is, relative to the origin of the scanner’s fixed reference frame (e.g., the iso-center of the MRI’s bore). Another coordinate frame to consider is that of the grid of *voxels* included in the measurement itself.

Volumetric imaging data follow particular anatomical conventions that facilitate relative positioning and orientation. These conventions split the space according to 3 orthogonal planes: *axial, coronal*, and *sagittal* (Fig. 2A). When storing real-world spatial data as discrete imaging data, the information can be arranged according to a particular basis. Two typical conventions are *RAS+* (right, anterior, superior, with the $+$ sign indicating that coordinates increase from left to right, posterior to anterior, and inferior to superior) and *LPS+* (left, posterior, superior; the same principle applies to the $+$ sign). If no metadata are accessible, applications reading tractography results may assume the incorrect convention (Fig. 2C).

At times, the conventions implied by a particular format are not made apparent in the corresponding documentation. A typical example of this issue is the definition of the origin of a voxel: some tractography formats consider the origin to be at the center of the voxel, whereas others consider it to lie at the corner. This may arise in the correspondence between streamline coordinates and other coordinate-based (e.g., volume-based) data: unless this is carefully considered, a half-voxel shift will exist between the streamlines relative to their intended world coordinates. Visual inspection is not always sufficient to notice this (Fig. 2D), and such small systematic errors can have significant consequences in downstream applications. Some tractography data-processing tools may fail to read streamlines that, due to not accounting for this shift, lie outside the volume bounding box. In connectivity analyses, these errors may lead to mismatches between surfaces or label maps reconstructed from T1-weighted data and the tractography space, resulting in incorrect streamline assignments and spurious inter- or intrahemispheric connectivity patterns.

Finally, streamlines generated by tractography algorithms may have their own positions relative to each of the other coordinate frames and may be tied to an additional piece of data (e.g., another data file). The units used to store their position may also differ from the units used to refer to the measurement (discrete voxels, or continuous millimeters). Incongruent coordinate frames and units can lead to errors such as the ones shown in Fig. 2E.

Overall, despite a well-established understanding of the coordinate frameworks involved in tractography reconstruction, current pipelines continue to rely on implicit or insufficiently documented conventions. Clear and unambiguous guidance for debugging, validating, and converting data across coordinate spaces and formats remains lacking. Consequently, meaningful progress in this area has remained limited. A tractography data standard that explicitly and unambiguously documents its spatial coordinate framework (e.g., according to the BIDS convention [78]) and version-controlled, general resources (i.e., not tied to a particular processing package) are required. This includes testing data stored in software-agnostic repositories and guidelines toward conformance, error checking, and conversion of retrospective data.

### Translation across species and methods

Anatomical tract-tracing methods involve injecting a dye or tracer into a living brain, waiting a set period of time, and then extracting the brain for histology and microscopy [79]. Thus, these methods provide information that is not accessible with MRI but, for obvious reasons, cannot be performed in humans. Thus, tract-tracing is performed in brains from nonhuman animals, such as primates and rodents. Combining these methods with dMRI in nonhuman models provides a unique opportunity to verify dMRI-based tractography against anatomical gold standards and to iteratively improve these methods. Several large-scale efforts aim to provide high-quality dMRI along with microscopy and tracers in the same nonhuman primate brains (e.g., the Large-scale Imaging of Neural Circuits [LINC] BRAIN CONNECTS center [80] or the Center for Mesoscale Connectomics [CMC] [81]). Openly accessible resources from these consortia and others (such as PRIME-DE [82] or the BRAIN/MINDS portal [83]) will be invaluable in optimizing, standardizing, and developing the next generation of tractography approaches. Using unified methods that combine these multimodal and multiscale data would ultimately enable informed judgment about cross-species homologies/dishomologies. However, this process is complicated by several factors. Nonhuman brains—even in our closest primate relatives—are not simply smaller but also have different morphological and biophysical properties.

This poses foremost a challenge to the validity of inferences between species. Integration between human and nonhuman primate data remains conceptually challenging because nonhuman anatomical knowledge relies heavily on atlases based on cytoarchitectonics (e.g., [84]). These do not directly align with the common atlases used in human MRI studies (e.g., [85]). While cytoarchitectonics-based human brain atlases have been developed [86], some even with a particular stress on cross-species homology [87], these have not been integrated with widely used neuroimaging tools. At the same time, common coordinate frameworks for nonhuman primates are beginning to emerge (see, e.g., [88]), in some cases with the explicit goal of establishing a direct relationship with the developing human connectome [89]. Tractography fingerprinting [90] (i.e., using white matter bundles as latent landmarks to assess similarities and divergences across the 2 species) is geometry-agnostic and can provide additional solutions to the challenge of integrating cross-species coordinate frameworks.

Integration with studies in rodents is further complicated by the extent of species dishomology. Their white matter bundles are organized very differently from primates’ [91], and their lissencephalic brains mean that many of the MRI-based tractography methods that work in primate brains do not translate. On the other hand, a host of genetic, molecular, and imaging tools readily available for circuit characterization and manipulation in mice is not feasible in humans or nonhuman primates. Thus, merging dMRI tractography with tract-tracing, spatial transcriptomics, and imaging data in the mouse model could be highly valuable. However, while anatomical atlases in primate models have a long history (e.g., [84, 92]), just 2 decades ago, there were virtually no standard frameworks (atlases, metadata, structural ontologies, etc.) for the mouse brain. However, with major investments in generating large-scale datasets of mouse brain transcriptomics, cell typing, connectivity, and function, the need for a standard framework became overwhelming. The Allen Mouse Brain Common Coordinate Framework (CCF) [93, 94] (current version CCFv3 [95]) has emerged as a standard framework for organizing data about mouse brain connectivity. The enormous success of this framework illustrates the value of standardization. An extensive informatics pipeline at the Allen Institute for Brain Science allows users to download everything from raw images to structural summaries of connectivity, and tools that use the CCF can integrate data from multiple sources. However, these tools are not currently interoperable with MRI data, aside from at the crudest (region-to-region connectivity summaries) level.

The methods employed to map connectivity across species also differ in profound ways due to the nature of the acquired data; while axonal pathways in the rodent brain are reconstructed from optical data (e.g., electron microscopy), human brain pathway reconstruction using tractography employs dMRI data. Thus, the data representation structures employed are different: in rodent data, anatomical structures (gray/white matter boundaries, nuclei volumes, cells, fibers, etc.) are represented in standard anatomical reconstruction software (e.g., Neurolucida) that interfaces with microscopes as points, contours, and surfaces. Downstream processing software such as IMOD [96] inherits the file formats that organize these structures. This is conceptually different from the MRI file conventions, which use voxels as their primary representational unit. The nature of the contrast in the acquired data necessitates the development of distinct methods (e.g., segmentation and stitching as opposed to streamline propagation), introducing a supplemental layer for divergence. As a consequence, the progress to bridge the gap in tractography data representation and software across species and imaging modalities has been limited. Addressing these challenges requires a comprehensive approach that includes standardized multimodality and multiresolution data acquisition, advancements in hardware for efficient *in vivo* scanning, essential validation studies, and methodological innovations, such as automated tools for establishing a common framework of correspondence.

While the challenges to validity and generalization are evident, these issues also raise challenges to robustness. This is because the differences in measurement tools and in analysis methods need to be thoroughly adapted and validated when they are used in species other than the ones on which they have been developed. Examples in which tractography tools that were developed for use in human dMRI data were adapted to be used in nonhuman data [97] suggest that there is moderate progress on this challenge.

### The scale of data

Like many other datasets of spatial biological information, tractography data can take up large amounts of storage. A typical dMRI tractography file occupies several gigabytes (GB) of data. The size depends on the spatial and angular resolution of the diffusion data, the sampling density employed to reconstruct streamlines, the number of streamlines that were generated, the data associated with each point or each streamline (e.g., scalars that represent tissue properties along their length), and the precision employed to store the data. Additionally, the size of the tractography output may also depend on the resolution of the acquisition. For example, with sub-millimeter resolution MRI acquisitions, the size of tractograms can easily reach the terabyte (TB) scale [98]. With other modalities that provide even higher image resolution in *ex vivo* tissue samples, such as polarization microscopy [7, 8, 10, 12], X-ray microcomputed tomography [99], X-ray scattering [9], or synchrotron technology [14], data are orders of magnitude larger (see, e.g., [100, 101]). Manipulating such data volumes poses a challenge in terms of the required hardware and computational power. As imaging acquisition hardware evolves, and data storage capacity and physical memory limits increase, current analysis tools may buckle under the strain. This means that some approaches, which work well with smaller datasets, can become infeasible when translated into larger data, threatening robustness. A few technical and standardization developments may ease this strain. First, tractography data lend themselves to compression, with *linearization*, where collinear points are discarded (with some tolerance threshold), being the fastest and most efficient approach [102–104]. Additional compression is provided by using lower-precision numerical representations (i.e., 16-bit floating point precision, instead of the extended 64), which can provide significant space saving, without much loss of anatomical information [105]. Similarly, some visualization techniques, such as level of detail, occlusion culling, or visibility-based rendering optimization, aim to save computational resources by avoiding rendering data that are occluded by other objects or by rendering them at lower resolutions (see [106] for a recent method). Second, new file formats and distributed computing paradigms offer opportunities to scale computing to very large datasets. As an example, a newly proposed file format, *TRactography eXchange* (TRX) [107], was designed to make it easy to create large tractograms with minimal random access memory requirements. This enables memory mapping, providing a convenient and efficient way to access data directly from disk.

Software and data format enhancements have so far enabled only limited progress, due to heterogeneous precision adjustment across pipelines and partial adoption of the TRX file format. Data storage and visualization remain major bottlenecks, and significant advances have relied primarily on improvements in computational power, with efficient mapping of brain connectivity at scale being possible on supercomputing hardware [108] or using Graphics Processing Units (GPUs) [109, 110]. Distributed data storage (e.g., with zarr [111]) could offer an attractive possibility for work with very large datasets. Effective visualization of memory-intensive tractography data will require implementations in low-level programming languages, such as C++ or Rust, and/or compatibility with GPU hardware acceleration.

### Integration of modern machine learning methods

The increasing adoption of machine learning and artificial intelligence (ML/AI) methods in dMRI and tractography has raised new challenges and exposed limitations in current standardization practices. Modern ML/AI methods require large, diverse, and well-curated datasets to achieve robust generalization. These requirements compound many of the challenges described. For example, in the absence of reliable and automated QC tools, labor-intensive quality control procedures are required. Spatial coordinate inconsistencies within large heterogeneous datasets may cause deep learning models to fail to converge. Similarly, poor data organization and metadata standardization—due to both poor compliance with BIDS and the preliminary status of the related extensions—hinder reproducibility and compromise downstream analyses, making cross-study comparisons more challenging. Inadequate handling of data splits can lead to train–test leakage [112]. Such leakage risks inflate reported performance and obscure the true generalizability of ML/AI-based tractography methods. Although some annotated datasets can be used as the basis for training ML/AI, the scarcity of large, well-annotated, and openly shared tractography datasets —particularly multisite and longitudinal datasets that include nonhealthy individuals for clinical relevance—constrains the development of robust and reliable tractography. The difficulty of reliable evaluation and the absence of universally accepted anatomical ground truth, in particular, are not specific limitations for the evaluation of ML/AI algorithms, but for the evaluation of tractography in general. Nevertheless, while these issues are broadly applicable to tractography methods more generally, they are exacerbated in ML/AI methods, which are oriented toward quantitative metrics of performance for their consistent improvement.

Furthermore, there is a need for standardization within the ML/AI methods themselves. ML/AI-based tractography techniques exhibit substantial heterogeneity in model architecture, input representation, and learning objectives, often making direct comparisons difficult (see [112–114] for relevant review works). Existing approaches range from voxel-wise classifiers and streamline propagation networks to graph-based and geometric learning formulations. As such, the input to the model may be the raw diffusion signal, the local orientation information represented as spherical harmonics coefficients or fiber peaks, and so on. Additionally, the contextual information to regularize the optimization process is also heterogeneous across methods and includes tissue maps, neighboring voxel information, and so forth. Other approaches avoid reliance on the modality that sensitizes axonal pathway architecture (e.g., dMRI or other) and estimate tractography without explicit orientation data. These factors result in differing dataset requirements for training various models, often leading to substantial increases in computational requirements, in terms of storage and processing resources. The high computational cost—including the need for specialized hardware, such as GPUs—makes regular testing and evaluation of methods expensive and resource-intensive.

This heterogeneity is compounded by the absence of a universally accepted anatomical ground truth for white matter pathways, a limitation that challenges the current tractography landscape. The evaluation of AI-based tractography algorithms frequently relies on indirect metrics, simulated phantoms, or expert-defined references, each with inherent limitations. In this context, the absence of standardized reporting conventions is particularly problematic for evaluating AI models. Although volume- and streamline-oriented measures have been well established since landmark studies [17, 115], evaluation tools remain poorly maintained, with most pipelines relying on bespoke implementations. Additionally, the assessment of application-specific derivatives—such as tract-specific metrics, connectivity measures, or quantitative microstructural parameters—is often inconsistent or omitted entirely. Additionally, representations and trends learned by ML/AI systems may be dominated by site- or protocol-specific effects. Systematic reporting of scanner characteristics, acquisition parameters, session-level metadata, and preprocessing choices is essential for replicability and robustness and for interpreting performance gains as genuine methodological advances.

The result is a fragmented landscape in which performance claims are highly dependent on specific datasets, tasks, and evaluation choices, increasing the difficulty of reliable evaluation. Together, this underscores the necessity of standardization for AI-based tractography, encompassing access to well-annotated and fully described datasets, common validation frameworks with standardized benchmarks, metrics, and reporting procedures. Such standardization would support integration with established neuroimaging pipelines, as well as improve reproducibility, robustness, and maintainability. At the same time, it is important not to overstandardize too early. That is, early-stage research needs to maintain diversity in the approaches a scientific community explores, which is essential for innovation, and increases the opportunities for the community to discover good solutions. Taken together, these considerations suggest that standardization of ML/AI approaches merits caution and careful consideration, to support the goals of reliability, reproducibility, and robustness, even while not stifling innovation.

Building on the heterogeneous methodological terrain described above, competition among large corporate entities (e.g., technology companies) is also driving further barriers to interoperability in this ecosystem, as different entities try to position their tools as dominant in the marketplace. As externally developed components are introduced into established processing pipelines, they promise incremental value but require careful accommodation within historically entrenched workflows. In cases where models are shared not only as software but also in the form of parameter values (or “weights”), the standardization of the format of model parameters and of the metadata associated with these parameters becomes important. This is where industry standards such as the Open Neural Network Exchange (ONNX) standard [116] could play an important role. For these use-cases, there is much to benefit from the adoption of already existing standards that apply broadly, rather than reinventing these standards for narrow applications.

### Translation of tractography methods to clinical applications

Tractography is used clinically to aid in the planning and execution of neurosurgical procedures [117]. Tractography has proven useful during the resection of epileptic foci or brain tumors [118, 119]. In this case, a surgeon might utilize a different surgical approach to the tumor to avoid certain white matter tracts, particularly those involved with motor, language, and visual function. This is especially the case in slow-growing tumors and pediatric developmental abnormalities, where the standard anatomical organization of white matter pathways can be significantly altered while remaining functional. To this end, tractography-guided brain tumor resections rely on functional brain mapping through direct brain electrical stimulation to confirm white matter tract positions and resection functional boundaries during awake surgery. In other instances, tractography is utilized for precision targeting in stereotactic procedures such as deep brain stimulation and focused ultrasound, such as when localizing the dentatorubrothalamic tract, a neuromodulation target for treatment of essential tremor and tremor-dominant Parkinson’s disease [120–122].

Despite constituting a useful tool for improving neurosurgical outcomes and mitigating the likelihood of postoperative complications, its use remains limited [123, 124]. Diffusion tensor-based deterministic tractography remains the prevalent tool in neurosurgical preoperative planning, largely because it is supported by many commercially available navigation platforms [125]. Yet, limitations of the tensor model result in incomplete reconstruction and visualization of complex fiber architecture (e.g., crossing, fanning, and bending pathways) in clinical practice.

Tractography with advanced models is beginning to appear in commercial software, but adoption remains slow due to increased likelihood of spurious fibers, limited clinical validation, and inconsistent protocols and heterogeneous methodological frameworks [124, 126]. This is especially pronounced when examining fine-scaled structures, such as cranial nerves, highlighting the sensitivity of the tracking parameters with respect to the structures of interest [127]. Additionally, results provided by intraoperative tractography are constrained by the limited acquisition and processing time.

Standardization of clinical workflows is complicated because these applications require thorough validation and consensus among experts. This requires processing software to remain very stable over time and makes updates to the software very difficult in these settings. Variable imaging protocols also make consensus more difficult to reach (see Data acquisition section). Benchmarks and standard validation methods are very challenging to formulate, because clinical use-cases are diverse, and there are concerns that methods may be affected by the presence of pathologies. Furthermore, clinicians (e.g., radiologists or neurosurgeons) may use bespoke pipelines developed in-house to perform tractography, making translation across settings difficult. Finally, depending on the clinical setting and operative urgency, some processing pipelines that are common in research settings may be too time-consuming and/or computationally intensive.

Thus, unlocking tractography’s full potential in clinical routine necessitates further development along several critical dimensions. Consistent acquisition and processing protocols are required to produce tractography results that can be translated across clinical settings. In addition, methodological innovations are needed for time-constrained clinical acquisitions—such as optimized pulse sequences, undersampling strategies, and super-resolution techniques—that enable robust tractography results, alongside tractography methods that remain reliable when employed on lower-quality data. Additional measures to support clinical adoption include systematic reporting of reconstruction uncertainty across varying data quality conditions (e.g., noise levels) and reconstruction settings (e.g., seeding strategies). Finally, establishing reliable proxies for clinical outcomes is essential to validate the reliability of fiber reconstruction techniques.

### Standardization of methods throughout the lifespan

The brain changes significantly throughout the lifespan, and there is a wealth of evidence that lifespan development of brain connections is linked to health outcomes (see [128] for a review). The changes in the properties of the brain also pose challenges for the standardization of methods across different epochs of life. For example, studying early life brain development is challenged by the simple fact that the brain is substantially smaller at birth, with total brain volume more than doubling during the first 12 months of life [129]. This poses a particular challenge to the use of standard atlases that localize certain structures, as these atlases are usually constructed based on healthy young brains. To address this challenge, researchers have been developing detailed and time-resolved atlases in early life [130, 131] and even during gestation [132]. Furthermore, while the major brain tracts are already established at birth [133], it is not simply the case that the infant brain is a scaled-down version of the adult brain. There is some evidence that the curvature of some structures is different in this early phase relative to later development [134]. In addition, the tissue properties of brain connections in early life are quite different from those of a more mature brain. This can pose a challenge to the use of standard tractography methods, which sometimes rely on assumptions about the biophysics of the tissue, which may not hold [135]. Similar changes apply in aging, as tissue properties of the white matter change again with age in a manner that can impact standard tractography methods [136]. The challenge of studying the developing and aging brain often intersects with challenges related to harmonization (mentioned in the Data acquisition section) because it is difficult to obtain a large sample that covers all ages within a single study, necessitating integration of data across studies and differing acquisitions.

Progress in offering authoritative, centralized, and standardized resources for brain connectivity mapping across the lifespan has been limited; as an example, the extension of the BIDS standard to describe atlases does not, at the moment, cover tractography templates. Standardizing tractography across the lifespan will require creating such templates, generating normative derivatives linking structural connectivity to tissue microstructure (tractometry), providing uniform method implementations, and establishing guidelines for calibrating processing parameters.

## Summary and Recommendations

Despite the above challenges, if a series of recommendations is followed, tractography reconstruction and interpretation can be highly consistent and robust. Below, we provide a set of recommendations for building standardized, reliable, and robust tractography data reconstruction and sharing procedures. We also discuss the feasibility of the suggested measures in terms of the effort required to implement them (minimal, moderate, or substantial).

1. Implement standard operating procedures [137] during data acquisition (i.e., written sets of instructions that specify the processes that take place during the measurement). Feasibility: A moderate effort is required to implement centralized SOPs toward tractography standardization. That said, some examples of SOPs for tractography data acquisition [138], quality assurance [58], and analysis [139] already exist (albeit with a focus on dMRI) and could easily be adopted by the community.
2. Adopt a vendor-agnostic, open-source pulse sequence design and reconstruction frameworks (e.g., Pulseq [140, 141]) to facilitate more similar results across the instruments of different vendors. Feasibility: A substantial effort is required to make vendor-agnostic pulse sequences extensive within the data acquisition hardware, as it involves vendor support and validation toward regulatory clearance.
3. Advance BIDS-compliant standards for tractography inputs and outputs. Akin to a wide range of neuroimaging use-cases, including human electrophysiology and microscopy, where BIDS has demonstrated its utility, a tractography-specific extension is needed toward reliable, reproducible, and robust tractography data representation and sharing. Note that the particular adoption of the BIDS specification for tractography should not prevent software from processing isolated tractography files. Feasibility: As of writing this article, the BIDS tractography extension is being drafted, and a small effort is needed to complete it.
4. Quality control. Standardized quality control procedures need to be fostered, including automated procedures at every stage of processing. Further research on the impact of different decisions in QC is needed to ultimately develop guidelines for best practices. Feasibility: Due to the challenges inherent to tractography validation, a moderate effort will be required to define consensus measures and procedures toward implementing standardized QC protocols and pipelines.
5. Make analysis tools that are flexible to be used across different data acquisition methods, different species, and different settings. FAIR-Software principles can be applied to make sure that methods are transparent, rigorously designed, and available to the community [142]. Feasibility: A substantial effort in validation is required to generalize tools across methods, species, and settings. This may include generating publicly shared, reproducible records of the datasets and procedures employed.
6. Scalability. Software and platforms for sharing and computing on brain connectivity data need to be built with the large datasets of the future in mind and with the ability to scale to much larger data than is currently available, in anticipation of the inevitable deluge of data that is expected to occur. Infrastructure for distributed and cloud computing includes evolving standards for representation of large array-based [143], tabular [144], and even trajectory data [145] in these kinds of systems, and formats for representation of tractography data could be adapted to capitalize on these developments. Feasibility: A moderate effort can be expected to design frameworks capable of handling large volumes of data. This effort is related to the development of data representation standards inherent to tractography and the reliance on the development of general-purpose, advanced scientific software.
7. Create and share annotated datasets for machine learning training. For improved interoperability and reproducibility, use industry standards for machine learning methods, such as ONNX, and widely used sharing platforms for ML/AI models and tools, such as Hugging Face. Feasibility: Both require minor efforts on the part of scientists and researchers who develop these methods.
8. Future-proof standardized file formats. The community needs to advance new file formats that address the needs for consistent, explicit tractography data spatial representation, while also supporting the needs for new large-scale datasets. This includes standardized, traceable, openly available, and well-documented specifications, including community-sustained conversion information and tools. We identify the nascent TRX file format as a format that has the potential to address many of the issues raised here, specifically because it was developed taking these aspects into consideration. Feasibility: A minor effort remains to be done in terms of documentation of the TRX file format. A few initial studies already provide evidence for the suitability of TRX for reliable and robust tractography data representation [105, 146]. A moderate effort will be required to incorporate the format as the output of existing software pipelines, which would make it more widely used.
9. Prioritize the articulation of clearly specified validation frameworks with common benchmarks and metrics, alongside the development of centralized, consensus evaluation tools. Importantly, these will enable an unambiguous assessment of relevant aspects like heterogeneous scanners, acquisition protocols, and imaging sources, critical to assess reliability and robustness of tractography. Feasibility: A substantial effort will be required to specify the resources—including data, metrics, and technological requirements—and to operationalize and sustain the resulting infrastructure.
10. Build the bridges between research and clinical tractography: Delivery of advanced methods into clinical practice can be facilitated by standardizing workflows and by increasing the interoperability between different parts of the clinical informatics infrastructure—for example, via integration of visualization into surgery image-guided systems, Picture Archiving and Communication Systems used in clinical settings, and electronic medical records [147]. This will also set the scene to improve the bench-to-bedside pipeline of new computational methods. Feasibility: While research and clinical requirements remain different, anatomically refined tractography methods can reach clinical practice through rigorous, coordinated validation and collaboration among researchers, clinicians, and vendors.

## Availability of Source Code and Requirements

Project name: Standardization-Position-Paper
Project homepage: https://github.com/International-Society-for-Tractography/Standardization-Position-Paper
Operating system: Not applicable
Programming language: Python
Other requirements: The data used to generate these plots are publicly available at https://osf.io/qcm7a/overview
License: Apache-2.0

## Abbreviations

3D: 3-dimensional; AI: artificial intelligence; BIDS: Brain Imaging Data Structure; CCF: Common Coordinate Framework; DICOM: Digital Imaging and Communications in Medicine; dMRI: diffusion magnetic resonance imaging; FAIR: Findable, Accessible, Interoperable, Reusable; GB: gigabyte; GPU: Graphics Processing Unit; ISMRM: International Society for Magnetic Resonance in Medicine; IST: International Society for Tractography; LPS: left, posterior, superior; ML: machine learning; MRI: magnetic resonance imaging; PACS: Picture Archiving and Communication Systems; QC: quality control; RAS: right, anterior, superior; SOP: standard operating procedures; TB: terabyte; TRX: TRactography eXchange.

## Supplementary Material

giag034_Authors_Response_To_Reviewer_Comments_original_submission

giag034_GIGA-D-25-00365_original_submission

giag034_GIGA-D-25-00365_Revision_1

giag034_GIGA-D-25-00365_Revision_2

giag034_Reviewer_1_Report_original_submission Reviewer 1 -- 10/9/2025

giag034_Reviewer_1_Report_revision_1 Reviewer 1 -- 2/17/2026

giag034_Reviewer_2_Report_original_submission Reviewer 2 -- 10/31/2025

giag034_Reviewer_2_Report_revision_1 Reviewer 2 -- 2/20/2026

## Data Availability

Not applicable.
