# Supplementary material for: What needs to be standardized for reliable, reproducible, and robust tractography?
Source: Gigascience. 2026 Mar 25;15:giag034. doi: 10.1093/gigascience/giag034 (PMC13137869; doi:10.1093/gigascience/giag034)
Supplement: giag034_GIGA-D-25-00365_original_submission [file giag034_giga-d-25-00365_original_submission.pdf]

GigaScience

What needs to be standardized for reliable, reproducible, and robust tractography?

--Manuscript Draft--

**Manuscript Number:**	GIGA-D-25-00365	
**Full Title:**	What needs to be standardized for reliable, reproducible, and robust tractography?	
**Article Type:**	Review	
**Funding Information:**	National Institute of Biomedical Imaging and Bioengineering (2R01EB027585-04A1)	PhD Wei Tang
	National Institute of Biomedical Imaging and Bioengineering (K01EB032898)	PhD Kurt Schilling
	National Institute of Mental Health (2R01MH112847)	PhD Matthew Cieslak
	National Institute of Mental Health (2R01MH120482)	PhD Matthew Cieslak
	National Institute of Mental Health (2R01MH113550)	PhD Matthew Cieslak
	HORIZON EUROPE European Research Council (101163214)	PhD Alberto De Luca
	Galen and Hilary Weston foundation	PhD Alberto De Luca
	Stichting Hanarth Fonds	PhD Alberto De Luca
	Division of Graduate Education (DGE-2140004)	Mr John Kruper
	Natural Sciences and Engineering Research Council of Canada	PhD Francois Rheault
	HORIZON EUROPE European Research Council (101000969)	Prof Stamatios N. Sotiropoulos
	Wellcome Trust (226486/Z/22/Z)	PhD Franco Pestilli
	National Institute of Neurological Disorders and Stroke (UM1NS132207)	PhD Franco Pestilli<br>PhD Sarah Heilbronner
	National Institute of Neurological Disorders and Stroke (U24NS140384)	PhD Franco Pestilli
	Royal Children's Hospital Foundation (RCHF 2022-1402)	MD, PhD Joseph Yuan-Mou Yang
	Kids' Cancer Project	MD, PhD Joseph Yuan-Mou Yang
	Université de Sherbrooke (Research Chair in Neuroinformatics)	Prof Maxime Descoteaux
	National Institute of Mental Health (MH121868)	PhD Ariel Rokem
	National Institute of Mental Health (MH121867)	PhD Ariel Rokem
	National Institute of Mental Health (R25MH112480)	PhD Ariel Rokem
	National Institute on Aging (R01AG060942)	PhD Ariel Rokem
	National Institute on Aging (U19AG066567)	PhD Ariel Rokem
	National Institute of Biomedical Imaging and Bioengineering (R01EB027585)	PhD Ariel Rokem
	National Science Foundation (1934292)	PhD Ariel Rokem
	National Science Foundation	PhD Ariel Rokem

Powered by Editorial Manager® and ProduXion Manager® from Aries Systems Corporation

	(2334483)	
	Chan Zuckerberg Initiative	PhD Ariel Rokem

**Abstract:**	Tractography is a key component of efforts to map brain connectivity. As a rapidly-evolving field of neuroscience, current tractography methods are diverse, often varying across research laboratories and different software pipelines. Therefore, it suffers from a lack of standardization leading to inconsistencies in results, which can limit reproducibility, and affect the robustness needed for research and clinical applications of these methods. Variability in data acquisition procedures, inconsistencies in spatial referencing schemes and implementations, and anatomical heterogeneity —at the individual level, across the lifespan, and across species— hinders comparative analyses. Additionally, the lack of consensus on best practices complicates the development of robust automated quality control pipelines and limits the clinical translation of tractography-based procedures. Establishing standardized protocols for acquisition, preprocessing, and tractography reconstruction are critical towards enabling reliable tract-specific analyses, facilitating cross-study harmonization, and supporting replicable large-scale population studies. The present article provides an overview of the current challenges in tractography standardization and identifies the key aspects that require standardization for reliable, reproducible, and robust tractography.
**Corresponding Author:**	Jon Haitz Legarreta<br>Brigham and Women's Hospital<br>Somerville, Massachusetts UNITED STATES
**Corresponding Author Secondary Information:**	
**Corresponding Author's Institution:**	Brigham and Women's Hospital
**Corresponding Author's Secondary Institution:**	
**First Author:**	Jon Haitz Legarreta
**First Author Secondary Information:**	
**Order of Authors:**	Jon Haitz Legarreta
	Simona Schiavi
	Wei Tang
	Garrett Banks
	Matthew Cieslak
	Kurt Schilling
	Alberto De Luca
	Jacques-Donald Tournier
	John Kruper
	Francois Rheault
	Stamatios N. Sotiropoulos
	Franco Pestilli
	Jelle Veraart
	Joseph Yuan-Mou Yang
	Maxime Descoteaux
	Sarah Heilbronner
	Ariel Rokem
**Order of Authors Secondary Information:**	

Powered by Editorial Manager® and ProduXion Manager® from Aries Systems Corporation

Additional Information:	
**Question**	**Response**
Are you submitting this manuscript to a special series or article collection?	No
**Experimental design and statistics**<br><br>Full details of the experimental design and statistical methods used should be given in the Methods section, as detailed in our Minimum Standards Reporting Checklist. Information essential to interpreting the data presented should be made available in the figure legends.<br><br>Have you included all the information requested in your manuscript?	Yes
**Resources**<br><br>A description of all resources used, including antibodies, cell lines, animals and software tools, with enough information to allow them to be uniquely identified, should be included in the Methods section. Authors are strongly encouraged to cite Research Resource Identifiers (RRIDs) for antibodies, model organisms and tools, where possible.<br><br>Have you included the information requested as detailed in our Minimum Standards Reporting Checklist?	Yes
**Availability of data and materials**<br><br>All datasets and code on which the conclusions of the paper rely must be either included in your submission or deposited in publicly available repositories (where available and ethically appropriate), referencing such data using a unique identifier in the references and in the “Availability of Data and Materials” section of your manuscript.	Yes

Powered by Editorial Manager® and ProduXion Manager® from Aries Systems Corporation

Have you have met the above requirement as detailed in our Minimum Standards Reporting Checklist?	
GigaScience has policies and guidelines in place for the use of generative AI-writing tools such as ChatGPT. If you have used such writing tools to assist with writing the manuscript this must be declared and cited in the text. Authors should not list AI-writing tools and other AI-assisted technologies as an author or co-author and should acknowledge that they are fully responsible for text generated or refined by AI-writing tools.<p><br><br>A summary of use (particularly in the introduction or among methods) needs to be included at the end of the paper, and the outputs should also be included as a supplementary file hosted in GigaDB or other open repositories. Please read our guidelines for more information. <p><br><br>By submitting to GigaScience, you are aware of the journal's AI-writing tools policy, and if you have declared use of such tools below, you have acknowledged this where appropriate in your manuscript and have made a summary of use and outputs available. <p><br><b>AI-assisted writing tools have been used in the preparation of this manuscript?	No

Powered by Editorial Manager® and ProduXion Manager® from Aries Systems Corporation

Click here to access/download;Manuscript;what_does_tractography_standar

GigaScience, 2023, 1–11

doi: xx.xxxx/xxxx
Manuscript in Preparation
Review

REVIEW

What needs to be standardized for reliable, reproducible, and robust tractography?

Jon Haitz Legarreta[1,2*], Simona Schiavi[3,4], Wei Tang[5], Garrett Banks[6], Matthew Cieslak[7,8,9], Kurt Schilling[10], Alberto De Luca[11], Jacques-Donald Tournier[12,13], John Kruper[14], Francois Rheault[15], Stamatios N. Sotiropoulos[16,17], Franco Pestilli[18,19], Jelle Veraart[20], Joseph Yuan-Mou Yang[21,22,23], Maxime Descoteaux[15], Sarah Heilbronner[6] and Ariel Rokem[14,24*]

[1]Department of Radiology, Brigham and Women's Hospital, Mass General Brigham, Boston, MA, USA and [2]Harvard Medical School, Boston, MA, USA and [3]ASG Superconductors S.p.A., Genoa, Italy and [4]Department of Computer Science, University of Verona, Verona, Italy and [5]Department of Psychological and Brain Sciences, Indiana University, Bloomington, IN, USA and [6]Department of Neurosurgery, Baylor College of Medicine, Houston, TX, USA and [7]Lifespan Informatics and Neuroimaging Center (PennLINC), Department of Psychiatry, Perelman School of Medicine, University of Pennsylvania, Philadelphia, PA, USA and [8]Penn/CHOP Lifespan Brain Institute, Perelman School of Medicine, Children's Hospital of Philadelphia Research Institute, Philadelphia, PA, USA and [9]Department of Psychiatry, Perelman School of Medicine, University of Pennsylvania, Philadelphia, PA, USA and [10]Department of Radiology, Vanderbilt University Medical Center, Nashville, TN, USA and [11]Image Sciences Institute, University Medical Center Utrecht, Utrecht, The Netherlands and [12]Department of Biomedical Engineering, School of Biomedical Engineering and Imaging Sciences, King's College London, King's Health Partners, St. Thomas' Hospital, London, UK and [13]Centre for the Developing Brain, School of Biomedical Engineering and Imaging Sciences, King's College London, King's Health Partners, St. Thomas' Hospital, London, UK and [14]Department of Psychology, University of Washington, Seattle, WA, USA and [15]Sherbrooke Connectivity Imaging Lab (SCIL), Department of Computer Science, Université de Sherbrooke, Sherbrooke, Québec, Canada and [16]Sir Peter Mansfield Imaging Centre, School of Medicine, University of Nottingham, Nottingham, UK and [17]NIHR Nottingham Biomedical Research Centre, School of Medicine, Queen's Medical Centre, Nottingham, UK and [18]Department of Psychology, The University of Texas at Austin, Austin, TX, USA and [19]Department of Neuroscience, The University of Texas at Austin, Austin, TX, USA and [20]School of Medicine, New York University, New York, NY, USA and [21]Department of Neurosurgery, Neuroscience Advanced Clinical Imaging Service (NACIS), Royal Children's Hospital, Melbourne, Victoria, Australia and [22]Neuroscience Research, Murdoch Children's Research Institute, Melbourne, Victoria, Australia and [23]Department of Paediatrics, University of Melbourne, Melbourne, Victoria, Australia and [24]The University of Washington eScience Institute, University of Washington, Seattle, WA, USA

*jhlegarreta@bwh.harvard.edu; arokem@uw.edu

Abstract

Tractography is a key component of efforts to map brain connectivity. As a rapidly-evolving field of neuroscience, current tractography methods are diverse, often varying across research laboratories and different software pipelines. Therefore, it suffers from a lack of standardization leading to inconsistencies in results, which can limit reproducibility, and affect the robustness needed for research and clinical applications of these methods. Variability in data acquisition procedures, inconsistencies in spatial referencing schemes and implementations, and anatomical heterogeneity —at the individual level, across the lifespan, and across species— hinders comparative analyses. Additionally, the lack of consensus on best practices complicates the development of robust automated quality control pipelines and limits the clinical translation of tractography-based procedures. Establishing standardized protocols for acquisition, preprocessing, and tractography reconstruction are critical towards enabling reliable tract-specific analyses, facilitating cross-study harmonization, and supporting replicable large-scale population studies. The present article provides an overview of the current challenges in tractography standardization and identifies the key aspects that require standardization for reliable, reproducible, and robust tractography.

Key words: Neuroanatomy; standardization; tractography; brain connectivity; white matter; computational neuroimaging

Background

Understanding the human brain anatomy across different organizational levels is a central goal of contemporary neuroscience. There is increasing evidence that structural connectivity across the white matter underlies many of the capacities of the living brain and that their physical properties are linked to human brain health [1, 2, 3, 4]. This understanding has grown with the development of methods to measure brain connections and it has also fueled a new generation of data collection and data analysis methods. Magnetic resonance imaging (MRI) measurements are non-invasive and can be used to study brain connectivity *in vivo* [5]. These data are complemented by other invasive techniques that highlight brain connections by using specific chemical tracers, histological stains and molecular markers, by dissecting the tissue, and by direct optical observation [6, 7, 8, 9, 10, 11, 12, 13], or using X-ray imaging methods [9, 14]. *Computational tractography* assesses the location, direction and pattern of brain connections estimating their trajectories (with an individual trajectory often referred to as a *streamline*). However, as the methods evolve and the promises from their application grow, it is important to take stock of challenges that hinder leveraging their full potential. Other works have examined challenges related to accurate delineation of brain connections [15, 16, 17, 18] or challenges related to definitions of certain anatomical concepts in such data [19, 20]. The present paper focuses specifically on the challenges related to standardizing representations of tractography-derived brain connections in digital formats.

Establishing standards and best practices supports transparent, reproducible and robust research through application of the FAIR (Findable, Accessible, Interoperable, Reusable) principles [21]. In addition, establishing usable standards can enable research that is otherwise difficult, making things that are hard easier, and making things that are otherwise impossible practical. With the recent establishment of the International Society for Tractography (IST) and its standardization unit (members of which are authors of this paper), together with several consensus efforts in diffusion MRI (dMRI) acquisition and processing [22, 18, 23] led by the Diffusion Study Group of the International Society for Magnetic Resonance in Medicine (ISMRM), we see an opportunity to advance broadly-applicable and widely-accepted community standards that will pave the way towards future research of brain connectivity.

This work introduces some of the current challenges that arise from gaps in standardization for tractography and offers some potential solutions to them. We provide a set of recommendations for the broader research community to pursue the solutions that we identify. The recommendations presented herein will improve the rigor and impact of work that uses brain tractography and will enable better understanding of brain connectivity. The paper is organized by the different stages of the data life-cycle and a range of domains in which standardization poses challenges (Figure 1), concluding with a set of recommendations (section Summary and recommendations).

Challenges and Solutions

Data acquisition

The utility of standards starts with the moment that data are created. Different experimental methods produce tractography data, and considerations for standardization may be different for each one of these. Furthermore, the creation of some data is already governed by numerous existing standards. For example, in a large number of cases, MRI data is acquired following the Digital Imaging and Communications in Medicine (DICOM) standard [24]. Similarly, modern storage and sharing of reconstructed (raw or processed) research MRI data is standardized by the Brain Imaging Data Structure (BIDS) specification [25], including several extensions for dMRI and brain connectivity (e.g., [26, 27]). These standards facilitate not only the structured storage of the data itself, but also prescribe necessary metadata. In principle, this should facilitate the standard processing in subsequent steps, but undocumented and poorly standardized procedures are typical of many different experimental techniques and a source of unwanted variability.

Differences across datasets can also arise due to a range of non-biological variables, such as variation in acquisition protocols, scanner hardware, reconstruction pipelines, and tractography workflows, that are hard to reconcile even given full description of the data acquisition. These sources of variability can have significant impact on downstream tractography results, particularly in multi-site studies and large-scale datasets. For example, acquisition resolution, diffusion sampling schemes, and vendor-specific differences introduce variability in the spatial geometry and microstructural characteristics of reconstructed brain tracts [28, 29, 30, 31, 32].

Statistical methods for *harmonization*, which aim to eliminate such differences at the level of the raw data [33], provide promising solutions to some of these challenges, but the intricacy of downstream tractography analysis workflows poses further challenges. These workflows involve subjective parameter choices, user-defined constraints, and possibly different priors or anatomical reference definitions that influence reproducibility and anatomical fidelity. Cross-scanner and cross-vendor effects propagate through these pipelines, leading to inconsistencies in reconstructed geometry, volume, and quantification of tract properties. Recent efforts to standardize processing pipelines and implement robust quality control protocols across sites have shown promise in reducing these inconsistencies [34, 35, 36, 37], but further work is needed to develop automated and reproducible methods for harmonizing tractography.

Quality control

There are many well-established pipelines for quality control (QC) of raw MRI data [38], including dMRI data [39, 40], as well as data that has undergone initial pre-processing (i.e., denoising, correction of motion and eddy current artifacts, and removal of other artifacts) [41, 42, 43]. Presumably, QC procedures that are applied at these early stages should catch many of the issues that would impact subsequent analysis steps. However, QC can and should be done at multiple different stages of the analysis, because errors can occur at each one: e.g., registration between different imaging modalities, separation of the image into different tissue types and regions of interest for tractography initiation, etc. Often times, many of these issues only become apparent when computational tractography is conducted. This means that QC of tractography results is still necessary. In practice, QC of computational tractography pipelines is often done through visual examination of whole-brain tractograms (the collection of all streamlines estimated in a single brain), or by their ability to identify the locations and trajectories of large, well-known tracts. However, this approach is challenged by low inter-rater variability and an apparent lack of consensus on the structure of these tracts, even in the same set of streamlines [19, 20]. Additionally, although there are many advanced tractography visualization tools and paradigms [44], and despite some pipelines offering automated reporting, their use for QC purposes has not been thoroughly studied. As a result, best practices for ex-

Compiled on: September 4, 2025.
Draft manuscript prepared by the author.

cluding low-quality results based on these visualizations remain unclear.

Another approach for QC of tractography results is through numerical validation. In this approach, individual streamlines and sets of streamlines are subjected to statistical evaluation with respect to the measured data. In these methods [45, 46, 47, 48, 49, 50] individual streamlines or sets of streamlines are given objective scores, based on how well their trajectory conforms to the data that was used to generate the streamlines (e.g., in contrast to smoothness constraints, anatomical constraints, or randomness that is introduced in the process of tractography). Other methods use machine learning and deep learning techniques to filter streamlines based on their geometric properties or a representation of them [51, 52]. The benefit of these approaches is that they provide objective numerical values that can then be used as benchmarks for QC procedures. Overall, the field would benefit from more studies that demonstrate the utility and sensitivity of different QC procedures in relevant scientific inferences [53, 54], including validation by reference to known anatomical structure, and via the detection of individual differences related to development, aging, or clinical conditions [55].

Spatial coordinates

Representing spatial coordinates in an unequivocal frame is a known challenge in many fields of science that deal with spatial information. This is a well-recognized issue in anatomical studies of the human brain, where different types of information (e.g., structural, functional, physiological, etc.) need to be integrated. Given the variety of information sources that are involved, each acquisition modality having particular spatial properties, the potential for errors is high and the consequences detrimental to the study of brain connectivity [56].

Tractography algorithms typically output data as a set of three-dimensional (3D) coordinates that represent the trajectory of particular brain white matter tracts (see Figure 2A,B). In reconstructing and interpreting these structures, several coordinate frames need to be considered. One coordinate frame is the one that refers only to the individual participant's brain, allowing to locate a structure relative to a particular anatomical landmark. Another coordinate frame is the position of the individual's brain within the scanner, i.e., relative to the origin of the scanner's fixed reference frame (e.g., the iso-center of the MRI's bore). Another coordinate frame to consider is that of the grid of *voxels* that are included in the measurement itself.

Volumetric imaging data follows particular anatomical conventions that facilitate relative positioning and orientation. These conventions split the space according to three orthogonal planes, namely *axial*, *coronal*, and *sagittal* planes (Figure 2A). When storing real-world spatial data into discrete imaging data, the information can be arranged according to a particular basis. Two typical conventions are *RAS+* (Right, Anterior, Superior, the + sign indicating that coordinates increase from left to right, posterior to anterior, and inferior to superior) and *LPS+* (Left, Posterior, Superior; the same principle applying to the + sign). If no metadata is accessible, applications reading tractography results may assume the incorrect convention (Figure 2C).

At times, the conventions implied by a particular format are not made apparent in the corresponding documentation. A typical example of this issue is the definition of the origin of a voxel: some tractography formats consider the origin being at the center of the voxel, whereas others consider that it lies at the corner. This may arise in the correspondence between streamline coordinates and other coordinate-based (e.g., volume-based) data: unless this is carefully considered, a half-voxel shift will exist between the streamlines relative to their intended world coordinates. Visual inspection is not always sufficient to notice this (Figure 2D), and such small systematic errors can have significant consequences in downstream applications.

Finally, streamlines generated by tractography algorithms may have their own positions relative to each of the other coordinate frames, and may be tied to an additional piece of data (e.g., another data file). The units used to store their position may also differ from the units used to refer to the measurement (discrete voxels, or continuous mm). Incongruent coordinate frames and units can lead to errors such as the ones shown in Figure 2E.

Translation across species and methods

Anatomical tract-tracing methods involve injecting a dye or tracer into a living brain, waiting a set period of time, then extracting the brain for histology and microscopy [57]. Thus, these methods provide information that is not accessible with MRI but, for obvious reasons, cannot be performed in humans. Thus, tract-tracing is performed in brains from non-human animals, such as primates and rodents. Combining these methods with dMRI in non-human models provides a unique opportunity to verify dMRI-based tractography against anatomical gold standards, and to iteratively improve these methods. Several large-scale efforts aim to provide high-quality dMRI along with microscopy and tracers in the same non-human primate brains (e.g., the Large-scale imaging of neural circuit (LINC) BRAIN CONNECTS center, or the Center for mesoscale connectomics (CMC)). Openly accessible resources from these consortia and others (such as PRIME-DE [58], or the BRAIN/MINDS portal [59] will be invaluable in optimizing, standardizing, and developing the next generation of tractography approaches. Using unified methods that combine these multi-modal and multi-scale data would ultimately enable informed judgment about cross-species homologies/dishomologies. However, this process is complicated by several factors. Non-human brains —even in our closest primate relatives— are not simply smaller, but also have different morphological and biophysical properties. Integration of data between methods is also hampered by differences in data representation: anatomical structures (gray/white matter boundaries, nuclei volumes, cells, fibers, etc.) are represented in standard anatomical reconstruction software (e.g., Neurolucida) that interface with microscopes as points, contours and surfaces. Downstream processing software such as IMOD [60] inherits the file formats that organize these structures. This is conceptually different from the MRI file conventions, which use voxels as their primary representational unit.

Integration between human and non-human primate data also remains conceptually challenging. Non-human anatomical knowledge relies heavily on atlases based on cytoarchitectonics (e.g., [61]). These do not directly align with the common atlases used in human MRI studies (e.g., [62]). While cytoarchitectonics-based human brain atlases have been developed [63], some even with a particular stress on cross-species homology [64], these have not been integrated with widely used neuroimaging tools. Tractography fingerprinting [65], i.e., using white matter bundles as latent landmarks to assess similarities and divergences across the two species, is geometry-agnostic and can provide solutions to this challenge.

Integration with studies in rodents are further complicated by the extent of species dishomology. Their white matter bundles are organized very differently from primates' [66], and their lissencephalic brains mean that many of the MRI-based tractography methods that work in primate brains do not translate. On the other hand, there are a host of genetic, molecular, and imaging tools readily available for circuit characterization and manipulation in mice that are not feasible in humans or non-human primates. Thus, merging dMRI tractography with tract-tracing, spatial transcriptomics, and imaging data in the mouse model could be highly valuable. However, while anatomical atlases in primate models have a long history, just two decades ago, there were virtually no stan-

dard frameworks (atlases, metadata, structural ontologies, etc.) for the mouse brain. However, with major investments in generating large-scale datasets of mouse brain transcriptomics, cell typing, connectivity, and function, the need for a standard framework became overwhelming. The Allen Mouse Brain Common Coordinate Framework (CCF) [67] (current version CCFv3 [68]) has emerged as a standard framework for organizing data about mouse brain connectivity. The enormous success of this framework illustrates the value of standardization. An extensive informatics pipeline at the Allen Institute for Brain Science allows users to download everything from raw images to structural summaries of connectivity, and tools that use the CCF can integrate data from multiple sources. However, these tools are not currently interoperable with MRI data, aside from at the crudest (region-to-region connectivity summaries) level.

The scale of data

Like many other datasets of spatial biological information, tractography data can take up large amounts of storage. A typical dMRI tractography file occupies several gigabytes (GB) of data. The size depends on the sampling density employed to reconstruct streamlines, the number of streamlines that were generated, the data associated with each point or each streamline (e.g., scalars that represent tissue properties along their length etc.), as well as the precision employed to store the data. Additionally, the size of the tractography output may also depend on the resolution of the acquisition. For example, with sub-millimeter resolution MRI acquisitions, the size of tractograms can easily reach the terabyte-scale (TB) [69]. With other modalities that provide even higher image resolution in *ex vivo* tissue samples, such as polarization microscopy [7, 8, 10, 12], X-ray microcomputed tomography [70], X-ray scattering [9], or synchrotron technology [14], data is orders of magnitude larger (see, for example, [71, 72]). Manipulating such data volumes pose a challenge in terms of the required hardware and computational power. As imaging acquisition hardware evolves, and data storage capacity and physical memory limits increase, current analysis tools may buckle under the strain, and efforts will be required to address these challenges. A few technical and standardization developments may ease this strain. First, tractography data lends itself to compression, with *linearization*, where collinear points are discarded (with some tolerance threshold), being the fastest and most efficient approach [73, 74, 75]. Additional compressions is provided by using lower precision numerical representations (i.e., 16-bit floating point precision, instead of the extended 64), which can provide significant space saving, without much loss of anatomical information [76]. Similarly, some visualization techniques such as level of detail, occlusion culling or visibility-based rendering optimization aim to save computational resources by avoiding rendering data that is occluded by other objects or by rendering it at lower resolutions. Second, new file formats and distributed computing paradigms offer opportunities to scale compute to very large datasets. As an example, a newly-proposed file format, *TRactography eXchange* (TRX) [77], was designed to make it easy to create large tractograms with minimal random access memory requirements. This enables memory mapping, providing a convenient and efficient way to access data directly from disk.

Translation of tractography methods to clinical applications

Tractography is used clinically to aid in the planning and execution of neurosurgical procedures [78]. One use is during the resection of epileptic foci, or brain tumors [79, 80]. In this case, a surgeon might utilize a different surgical approach to the tumor to avoid certain white matter tracts, particularly those involved with motor, language and visual function. In other instances, tractography is utilized for precision targeting in stereotactic procedures such as deep brain stimulation and focused ultrasound, such as when localizing the dentatorubrothalamic tract, a neuromodulation target for treatment of essential tremor and tremor-dominant Parkinson's disease [81, 82, 83].

Standardization of clinical workflows is complicated, because these applications require thorough validation and consensus among experts. This requires processing software to remain very stable over time, and makes updates to the software very difficult in these settings. Variable imaging protocols also make consensus more difficult to reach (see section Data acquisition). Benchmarks and standard validations methods are very challenging to formulate, because clinical use-cases are diverse, and there are concerns that methods may be affected by the presence of pathologies. Furthermore, clinicians (e.g., radiologists or neurosurgeons) may use bespoke pipelines developed in-house to perform tractography, making translation across settings difficult. Finally, depending on the clinical setting and operative urgency, some processing pipelines that are common in research settings may be too time consuming and/or computationally intensive. To help bridge basic research and clinical work, acquisition and processing protocols need to be made more consistent across settings.

Standardization of methods throughout the lifespan

The brain changes significantly throughout the lifespan, and there is a wealth of evidence that lifespan development of brain connections is linked to health outcomes (see [84] for a review). The changes in the properties of the brain also pose challenges for standardization of methods across different epochs of the life. For example, studying early life brain development is challenged by the simple fact that the brain is substantially smaller at birth, with total brain volume more than doubling during the first 12 months of life [85]. This poses a particular challenge to the use of standard atlases that localize certain structures, as these atlases are usually constructed based on healthy young brains. To address this challenge, researchers have been developing detailed and time-resolved atlases in early life [86, 87] and even during gestation [88]. Furthermore, while the major brain tracts are already established at birth [89], it is not simply the case that the infant brain is a scaled-down version of the adult brain. There is some evidence that the curvature of some structures is different in this early phase relative to later development [90]. In addition, the tissue properties of brain connections in early life are quite different than those of a more mature brain. This can pose a challenge to the use of standard tractography methods, which sometimes rely on assumptions about the biophysics of the tissue, which may not hold [91]. Similar changes apply in aging, as tissue properties of the white matter change again with age in a manner that can impact standard tractography methods [92]. The challenge of studying the developing and aging brain often intersects with challenges related to harmonization (mentioned in section Data acquisition) because it is difficult to obtain a large sample that covers all ages within a single study, necessitating integration of data across studies and differing acquisitions.

Summary and recommendations

i. Implement standard operating procedures (SOPs) [93] during data acquisition, i.e., written sets of instructions that specify the processes that take place during the measurement.
ii. Adopt vendor-agnostic, open-source pulse sequence design and reconstruction frameworks (e.g., Pulseq [94, 95]) to facilitate more similar results across the instruments of different vendors.
iii. Advance BIDS-compliant standards for tractography inputs and outputs. Akin to a wide range of neuroimaging use-cases, including human electrophysiology and microscopy, where BIDS

has demonstrated its utility, a tractography-specific extension is needed towards reliable, reproducible, and robust tractography data representation and sharing.

iv. Quality control. Standardized quality control procedures need to be fostered including automated procedures at every stage of processing. Further research on the impact of different decisions in QC is needed, to ultimately develop guidelines for best practices.

v. Make analysis tools that are flexible to be used across different data acquisition methods, different species and different settings. FAIR-Software principles can be applied, to make sure that methods are transparent, rigorously designed and available to the community [96].

vi. Scalability. Software and platforms for sharing and computing on brain connectivity data need to be built with the large datasets of the future in mind, and with the ability to scale to much larger data than is currently available, in anticipation of the inevitable deluge of data that is expected to occur. Infrastructure for distributed and cloud computing includes evolving standards for representation of large array-based [97], tabular [98], and even trajectory data [99] in these kinds of systems, and formats for representation of tractography data could be adapted to capitalize on these developments.

vii. Future-proof standardized file formats. The community needs to advance new file formats that address the needs for consistent explicit tractography data spatial representation, while also supporting the needs for new large-scale datasets. This includes standardized, traceable, openly available, and well-documented specifications, including community-sustained conversion information and tools. We identify the nascent TRX file-format as a format that has the potential to address many of the issues raised here, specifically because it was developed taking these aspects into consideration.

viii. Build the bridges between research and clinical tractography: Delivery of advanced methods into clinical practice can be facilitated by standardizing workflows, and by increasing the interoperability between different parts of the clinical informatics infrastructure. For example, via integration of visualization into surgery image-guided systems, into Picture Archiving and Communication Systems (PACS) used in clinical, and into electronic medical records [100]. This will also set the scene to improve the bench-to-bedside pipeline of new computational methods.

Data availability

Not applicable.

Code availability

The plots in Panels a, c, d and e in Figure 2 were created based on actual human brain *in vivo* diffusion MRI tractography data cases and the software openly available at https://github.com/International-Society-for-Tractography/Standardization-Position-Paper under an Apache 2.0 license.

Declarations

List of abbreviations

3D: three-dimensional; BIDS: Brain Imaging Data Structure; CCF: Common Coordinate Framework; DICOM: Digital Imaging and Communications in Medicine; dMRI: diffusion magnetic resonance imaging; FAIR: Findable, Accessible, Interoperable, Reusable; GB: gigabyte; ISMRM: International Society for Magnetic Resonance in Medicine; IST: International Society for Tractography; LPS: Left, Posterior, Superior; MRI: magnetic resonance imaging; PACS: Picture Archiving and Communication Systems; QC: quality control; RAS: Right, Anterior, Superior; SOP: standard operating procedures; TB: terabyte; TRX: TRactography eXchange

Ethical Approval

Not applicable.

Consent for publication

Not applicable.

Competing Interests

S.S. is an employee of ASG Superconductors; M.D. is shareholder at Imeka Solutions Inc.

Funding

W.T. is supported by the National Institute of Biomedical Imaging and Bioengineering (NIBIB) grant 2R01EB027585-04A1; K.S. is supported by grant NIH K01EB032898; M.C. is supported through NIH grants 2R01MH112847, 2R01MH120482, and 2R01MH113550; A.D.L. is supported by a Starting Grant from the European Research Council (agreement 101163214), the Galen and Hilary Weston foundation, and Stichting Hanarth Fonds; J.K. is supported by NSF Graduate Research Fellowship DGE-2140004; F.R. acknowledges support of the Natural Sciences and Engineering Research Council of Canada (NSERC); S.N.S is supported by a European Research Council Consolidator Grant (101000969); F.P. is supported by Wellcome Trust (grant no. 226486/Z/22/Z, Principal Investigator F. Pestilli), NINDS UM1NS132207, BRAIN CONNECTS: Center for Mesoscale Connectomics (Principal Investigator K. Ugurbil), and NINDS U24NS140384, BRAIN CONNECTS: The Axonal Projectome EXchange (APEX) (Principal Investigator F. Pestilli); J.Y.-M.Y acknowledges position funding support from the Royal Children's Hospital Foundation (RCHF 2022-1402), and support from The Kids' Cancer Project (TKCP) Col Reynolds Fellowship; M.D. is supported by the Université de Sherbrooke research chair in Neuroinformatics; S.H. is supported by grant NIH UM1NS132207; A.R.'s work is funded by National Institutes of Health grants MH121868, MH121867, R25MH112480, R01AG060942, and U19AG066567, and R01EB027585, as well as by National Science Foundation grants 1934292 and 2334483, and by the Chan Zuckerberg Initiative's Essential Open Source Software for Science program.

Author's Contributions

J.H.L., F.R., and A.R. developed the code that served as the basis for the plots in Panels a, c, d and e in Figure 2. All authors discussed the content, reviewed and wrote the paper.

Acknowledgements

Not applicable.

References

1. Thiebaut de Schotten M, Forkel SJ. The emergent properties of the connected brain. Science (New York, NY) 2022;378(6619):505–510. http://dx.doi.org/10.1126/science.abq2591.
2. Fields RD. White matter in learning, cognition and psychiatric

disorders. Trends in neurosciences 2008;31(7):361–370. http://dx.doi.org/10.1016/j.tins.2008.04.001.
3. Passingham RE, Stephan KE, Kötter R. The anatomical basis of functional localization in the cortex. Nature reviews Neuroscience 2002;3(8):606–616. http://dx.doi.org/10.1038/nrn893.
4. Wandell BA. Clarifying Human White Matter. Annual review of neuroscience 2016 1 Apr;http://dx.doi.org/10.1146/annurev-neuro-070815-013815.
5. Jbabdi S, Sotiropoulos SN, Haber SN, Van Essen DC, Behrens TE. Measuring macroscopic brain connections in vivo. Nat Neurosci 2015 Nov;18(11):1546–1555.
6. Schmahmann JD, Petrides M, Pandya DN. Fiber pathways of the brain. New York, NY: Oxford University Press; 2006.
7. Axer M, Strohmer S, Gräßel D, Bücker O, Dohmen M, Reckfort J, et al. Estimating fiber orientation distribution functions in 3D-Polarized Light Imaging. Frontiers in neuroanatomy 2016 19 Apr;10:40. http://dx.doi.org/10.3389/fnana.2016.00040.
8. Lefebvre J, Delafontaine-Martel P, Pouliot P, Girouard H, Descoteaux M, Lesage F. Fully automated dual-resolution serial optical coherence tomography aimed at diffusion MRI validation in whole mouse brains. Neurophotonics 2018 Oct;5(4):045004. http://dx.doi.org/10.1117/1.NPh.5.4.045004.
9. Menzel M, Gräßel D, Rajkovic I, Zeineh MM, Georgiadis M. Using light and X-ray scattering to untangle complex neuronal orientations and validate diffusion MRI. eLife 2023 11 May;12. http://dx.doi.org/10.7554/eLife.84024.
10. Mollink J, Kleinnijenhuis M, van Cappellen van Walsum AM, Sotiropoulos SN, Cottaar M, Mirfin C, et al. Evaluating fibre orientation dispersion in white matter: Comparison of diffusion MRI, histology and polarized light imaging. NeuroImage 2017 15 Aug;157:561–574. http://dx.doi.org/10.1016/j.neuroimage.2017.06.001.
11. Schurr R, Mezer AA. The glial framework reveals white matter fiber architecture in human and primate brains. Science (New York, NY) 2021 5 Nov;374(6568):762–767. http://dx.doi.org/10.1126/science.abj7960.
12. Wang H, Lenglet C, Akkin T. Structure tensor analysis of serial optical coherence scanner images for mapping fiber orientations and tractography in the brain. Journal of biomedical optics 2015 Mar;20(3):036003. http://dx.doi.org/10.1117/1.JBO.20.3.036003.
13. Xu F, Shen Y, Ding L, Yang CY, Tan H, Wang H, et al. High-throughput mapping of a whole rhesus monkey brain at micrometer resolution. Nature biotechnology 2021 26 Dec;39(12):1521–1528. http://dx.doi.org/10.1038/s41587-021-00986-5.
14. Kjer HM, Andersson M, He Y, Pacureanu A, Daducci A, Pizzolato M, et al. Bridging the 3D geometrical organisation of white matter pathways across anatomical length scales and species. eLife 2025 28 Feb;13. http://dx.doi.org/10.7554/eLife.94917.
15. Girard G, Rafael-Patiño J, Truffet R, Aydogan DB, Adluru N, Nair VA, et al. Tractography passes the test: Results from the diffusion-simulated connectivity (disco) challenge. NeuroImage 2023 15 Aug;277(120231):120231. http://dx.doi.org/10.1016/j.neuroimage.2023.120231.
16. Maffei C, Girard G, Schilling KG, Aydogan DB, Adluru N, Zhylka A, et al. Insights from the IronTract challenge: Optimal methods for mapping brain pathways from multi-shell diffusion MRI. NeuroImage 2022 15 Aug;257(119327):119327. http://dx.doi.org/10.1016/j.neuroimage.2022.119327.
17. Maier-Hein KH, Neher PF, Houde JC, Côté MA, Garyfallidis E, Zhong J, et al. The challenge of mapping the human connectome based on diffusion tractography. Nature communications 2017 7 Nov;8(1):1349. http://dx.doi.org/10.1038/s41467-017-01285-x.
18. Schilling KG, Grussu F, Ianus A, Hansen B, Howard AFD, Barrett RLC, et al., Considerations and recommendations from the ISMRM Diffusion Study Group for preclinical diffusion MRI: Part 2 – Ex vivo imaging: added value and acquisition; 2022. http://arxiv.org/abs/2209.13371.
19. Rheault F, De Benedictis A, Daducci A, Maffei C, Tax CMW, Romascano D, et al. Tractostorm: The what, why, and how of tractography dissection reproducibility. Human brain mapping 2020;41(7):1859–1874. http://dx.doi.org/10.1002/hbm.24917.
20. Schilling KG, Rheault F, Petit L, Hansen CB, Nath V, Yeh FC, et al. Tractography dissection variability: What happens when 42 groups dissect 14 white matter bundles on the same dataset? NeuroImage 2021 1 Nov;243(118502):118502. http://dx.doi.org/10.1016/j.neuroimage.2021.118502.
21. Wilkinson MD, Dumontier M, Aalbersberg IJJ, Appleton G, Axton M, Baak A, et al. The FAIR Guiding Principles for scientific data management and stewardship. Scientific data 2016 15 Mar;3:160018. http://dx.doi.org/10.1038/sdata.2016.18.
22. Jelescu IO, Grussu F, Ianus A, Hansen B, Barrett RLC, Aggarwal M, et al., Considerations and Recommendations from the ISMRM Diffusion Study Group for preclinical diffusion MRI: Part 1 – In vivo small-animal imaging; 2022. http://arxiv.org/abs/2209.12994.
23. Schilling KG, Howard AFD, Grussu F, Ianus A, Hansen B, Barrett RLC, et al., Considerations and recommendations from the ISMRM Diffusion Study Group for preclinical diffusion MRI: Part 3 – Ex vivo imaging: data processing, comparisons with microscopy, and tractography; 2024. http://arxiv.org/abs/2411.05021.
24. Mustra M, Delac K, Grgic M. Overview of the DICOM standard. In: 2008 50th International Symposium ELMAR, vol. 1 Zadar, Croatia: IEEE; 2008. p. 39–44. https://ieeexplore.ieee.org/abstract/document/4747434/.
25. Gorgolewski KJ, Auer T, Calhoun VD, Cameron Craddock R, Das S, Duff EP, et al. The brain imaging data structure, a format for organizing and describing outputs of neuroimaging experiments. Scientific data 2016 21 Jun;3(1):1–9. https://www.nature.com/articles/sdata201644.
26. Gholam J, Szczepankiewicz F, Tax CMW, Mueller L, Kopanoglu E, Nilsson M, et al., aDWI-BIDS: an extension to the brain imaging data structure for advanced diffusion weighted imaging; 2021. http://arxiv.org/abs/2103.14485.
27. Pestilli F, Poldrack R, Rokem A, Satterthwaite T, Feingold F, Duff E, et al. A community-driven development of the brain imaging data standard (BIDS) to describe macroscopic brain connections 2021;.
28. Cai LY, Yang Q, Kanakaraj P, Nath V, Newton AT, Edmonson HA, et al. MASiVar: Multisite, multiscanner, and multisubject acquisitions for studying variability in diffusion weighted MRI. Magnetic resonance in medicine 2021 1 Dec;86(6):3304–3320. http://dx.doi.org/10.1002/mrm.28926.
29. Gajwani M, Oldham S, Pang JC, Arnatkevičiūtė A, Tiego J, Bellgrove MA, et al. Can hubs of the human connectome be identified consistently with diffusion MRI? Network neuroscience (Cambridge, Mass) 2023 22 Dec;7(4):1326–1350. http://dx.doi.org/10.1162/netn_a_00324.
30. Schilling KG, Tax CMW, Rheault F, Hansen C, Yang Q, Yeh FC, et al. Fiber tractography bundle segmentation depends on scanner effects, vendor effects, acquisition resolution, diffusion sampling scheme, diffusion sensitization, and bundle segmentation workflow. NeuroImage 2021 15 Nov;242:118451. http://dx.doi.org/10.1016/j.neuroimage.2021.118451.
31. Tong Q, He H, Gong T, Li C, Liang P, Qian T, et al. Reproducibility of multi-shell diffusion tractography on traveling subjects: A multicenter study prospective. Magnetic resonance imaging 2019 1 Jun;59:1–9. http://dx.doi.org/10.1016/j.mri.2019.

02.011.
32. Warrington S, Torchi A, Mougin O, Campbell J, Ntata A, Craig M, et al. A multi-site, multi-modal travelling-heads resource for brain MRI harmonisation. Sci Data 2025 Apr;12(1):609.
33. Pinto MS, Paolella R, Billiet T, Van Dyck P, Guns PJ, Jeurissen B, et al. Harmonization of Brain Diffusion MRI: Concepts and Methods. Front Neurosci 2020 May;14:396.
34. Moyer D, Ver Steeg G, Tax CMW, Thompson PM. Scanner invariant representations for diffusion MRI harmonization. Magnetic resonance in medicine 2020 1 Oct;84(4):2174–2189. http://dx.doi.org/10.1002/mrm.28243.
35. Ning L, Bonet-Carne E, Grussu F, Sepehrband F, Kaden E, Veraart J, et al. Muti-shell diffusion MRI harmonisation and enhancement challenge (MUSHAC): Progress and results. In: Computational Diffusion MRI Mathematics and visualization, Cham: Springer International Publishing; 2019.p. 217–224.
36. Tax CM, Grussu F, Kaden E, Ning L, Rudrapatna U, John Evans C, et al. Cross-scanner and cross-protocol diffusion MRI data harmonisation: A benchmark database and evaluation of algorithms. NeuroImage 2019 15 Jul;195:285–299. http://dx.doi.org/10.1016/j.neuroimage.2019.01.077.
37. Warrington S, Ntata A, Mougin O, Campbell J, Torchi A, Craig M, et al. A resource for development and comparison of multimodal brain 3T MRI harmonisation approaches. Imaging Neurosci (Camb) 2023 Nov;1:1–27.
38. Esteban O, Birman D, Schaer M, Koyejo OO, Poldrack RA, Gorgolewski KJ. MRIQC: Advancing the automatic prediction of image quality in MRI from unseen sites. PloS one 2017 25 Sep;12(9):e0184661. http://dx.doi.org/10.1371/journal.pone.0184661.
39. Bastiani M, Cottaar M, Fitzgibbon SP, Suri S, Alfaro-Almagro F, Sotiropoulos SN, et al. Automated quality control for within and between studies diffusion MRI data using a non-parametric framework for movement and distortion correction. NeuroImage 2019 1 Jan;184:801–812. http://dx.doi.org/10.1016/j.neuroimage.2018.09.073.
40. Hagen MP, Provins C, MacNicol E, Li J, Gomez T, Garcia M, et al., Quality assessment and control of unprocessed anatomical, functional, and diffusion MRI of the human brain using MRIQC; 2024.
41. Cieslak M, Cook PA, He X, Yeh FC, Dhollander T, Adebimpe A, et al. QSIPrep: an integrative platform for preprocessing and reconstructing diffusion MRI data. Nature methods 2021;18(7):775–778. http://dx.doi.org/10.1038/s41592-021-01185-5.
42. Cirstian R, Forde NJ, Andersson JLR, Sotiropoulos SN, Beckmann CF, Marquand AF. Objective QC for diffusion MRI data: Artefact detection using normative modelling. Imaging Neuroscience 2024 26 Apr;2:1–14. https://dx.doi.org/10.1162/imag_a_00144.
43. Richie-Halford A, Cieslak M, Ai L, Caffarra S, Covitz S, Franco AR, et al. An analysis-ready and quality controlled resource for pediatric brain white-matter research. Scientific data 2022 12 Oct;9(1):616. http://dx.doi.org/10.1038/s41597-022-01695-7.
44. Laamoumi M, Hendriks T, Chamberland M. A taxonomic guide to diffusion MRI tractography visualization tools. NMR in Biomedicine 2025 7 Jan;38(1):e5267. http://dx.doi.org/10.1002/nbm.5267.
45. Smith RE, Tournier JD, Calamante F, Connelly A. SIFT: Spherical-deconvolution informed filtering of tractograms. NeuroImage 2013 15 Feb;67:298–312. http://dx.doi.org/10.1016/j.neuroimage.2012.11.049.
46. Smith RE, Tournier JD, Calamante F, Connelly A. SIFT2: Enabling dense quantitative assessment of brain white matter connectivity using streamlines tractography. NeuroImage 2015 1 Oct;119:338–351. http://dx.doi.org/10.1016/j.neuroimage.2015.06.092.
47. Daducci A, Palù AD, Lemkaddem A, Thiran J. COMMIT: Convex optimization modeling for microstructure informed tractography. IEEE transactions on medical imaging 2015 1 Jan;34:246–257. http://dx.doi.org/10.1109/TMI.2014.2352414.
48. Schiavi S, Ocampo-Pineda M, Barakovic M, Petit L, Descoteaux M, Thiran JP, et al. A new method for accurate in vivo mapping of human brain connections using microstructural and anatomical information. Science Advances 2020 31 Jul;6(31). http://dx.doi.org/10.1126/sciadv.aba8245.
49. Caiafa CF, Pestilli F. Multidimensional encoding of brain connectomes. Scientific reports 2017 13 Sep;7(1):11491. http://dx.doi.org/10.1038/s41598-017-09250-w.
50. Pestilli F, Yeatman JD, Rokem A, Kay KN, Wandell BA. Evaluation and statistical inference for human connectomes. Nature methods 2014 Oct;11(10):1058–1063. http://dx.doi.org/10.1038/nmeth.3098.
51. Astolfi P, Verhagen R, Petit L, Olivetti E, Sarubbo S, Masci J, et al. Supervised tractogram filtering using Geometric Deep Learning. Medical image analysis 2023 1 Dec;90(102893):102893. http://dx.doi.org/10.1016/j.media.2023.102893.
52. Legarreta JH, Petit L, Rheault F, Theaud G, Lemaire C, Descoteaux M, et al. Filtering in Tractography using Autoencoders (FINTA). Medical image analysis 2021 7 Jun;p. 102126. http://dx.doi.org/10.1016/j.media.2021.102126.
53. Sarwar T, Ramamohanarao K, Zalesky A. A critical review of connectome validation studies. NMR in biomedicine 2021 1 Dec;34(12):e4605. http://dx.doi.org/10.1002/nbm.4605.
54. Sarwar T, Ramamohanarao K, Daducci A, Schiavi S, Smith RE, Zalesky A. Evaluation of tractogram filtering methods using human-like connectome phantoms. NeuroImage 2023 1 Nov;281(120376):120376. http://dx.doi.org/10.1016/j.neuroimage.2023.120376.
55. Zalesky A, Sarwar T, Ramamohanarao K. A cautionary note on the use of SIFT in pathological connectomes. Magnetic resonance in medicine 2020 1 Mar;83(3):791–794. http://dx.doi.org/10.1002/mrm.28037.
56. Glen DR, Taylor PA, Buchsbaum BR, Cox RW, Reynolds RC. Beware (surprisingly common) left-right flips in your MRI data: An efficient and robust method to check MRI dataset consistency using AFNI. Frontiers in neuroinformatics 2020 25 May;14:18. http://dx.doi.org/10.3389/fninf.2020.00018.
57. Lanciego JL, Wouterlood FG. Neuroanatomical tract-tracing techniques that did go viral. Brain Struct Funct 2020 May;225(4):1193–1224.
58. Milham MP, Ai L, Koo B, Xu T, Amiez C, Balezeau F, et al. An open resource for non-human primate imaging. Neuron 2018 10 Oct;100(1):61–74.e2. http://dx.doi.org/10.1016/j.neuron.2018.08.039.
59. Hata J, Nakae K, Tsukada H, Woodward A, Haga Y, Iida M, et al. Multi-modal brain magnetic resonance imaging database covering marmosets with a wide age range. Scientific data 2023 27 Apr;10(1):221. http://dx.doi.org/10.1038/s41597-023-02121-2.
60. Kremer JR, Mastronarde DN, McIntosh JR. Computer visualization of three-dimensional image data using IMOD. Journal of structural biology 1996 1 Jan;116(1):71–76. http://dx.doi.org/10.1006/jsbi.1996.0013.
61. Paxinos G, Petrides M, Evrard HC. The rhesus monkey brain in stereotaxic coordinates. 4 ed. San Diego, CA: Academic Press; 2023.
62. Desikan RS, Ségonne F, Fischl B, Quinn BT, Dickerson BC, Blacker D, et al. An automated labeling system for subdividing the human cerebral cortex on MRI scans into gyral based regions of interest. NeuroImage 2006 1 Jul;31(3):968–980. http://dx.doi.org/10.1016/j.neuroimage.2006.01.021.

63. Mai JK, Majtanik M. Myeloarchitectonic maps of the human cerebral cortex registered to surface and sections of a standard atlas brain. Translational neuroscience 2023 1 Jan;14(1):20220325. http://dx.doi.org/10.1515/tnsci-2022-0325.
64. Petrides M, Tomaiuolo F, Yeterian EH, Pandya DN. The prefrontal cortex: comparative architectonic organization in the human and the macaque monkey brains. Cortex; a journal devoted to the study of the nervous system and behavior 2012 1 Jan;48(1):46–57. http://dx.doi.org/10.1016/j.cortex.2011.07.002.
65. Warrington S, Thompson E, Bastiani M, Dubois J, Baxter L, Slater R, et al. Concurrent mapping of brain ontogeny and phylogeny within a common space: Standardized tractography and applications. Science advances 2022 21 Oct;8(42):eabq2022. http://dx.doi.org/10.1126/sciadv.abq2022.
66. Coizet V, Heilbronner SR, Carcenac C, Mailly P, Lehman JF, Savasta M, et al. Organization of the anterior limb of the internal capsule in the rat. The Journal of neuroscience: the official journal of the Society for Neuroscience 2017 8 Mar;37(10):2539–2554. http://dx.doi.org/10.1523/JNEUROSCI.3304-16.2017.
67. Lein ES, Hawrylycz MJ, Ao N, Ayres M, Bensinger A, Bernard A, et al. Genome-wide atlas of gene expression in the adult mouse brain. Nature 2007 11 Jan;445(7124):168–176. http://dx.doi.org/10.1038/nature05453.
68. Wang Q, Ding SL, Li Y, Royall J, Feng D, Lesnar P, et al. The Allen Mouse Brain Common Coordinate Framework: A 3D reference atlas. Cell 2020 14 May;181(4):936–953.e20. http://dx.doi.org/10.1016/j.cell.2020.04.007.
69. Hayot-Sasson V, Glatard T, Rokem A. The benefits of prefetching for large-scale cloud-based neuroimaging analysis workflows. In: 2021 IEEE Workshop on Workflows in Support of Large-Scale Science (WORKS); 2021. p. 42–49.
70. Trinkle S, Foxley S, Kasthuri N, La Rivière P. Synchrotron X-ray micro-CT as a validation dataset for diffusion MRI in whole mouse brain. Magnetic resonance in medicine 2021 1 Aug;86(2):1067–1076. http://dx.doi.org/10.1002/mrm.28776.
71. Foxley S, Sampathkumar V, De Andrade V, Trinkle S, Sorokina A, Norwood K, et al. Multi-modal imaging of a single mouse brain over five orders of magnitude of resolution. NeuroImage 2021 1 Sep;238(118250):118250. http://dx.doi.org/10.1016/j.neuroimage.2021.118250.
72. Shapson-Coe A, Januszewski M, Berger DR, Pope A, Wu Y, Blakely T, et al. A petavoxel fragment of human cerebral cortex reconstructed at nanoscale resolution. Science (New York, NY) 2024 10 May;384(6696):eadk4858. http://dx.doi.org/10.1126/science.adk4858.
73. Presseau C, Jodoin PM, Houde JC, Descoteaux M. A new compression format for fiber tracking datasets. Neuroimage 2022 Sep;109(102893):160018.
74. Rheault F, Houde JC, Descoteaux M. Visualization, interaction and tractometry: Dealing with millions of streamlines from diffusion MRI tractography. Frontiers in neuroinformatics 2017 26 Jun;11:42. http://dx.doi.org/10.3389/fninf.2017.00042.
75. Gabusi I, Battocchio M, Bosticardo S, Schiavi S, Daducci A. Blurred streamlines: A novel representation to reduce redundancy in tractography. Med Image Anal 2024 Apr;93(103101):103101.
76. Kruper J, Hagen MP, Rheault F, Crane I, Gilmore A, Narayan M, et al. Tractometry of the Human Connectome Project: resources and insights. Frontiers in neuroscience 2024 12 Jun;18:1389680. http://dx.doi.org/10.3389/fnins.2024.1389680.
77. Rheault F, Hayot-Sasson V, Smith RE, Rorden C, Tournier JD, Garyfallidis E, et al. TRX: A Community-Oriented Tractography File Format. In: 28th Annual Meeting of The Organization for Human Brain Mapping (OHBM) Glasgow, Scotland; 2022. .
78. Bizzi A, Yang JYM, Aliaga-Arias J, Dell'Acqua F, Lavrador JP, Vergani F. Chapter 31 - Neurosurgical applications of clinical tractography. In: Dell'Acqua F, Descoteaux M, Leemans A, editors. Handbook of Diffusion MR Tractography : Academic Press; 2025.p. 631–652.
79. Costabile JD, Alaswad E, D'Souza S, Thompson JA, Ormond DR. Current applications of diffusion tensor imaging and tractography in intracranial tumor resection. Frontiers in oncology 2019 29 May;9:426. http://dx.doi.org/10.3389/fonc.2019.00426.
80. Vanderweyen DC, Theaud G, Sidhu J, Rheault F, Sarubbo S, Descoteaux M, et al. The role of diffusion tractography in refining glial tumor resection. Brain structure & function 2020 May;225(4):1413–1436. http://dx.doi.org/10.1007/s00429-020-02056-z.
81. Kwon HG, Hong JH, Hong CP, Lee DH, Ahn SH, Jang SH. Dentatorubrothalamic tract in human brain: diffusion tensor tractography study. Neuroradiology 2011 Oct;53(10):787–791. http://dx.doi.org/10.1007/s00234-011-0878-7.
82. Lehman VT, Lee KH, Klassen BT, Blezek DJ, Goyal A, Shah BR, et al. MRI and tractography techniques to localize the ventral intermediate nucleus and dentatorubrothalamic tract for deep brain stimulation and MR-guided focused ultrasound: a narrative review and update. Neurosurgical focus 2020 1 Jul;49(1):E8. http://dx.doi.org/10.3171/2020.4.FOCUS20170.
83. Nowacki A, Debove I, Rossi F, Schlaeppi JA, Petermann K, Wiest R, et al. Targeting the posterior subthalamic area for essential tremor: proposal for MRI-based anatomical landmarks. Journal of neurosurgery 2019 Sep;131(3):820–827. http://dx.doi.org/10.3171/2018.4.JNS18373.
84. de Faria O Jr, Pivonkova H, Varga B, Timmler S, Evans KA, Káradóttir RT. Periods of synchronized myelin changes shape brain function and plasticity. Nat Neurosci 2021 Nov;24(11):1508–1521.
85. Knickmeyer RC, Gouttard S, Kang C, Evans D, Wilber K, Smith JK, et al. A structural MRI study of human brain development from birth to 2 years. J Neurosci 2008 Nov;28(47):12176–12182.
86. Shi F, Yap PT, Wu G, Jia H, Gilmore JH, Lin W, et al. Infant brain atlases from neonates to 1- and 2-year-olds. PLoS One 2011 Apr;6(4):e18746.
87. Sanchez CE, Richards JE, Almli CR. Neurodevelopmental MRI brain templates for children from 2 weeks to 4 years of age. Developmental psychobiology 2012;54(1):77–91.
88. Calixto C, Dorigatti Soldatelli M, Jaimes C, Pierotich L, Warfield SK, Gholipour A, et al. A detailed spatiotemporal atlas of the white matter tracts for the fetal brain. Proceedings of the National Academy of Sciences 2025 7 Jan;122(1):e2410341121. http://dx.doi.org/10.1073/pnas.2410341121.
89. Gilmore JH, Knickmeyer RC, Gao W. Imaging structural and functional brain development in early childhood. Nat Rev Neurosci 2018 Feb;19(3):123–137.
90. Grotheer M, Rosenke M, Wu H, Kular H, Querdasi FR, Natu VS, et al. White matter myelination during early infancy is linked to spatial gradients and myelin content at birth. Nature communications 2022;13(1):1–12.
91. Guerrero JM, Adluru N, Bendlin BB, Goldsmith HH, Schaefer SM, Davidson RJ, et al. Optimizing the intrinsic parallel diffusivity in NODDI: An extensive empirical evaluation. PLoS One 2019 Sep;14(9):e0217118.
92. Chang KH, Burke L, LaPiana N, Howlett B, Hunt D, Dezelar M, et al. Free water elimination tractometry for aging brains. bioRxiv 2024 Nov;p. 2024.11.10.622861.
93. Hollmann S, Frohme M, Endrullat C, Kremer A, D'Elia D,

Regierer B, et al. Ten simple rules on how to write a standard operating procedure. PLoS computational biology 2020 3 Sep;16(9):e1008095. http://dx.doi.org/10.1371/journal.pcbi.1008095.

94. Layton KJ, Kroboth S, Jia F, Littin S, Yu H, Leupold J, et al. Pulseq: A rapid and hardware-independent pulse sequence prototyping framework. Magn Reson Med 2017 Apr;77(4):1544–1552.
95. Liu Q, Ning L, Shaik IA, Liao C, Gagoski B, Bilgic B, et al. Reduced cross-scanner variability using vendor-agnostic sequences for single-shell diffusion MRI. Magn Reson Med 2024 Jul;92(1):246–256.
96. Barker M, Chue Hong NP, Katz DS, Lamprecht AL, Martinez-Ortiz C, Psomopoulos F, et al. Introducing the FAIR Principles for research software. Sci Data 2022 Oct;9(1):622.
97. Ambatipudi S, Byna S. A comparison of HDF5, zarr, and netCDF4 in performing common I/O operations. arXiv [csDC] 2022 Jul;.
98. Vohra D. Apache Parquet. In: Practical Hadoop Ecosystem Berkeley, CA: Apress; 2016.p. 325–335.
99. Lopez-Gomez J, Blomer J. RNTuple performance: Status and Outlook. arXiv [physicsdata-an] 2022 Apr;.
100. Beare R, Alexander B, Warren A, Kean M, Seal M, Wray A, et al. Karawun: a software package for assisting evaluation of advances in multimodal imaging for neurosurgical planning and intraoperative neuronavigation. Int J Comput Assist Radiol Surg 2023 Jan;18(1):171–179.

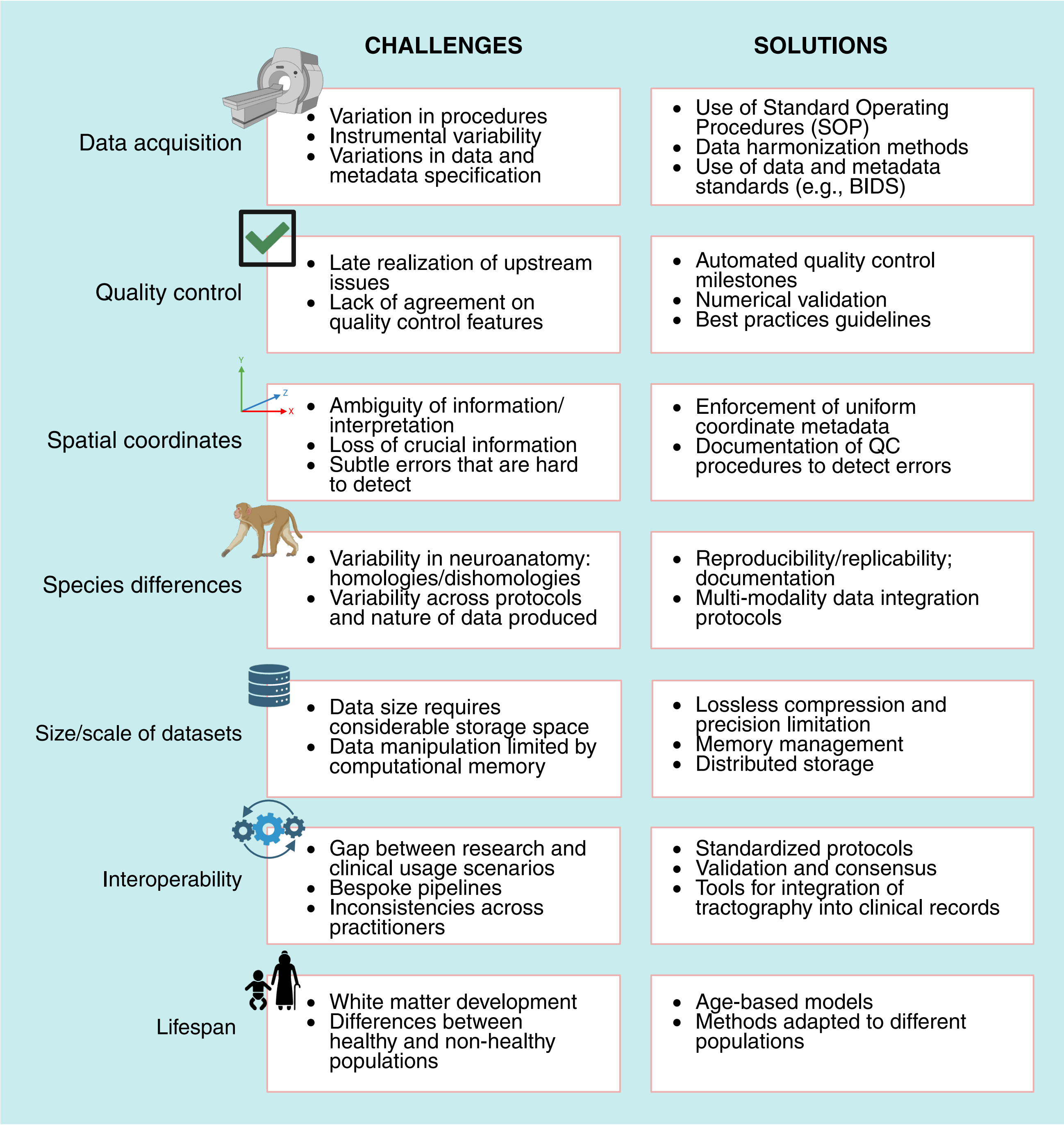

Figure 1. Summary of main challenges and suggested standardization solutions towards reliable, reproducible, and robust tractography.

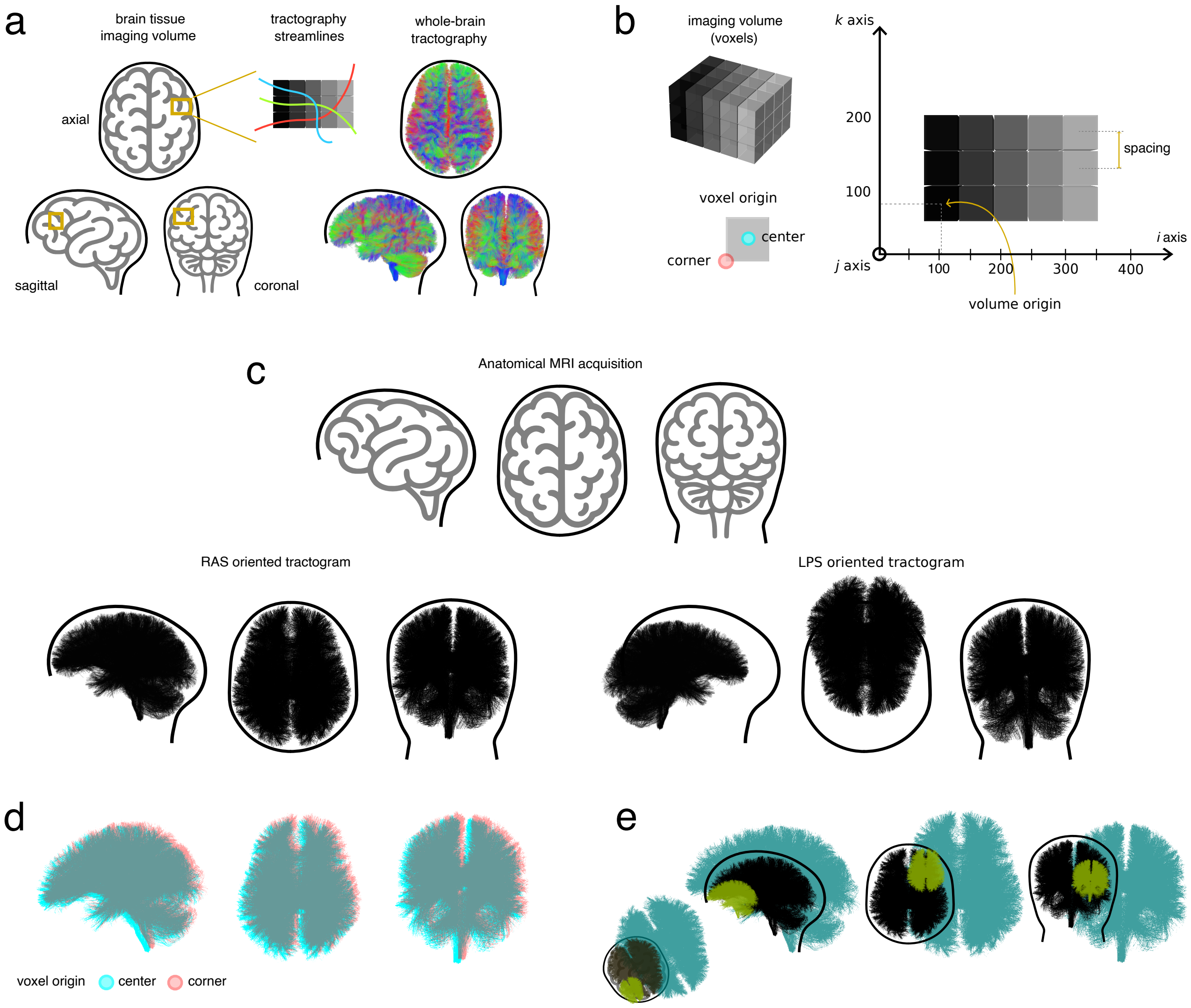

Figure 2. Tractography data representation issues. Panel a: schematic view of the process of generating tractography streamline data from an imaged brain tissue volume; Panel b: miscellaneous concepts related to tractography data; Panel c: illustration of the spatial mismatch between a tractography file natively serialized following the *LPS+* convention but the structural data (e.g., a T1-weighted MRI acquisition) being arranged following the *RAS+* convention; Panel d: effect of the voxel origin convention visualized as a half-voxel shift between the *corner* and *center* conventions on a whole-brain tractogram; Panel e: illustration of tractography data representation issues arising from mismatches between the spatial coordinate transformation matrix employed when reconstructing streamlines *vs.* the one used when serializing the tractography data, or from storing the data in voxel space *vs.* real-world space coordinates, or from inadvertently applying spatial transformations multiple times. In all cases, the tractogram in black is at the correct location, and the tractograms in green and turquoise do not lie at the correct anatomical location.

Personal Cover
Click here to access/download;Personal Cover;2025-Cover-GigaScience-IST-Standardization-Proposal.pdf

Ariel Rokem, PhD
Department of Psychology
Guthrie Hall 119 A
Seattle, WA 98105
Phone: +1 (510) 387-6264
E-mail: arokem@uw.edu
https://neuroinformatics.uw.edu

September 4th , 2025

Dear *GigaScience* Editors,

We are pleased to submit a proposal for a potential "Review" contribution to *GigaScience* titled "What needs to be standardized for reliable, reproducible, and robust tractography?"

Tractography is a cornerstone method for neuroanatomical studies, including many Open/Big Data initiatives, yet it remains plagued by ambiguity –ranging from fragmentation in spatial reference information, to the absence of unified analysis protocols. To address these challenges, the International Society for Tractography (https://tractography.io/) convened a panel of experts for a series of discussions focused on tractography standardization. This has resulted in a detailed white paper on the standards and policy related to these questions that we are now pleased to submit for your consideration.

The set of authors for this potential contribution is authoritative and very diverse, and is composed of 17 recognized leaders in the field from 21 different institutions in 6 different countries. The work is timely given the increasing reliance on tractography data for both clinical applications and large-scale neuroscience initiatives. The authors of the proposed contribution cover a broad range of expertise, including researchers that use many different methods and model systems in basic research, as well as clinicians who use tractography in their work.

We believe that this white paper could serve as the basis for a "Review" paper in *GigaScience*, as it provides a forward-looking and critical assessment of the scientific and technical challenges and opportunities that currently exist for standardization in the highly interdisciplinary field of tractography. In this work, we have also formulated a series of steps towards improvements of the state of the field in the future.

We confirm that all authors have approved the manuscript for submission, and that potential competing interests have been disclosed in the manuscript. The content of the manuscript has not been published, or submitted for publication elsewhere.

Thank you for your consideration of our proposal.

Sincerely,

Ariel Rokem, PhD
Research Associate Professor, Department of Psychology
Adjunct Associate Professor, Paul G. Allen School of Computer Science & Engineering
Senior Data Scientist, eScience Institute
University of Washington
