# Supplementary material for: What needs to be standardized for reliable, reproducible, and robust tractography?
Source: Gigascience. 2026 Mar 25;15:giag034. doi: 10.1093/gigascience/giag034 (PMC13137869; doi:10.1093/gigascience/giag034)
Supplement: giag034_GIGA-D-25-00365_Revision_1 [file giag034_giga-d-25-00365_revision_1.pdf]

GigaScience

What needs to be standardized for reliable, reproducible, and robust tractography?

--Manuscript Draft--

**Manuscript Number:**	GIGA-D-25-00365R1
**Full Title:**	What needs to be standardized for reliable, reproducible, and robust tractography?
**Article Type:**	Review

Funding Information:

National Institute of Biomedical Imaging and Bioengineering (2R01EB027585-04A1)	PhD Wei Tang
National Institute of Biomedical Imaging and Bioengineering (K01EB032898)	PhD Kurt Schilling
National Institute of Mental Health (2R01MH112847)	PhD Matthew Cieslak
National Institute of Mental Health (2R01MH120482)	PhD Matthew Cieslak
National Institute of Mental Health (2R01MH113550)	PhD Matthew Cieslak
HORIZON EUROPE European Research Council (101163214)	PhD Alberto De Luca
Galen and Hilary Weston foundation	PhD Alberto De Luca
Stichting Hanarth Fonds	PhD Alberto De Luca
Division of Graduate Education (DGE-2140004)	MSc John Kruper
Natural Sciences and Engineering Research Council of Canada	PhD Francois Rheault
HORIZON EUROPE European Research Council (101000969)	Prof Stamatios N. Sotiropoulos
Wellcome Trust (226486/Z/22/Z)	PhD Franco Pestilli
National Institute of Neurological Disorders and Stroke (UM1NS132207)	PhD Franco Pestilli<br>PhD Sarah Heilbronner
National Institute of Neurological Disorders and Stroke (U24NS140384)	PhD Franco Pestilli
Kids' Cancer Project	MD, PhD Joseph Yuan-Mou Yang
Université de Sherbrooke (Research Chair in Neuroinformatics)	Prof Maxime Descoteaux
National Institute of Mental Health (MH121868)	PhD Ariel Rokem
National Institute of Mental Health (MH121867)	PhD Ariel Rokem
National Institute of Mental Health (R25MH112480)	PhD Ariel Rokem
National Institute on Aging (R01AG060942)	PhD Ariel Rokem
National Institute on Aging (U19AG066567)	PhD Ariel Rokem
National Institute of Biomedical Imaging and Bioengineering (R01EB027585)	PhD Ariel Rokem
National Science Foundation (1934292)	PhD Ariel Rokem
National Science Foundation (2334483)	PhD Ariel Rokem
Chan Zuckerberg Initiative	PhD Ariel Rokem

Powered by Editorial Manager® and ProduXion Manager® from Aries Systems Corporation

	Royal Children's Hospital Foundation (RCHF 2022-1402, RCHF 2025-1621)	MD, PhD Joseph Yuan-Mou Yang
**Abstract:**	Tractography is a key component of efforts to map brain connectivity. As a rapidly-evolving field of neuroscience, current tractography methods are diverse, often varying across research laboratories and different software pipelines. Therefore, it suffers from a lack of standardization leading to inconsistencies in results, which can limit reproducibility, and affect the robustness needed for research and clinical applications of these methods. Variability in data acquisition procedures, inconsistencies in spatial referencing schemes and implementations, and anatomical heterogeneity —at the individual level, across the lifespan, and across species— hinders comparative analyses. Additionally, the lack of consensus on best practices complicates the development of robust automated quality control pipelines and limits the clinical translation of tractography-based procedures. Establishing standardized protocols for acquisition, preprocessing, and tractography reconstruction are critical towards enabling reliable tract-specific analyses, facilitating cross-study harmonization, and supporting replicable large-scale population studies. The present article provides an overview of the current challenges in tractography standardization and identifies the key aspects that require standardization for reliable, reproducible, and robust tractography.	
**Corresponding Author:**	Jon Haitz Legarreta<br>Brigham and Women's Hospital<br>Somerville, Massachusetts UNITED STATES	
**Corresponding Author Secondary Information:**		
**Corresponding Author's Institution:**	Brigham and Women's Hospital	
**Corresponding Author's Secondary Institution:**		
**First Author:**	Jon Haitz Legarreta	
**First Author Secondary Information:**		
**Order of Authors:**	Jon Haitz Legarreta	
	Simona Schiavi	
	Wei Tang	
	Garrett Banks	
	Matthew Cieslak	
	Kurt Schilling	
	Alberto De Luca	
	Jacques-Donald Tournier	
	John Kruper	
	Francois Rheault	
	Stamatios N. Sotiropoulos	
	Franco Pestilli	
	Jelle Veraart	
	Joseph Yuan-Mou Yang	
	Maxime Descoteaux	
	Sarah Heilbronner	
	Ariel Rokem	
**Order of Authors Secondary Information:**		
**Response to Reviewers:**	Letter to Editor	

Powered by Editorial Manager® and ProduXion Manager® from Aries Systems Corporation

----

Prof. Hongfang Zhang
Editor
GigaScience

Department of Radiology
Brigham and Women's Hospital
Harvard Medical School
02145 Somerville MA USA
jhlegarreta@bwh.harvard.edu

February 3, 2026
Revision to manuscript GIGA-D-25-00365

The authors would like to thank the editor and reviewers for their constructive comments and suggestions that have helped improve the quality of this manuscript. The manuscript has undergone a thorough revision according to the editor and reviewers' comments. Please see below our point-by-point responses. For the reviewers' convenience, we have highlighted changes in the revised manuscript in strikethrough
red/underlined blue.

The main changes to the manuscript are as follows:
- We have extensively revised the manuscript, providing further clarifications to the points raised by the reviewers.
- We discuss additional points raised by the reviewers that contribute to a more comprehensive view of the need for tractography standardization.
- We detail actionable measures needed to support standardization throughout the manuscript, including the effort required for each of the recommendations.
- We have corrected the typos and added suggested references.

We look forward to your response.

Truly yours,
Jon Haitz Legarreta, PhD
On behalf of the co-authors

Response to Reviewers

Reviewer 1

This aim of article is threefold: introduce some of the problems related to lack of standardization for tractography, offers potential solutions, and provide recommendations for the research community relating to the solutions. The three contexts identified in the abstract are: (1) enabling reliable tract-specific analyses, (2) facilitating cross-study harmonization, and (3) supporting replicable large-scale population studies.

Tractography is a widely used MRI analysis technique, applied to both research and clinical decision-making. Owing to its complexity, results are hard to quantify and interpret, and this lack of reliability is an important limitation.

Reviewer Comment 1.1

The aim of the article is clear. It seems to align with the journal's criteria for reviews: "critical assessments of emerging technologies [. . .] white papers on standards and policy." However, the focus is not specifically aimed at "research areas relating to the utilisation of large scale research data".

Powered by Editorial Manager® and ProduXion Manager® from Aries Systems Corporation

Reply:

Tractography is one of the many research fields that have seen a transition from smaller bespoke datasets to very large consortium led data acquisition efforts. At the same time, data acquisition methods are generating data at ever-higher resolution. This means that TB- and even PB-scale datasets are more prevalent. These large-scale datasets are an important motivation for the present review, as with these datasets standardization becomes not only more convenient, but mission critical.

To emphasize this issue, we added text in the "Background" section (L27-38):

These challenges have become increasingly pressing as the field has transitioned in recent years towards larger and larger datasets. This increase in data volume and complexity arises from large-scale data acquisition projects, with thousands of subjects, on the one hand. Examples of these include the Adolescent Brain Cognitive Development (ABCD) [21], Healthy Brain and Child Development (HBCD) [22], and Healthy Brain Network [23, 24] studies, as well as the UK Biobank [25], and many others. On the other, it arises from the increase in the resolution as well as coverage of measurements.
These larger data have led to the application of data-driven discovery methods, where consistent and comprehensive standards are necessary.

Reviewer Comment 1.2

There is a disconnect between the title of the review and the contents. Specifically, while the terms "reliable", "replicable", and "robust" are used in the title, they are not defined in the text and are not used prominently to organize the discussion. It would have been helpful for the authors to translate these concepts into operational terms to make the usefulness of their recommendations clearer.

Reply:

To clarify the theoretical underpinnings of our work, and to make these terms more concrete, we have provided a definition of these terms (L65-88) and in each of our sections, we describe how the standardization challenges and solutions that are proposed relate to these terms (or where they are less relevant to these terms):

Reliability, reproducibility, and robustness in tractography encompass multiple methodological dimensions. They manifest across the full data life-cycle of tractography, including acquisition, processing, and analysis. They also manifest in inferences applied across different species, different types of measurements, different ages, and across basic research and clinical application. In this context, we define reliability as the consistency of the reconstruction under fixed conditions (e.g., repeated measurements on the same participant, repeated computations of the same reconstruction method, and repeated use of the same tractography algorithm). Reproducibility (sometimes also referred to as "replicability ", see [29]) is the ability to obtain equivalent results across sites, operators, and implementations, while preserving the expected anatomical observations (e.g., consistency across software packages, versions, and hardware platforms, consistent results obtained from the same shared data, etc.). Finally, robustness characterizes the stability of tractography algorithms in the face of varying conditions (e.g., noise perturbations, multi-site or multi-vendor datasets, etc.). As we will demonstrate in addressing a range of practical and conceptual challenges, standardization is essential to achieve these goals. Therefore, as we survey a range of challenges and proposed solutions, we will highlight key factors influencing these aspects and propose ways to increase them.

Reviewer Comment 1.3

I note that in the metadata accompanying the manuscript, the authors reply "yes" to the question "Have you included all the information requested [regarding design and statistics] in your manuscript?" However, given that the format is effectively that of a narrative review the more correct answer would be "not applicable".

Powered by Editorial Manager® and ProduXion Manager® from Aries Systems Corporation

	Reply:<br><br>We agree with the reviewer’s comment, and have changed our answer to “Not applicable”.<br><br>Reviewer Comment 1.4<br>--------------------<br><br>I would say many of the problems discussed are already well-known (for example, spatial coordinates). Implementation of the solutions can be difficult. It might have been useful to classify the degree of progress regarding each of the problems mentioned. Some seem easy to achieve (harmonization of data formats) while others are only a distant dream. For example, people were discussing vendor-agnostic sequences in the 1990s, but the documented progress has been limited and the limitations are not only technical but also institutional (commercial).<br><br>Reply:<br><br>We have edited each section so that they end with a paragraph that clearly describes the current state of the challenges described.<br><br>Data acquisition (L143-183):<br><br>Despite the progress that has been achieved, further work is needed to provide an adequate basis for tractography. Using BIDS for neuroimaging data sharing offers a high-level of consistency; however, its adoption across pipelines is not yet all-pervading, and its implementation is not uniform. Furthermore, although the diffusion extension proposal has been submitted for inclusion in the standard, it only covers scalar derivatives and tensors. The tractography extension has not been submitted yet for inclusion. Similarly, vendor-agnostic, standardized sequences are yet to be implemented across the variety of scanning hardware and protocols, and be validated extensively. Limited support and interest for regulatory clearance entail additional hindrance to their wide adoption [45]. Finally, harmonization is still a topic under active research, or present limitations (e.g., fixed, well populated site numbers, homogeneous populations) that have yet to be overcome. Thus, significant methodological gaps remain in diffusion MRI data acquisition that limit the development of automated and robust tractography downstream.<br><br>Standard operating procedures (SOPs) are necessary to remove variability at data collection and provide some level of reliability in tractography reconstruction from a given study. SOPs have been employed to describe the imaging and processing protocols for large-scale studies (e.g., the Human Connectome Project (HCP), the ABCD study, etc.); more recent studies (e.g., the HBCD study, or the Human Connectome PHantom Study (HCPh)) have adopted contemporary web technologies to modernize data presentation. Smaller-scale, single-site studies would likewise benefit from this practice, contributing to improved transparency in reporting and reproducibility. Similarly, standardized data and metadata records and organization (e.g., through BIDS) are required to ensure reproducibility. This includes developing the extensions to derivatives (e.g., fiber orientation distributions) that are employed to generate tractography results. Finally, harmonization solutions (within and across scanners and sites, across acquisition upgrade packages, etc.) are required to resolve data source variability. Combined with the adoption of unambiguous, self-reporting data formats (see Spatial coordinates), these measures would mitigate practical barriers and contribute to reducing overhead in multi-center studies with disparate settings.<br><br>Quality control (L230-250):<br><br>Taken together, progress has been limited in tractography QC standardization practice and adoption levels. Although there are many advanced tractography visualization tools and paradigms [51], and de- spite some pipelines offering automated reporting, their use for QC purposes has not been thoroughly studied. Similarly, validating numerical tractography QC methods is still impacted by the inability to have gold

standards on real brain data, and the lack of anatomical measures defining the success of a tractography method. As a result, best practices for excluding low-quality results based on either visual or numerical reports remain insufficiently established. Reliable tractography would benefit from clearly defined, shared criteria determining the quality of results, including standardized reporting. Additionally, universally shared phantom, and synthetic data, as well as anonymized in vivo and ex vivo data from both healthy and diseased participants are required to guarantee a common ground for quality control of a given tractography pipeline. This includes sharing data in software-agnostic repositories (e.g., OpenNeuro or Zenodo). Note that this extends beyond datasets used across international challenges that quantify tractography results only within a particular dataset (see, for example, [63,17, 64, 16]).

Spatial coordinates (L310-323):

Overall, despite a well-established understanding of the coordinate frameworks involved in tractography reconstruction, current pipelines continue to rely on implicit or insufficiently documented conventions. Clear and unambiguous guidance for debugging, validating, and converting data across coordinate spaces and formats remains lacking. Consequently, meaningful progress in this area has remained limited. A tractography data standard that explicitly and unambiguously documents its spatial coordinate framework (e.g., according to the BIDS convention), and version-controlled, general resources (i.e., not tied to a particular processing package) are required. This includes testing data stored in software-agnostic repositories, and guidelines towards conformance, error checking and conversion of retrospective data.

Translation across species and methods (L421-437):

(...). As a consequence, the progress to bridge the gap in tractography data representation and software across species and imaging modalities has been limited. Addressing these challenges requires a comprehensive approach that includes standardized multi-modality and multi-resolution data acquisition, advancements in hardware for efficient in vivo scanning, essential validation studies, and methodological innovations, such as automated tools for establishing a common framework of correspondence.

While the challenges to validity and generalization are evident, these issues also raise challenges to robustness. This is because the differences in measurement tools and in analysis methods need to be thoroughly adapted and validated when they are used in species other than the ones on which they have been developed. Examples in which tractography tools that were developed for use in human dMRI data were adapted to be used in non-human data [82] suggest that there is moderate progress on this challenge.

The scale of data (L482-494):

Software and data-format enhancements have so far enabled only limited progress, due to heterogeneous precision adjustment across pipelines and partial adoption of the TRX file format. Data storage and visualization remain major bottlenecks, and significant advances have relied primarily on improvements in computational power, with efficient mapping of brain connectivity at scale being possible on supercomputing hardware [93] or using Graphics Processing Units (GPUs) [94, 95]. Distributed data storage (e.g., with zarr [96]) could offer an attractive possibility for work with very large datasets. Effective visualization of memory-intensive tractography data will require implementations in low-level programming languages, such as C++ or Rust, and/or compatibility with GPU hardware acceleration.

Integration of modern machine learning methods (L571-584):

More broadly, the development and application of machine learning methods are facing a fragmented landscape, where different software is used in different use-cases. Competition among large corporate entities (e.g., technology companies) is also driving this fragmentation, as different entities try to position their tools as leading in the marketplace. In cases where models are shared not only as software, but also in the form of parameter values (or “weights”), the standardization of the format of model

Powered by Editorial Manager® and ProduXion Manager® from Aries Systems Corporation

parameters, and of the metadata associated with these parameters becomes important. This is where industry standards such as the Open Neural Network Exchange (ONNX) standard [101] could play an important role. For these use-cases, there is much to benefit from the adoption of already-existing standards that apply broadly, rather than reinventing these standards for narrow applications.

Translation of tractography methods to clinical applications (L639-655):

Thus, limited progress has been made to create reference procedures to bridge basic research in tractography and clinical work. Consistent acquisition and processing protocols are required to produce tractography results that can be translated across clinical settings. In addition, methodological innovations are needed for time-constrained clinical acquisitions —such as optimized pulse sequences, undersampling strategies, and super-resolution techniques— that enable robust tractography results, alongside tractography methods that remain reliable when employed on lower-quality data. Additional measures to support clinical adoption include systematic reporting of reconstruction uncertainty across varying data quality conditions (e.g., noise levels) and reconstruction settings (e.g., seeding strategies). Finally, establishing reliable proxies for clinical outcomes is essential to validate the reliability of fiber reconstruction techniques.

Standardization of methods throughout the lifespan (L687-696):

Progress in offering authoritative, centralized and standardized resources for brain connectivity mapping across the lifespan has been limited; as an example, the extension of the BIDS standard to describe atlases does not, at the moment, cover tractography templates. Standardizing tractography across the lifespan will require creating such templates, generating normative derivatives linking structural connectivity to tissue microstructure (tractometry), providing uniform method implementations, and guidelines for calibrating processing parameters.

Additionally, we have provided some qualitative assessment related to the degree of progress of each aspect, and have a adopted a three-tier scale from limited to substantial, moderate being between them (see response to Comment 1.11).

Reviewer Comment 1.5

For a review article, the manuscript is rather brief. It is more of a summary of previous literature than a comprehensive synthesis. Likewise, the solutions offered are extremely concise. They could not be used, for example, to guide the design of a workflow aimed at acquiring and analysing multicentre DWI for tractography. It might have been useful to take one or two concrete scenarios and make specific recommendations related to these. To take an example more-or-less at random, the 2019 review of Jacquesson et al. on cranial nerve tractography synthesized previous studies and offered an opinion on emergent best practice. Although the authors may be aiming at a higher-level synthesis in the current work, their stated aim encompasses three distinct scenarios (see Scope and Rationale) and it is not clear how the recommendations might differ for each of them.

Reply:

In addressing the comments made by the reviewers, the manuscript has indeed grown in volume. Indeed, the article provides some summary of the previous literature, in addition to recommendations that appear in the section titled “Summary and recommendations”. We agree that we have not gone into the level of detail provided in the Jacquesson et al. article, regarding specific protocols. The goal of the present article is a bit broader, and we also hope to reach and interest the much broader audience of GigaScience (which includes non-neuroscientists). As you can see in the article’s byline, the authors represent a very broad range of institutions. They also come from a wide range of disciplinary backgrounds and expertise, including computer scientists, computational neuroscientists, neuroanatomists, clinicians, and psychologists. This means that writing this article entailed seeking a broad consensus

that cuts across all of these groups and disciplines. Nevertheless, despite this broad group of authors, we have managed to find common ground on many issues, and we have taken a strong stance on some practices that we consider to be important for progress on the issues that we identify.

Reviewer Comment 1.6

There is no doubt that the problems identified are real ones and this is supported by evidence in the literature. It is not made clear which are the most important in practice, either in terms of the widespread adoption of suboptimal protocols or in terms of the degree of loss in reliability or robustness. Not all of the sections appear to be equally relevant; as an example, how does “translation across species” relate to reliability, reproducibility or robustness? It seems that the main link is the framework developed by the Allen Mouse Brain Common Coordinate Framework, but this works only with mouse data. The problem of comparing concepts across species is more related to validity than reliability, and as such does not fit neatly into the topic of the manuscript.

Reply:

With the new section that defines these terms, we hope that it is now more clear how the cross-species comparisons relate to these terms. To assist the reader in making the connection, we have added the following text (L430-437):

While the challenges to validity and generalization are evident, these issues also raise challenges to robustness. This is because the differences in measurement tools and in analysis methods need to be
thoroughly adapted and validated when they are used in species other than the ones on which they have been developed. Examples in which tractography tools that were developed for use in human dMRI data were adapted to be used in non-human data [82] suggest that there is moderate progress on this challenge.

We have also added standard coordinate frames that support work in non-human primate species, which should lend a broader scope to this section. The new text in the manuscript is as follows (L357-375):

This poses foremost a challenge to the validity of inferences between species. Integration between human and non-human primate data remains conceptually challenging, because non-human anatomical knowledge relies heavily on atlases based on cytoarchitectonics (e.g., [71]). These do not directly align with the common atlases used in human MRI studies (e.g., [72]). While cytoarchitectonics-based human brain atlases have been developed [73], some even with a particular stress on cross-species homology [74], these have not been integrated with widely used neuroimaging tools. At the same time, common coordinate frameworks for non-human primates are beginning to emerge (see, for example, [75]), in some cases with the explicit goal of establishing a direct relationship with the developing human connectome [76]. Tractography fingerprinting [77], i.e., using white matter bundles as latent landmarks to assess similarities and divergences across the two species, is geometry-agnostic and can provide additional solutions to the challenge of integrating cross-species coordinate frameworks.

Reviewer Comment 1.7

As stated, the solutions are plausible but some are much more readily available than others. It would have been helpful if the authors had compared the feasibility of the different solutions in terms of methods and technology available today. Sometimes, it seems as though suggestions are contradictory or at least are not uniformly applicable. On the one hand, standardization of file formats is implicit in recommendations i and iv, while on the other “the community needs to advance new file formats” (recommendation viii) that address the limitations of currently-existing formats. To follow this recommendation literally, researchers would have to delay studies until widely agreed-upon future-proofed formats were available. As another example,

building bridges between research and clinical tractography (recommendation viii) sounds like a good idea but the requirements of research and clinical scanning are different. For clinical use, MR scanners must be approved by the FDA, EU, or similar authority. Would vendor-agnostic sequences (recommendation ii) be authorised for such use?

Reply:

We have added comments on the perceived feasibility of the suggested measures (see response to Comment 1.11). In particular, we point out that only a minor effort would be needed to use future-proof file formats, and we identify that already-existing TRX file format as a good option for scientists to already use now.

A goal of the current paper is to identify the relatively wide agreement that already exists on this topic, and to encourage scientists not to wait any further.

We have remained agnostic to the data or file format chosen when recommending SOPs. Similarly, we suggest the QC protocols be developed around the idea of what aspects of tractography need to be checked rather than what file format these protocols will read.

We do not see why vendor-agnostic sequences should not get regulatory clearance if their benefits can be demonstrated and the relevant validation experiments are presented.

Reviewer Comment 1.8

The work is a useful summary of previous literature but does not, in my view offer a new understanding, or real practical guidance, to practitioners in the field. A statement like “To help bridge basic research and clinical work, acquisition and processing protocols need to be made more consistent across settings” is doubtless true, but the potential reader of the work might have hoped for more concrete guidance as to where to focus their efforts, or simply examples of cases where this has been done successfully.

Reply:

We have added explicit examples of concrete solutions required to advance tractography toward standardization, reproducibility, and robustness. We believe these additions address the reviewer’s concern regarding the lack of practical guidance in the original manuscript.

Reviewer Comment 1.9

At a basic level the work is logically organised, but it is not clear that all the sections address the problem that is nominally the object of the work. Apart from examples mentioned above, the section on “the scale of data” is highly relevant to the focus of the journal but less well-aligned with the reliability of tractography - feasibility possibly, but the authors do not make a case that acquisition or processing pipelines are not currently robust due to a data management problem.

Reply:

As mentioned above, we have added pointers throughout to the definitions of reliability, reproducibility and robustness. This includes an explanation of the robustness challenges that occur when procedures that work on data at a small scale fail to translate to large-scale data. This argument is now made on lines L455-463:

Manipulating such data volumes pose a challenge in terms of the required hardware and computational power. As imaging acquisition hardware evolves, and data storage capacity and physical memory limits increase, current analysis tools may buckle under the strain. This means that some approaches, which work well with smaller datasets,

can become infeasible when translated into larger data, threatening robustness. A few technical and standardization developments may ease this strain. First, (...)

Reviewer Comment 1.10

Figure 2 may be useful for those unfamiliar with MRI acquisition but does not add much value for experienced practitioners.

Reply:

We thank the reviewer for the comment. We believe that the mentioned figure illustrates in an explicit and graphical way data formatting problems that are relevant to modern tractography research. Crucially, these issues (i) are present and pervasive across current tractography practice, researchers investing significant amounts of time dealing with them; and (ii) are only apparent at late stages (e.g., when computing tractography derivatives). The variety of software packages and versions, the atlases that are freely available in a multiplicity of formats, make these issues surface continuously, especially when (i) sharing data across research groups, or (ii) employing mixed software and pipelines that naturally work with different formats. Thus, we believe that the figure is appropriate to highlight these. We advocate for early (ideally, automated) checks to avoid these problems, avoiding to waste computational resources through early identification, and thus, allowing faster scientific throughput and discovery.

Reviewer Comment 1.11

[Are any take-home messages clear and useful for researchers and practitioners?] No, this work aims more at raising awareness about likely problems than in providing solutions for specific scenarios.

Reply:

We have incorporated this feedback and now emphasize the feasibility of each of the recommendations in the last section of the article (L706-810):

1. Implement standard operating procedures [120] during data acquisition, i.e., written sets of instructions that specify the processes that take place during the measurement. Feasibility: a moderate effort is required to implement centralized SOPs towards tractography standardization. That said, some examples of SOPs for tractography data acquisition [121], quality assurance [48] and analysis [122] already exist (albeit, with a focus on dMRI), and could easily be adopted by the community.

2. Adopt vendor-agnostic, open-source pulse sequence design and reconstruction frameworks (e.g., Pulseq [123, 124]) to facilitate more similar results across the instruments of different vendors. Feasibility: a substantial effort is required to make vendor-agnostic pulse sequences extensive within the data acquisition hardware, as it involves vendor support and validation towards regulatory clearance.

3. Advance BIDS-compliant standards for tractography inputs and outputs. Akin to a wide range of neuroimaging use-cases, including human electrophysiology and microscopy, where BIDS has demonstrated its utility, a tractography-specific extension is needed towards reliable, reproducible, and robust tractography data representation and sharing. Note that the particular adoption of the BIDS specification for tractography should not prevent software from processing isolated tractography files. Feasibility: As of writing of this article, the BIDS tractography extension is being drafted, and a small effort is needed to complete it.

4. Quality control. Standardized quality control procedures need to be fostered including automated procedures at every stage of processing. Further research on the impact of different decisions in QC is needed, to ultimately develop guidelines for best practices. Feasibility: due to the challenges inherent to tractography validation, a moderate effort will be required to define consensus measures and procedures towards implementing standardized QC protocols and pipelines.

Powered by Editorial Manager® and ProduXion Manager® from Aries Systems Corporation

5. Make analysis tools that are flexible to be used across different data acquisition methods, different species and different settings. FAIR-Software principles can be applied, to make sure that methods are transparent, rigorously designed and available to the community [125]. Feasibility: a substantial effort in validation is required to generalize tools across methods, species and settings. This may include generating publicly shared, reproducible records of the datasets and procedures employed.

6. Scalability. Software and platforms for sharing and computing on brain connectivity data need to be built with the large datasets of the future in mind, and with the ability to scale to much larger data than is currently available, in anticipation of the inevitable deluge of data that is expected to occur. Infrastructure for distributed and cloud computing includes evolving standards for representation of large array-based [126], tabular [127], and even trajectory data [128] in these kinds of systems, and formats for representation of tractography data could be adapted to capitalize on these developments. Feasibility: a moderate effort can be expected to design frameworks capable of handling large volumes of data. This effort is related to the development of data representation standards inherent to tractography, and the reliance on the development of general purpose, advanced scientific software.

7. Create and share annotated datasets for machine learning training. For improved interoperability and reproducibility, use industry standards for machine learning methods, such as ONNX and widely used sharing platforms for ML/AI models and tools, such as Hugging Face. Feasibility: These both require minor efforts on the side of scientists and researchers who develop these methods.

8. Future-proof standardized file formats. The community needs to advance new file formats that address the needs for consistent explicit tractography data spatial representation, while also supporting the needs for new large-scale datasets. This includes standardized, traceable, openly available, and well-documented specifications, including community-sustained conversion information and tools. We identify the nascent TRX file-format as a format that has the potential to address many of the issues raised here, specifically because it was developed taking these aspects into consideration. Feasibility: a minor effort remains to be done in terms of documentation of the TRX file format. A few initial studies already provide evidence for the suitability of TRX for reliable, and robust tractography data representation [90, 129]. A moderate effort will be required in order to incorporate the format as the output of existing software pipelines, which would make it more widely used.

9. Prioritize the articulation of clearly specified validation frameworks with common benchmarks and metrics, alongside the development of centralized, consensus evaluation tools. Importantly, these will enable unambiguous assessment of relevant aspects like heterogeneous scanners, acquisition protocols, and imaging sources, critical to assess reliability and robustness of tractography. Feasibility: a substantial effort will be required to specify the resources —including data, metrics, and technological requirements— and to operationalize and sustain the resulting infrastructure.

10. Build the bridges between research and clinical tractography: Delivery of advanced methods into clinical practice can be facilitated by standardizing workflows, and by increasing the interoperability between different parts of the clinical informatics infrastructure. For example, via integration of visualization into surgery image-guided systems, into Picture Archiving and Communication Systems (PACS) used in clinical, and into electronic medical records [130]. This will also set the scene to improve the bench-to-bedside pipeline of new computational methods. Feasibility: while research and clinical requirements remain different, the usefulness of increasingly more anatomically reliable tractography methods needs to be translated into clinical practice. Substantial efforts in terms of validation and collaboration with vendors can be foreseen.

Reviewer Comment 1.12
----

p. 4. “Additional compressions is provided”.

Powered by Editorial Manager® and ProduXion Manager® from Aries Systems Corporation

Reply:

Thank you for having spot the grammar error. We have fixed it in the manuscript.

Reviewer Comment 1.13
----

p. 5. “this approach is challenged by low inter-rater variability”. Presumably the authors mean high inter-rater variability. Low variability would be a good thing.

Reply:

Correct. We have fixed the oversight in the revised manuscript.

Reviewer 2

Tractography is an important technique for mapping brain connectivity in vivo, with many clinical and scientific applications and well-documented benefits. At the same time, many prior works have reported remaining challenges that hamper clinical adoption and might question the reproducibility and validity of some scientific conclusions that have been based on tractography. These challenges include a high degree of variation in tractography results which are, among other factors, due to the use of different acquisition protocols, processing pipelines, the dependency on subjective parameter settings, and differences in white matter tract definitions, which are difficult to reconcile due to a lack of a definite gold standard in most cases; high false positive rates of many widely used approaches; a resulting need for manual intervention, fine-tuning, and filtering; and issues of trustworthiness and generalization when attempting to resolve some of those challenges via machine learning.

Therefore, bringing together the perspectives of a broad range of experts in this field to try and resolve challenges with respect to the reliability, reproducibility, and robustness of tractography through standardization, which is the goal of the standardization unit of the recently founded International Society for Tractography, is a meaningful and valuable endeavor. Given the scale of these long-standing challenges, it is clear that they cannot be resolved all at once. Therefore, the current manuscript represents an initial step of this process, which is to define a specific list of points that require standardization, and to provide a review of what has so far been achieved with respect to each of them, and what remains to be done.

Reply:

Thank you for identifying the contribution of the article. We completely agree that this is just the first step in a long road. In order to make this point even more apparent, and in response to suggestions made by Reviewer 1, we have added more explicit language about the feasibility of the next steps in each of the areas in which progress needs to be made.

At this point, I see the following opportunities for further strengthening this review:

Reviewer Comment 2.1

In my opinion, the current weight on issues related to spatial coordinates is disproportionally large, considering the relatively large amount of text devoted to this topic and, even more importantly, the fact that this is the only challenge that is illustrated with a separate and quite large figure. The problems illustrated here are certainly important in practice, but they are also well-known and not specific to tractography. I would find this kind of material more appropriate for an introductory tutorial than for a position paper that aims to shape the future of the field. I see the fact that this section contains only a single citation as further evidence that this topic is somewhat out of scope for a review.

Powered by Editorial Manager® and ProduXion Manager® from Aries Systems Corporation

Reply:

We agree with the reviewer that this is a technical issue where there is not necessarily scientific articles to reference. Nevertheless, there are two reasons that we would like to retain the emphasis on this point, as well as the graphical representation of this issue. The first is that, given the broad scientific purview of GigaScience, the intended target audience for this article may include researchers from other fields, and we feel that it is important to explain and demonstrate how this simple-seeming issue is a source of significant error and significant technical difficulty. For these readers, it would be important to present a more extensive treatment of this issue, as we have done here. The second reason is specifically because there is not currently an article that we can refer to, which explains this issue with clarity. Therefore, we find that the lack of references in this section represents precisely the fact that the field needs a standard reference for these issues. This point is particularly important given how wide-spread these issues are (based on the experience of the authors of this paper). We believe our review will be a valuable resource in this respect.

Reviewer Comment 2.2

At the same time, I believe that issues related to the use of machine learning in tractography are currently underrepresented. Deep learning is briefly mentioned as a potential strategy that might support quality control, but it could in fact be used at almost all stages of the pipeline (image reconstruction, harmonization, tracking as such) and has the potential to address some of the fundamental challenges that the field has long been struggling with, in particular, ambiguous correspondences between the diffusion MRI signal and tract geometry (as discussed in [17]). However, learning based approaches come with their own set of challenges, such as generalization across acquisition devices / protocols and patient populations, reliable uncertainty quantification, or explainability, that would in my opinion merit a separate discussion.

Reply:

We acknowledge that tractography methods based on artificial intelligence techniques pose challenges across all stages of a tractography pipeline, including its methodological foundations (i.e., generating local orientation fields to propagate streamlines). We have added a section named “Integration of modern machine learning methods” (L496-584) (which we reproduce below) to explicitly highlight the challenges that modern artificial intelligence-based method represent towards tractography standardization:

The increasing adoption of machine learning and artificial intelligence (ML/AI) methods in dMRI and tractography has raised new challenges and exposed limitations in current standardization practices. Modern ML/AI methods require large, diverse, and well-curated datasets to achieve robust generalization. These requirements compound many of the challenges described. For example, in the absence of reliable and automated QC tools, labor-intensive quality control procedures are required. Spatial coordinate inconsistencies within large heterogeneous datasets may cause deep learning models to fail to converge. Similarly, poor data organization and metadata standardization —due to both poor compliance with BIDS and the preliminary status of the related extensions— hinder reproducibility and compromise downstream analyses, complicating cross-study comparisons. Inadequate handling of data splits can lead to train–test leakage [97]. Such leakage risks inflating reported performance and obscure the true generalizability of ML/AI-based tractography methods. Though there are some annotated datasets that can be used as the basis for training ML/AI , the scarcity of large, well- annotated, and openly shared tractography datasets —particularly multi-site and longitudinal datasets that include non-healthy individuals for clinical relevance — constrains the development of robust and reliable tractography.

Furthermore, there is need for standardization within the ML/AI methods themselves. ML/AI-based tractography techniques exhibit substantial heterogeneity in model architecture, input representation, and learning objectives, often making direct comparisons difficult (see [97, 98, 99] for relevant review works). Existing approaches

range from voxel-wise classifiers and streamline propagation networks to graph-based and geometric learning formulations. As such, the input to the model may be the raw diffusion signal, the local orientation information represented as spherical harmonics coefficients or fiber peaks, etc. Additionally, the contextual information to regularize the optimization process is also heterogeneous across methods, and includes tissue maps, neighboring voxel information, etc. Other approaches avoid reliance on the modality that sensitizes axonal pathway architecture (e.g., dMRI or other), and estimate tractography without explicit orientation data. These factors result in differing dataset requirements for training various models, often leading to substantial increases in computational requirements, both in terms of storage and processing resources. The high computational cost —including the need for specialized hardware, such as GPUs— makes regular testing and evaluation of methods expensive and resource-intensive.

This heterogeneity is compounded by the absence of universally accepted anatomical ground truth for white-matter pathways, a limitation that challenges the current tractography landscape. The evaluation of AI-based tractography algorithms frequently relies on indirect metrics, simulated phantoms, or expert-defined references, each with inherent limitations. In this context, the absence of standardized reporting conventions is particularly problematic for the evaluation of AI models. Although volume- and streamline-oriented measures are well-established since landmark studies [17, 100], evaluation tools remain poorly maintained, with most pipelines relying on bespoke implementations. Additionally, the assessment of application-specific derivatives —such as tract-specific metrics, connectivity measures, or quantitative microstructural parameters— is often inconsistent or omitted entirely. Insufficient reporting of scanner or session biases can also mask site- or protocol-specific effects that may dominate learned representations, resulting in performance gains that are often difficult to attribute to genuine methodological advances.

The result is a fragmented landscape in which performance claims are highly dependent on specific datasets, tasks, and evaluation choices, increasing the difficulty of reliable evaluation. Together, this underscores the necessity of standardization for AI-based tractography, encompassing access to well-annotated and fully described datasets, common validation frameworks with standardized benchmarks, metrics and reporting procedures. Such standardization would support integration with established neuroimaging pipelines, improve reproducibility, robustness, and maintainability.

More broadly, the development and application of machine learning methods are facing a fragmented landscape, where different software is used in different use-cases. Competition among large corporate entities (e.g., technology companies) is also driving this fragmentation, as different entities try to position their tools as leading in the marketplace. In cases where models are shared not only as software, but also in the form of parameter values (or “weights”), the standardization of the format of model parameters, and of the metadata associated with these parameters becomes important. This is where industry standards such as the Open Neural Network Exchange (ONNX) standard [101] could play an important role. For these use-cases, there is much to benefit from the adoption of already-existing standards that apply broadly, rather than reinventing these standards for narrow applications.

Reviewer Comment 2.3

Another aspect that I would like to see discussed in some more detail is at the data acquisition stage: Tractography is rarely the sole purpose of acquiring diffusion MRI data. Often, quantifying tissue microstructure is another important goal, and introduces additional, sometimes conflicting requirements from a diverse set of respective methods. I expect that, in practice, attempts to standardize data acquisition for tractography will not succeed if they do not account for this.

Reply:

We thank the reviewer for highlighting this important observation. We have edited the manuscript to discuss the challenges that quantitative tissue microstructure characterization represents in tractography standardization in the “Data acquisition”

Powered by Editorial Manager® and ProduXion Manager® from Aries Systems Corporation

	section (L131-142):<br><br>Tractography is rarely the sole purpose of acquiring dMRI data. Quantitative characterization of tissue microstructure is often a concurrent goal, which imposes additional and occasionally conflicting constraints stemming from distinct acquisition requirements. As the sequences for probing the tissue microstructure get more sophisticated (e.g., using tensor-valued encoding), more variability can be expected to be present in the dMRI data, and more nuanced tractography reconstructions can be expected [44]. Thus, additional methodological development is needed to align such multi-dimensional acquisition protocols across sites, to manage scan time constraints while jointly supporting tissue microstructure modeling and tractography.<br><br>Reviewer Comment 2.4<br>--------------------<br><br>Finally, I would find it helpful if the "summary and recommendations" section were more clearly structured according to a separate discussion of a) consensus recommendations of best practices and b) open issues that require further work.<br><br>Reply:<br><br>Instead of restructuring the section entirely, and also in response to comments from Reviewer 1 (see responses to Comment 1.4 and Comment 1.11), we have now interspersed these open issues and added assessments of their feasibility into each point in the discussion, following the sections used in the body of the article.
**Additional Information:**	
**Question**	**Response**
Are you submitting this manuscript to a special series or article collection?	No
**Experimental design and statistics**<br><br>Full details of the experimental design and statistical methods used should be given in the Methods section, as detailed in our Minimum Standards Reporting Checklist. Information essential to interpreting the data presented should be made available in the figure legends.<br><br>Have you included all the information requested in your manuscript?	Yes
**Resources**<br><br>A description of all resources used, including antibodies, cell lines, animals and software tools, with enough information to allow them to be uniquely identified, should be included in the Methods section. Authors are strongly encouraged to cite Research Resource Identifiers (RRIDs) for antibodies, model	Yes

organisms and tools, where possible.<br><br>Have you included the information requested as detailed in our Minimum Standards Reporting Checklist?	
**Availability of data and materials**<br><br>All datasets and code on which the conclusions of the paper rely must be either included in your submission or deposited in publicly available repositories (where available and ethically appropriate), referencing such data using a unique identifier in the references and in the “Availability of Data and Materials” section of your manuscript.<br><br>Have you have met the above requirement as detailed in our Minimum Standards Reporting Checklist?	Yes
GigaScience has policies and guidelines in place for the use of generative AI-writing tools such as ChatGPT. If you have used such writing tools to assist with writing the manuscript this must be declared and cited in the text. Authors should not list AI-writing tools and other AI-assisted technologies as an author or co-author and should acknowledge that they are fully responsible for text generated or refined by AI-writing tools.<p><br><br>A summary of use (particularly in the introduction or among methods) needs to be included at the end of the paper, and the outputs should also be included as a supplementary file hosted in GigaDB or other open repositories. Please read our guidelines for more information. <p><br><br>By submitting to GigaScience, you are aware of the journal's AI-writing tools policy, and if you have declared use of such tools below, you have acknowledged this where appropriate in your manuscript and have made a summary of use and outputs available. <p><br><b>AI-assisted writing tools have been	No

Powered by Editorial Manager® and ProduXion Manager® from Aries Systems Corporation

used in the preparation of this manuscript?	

Powered by Editorial Manager® and ProduXion Manager® from Aries Systems Corporation

Revised manuscript

Click here to access/download;Manuscript;ist_su_what_needs_to_be_stand

GigaScience, 2023, 1–14

doi: xx.xxxx/xxxx
Manuscript in Preparation
Review

REVIEW

What needs to be standardized for reliable, reproducible, and robust tractography?

Jon Haitz Legarreta[1,2*], Simona Schiavi[3,4], Wei Tang[5], Garrett Banks[6], Matthew Cieslak[7,8,9], Kurt Schilling[10], Alberto De Luca[11], Jacques-Donald Tournier[12,13], John Kruper[14], Francois Rheault[15], Stamatios N. Sotiropoulos[16,17], Franco Pestilli[18,19], Jelle Veraart[20], Joseph Yuan-Mou Yang[21,22,23], Maxime Descoteaux[15], Sarah Heilbronner[6] and Ariel Rokem[14,24*]

[1]Department of Radiology, Brigham and Women's Hospital, Mass General Brigham, Boston, MA, USA and [2]Harvard Medical School, Boston, MA, USA and [3]ASG Superconductors S.p.A., Genoa, Italy and [4]Department of Computer Science, University of Verona, Verona, Italy and [5]Department of Psychological and Brain Sciences, Indiana University, Bloomington, IN, USA and [6]Department of Neurosurgery, Baylor College of Medicine, Houston, TX, USA and [7]Lifespan Informatics and Neuroimaging Center (PennLINC), Department of Psychiatry, Perelman School of Medicine, University of Pennsylvania, Philadelphia, PA, USA and [8]Penn/CHOP Lifespan Brain Institute, Perelman School of Medicine, Children's Hospital of Philadelphia Research Institute, Philadelphia, PA, USA and [9]Department of Psychiatry, Perelman School of Medicine, University of Pennsylvania, Philadelphia, PA, USA and [10]Department of Radiology, Vanderbilt University Medical Center, Nashville, TN, USA and [11]Image Sciences Institute, University Medical Center Utrecht, Utrecht, The Netherlands and [12]Department of Biomedical Engineering, School of Biomedical Engineering and Imaging Sciences, King's College London, King's Health Partners, St. Thomas' Hospital, London, UK and [13]Centre for the Developing Brain, School of Biomedical Engineering and Imaging Sciences, King's College London, King's Health Partners, St. Thomas' Hospital, London, UK and [14]Department of Psychology, University of Washington, Seattle, WA, USA and [15]Sherbrooke Connectivity Imaging Lab (SCIL), Department of Computer Science, Université de Sherbrooke, Sherbrooke, Québec, Canada and [16]Sir Peter Mansfield Imaging Centre, School of Medicine, University of Nottingham, Nottingham, UK and [17]NIHR Nottingham Biomedical Research Centre, School of Medicine, Queen's Medical Centre, Nottingham, UK and [18]Department of Psychology, The University of Texas at Austin, Austin, TX, USA and [19]Department of Neuroscience, The University of Texas at Austin, Austin, TX, USA and [20]School of Medicine, New York University, New York, NY, USA and [21]Department of Neurosurgery, Neuroscience Advanced Clinical Imaging Service (NACIS), Royal Children's Hospital, Melbourne, Victoria, Australia and [22]Neuroscience Research, Murdoch Children's Research Institute, Melbourne, Victoria, Australia and [23]Department of Paediatrics, University of Melbourne, Melbourne, Victoria, Australia and [24]The University of Washington eScience Institute, University of Washington, Seattle, WA, USA

*jhlegarreta@bwh.harvard.edu; arokem@uw.edu

Abstract

Tractography is a key component of efforts to map brain connectivity. As a rapidly-evolving field of neuroscience, current tractography methods are diverse, often varying across research laboratories and different software pipelines. Therefore, it suffers from a lack of standardization leading to inconsistencies in results, which can limit reproducibility, and affect the robustness needed for research and clinical applications of these methods. Variability in data acquisition procedures, inconsistencies in spatial referencing schemes and implementations, and anatomical heterogeneity —at the individual level, across the lifespan, and across species— hinders comparative analyses. Additionally, the lack of consensus on best practices complicates the development of robust automated quality control pipelines and limits the clinical translation of tractography-based procedures. Establishing standardized protocols for acquisition, preprocessing, and tractography reconstruction are critical towards enabling reliable tract-specific analyses, facilitating cross-study harmonization, and supporting replicable large-scale population studies. The present article provides an overview of the current challenges in tractography standardization and identifies the key aspects that require standardization for reliable, reproducible, and robust tractography.

Key words: Neuroanatomy; standardization; tractography; brain connectivity; white matter; computational neuroimaging

Background

Understanding ~~the~~ human brain anatomy across different organizational levels is a central goal of contemporary neuroscience. There is increasing evidence that structural connectivity across the white matter underlies many of the capacities of the living brain and that their physical properties are linked to human brain health [1, 2, 3, 4]. This understanding has grown with the development of methods to measure brain connections and it has also fueled a new generation of data collection and data analysis methods. Magnetic resonance imaging (MRI) measurements are non-invasive and can be used to study brain connectivity *in vivo* [5]. These data are complemented by other invasive techniques that highlight brain connections by using specific chemical tracers, histological stains and molecular markers, by dissecting the tissue, and by direct optical observation [6, 7, 8, 9, 10, 11, 12, 13], or using X-ray imaging methods [9, 14]. *Computational tractography* assesses the location, direction and pattern of brain connections estimating their trajectories (with an individual trajectory often referred to as a *streamline*). However, as the methods evolve and the promises from their application grow, it is important to take stock of challenges that hinder leveraging their full potential. Other works have examined challenges related to accurate delineation of brain connections [15, 16, 17, 18] or challenges related to definitions of certain anatomical concepts in such data [19, 20]. The present paper focuses specifically on the challenges related to standardizing representations of tractography-derived brain connections in digital formats.

These challenges have become increasingly pressing as the field has transitioned in recent years towards larger and larger datasets. This increase in data volume and complexity arises from large-scale data acquisition projects, with thousands of subjects, on the one hand. Examples of these include the Adolescent Brain Cognitive Development (ABCD) [21], Healthy Brain and Child Development (HBCD) [22], and Healthy Brain Network [23, 24] studies, as well as the UK Biobank [25], and many others. On the other, it arises from the increase in the resolution as well as coverage of measurements. These larger data have led to the application of data-driven discovery methods, where consistent and comprehensive standards are necessary.

Establishing standards and best practices supports transparent, reproducible and robust research through application of the FAIR (Findable, Accessible, Interoperable, Reusable) principles [26]. In addition, establishing usable standards can enable research that is otherwise difficult, making things that are hard easier, and making things that are otherwise impossible practical. With the recent establishment of the International Society for Tractography (IST) and its standardization unit (members of which are authors of this paper), together with several consensus efforts in diffusion MRI (dMRI) acquisition and processing [27, 18, 28] led by the Diffusion Study Group of the International Society for Magnetic Resonance in Medicine (ISMRM), we see an opportunity to advance broadly-applicable and widely-accepted community standards that will pave the way towards future research of brain connectivity.

This work introduces some of the current challenges that arise from gaps in standardization for tractography and offers some potential solutions to them. We provide a set of recommendations for the broader research community to pursue the solutions that we identify. The recommendations presented herein will improve the rigor and impact of work that uses brain tractography and will enable better understanding of brain connectivity. The paper is organized by the different stages of the data life-cycle and a range of domains in which standardization poses challenges (Figure 1), concluding with a set of recommendations (section Summary and recommendations).

Challenges and Solutions

Reliability, reproducibility, and robustness in tractography encompass multiple methodological dimensions. They manifest across the full data life-cycle of tractography, including acquisition, processing, and analysis. They also manifest in inferences applied across different species, different types of measurements, different ages, and across basic research and clinical application. In this context, we define *reliability* as the consistency of the reconstruction under fixed conditions (e.g.,repeated measurements on the same participant, repeated computations of the same reconstruction method, and repeated use of the same tractography algorithm). *Reproducibility* (sometimes also referred to as "*replicability*", see [29]) is the ability to obtain equivalent results across sites, operators, and implementations, while preserving the expected anatomical observations (e.g.,consistency across software packages, versions, and hardware platforms, consistent results obtained from the same shared data, etc.). Finally, robustness characterizes the stability of tractography algorithms in the face of varying conditions (e.g.,noise perturbations, multi-site or multi-vendor datasets, etc.). As we will demonstrate in addressing a range of practical and conceptual challenges, standardization is essential to achieve these goals. Therefore, as we survey a range of challenges and proposed solutions, we will highlight key factors influencing these aspects and propose ways to increase them.

Data acquisition

The utility of standards starts with the moment that data are created. Different experimental methods produce tractography data, and considerations for standardization may be different for each one of these. Furthermore, the creation of some data is already governed by numerous existing standards. For example, in a large number of cases, MRI data is acquired following the Digital Imaging and Communications in Medicine (DICOM) standard [30]. Similarly, modern storage and sharing of reconstructed (raw or processed) research MRI data is standardized by the Brain Imaging Data Structure (BIDS) specification [31], including several extensions for dMRI and brain connectivity (e.g., [32, 33]). These standards facilitate not only the structured storage of the data itself, but also prescribe necessary metadata. In principle, this should facilitate the standard processing in subsequent steps, but undocumented and poorly standardized procedures are typical of many different experimental techniques and a source of unwanted variability.

Differences across datasets can also arise due to a range of non-biological variables, such as variation in acquisition protocols, scanner hardware, reconstruction pipelines, and tractography workflows, that are hard to reconcile even given full description of the data acquisition. These sources of variability can have significant impact on downstream tractography results, particularly in multi-site studies and large-scale datasets. For example, acquisition resolution, diffusion sampling schemes, and vendor-specific differences introduce variability in the spatial geometry and microstructural characteristics of reconstructed brain tracts that compromise reproducibility and robustness [34, 35, 36, 37, 38].

Statistical methods for *harmonization*, which aim to eliminate such differences at the level of the raw data [39], provide promising solutions to some of these challenges, but the intricacy of downstream tractography analysis workflows poses further challenges. These workflows involve subjective parameter choices, user-defined constraints, and possibly different priors or anatomical reference definitions that influence reproducibil-

Compiled on: February 2, 2026.
Draft manuscript prepared by the author.

ity and anatomical fidelity. Cross-scanner and cross-vendor effects propagate through these pipelines, leading to inconsistencies in reconstructed geometry, volume, and quantification of tract properties. Recent efforts to standardize processing pipelines and implement robust quality control protocols across sites have shown promise in reducing these inconsistencies, thus improving robustness [40, 41, 42, 43]~~, but~~.

Tractography is rarely the sole purpose of acquiring dMRI data. Quantitative characterization of tissue microstructure is often a concurrent goal, which imposes additional and occasionally conflicting constraints stemming from distinct acquisition requirements. As the sequences for probing the tissue microstructure get more sophisticated (e.g.,using tensor-valued encoding), more variability can be expected to be present in the dMRI data, and more nuanced tractography reconstructions can be expected [44]. Thus, additional methodological development is needed to align such multi-dimensional acquisition protocols across sites, to manage scan time constraints while jointly supporting tissue microstructure modeling and tractography.

Despite the progress that has been achieved, further work is needed to ~~develop automated and reproducible methods for harmonizing tractography .~~ provide an adequate basis for tractography. Using BIDS for neuroimaging data sharing offers a high-level of consistency; however, its adoption across pipelines is not yet all-pervading, and its implementation is not uniform. Furthermore, although the diffusion extension proposal has been submitted for inclusion in the standard, it only covers scalar derivatives and tensors. The tractography extension has not been submitted yet for inclusion. Similarly, vendor-agnostic, standardized sequences are yet to be implemented across the variety of scanning hardware and protocols, and be validated extensively. Limited support and interest for regulatory clearance entail additional hindrance to their wide adoption [45]. Finally, harmonization is still a topic under active research, or present limitations (e.g.,fixed, well populated site numbers, homogeneous populations) that have yet to be overcome. Thus, significant methodological gaps remain in diffusion MRI data acquisition that limit the development of automated and robust tractography downstream.

Standard operating procedures (SOPs) are necessary to remove variability at data collection and provide some level of reliability in tractography reconstruction from a given study. SOPs have been employed to describe the imaging and processing protocols for large-scale studies (e.g.,the Human Connectome Project (HCP), the ABCD study, etc.); more recent studies (e.g.,the HBCD study, or the Human Connectome PHantom Study (HCPh)) have adopted contemporary web technologies to modernize data presentation. Smaller-scale, single-site studies would likewise benefit from this practice, contributing to improved transparency in reporting and reproducibility. Similarly, standardized data and metadata records and organization (e.g.,through BIDS) are required to ensure reproducibility. This includes developing the extensions to derivatives (e.g.,fiber orientation distributions) that are employed to generate tractography results. Finally, harmonization solutions (within and across scanners and sites, across acquisition upgrade packages, etc.) are required to resolve data source variability. Combined with the adoption of unambiguous, self-reporting data formats (see Spatial coordinates), these measures would mitigate practical barriers and contribute to reducing overhead in multi-center studies with disparate settings.

Quality control

There are many well-established pipelines for quality control (QC) of raw MRI data [46], including dMRI data [47, 48], as well as data that has undergone initial pre-processing (i.e., denoising, correction of motion and eddy current artifacts, and removal of other artifacts) [49, 50, 24]. Presumably, QC procedures that are applied at these early stages should catch many of the issues that would impact subsequent analysis steps. However, QC can and should be done at multiple different stages of the analysis, because errors can occur at each one: e.g., registration between different imaging modalities, separation of the image into different tissue types and regions of interest for tractography initiation, etc. Often times, many of these issues only become apparent when computational tractography is conducted. This means that QC of tractography results is still necessary. In practice, QC of computational tractography pipelines is often done through visual examination of whole-brain tractograms (the collection of all streamlines estimated in a single brain), or by their ability to identify the locations and trajectories of large, well-known tracts. However, this approach is challenged by ~~low~~ high inter-rater variability and an apparent lack of consensus on the structure of these tracts, even in the same set of streamlines [19, 20]. ~~Additionally, although there are many advanced tractography visualization tools and paradigms [51], and despite some pipelines offering automated reporting, their use for QC purposes has not been thoroughly studied. As a result, best practices for excluding low-quality results based on these visualizations remain unclear.~~

Another approach for QC of tractography results is through numerical validation. In this approach, individual streamlines and sets of streamlines are subjected to statistical evaluation with respect to the measured data. In these methods [52, 53, 54, 55, 56, 57] individual streamlines or sets of streamlines are given objective scores, based on how well their trajectory conforms to the data that was used to generate the streamlines (e.g., in contrast to smoothness constraints, anatomical constraints, or randomness that is introduced in the process of tractography). Other methods use machine learning and deep learning techniques to filter streamlines based on their geometric properties or a representation of them [58, 59]. The benefit of these approaches is that they provide objective numerical values that can then be used as benchmarks for QC procedures. Overall, the field would benefit from more studies that demonstrate the utility and sensitivity of different QC procedures in relevant scientific inferences [60, 61], including validation by reference to known anatomical structure, and via the detection of individual differences related to development, aging, or clinical conditions [62].

Taken together, progress has been limited in tractography QC standardization practice and adoption levels. Although there are many advanced tractography visualization tools and paradigms [51], and despite some pipelines offering automated reporting, their use for QC purposes has not been thoroughly studied. Similarly, validating numerical tractography QC methods is still impacted by the inability to have gold standards on real brain data, and the lack of anatomical measures defining the success of a tractography method. As a result, best practices for excluding low-quality results based on either visual or numerical reports remain insufficiently established. Reliable tractography would benefit from clearly defined, shared criteria determining the quality of results, including standardized reporting. Additionally, universally shared phantom, and synthetic data, as well as anonymized *in vivo* and *ex vivo* data from both healthy and diseased participants are required to guarantee a common ground for quality control of a given tractography pipeline. This includes sharing data in software-agnostic repositories (e.g., OpenNeuro or Zenodo). Note that this extends beyond datasets used across international challenges that quantify tractography results only within a particular dataset (see, for example, [63, 17, 64, 16]).

Spatial coordinates

Representing spatial coordinates in an unequivocal frame is a known challenge in many fields of science that deal with spatial

information. This is a well-recognized issue in anatomical studies of the human brain, where different types of information (e.g., structural, functional, physiological, etc.) need to be integrated. Given the variety of information sources that are involved, each acquisition modality having particular spatial properties, the potential for errors is high and the consequences detrimental to the study of brain connectivity [65].

Tractography algorithms typically output data as a set of three-dimensional (3D) coordinates that represent the trajectory of particular brain white matter tracts (see Figure 2~~A,B~~a,b). In reconstructing and interpreting these structures, several coordinate frames need to be considered. One coordinate frame is the one that refers only to the individual participant's brain, allowing to locate a structure relative to a particular anatomical landmark. Another coordinate frame is the position of the individual's brain within the scanner, i.e., relative to the origin of the scanner's fixed reference frame (e.g., the iso-center of the MRI's bore). Another coordinate frame to consider is that of the grid of *voxels* that are included in the measurement itself.

Volumetric imaging data follows particular anatomical conventions that facilitate relative positioning and orientation. These conventions split the space according to three orthogonal planes, namely *axial*, *coronal*, and *sagittal* planes (Figure 2~~A~~a). When storing real-world spatial data into discrete imaging data, the information can be arranged according to a particular basis. Two typical conventions are *RAS+* (Right, Anterior, Superior, the + sign indicating that coordinates increase from left to right, posterior to anterior, and inferior to superior) and *LPS+* (Left, Posterior, Superior; the same principle applying to the + sign). If no metadata is accessible, applications reading tractography results may assume the incorrect convention (Figure 2~~C~~c).

At times, the conventions implied by a particular format are not made apparent in the corresponding documentation. A typical example of this issue is the definition of the origin of a voxel: some tractography formats consider the origin being at the center of the voxel, whereas others consider that it lies at the corner. This may arise in the correspondence between streamline coordinates and other coordinate-based (e.g., volume-based) data: unless this is carefully considered, a half-voxel shift will exist between the streamlines relative to their intended world coordinates. Visual inspection is not always sufficient to notice this (Figure 2~~D~~d), and such small systematic errors can have significant consequences in downstream applications. Some tractography data processing tools may fail to read streamlines that, due to not accounting for this shift, lie outside the volume bounding box. In connectivity analyses, these errors may lead to mismatches between surfaces or label maps reconstructed from T1-weighted data and the tractography space, resulting in incorrect streamline assignments and spurious inter- or intra-hemispheric connectivity patterns.

Finally, streamlines generated by tractography algorithms may have their own positions relative to each of the other coordinate frames, and may be tied to an additional piece of data (e.g., another data file). The units used to store their position may also differ from the units used to refer to the measurement (discrete voxels, or continuous mm). Incongruent coordinate frames and units can lead to errors such as the ones shown in Figure 2~~E~~e.

Overall, despite a well-established understanding of the coordinate frameworks involved in tractography reconstruction, current pipelines continue to rely on implicit or insufficiently documented conventions. Clear and unambiguous guidance for debugging, validating, and converting data across coordinate spaces and formats remains lacking. Consequently, meaningful progress in this area has remained limited. A tractography data standard that explicitly and unambiguously documents its spatial coordinate framework (e.g., according to the BIDS convention), and version-controlled, general resources (i.e., not tied to a particular processing package) are required. This includes testing data stored in software-agnostic repositories, and guidelines towards conformance, error checking and conversion of retrospective data.

Translation across species and methods

Anatomical tract-tracing methods involve injecting a dye or tracer into a living brain, waiting a set period of time, then extracting the brain for histology and microscopy [66]. Thus, these methods provide information that is not accessible with MRI but, for obvious reasons, cannot be performed in humans. Thus, tract-tracing is performed in brains from non-human animals, such as primates and rodents. Combining these methods with dMRI in non-human models provides a unique opportunity to verify dMRI-based tractography against anatomical gold standards, and to iteratively improve these methods. Several large-scale efforts aim to provide high-quality dMRI along with microscopy and tracers in the same non-human primate brains (e.g., the Large-scale imaging of neural circuit (LINC) BRAIN CONNECTS center, or the Center for mesoscale connectomics (CMC)). Openly accessible resources from these consortia and others (such as PRIME-DE [67], or the BRAIN/MINDS portal [68] will be invaluable in optimizing, standardizing, and developing the next generation of tractography approaches. Using unified methods that combine these multi-modal and multi-scale data would ultimately enable informed judgment about cross-species homologies/dishomologies. However, this process is complicated by several factors. Non-human brains —even in our closest primate relatives— are not simply smaller, but also have different morphological and biophysical properties. ~~Integration of data between methods is also hampered by differences in data representation: anatomical structures (gray/white matter boundaries, nuclei volumes, cells, fibers,) are represented in standard anatomical reconstruction software (Neurolucida) that interface with microscopes as points, contours and surfaces. Downstream processing software such as IMOD [69] inherits the file formats that organize these structures. This is conceptually different from the MRI file conventions, which use voxels as their primary representational unit.~~

This poses foremost a challenge to the validity of inferences between species. Integration between human and non-human primate data ~~also~~ remains conceptually challenging~~. Non-human~~, because non-human anatomical knowledge relies heavily on atlases based on cytoarchitectonics (e.g., ~~[70]~~[71]). These do not directly align with the common atlases used in human MRI studies (e.g., [72]). While cytoarchitectonics-based human brain atlases have been developed [73], some even with a particular stress on cross-species homology [74], these have not been integrated with widely used neuroimaging tools. At the same time, common coordinate frameworks for non-human primates are beginning to emerge (see, for example, [75]), in some cases with the explicit goal of establishing a direct relationship with the developing human connectome [76]. Tractography fingerprinting [77], i.e., using white matter bundles as latent landmarks to assess similarities and divergences across the two species, is geometry-agnostic and can provide ~~solutions to this challenge~~ additional solutions to the challenge of integrating cross-species coordinate frameworks.

Integration with studies in rodents are further complicated by the extent of species dishomology. Their white matter bundles are organized very differently from primates' [78], and their lissencephalic brains mean that many of the MRI-based tractography methods that work in primate brains do not translate. On the other hand, there are a host of genetic, molecular, and imaging tools readily available for circuit characterization and manipulation in mice that are not feasible in humans or non-human primates. Thus, merging dMRI tractography with tract-tracing, spatial transcriptomics, and imaging data in the mouse model could be highly valuable. However, while anatomical atlases in primate models

have a long history (e.g.,[79, 71]), just two decades ago, there were virtually no standard frameworks (atlases, metadata, structural ontologies, etc.) for the mouse brain. However, with major investments in generating large-scale datasets of mouse brain transcriptomics, cell typing, connectivity, and function, the need for a standard framework became overwhelming. The ~~Allen Mouse Brain Common Coordinate Framework (CCF~~Allen Mouse Brain Common Coordinate Framework (CCF) [80] (current version CCFv3 [81]) has emerged as a standard framework for organizing data about mouse brain connectivity. The enormous success of this framework illustrates the value of standardization. An extensive informatics pipeline at the Allen Institute for Brain Science allows users to download everything from raw images to structural summaries of connectivity, and tools that use the CCF can integrate data from multiple sources. However, these tools are not currently interoperable with MRI data, aside from at the crudest (region-to-region connectivity summaries) level.

The methods employed to map connectivity across species also differs in profound ways due to the nature of the acquired data; while axonal pathways in the rodent brain are reconstructed from optical data (e.g.,electron microscopy), human brain pathway reconstruction using tractography employs dMRI data. Thus, the data representation structures employed are different: in rodent data, anatomical structures (gray/white matter boundaries, nuclei volumes, cells, fibers, etc.) are represented in standard anatomical reconstruction software (e.g.,Neurolucida) that interface with microscopes as points, contours and surfaces. Downstream processing software such as IMOD [69] inherits the file formats that organize these structures. This is conceptually different from the MRI file conventions, which use voxels as their primary representational unit. The nature of the contrast in the acquired data necessitates the development of distinct methods (e.g.,segmentation and stitching as opposed to streamline propagation), introducing a supplemental layer for divergence. As a consequence, the progress to bridge the gap in tractography data representation and software across species and imaging modalities has been limited. Addressing these challenges requires a comprehensive approach that includes standardized multi-modality and multi-resolution data acquisition, advancements in hardware for efficient *in vivo* scanning, essential validation studies, and methodological innovations, such as automated tools for establishing a common framework of correspondence.

While the challenges to validity and generalization are evident, these issues also raise challenges to robustness. This is because the differences in measurement tools and in analysis methods need to be thoroughly adapted and validated when they are used in species other than the ones on which they have been developed. Examples in which tractography tools that were developed for use in human dMRI data were adapted to be used in non-human data [82] suggest that there is moderate progress on this challenge.

The scale of data

Like many other datasets of spatial biological information, tractography data can take up large amounts of storage. A typical dMRI tractography file occupies several gigabytes (GB) of data. The size depends on the spatial and angular resolution of the diffusion data, the sampling density employed to reconstruct streamlines, the number of streamlines that were generated, the data associated with each point or each streamline (e.g., scalars that represent tissue properties along their length etc.), as well as the precision employed to store the data. Additionally, the size of the tractography output may also depend on the resolution of the acquisition. For example, with sub-millimeter resolution MRI acquisitions, the size of tractograms can easily reach the terabyte-scale (TB) [83]. With other modalities that provide even higher image resolution in *ex vivo* tissue samples, such as polarization microscopy [7, 8, 10, 12], X-ray microcomputed tomography [84], X-ray scattering [9], or synchrotron technology [14], data is orders of magnitude larger (see, for example, [85, 86]). Manipulating such data volumes pose a challenge in terms of the required hardware and computational power. As imaging acquisition hardware evolves, and data storage capacity and physical memory limits increase, current analysis tools may buckle under the strain~~, and efforts will be required to address these challenges~~. This means that some approaches, which work well with smaller datasets, can become infeasible when translated into larger data, threatening robustness. A few technical and standardization developments may ease this strain. First, tractography data lends itself to compression, with *linearization*, where collinear points are discarded (with some tolerance threshold), being the fastest and most efficient approach ~~[?~~ 87~~, 88]. Additional compressions~~ [89, 87, 88]. Additional compression is provided by using lower precision numerical representations (i.e., 16-bit floating point precision, instead of the extended 64), which can provide significant space saving, without much loss of anatomical information [90]. Similarly, some visualization techniques such as level of detail, occlusion culling or visibility-based rendering optimization aim to save computational resources by avoiding rendering data that is occluded by other objects or by rendering it at lower resolutions (see [91] for a recent method). Second, new file formats and distributed computing paradigms offer opportunities to scale compute to very large datasets. As an example, a newly-proposed file format, *TRactography eXchange* (TRX) [92], was designed to make it easy to create large tractograms with minimal random access memory requirements. This enables memory mapping, providing a convenient and efficient way to access data directly from disk.

Software and data-format enhancements have so far enabled only limited progress, due to heterogeneous precision adjustment across pipelines and partial adoption of the TRX file format. Data storage and visualization remain major bottlenecks, and significant advances have relied primarily on improvements in computational power, with efficient mapping of brain connectivity at scale being possible on supercomputing hardware [93] or using Graphics Processing Units (GPUs) [94, 95] . Distributed data storage (e.g.,with zarr [96]) could offer an attractive possibility for work with very large datasets. Effective visualization of memory-intensive tractography data will require implementations in low-level programming languages, such as C++ or Rust, and/or compatibility with GPU hardware acceleration.

Integration of modern machine learning methods

The increasing adoption of machine learning and artificial intelligence (ML/AI) methods in dMRI and tractography has raised new challenges and exposed limitations in current standardization practices. Modern ML/AI methods require large, diverse, and well-curated datasets to achieve robust generalization. These requirements compound many of the challenges described. For example, in the absence of reliable and automated QC tools, labor-intensive quality control procedures are required. Spatial coordinate inconsistencies within large heterogeneous datasets may cause deep learning models to fail to converge. Similarly, poor data organization and metadata standardization —due to both poor compliance with BIDS and the preliminary status of the related extensions—hinder reproducibility and compromise downstream analyses, complicating cross-study comparisons. Inadequate handling of data splits can lead to train–test leakage [97]. Such leakage risks inflating reported performance and obscure the true generalizability of ML/AI-based tractography methods. Though there are some annotated datasets that can be used as the basis for training ML/AI , the scarcity of large, well-annotated, and openly shared tractography datasets

—particularly multi-site and longitudinal datasets that include non-healthy individuals for clinical relevance —constrains the development of robust and reliable tractography.

Furthermore, there is need for standardization within the ML/AI methods themselves. ML/AI-based tractography techniques exhibit substantial heterogeneity in model architecture, input representation, and learning objectives, often making direct comparisons difficult (see [97, 98, 99] for relevant review works). Existing approaches range from voxel-wise classifiers and streamline propagation networks to graph-based and geometric learning formulations. As such, the input to the model may be the raw diffusion signal, the local orientation information represented as spherical harmonics coefficients or fiber peaks, etc. Additionally, the contextual information to regularize the optimization process is also heterogeneous across methods, and includes tissue maps, neighboring voxel information, etc. Other approaches avoid reliance on the modality that sensitizes axonal pathway architecture (e.g.,dMRI or other), and estimate tractography without explicit orientation data. These factors result in differing dataset requirements for training various models, often leading to substantial increases in computational requirements, both in terms of storage and processing resources. The high computational cost —including the need for specialized hardware, such as GPUs—makes regular testing and evaluation of methods expensive and resource-intensive.

This heterogeneity is compounded by the absence of universally accepted anatomical ground truth for white-matter pathways, a limitation that challenges the current tractography landscape. The evaluation of AI-based tractography algorithms frequently relies on indirect metrics, simulated phantoms, or expert-defined references, each with inherent limitations. In this context, the absence of standardized reporting conventions is particularly problematic for the evaluation of AI models. Although volume- and streamline-oriented measures are well-established since landmark studies [17, 100], evaluation tools remain poorly maintained, with most pipelines relying on bespoke implementations. Additionally, the assessment of application-specific derivatives —such as tract-specific metrics, connectivity measures, or quantitative microstructural parameters—is often inconsistent or omitted entirely. Insufficient reporting of scanner or session biases can also mask site- or protocol-specific effects that may dominate learned representations, resulting in performance gains that are often difficult to attribute to genuine methodological advances.

The result is a fragmented landscape in which performance claims are highly dependent on specific datasets, tasks, and evaluation choices, increasing the difficulty of reliable evaluation. Together, this underscores the necessity of standardization for AI-based tractography, encompassing access to well-annotated and fully described datasets, common validation frameworks with standardized benchmarks, metrics and reporting procedures. Such standardization would support integration with established neuroimaging pipelines, improve reproducibility, robustness, and maintainability.

More broadly, the development and application of machine learning methods are facing a fragmented landscape, where different software is used in different use-cases. Competition among large corporate entities (e.g., technology companies) is also driving this fragmentation, as different entities try to position their tools as leading in the marketplace. In cases where models are shared not only as software, but also in the form of parameter values (or "weights"), the standardization of the format of model parameters, and of the metadata associated with these parameters becomes important. This is where industry standards such as the Open Neural Network Exchange (ONNX) standard [101] could play an important role. For these use-cases, there is much to benefit from the adoption of already-existing standards that apply broadly, rather than reinventing these standards for narrow applications.

Translation of tractography methods to clinical applications

Tractography is used clinically to aid in the planning and execution of neurosurgical procedures [102]. Despite constituting a useful resource for improving neurosurgical outcomes and mitigating the likelihood of postoperative complications, its use remains limited [103]. Diffusion tensor-based deterministic tractography remains the prevalent tool in neurosurgical preoperative planning, largely because it is supported by many commercially available navigation platforms [104].

One use is during the resection of epileptic foci, or brain tumors [105, 106]. In this case, a surgeon might utilize a different surgical approach to the tumor to avoid certain white matter tracts, particularly those involved with motor, language and visual function. This is especially the case in slow growing tumors and pediatric developmental abnormalities, where the standard anatomical organization of white matter pathways can be significantly altered while remaining functional. To this end, tractography-guided brain tumour resections rely on functional brain mapping through direct brain electrical stimulation to confirm white matter tract positions and resection functional boundaries during awake surgery. In other instances, tractography is utilized for precision targeting in stereotactic procedures such as deep brain stimulation and focused ultrasound, such as when localizing the dentatorubrothalamic tract, a neuromodulation target for treatment of essential tremor and tremor-dominant Parkinson's disease [107, 108, 109].

Despite these uses, the reconstruction and visualization of complex fiber architecture (e.g.,crossing, fanning, and bending pathways) is still incomplete in clinical practice due to the limitations of the tensor model. Despite the availability of sophisticated diffusion and tractography methods, variability in results and large false positive rates hinder their adoption for clinical applications. This is especially pronounced when examining fine-scaled structures, such as cranial nerves, and highlight the sensitivity of the tracking parameters with respect to the structures of interest Jacquesson et al. [110]. Additionally, results provided by intraoperative tractography are constrained by the limited acquisition and processing time.

Standardization of clinical workflows is complicated, because these applications require thorough validation and consensus among experts. This requires processing software to remain very stable over time, and makes updates to the software very difficult in these settings. Variable imaging protocols also make consensus more difficult to reach (see section Data acquisition). Benchmarks and standard validations methods are very challenging to formulate, because clinical use-cases are diverse, and there are concerns that methods may be affected by the presence of pathologies. Furthermore, clinicians (e.g., radiologists or neurosurgeons) may use bespoke pipelines developed in-house to perform tractography, making translation across settings difficult. Finally, depending on the clinical setting and operative urgency, some processing pipelines that are common in research settings may be too time consuming and/or computationally intensive. ~~To help~~

Thus, limited progress has been made to create reference procedures to bridge basic research in tractography and clinical work~~,—~~. Consistent acquisition and processing protocols ~~need to be made more consistent across settings.~~ are required to produce tractography results that can be translated across clinical settings. In addition, methodological innovations are needed for time-constrained clinical acquisitions —such as optimized pulse sequences, undersampling strategies, and super-resolution techniques—that enable robust tractography results, alongside tractography methods that remain reliable when employed on lower-quality data. Additional measures to support clinical adoption include systematic reporting of reconstruction uncertainty across varying data quality

conditions (e.g.,noise levels) and reconstruction settings (e.g.,seeding strategies). Finally, establishing reliable proxies for clinical outcomes is essential to validate the reliability of fiber reconstruction techniques.

Standardization of methods throughout the lifespan

The brain changes significantly throughout the lifespan, and there is a wealth of evidence that lifespan development of brain connections is linked to health outcomes (see [111] for a review). The changes in the properties of the brain also pose challenges for standardization of methods across different epochs of ~~the~~ life. For example, studying early life brain development is challenged by the simple fact that the brain is substantially smaller at birth, with total brain volume more than doubling during the first 12 months of life [112]. This poses a particular challenge to the use of standard atlases that localize certain structures, as these atlases are usually constructed based on healthy young brains. To address this challenge, researchers have been developing detailed and time-resolved atlases in early life [113, 114] and even during gestation [115]. Furthermore, while the major brain tracts are already established at birth [116], it is not simply the case that the infant brain is a scaled-down version of the adult brain. There is some evidence that the curvature of some structures is different in this early phase relative to later development [117]. In addition, the tissue properties of brain connections in early life are quite different than those of a more mature brain. This can pose a challenge to the use of standard tractography methods, which sometimes rely on assumptions about the biophysics of the tissue, which may not hold [118]. Similar changes apply in aging, as tissue properties of the white matter change again with age in a manner that can impact standard tractography methods [119]. The challenge of studying the developing and aging brain often intersects with challenges related to harmonization (mentioned in section Data acquisition) because it is difficult to obtain a large sample that covers all ages within a single study, necessitating integration of data across studies and differing acquisitions.

Progress in offering authoritative, centralized and standardized resources for brain connectivity mapping across the lifespan has been limited; as an example, the extension of the BIDS standard to describe atlases does not, at the moment, cover tractography templates. Standardizing tractography across the lifespan will require creating such templates, generating normative derivatives linking structural connectivity to tissue microstructure (tractometry), providing uniform method implementations, and guidelines for calibrating processing parameters.

Summary and recommendations

Despite the above challenges, if a series of recommendations are followed, tractography reconstruction and interpretation can be highly consistent and robust. Below we provide a set of recommendations towards building standardized, reliable and robust tractography data reconstruction and sharing procedures. We also discuss the feasibility of the suggested measures in terms of the effort (minimal, moderate, substantial) required to implement them.

i. Implement standard operating procedures ~~(SOPs)~~ [120] during data acquisition, i.e., written sets of instructions that specify the processes that take place during the measurement. Feasibility: a moderate effort is required to implement centralized SOPs towards tractography standardization. That said, some examples of SOPs for tractography data acquisition [121], quality assurance [48] and analysis [122] already exist (albeit, with a focus on dMRI), and could easily be adopted by the community.

ii. Adopt vendor-agnostic, open-source pulse sequence design and reconstruction frameworks (e.g., Pulseq [123, 124]) to facilitate more similar results across the instruments of different vendors. Feasibility: a substantial effort is required to make vendor-agnostic pulse sequences extensive within the data acquisition hardware, as it involves vendor support and validation towards regulatory clearance.

iii. Advance BIDS-compliant standards for tractography inputs and outputs. Akin to a wide range of neuroimaging use-cases, including human electrophysiology and microscopy, where BIDS has demonstrated its utility, a tractography-specific extension is needed towards reliable, reproducible, and robust tractography data representation and sharing. Note that the particular adoption of the BIDS specification for tractography should not prevent software from processing isolated tractography files. Feasibility: As of writing of this article, the BIDS tractography extension is being drafted, and a small effort is needed to complete it.

iv. Quality control. Standardized quality control procedures need to be fostered including automated procedures at every stage of processing. Further research on the impact of different decisions in QC is needed, to ultimately develop guidelines for best practices. Feasibility: due to the challenges inherent to tractography validation, a moderate effort will be required to define consensus measures and procedures towards implementing standardized QC protocols and pipelines.

v. Make analysis tools that are flexible to be used across different data acquisition methods, different species and different settings. FAIR-Software principles can be applied, to make sure that methods are transparent, rigorously designed and available to the community [125]. Feasibility: a substantial effort in validation is required to generalize tools across methods, species and settings. This may include generating publicly shared, reproducible records of the datasets and procedures employed.

vi. Scalability. Software and platforms for sharing and computing on brain connectivity data need to be built with the large datasets of the future in mind, and with the ability to scale to much larger data than is currently available, in anticipation of the inevitable deluge of data that is expected to occur. Infrastructure for distributed and cloud computing includes evolving standards for representation of large array-based [126], tabular [127], and even trajectory data [128] in these kinds of systems, and formats for representation of tractography data could be adapted to capitalize on these developments. Feasibility: a moderate effort can be expected to design frameworks capable of handling large volumes of data. This effort is related to the development of data representation standards inherent to tractography, and the reliance on the development of general purpose, advanced scientific software.

vii. Create and share annotated datasets for machine learning training. For improved interoperability and reproducibility, use industry standards for machine learning methods, such as ONNX and widely used sharing platforms for ML/AI models and tools, such as Hugging Face. Feasibility: These both require minor efforts on the side of scientists and researchers who develop these methods.

viii. Future-proof standardized file formats. The community needs to advance new file formats that address the needs for consistent explicit tractography data spatial representation, while also supporting the needs for new large-scale datasets. This includes standardized, traceable, openly available, and well-documented specifications, including community-sustained conversion information and tools. We identify the nascent TRX file-format as a format that has the potential to address many of the issues raised here, specifically because it was developed taking these aspects into consideration. Feasibility: a minor

effort remains to be done in terms of documentation of the TRX file format. A few initial studies already provide evidence for the suitability of TRX for reliable, and robust tractography data representation [90, 129]. A moderate effort will be required in order to incorporate the format as the output of existing software pipelines, which would make it more widely used.

ix. Prioritize the articulation of clearly specified validation frameworks with common benchmarks and metrics, alongside the development of centralized, consensus evaluation tools. Importantly, these will enable unambiguous assessment of relevant aspects like heterogeneous scanners, acquisition protocols, and imaging sources, critical to assess reliability and robustness of tractography. Feasibility: a substantial effort will be required to specify the resources —including data, metrics, and technological requirements—and to operationalize and sustain the resulting infrastructure.

x. Build the bridges between research and clinical tractography: Delivery of advanced methods into clinical practice can be facilitated by standardizing workflows, and by increasing the interoperability between different parts of the clinical informatics infrastructure. For example, via integration of visualization into surgery image-guided systems, into Picture Archiving and Communication Systems (PACS) used in clinical, and into electronic medical records [130]. This will also set the scene to improve the bench-to-bedside pipeline of new computational methods. Feasibility: while research and clinical requirements remain different, the usefulness of increasingly more anatomically reliable tractography methods needs to be translated into clinical practice. Substantial efforts in terms of validation and collaboration with vendors can be foreseen.

Data availability

Not applicable.

Code availability

The plots in Panels a, c, d and e in Figure 2 were created based on actual human brain *in vivo* diffusion MRI tractography data cases and the software openly available at https://github.com/International-Society-for-Tractography/Standardization-Position-Paper under an Apache 2.0 license.

Declarations

List of abbreviations

3D: three-dimensional; AI: artificial intelligence; BIDS: Brain Imaging Data Structure; CCF: Common Coordinate Framework; DICOM: Digital Imaging and Communications in Medicine; dMRI: diffusion magnetic resonance imaging; FAIR: Findable, Accessible, Interoperable, Reusable; GB: gigabyte; GPU: Graphics Processing Unit; ISMRM: International Society for Magnetic Resonance in Medicine; IST: International Society for Tractography; LPS: Left, Posterior, Superior; ML: machine learning; MRI: magnetic resonance imaging; PACS: Picture Archiving and Communication Systems; QC: quality control; RAS: Right, Anterior, Superior; SOP: standard operating procedures; TB: terabyte; TRX: TRactography eXchange

Ethical Approval

Not applicable.

Consent for publication

Not applicable.

Competing Interests

S.S. is an employee of ASG Superconductors; M.D. is shareholder at Imeka Solutions Inc.

Funding

W.T. is supported by the National Institute of Biomedical Imaging and Bioengineering (NIBIB) grant 2R01EB027585-04A1; K.S. is supported by grant NIH K01EB032898; M.C. is supported through NIH grants 2R01MH112847, 2R01MH120482, and 2R01MH113550; A.D.L. is supported by a Starting Grant from the European Research Council (agreement 101163214), the Galen and Hilary Weston foundation, and Stichting Hanarth Fonds; J.K. is supported by NSF Graduate Research Fellowship DGE-2140004; F.R. acknowledges support of the Natural Sciences and Engineering Research Council of Canada (NSERC); S.N.S is supported by a European Research Council Consolidator Grant (101000969); F.P. is supported by Wellcome Trust (grant no. 226486/Z/22/Z, Principal Investigator F. Pestilli), NINDS UM1NS132207, BRAIN CONNECTS: Center for Mesoscale Connectomics (Principal Investigator K. Ugurbil), and NINDS U24NS140384, BRAIN CONNECTS: The Axonal Projectome EXchange (APEX) (Principal Investigator F. Pestilli); J.Y.-M.Y acknowledges position funding support from the Royal Children's Hospital Foundation (RCHF 2022-1402 and RCHF 2025-1621), and support from The Kids' Cancer Project (TKCP) Col Reynolds Fellowship; M.D. is supported by the Université de Sherbrooke research chair in Neuroinformatics; S.H. is supported by grant NIH UM1NS132207; A.R.'s work is funded by National Institutes of Health grants MH121868, MH121867, R25MH112480, R01AG060942, and U19AG066567, and R01EB027585, as well as by National Science Foundation grants 1934292 and 2334483, and by the Chan Zuckerberg Initiative's Essential Open Source Software for Science program.

Author's Contributions

J.H.L., F.R., and A.R. developed the code that served as the basis for the plots in Panels a, c, d and e in Figure 2. All authors discussed the content, reviewed and wrote the paper.

Acknowledgements

Not applicable.

References

1. Thiebaut de Schotten M, Forkel SJ. The emergent properties of the connected brain. Science (New York, NY) 2022;378(6619):505–510. http://dx.doi.org/10.1126/science.abq2591.
2. Fields RD. White matter in learning, cognition and psychiatric disorders. Trends in neurosciences 2008;31(7):361–370. http://dx.doi.org/10.1016/j.tins.2008.04.001.
3. Passingham RE, Stephan KE, Kötter R. The anatomical basis of functional localization in the cortex. Nature reviews Neuroscience 2002;3(8):606–616. http://dx.doi.org/10.1038/nrn893.
4. Wandell BA. Clarifying Human White Matter. Annual review of neuroscience 2016 1 Apr;39:103–128. http://dx.doi.org/10.1146/annurev-neuro-070815-013815.

5. Jbabdi S, Sotiropoulos SN, Haber SN, Van Essen DC, Behrens TE. Measuring macroscopic brain connections in vivo. Nat Neurosci 2015 Nov;18(11):1546–1555.
6. Schmahmann JD, Petrides M, Pandya DN. Fiber pathways of the brain. New York, NY: Oxford University Press; 2006.
7. Axer M, Strohmer S, Gräßel D, Bücker O, Dohmen M, Reckfort J, et al. Estimating fiber orientation distribution functions in 3D-Polarized Light Imaging. Frontiers in neuroanatomy 2016 19 Apr;10:40. http://dx.doi.org/10.3389/fnana.2016.00040.
8. Lefebvre J, Delafontaine-Martel P, Pouliot P, Girouard H, Descoteaux M, Lesage F. Fully automated dual-resolution serial optical coherence tomography aimed at diffusion MRI validation in whole mouse brains. Neurophotonics 2018 Oct;5(4):045004. http://dx.doi.org/10.1117/1.NPh.5.4.045004.
9. Menzel M, Gräßel D, Rajkovic I, Zeineh MM, Georgiadis M. Using light and X-ray scattering to untangle complex neuronal orientations and validate diffusion MRI. eLife 2023 11 May;12. http://dx.doi.org/10.7554/eLife.84024.
10. Mollink J, Kleinnijenhuis M, van Cappellen van Walsum AM, Sotiropoulos SN, Cottaar M, Mirfin C, et al. Evaluating fibre orientation dispersion in white matter: Comparison of diffusion MRI, histology and polarized light imaging. NeuroImage 2017 15 Aug;157:561–574. http://dx.doi.org/10.1016/j.neuroimage.2017.06.001.
11. Schurr R, Mezer AA. The glial framework reveals white matter fiber architecture in human and primate brains. Science (New York, NY) 2021 5 Nov;374(6568):762–767. http://dx.doi.org/10.1126/science.abj7960.
12. Wang H, Lenglet C, Akkin T. Structure tensor analysis of serial optical coherence scanner images for mapping fiber orientations and tractography in the brain. Journal of biomedical optics 2015 Mar;20(3):036003. http://dx.doi.org/10.1117/1.JBO.20.3.036003.
13. Xu F, Shen Y, Ding L, Yang CY, Tan H, Wang H, et al. High-throughput mapping of a whole rhesus monkey brain at micrometer resolution. Nature biotechnology 2021 26 Dec;39(12):1521–1528. http://dx.doi.org/10.1038/s41587-021-00986-5.
14. Kjer HM, Andersson M, He Y, Pacureanu A, Daducci A, Pizzolato M, et al. Bridging the 3D geometrical organisation of white matter pathways across anatomical length scales and species. eLife 2025 28 Feb;13. http://dx.doi.org/10.7554/eLife.94917.
15. Girard G, Rafael-Patiño J, Truffet R, Aydogan DB, Adluru N, Nair VA, et al. Tractography passes the test: Results from the diffusion-simulated connectivity (disco) challenge. NeuroImage 2023 15 Aug;277(120231):120231. http://dx.doi.org/10.1016/j.neuroimage.2023.120231.
16. Maffei C, Girard G, Schilling KG, Aydogan DB, Adluru N, Zhylka A, et al. Insights from the IronTract challenge: Optimal methods for mapping brain pathways from multi-shell diffusion MRI. NeuroImage 2022 15 Aug;257(119327):119327. http://dx.doi.org/10.1016/j.neuroimage.2022.119327.
17. Maier-Hein KH, Neher PF, Houde JC, Côté MA, Garyfallidis E, Zhong J, et al. The challenge of mapping the human connectome based on diffusion tractography. Nature communications 2017 7 Nov;8(1):1349. http://dx.doi.org/10.1038/s41467-017-01285-x.
18. Schilling KG, Grussu F, Ianus A, Hansen B, Howard AFD, Barrett RLC, et al., Considerations and recommendations from the ISMRM Diffusion Study Group for preclinical diffusion MRI: Part 2 – Ex vivo imaging: added value and acquisition; 2022. http://arxiv.org/abs/2209.13371.
19. Rheault F, De Benedictis A, Daducci A, Maffei C, Tax CMW, Romascano D, et al. Tractostorm: The what, why, and how of tractography dissection reproducibility. Human brain mapping 2020;41(7):1859–1874. http://dx.doi.org/10.1002/hbm.24917.
20. Schilling KG, Rheault F, Petit L, Hansen CB, Nath V, Yeh FC, et al. Tractography dissection variability: What happens when 42 groups dissect 14 white matter bundles on the same dataset? NeuroImage 2021 1 Nov;243(118502):118502. http://dx.doi.org/10.1016/j.neuroimage.2021.118502.
21. Casey BJ, Cannonier T, Conley MI, Cohen AO, Barch DM, Heitzeg MM, et al. The Adolescent Brain Cognitive Development (ABCD) study: Imaging acquisition across 21 sites. Dev Cogn Neurosci 2018 Aug;32:43–54.
22. Cieslak M, Irfanoglu MO, Meisler SL, Salo T, Raikes A, Cook PA, et al. Diffusion MRI processing in the HEALthy Brain and child development study: Innovations and applications. bioRxiv 2025 Nov;p. 2025.11.10.687672.
23. Alexander LM, Escalera J, Ai L, Andreotti C, Febre K, Mangone A, et al. An open resource for transdiagnostic research in pediatric mental health and learning disorders. Sci Data 2017 Dec;4:170181.
24. Richie-Halford A, Cieslak M, Ai L, Caffarra S, Covitz S, Franco AR, et al. An analysis-ready and quality controlled resource for pediatric brain white-matter research. Scientific data 2022 12 Oct;9(1):616. http://dx.doi.org/10.1038/s41597-022-01695-7.
25. Alfaro-Almagro F, Jenkinson M, Bangerter NK, Andersson JLR, Griffanti L, Douaud G, et al. Image processing and Quality Control for the first 10,000 brain imaging datasets from UK Biobank. Neuroimage 2018 Feb;166:400–424.
26. Wilkinson MD, Dumontier M, Aalbersberg IJJ, Appleton G, Axton M, Baak A, et al. The FAIR Guiding Principles for scientific data management and stewardship. Scientific data 2016 15 Mar;3:160018. http://dx.doi.org/10.1038/sdata.2016.18.
27. Jelescu IO, Grussu F, Ianus A, Hansen B, Barrett RLC, Aggarwal M, et al., Considerations and Recommendations from the ISMRM Diffusion Study Group for preclinical diffusion MRI: Part 1 – In vivo small-animal imaging; 2022. http://arxiv.org/abs/2209.12994.
28. Schilling KG, Howard AFD, Grussu F, Ianus A, Hansen B, Barrett RLC, et al., Considerations and recommendations from the ISMRM Diffusion Study Group for preclinical diffusion MRI: Part 3 – Ex vivo imaging: data processing, comparisons with microscopy, and tractography; 2024. http://arxiv.org/abs/2411.05021.
29. Kruper J, Yeatman JD, Richie-Halford A, Bloom D, Grotheer M, Caffarra S, et al. Evaluating the Reliability of Human Brain White Matter Tractometry. Apert Neuro 2021 Nov;1(1).
30. Mustra M, Delac K, Grgic M. Overview of the DICOM standard. In: 2008 50th International Symposium ELMAR, vol. 1 Zadar, Croatia: IEEE; 2008. p. 39–44. https://ieeexplore.ieee.org/abstract/document/4747434/.
31. Gorgolewski KJ, Auer T, Calhoun VD, Cameron Craddock R, Das S, Duff EP, et al. The brain imaging data structure, a format for organizing and describing outputs of neuroimaging experiments. Scientific data 2016 21 Jun;3(1):1–9.
32. Gholam J, Szczepankiewicz F, Tax CMW, Mueller L, Kopanoglu E, Nilsson M, et al., aDWI-BIDS: an extension to the brain imaging data structure for advanced diffusion weighted imaging; 2021. http://arxiv.org/abs/2103.14485.
33. Pestilli F, Poldrack R, Rokem A, Satterthwaite T, Feingold F, Duff E, et al. A community-driven development of the brain imaging data standard (BIDS) to describe macroscopic brain connections 2021;.
34. Cai LY, Yang Q, Kanakaraj P, Nath V, Newton AT, Edmonson HA, et al. MASiVar: Multisite, multiscanner, and multisubject acquisitions for studying variability in diffusion weighted MRI. Magnetic resonance in medicine 2021 1 Dec;86(6):3304–3320. http://dx.doi.org/10.1002/mrm.28926.
35. Gajwani M, Oldham S, Pang JC, Arnatkevičiūtė A, Tiego J,

Bellgrove MA, et al. Can hubs of the human connectome be identified consistently with diffusion MRI? Network neuroscience (Cambridge, Mass) 2023 22 Dec;7(4):1326–1350. http://dx.doi.org/10.1162/netn_a_00324.

36. Schilling KG, Tax CMW, Rheault F, Hansen C, Yang Q, Yeh FC, et al. Fiber tractography bundle segmentation depends on scanner effects, vendor effects, acquisition resolution, diffusion sampling scheme, diffusion sensitization, and bundle segmentation workflow. NeuroImage 2021 15 Nov;242:118451. http://dx.doi.org/10.1016/j.neuroimage.2021.118451.

37. Tong Q, He H, Gong T, Li C, Liang P, Qian T, et al. Reproducibility of multi-shell diffusion tractography on traveling subjects: A multicenter study prospective. Magnetic resonance imaging 2019 1 Jun;59:1–9. http://dx.doi.org/10.1016/j.mri.2019.02.011.

38. Warrington S, Torchi A, Mougin O, Campbell J, Ntata A, Craig M, et al. A multi-site, multi-modal travelling-heads resource for brain MRI harmonisation. Sci Data 2025 Apr;12(1):609.

39. Pinto MS, Paolella R, Billiet T, Van Dyck P, Guns PJ, Jeurissen B, et al. Harmonization of Brain Diffusion MRI: Concepts and Methods. Front Neurosci 2020 May;14:396.

40. Moyer D, Ver Steeg G, Tax CMW, Thompson PM. Scanner invariant representations for diffusion MRI harmonization. Magnetic resonance in medicine 2020 1 Oct;84(4):2174–2189. http://dx.doi.org/10.1002/mrm.28243.

41. Ning L, Bonet-Carne E, Grussu F, Sepehrband F, Kaden E, Veraart J, et al. Muti-shell diffusion MRI harmonisation and enhancement challenge (MUSHAC): Progress and results. In: Computational Diffusion MRI Mathematics and visualization, Cham: Springer International Publishing; 2019.p. 217–224.

42. Tax CM, Grussu F, Kaden E, Ning L, Rudrapatna U, John Evans C, et al. Cross-scanner and cross-protocol diffusion MRI data harmonisation: A benchmark database and evaluation of algorithms. NeuroImage 2019 15 Jul;195:285–299. http://dx.doi.org/10.1016/j.neuroimage.2019.01.077.

43. Warrington S, Ntata A, Mougin O, Campbell J, Torchi A, Craig M, et al. A resource for development and comparison of multimodal brain 3T MRI harmonisation approaches. Imaging Neurosci (Camb) 2023 Nov;1:1–27.

44. Magdoom KN, Avram AV, Sarlls JE, Dario G, Basser PJ. A novel framework for in-vivo diffusion tensor distribution MRI of the human brain. NeuroImage 2023 05;271:120003.

45. Yang Q, Shomal-Zadeh F, Gholipour A, Harmonization in Magnetic Resonance Imaging: A Survey of Acquisition, Image-level, and Feature-level Methods; 2025. https://arxiv.org/abs/2507.16962.

46. Esteban O, Birman D, Schaer M, Koyejo OO, Poldrack RA, Gorgolewski KJ. MRIQC: Advancing the automatic prediction of image quality in MRI from unseen sites. PloS one 2017 25 Sep;12(9):e0184661. http://dx.doi.org/10.1371/journal.pone.0184661.

47. Bastiani M, Cottaar M, Fitzgibbon SP, Suri S, Alfaro-Almagro F, Sotiropoulos SN, et al. Automated quality control for within and between studies diffusion MRI data using a non-parametric framework for movement and distortion correction. NeuroImage 2019 1 Jan;184:801–812. http://dx.doi.org/10.1016/j.neuroimage.2018.09.073.

48. Hagen MP, Provins C, MacNicol E, Li J, Gomez T, Garcia M, et al., Quality assessment and control of unprocessed anatomical, functional, and diffusion MRI of the human brain using MRIQC; 2024.

49. Cieslak M, Cook PA, He X, Yeh FC, Dhollander T, Adebimpe A, et al. QSIPrep: an integrative platform for preprocessing and reconstructing diffusion MRI data. Nature methods 2021;18(7):775–778. http://dx.doi.org/10.1038/s41592-021-01185-5.

50. Cirstian R, Forde NJ, Andersson JLR, Sotiropoulos SN, Beckmann CF, Marquand AF. Objective QC for diffusion MRI data: Artefact detection using normative modelling. Imaging Neuroscience 2024 26 Apr;2:1–14. https://dx.doi.org/10.1162/imag_a_00144.

51. Laamoumi M, Hendriks T, Chamberland M. A taxonomic guide to diffusion MRI tractography visualization tools. NMR in Biomedicine 2025 7 Jan;38(1):e5267. http://dx.doi.org/10.1002/nbm.5267.

52. Smith RE, Tournier JD, Calamante F, Connelly A. SIFT: Spherical-deconvolution informed filtering of tractograms. NeuroImage 2013 15 Feb;67:298–312. http://dx.doi.org/10.1016/j.neuroimage.2012.11.049.

53. Smith RE, Tournier JD, Calamante F, Connelly A. SIFT2: Enabling dense quantitative assessment of brain white matter connectivity using streamlines tractography. NeuroImage 2015 1 Oct;119:338–351. http://dx.doi.org/10.1016/j.neuroimage.2015.06.092.

54. Daducci A, Palù AD, Lemkaddem A, Thiran J. COMMIT: Convex optimization modeling for microstructure informed tractography. IEEE transactions on medical imaging 2015 1 Jan;34:246–257. http://dx.doi.org/10.1109/TMI.2014.2352414.

55. Schiavi S, Ocampo-Pineda M, Barakovic M, Petit L, Descoteaux M, Thiran JP, et al. A new method for accurate in vivo mapping of human brain connections using microstructural and anatomical information. Science Advances 2020 31 Jul;6(31). http://dx.doi.org/10.1126/sciadv.aba8245.

56. Caiafa CF, Pestilli F. Multidimensional encoding of brain connectomes. Scientific reports 2017 13 Sep;7(1):11491. http://dx.doi.org/10.1038/s41598-017-09250-w.

57. Pestilli F, Yeatman JD, Rokem A, Kay KN, Wandell BA. Evaluation and statistical inference for human connectomes. Nature methods 2014 Oct;11(10):1058–1063. http://dx.doi.org/10.1038/nmeth.3098.

58. Astolfi P, Verhagen R, Petit L, Olivetti E, Sarubbo S, Masci J, et al. Supervised tractogram filtering using Geometric Deep Learning. Medical image analysis 2023 1 Dec;90(102893):102893. http://dx.doi.org/10.1016/j.media.2023.102893.

59. Legarreta JH, Petit L, Rheault F, Theaud G, Lemaire C, Descoteaux M, et al. Filtering in Tractography using Autoencoders (FINTA). Medical image analysis 2021 7 Jun;p. 102126. http://dx.doi.org/10.1016/j.media.2021.102126.

60. Sarwar T, Ramamohanarao K, Zalesky A. A critical review of connectome validation studies. NMR in biomedicine 2021 1 Dec;34(12):e4605. http://dx.doi.org/10.1002/nbm.4605.

61. Sarwar T, Ramamohanarao K, Daducci A, Schiavi S, Smith RE, Zalesky A. Evaluation of tractogram filtering methods using human-like connectome phantoms. NeuroImage 2023 1 Nov;281(120376):120376. http://dx.doi.org/10.1016/j.neuroimage.2023.120376.

62. Zalesky A, Sarwar T, Ramamohanarao K. A cautionary note on the use of SIFT in pathological connectomes. Magnetic resonance in medicine 2020 1 Mar;83(3):791–794. http://dx.doi.org/10.1002/mrm.28037.

63. Fillard P, Descoteaux M, Goh A, Gouttard S, Jeurissen B, Malcolm J, et al. Quantitative evaluation of 10 tractography algorithms on a realistic diffusion MR phantom. NeuroImage 2011 05;56(1):220–234.

64. Girard G, no JRP, Truffet R, Aydogan DB, Adluru N, Nair VA, et al. Tractography passes the test: Results from the diffusion-simulated connectivity (disco) challenge. NeuroImage 2023 08;277:120231. https://www.sciencedirect.com/science/article/pii/S1053811923003828.

65. Glen DR, Taylor PA, Buchsbaum BR, Cox RW, Reynolds RC. Beware (surprisingly common) left-right flips in your MRI data: An efficient and robust method to check MRI dataset consistency using AFNI. Frontiers in neuroinformatics 2020 25 May;14:18. http://dx.doi.org/10.3389/fninf.2020.00018.

66. Lanciego JL, Wouterlood FG. Neuroanatomical tract-tracing techniques that did go viral. Brain Struct Funct 2020 May;225(4):1193–1224.
67. Milham MP, Ai L, Koo B, Xu T, Amiez C, Balezeau F, et al. An open resource for non-human primate imaging. Neuron 2018 10 Oct;100(1):61–74.e2. http://dx.doi.org/10.1016/j.neuron.2018.08.039.
68. Hata J, Nakae K, Tsukada H, Woodward A, Haga Y, Iida M, et al. Multi-modal brain magnetic resonance imaging database covering marmosets with a wide age range. Scientific data 2023 27 Apr;10(1):221. http://dx.doi.org/10.1038/s41597-023-02121-2.
69. Kremer JR, Mastronarde DN, McIntosh JR. Computer visualization of three-dimensional image data using IMOD. Journal of structural biology 1996 1 Jan;116(1):71–76. http://dx.doi.org/10.1006/jsbi.1996.0013.
70. Paxinos G, Petrides M, Evrard HC. The rhesus monkey brain in stereotaxic coordinates. 4 ed. San Diego, CA: Academic Press; 2023.
71. Paxinos G, Huang XF, Toga AW. The Rhesus Monkey Brain in Stereotaxic Coordinates. 1 ed. San Diego, CA: Academic Press; 1999. ISBN: 9780128158524.
72. Desikan RS, Ségonne F, Fischl B, Quinn BT, Dickerson BC, Blacker D, et al. An automated labeling system for subdividing the human cerebral cortex on MRI scans into gyral based regions of interest. NeuroImage 2006 1 Jul;31(3):968–980. http://dx.doi.org/10.1016/j.neuroimage.2006.01.021.
73. Mai JK, Majtanik M. Myeloarchitectonic maps of the human cerebral cortex registered to surface and sections of a standard atlas brain. Translational neuroscience 2023 1 Jan;14(1):20220325. http://dx.doi.org/10.1515/tnsci-2022-0325.
74. Petrides M, Tomaiuolo F, Yeterian EH, Pandya DN. The prefrontal cortex: comparative architectonic organization in the human and the macaque monkey brains. Cortex; a journal devoted to the study of the nervous system and behavior 2012 1 Jan;48(1):46–57. http://dx.doi.org/10.1016/j.cortex.2011.07.002.
75. Lu Y, Cui Y, Cao L, Dong Z, Cheng L, Wu W, et al. Macaque Brainnetome Atlas: A multifaceted brain map with parcellation, connection, and histology. Science Bulletin 2024;69(14):2241–2259. 07.
76. Zhang Z, Zhu T, Guo J, Ouyang M, Sousa A, Levine J, et al. Common coordinate framework of developmental macaque brain from birth to early childhood based on ultra-high-resolution diffusion MRI. In: 34th Annual Conference & Exhibition of the International Society for Magnetic Resonance in Medicine (ISMRM) Honolulu, HI USA: International Society for Magnetic Resonance in Medicine (ISMRM); 2025. Program #3654.
77. Warrington S, Thompson E, Bastiani M, Dubois J, Baxter L, Slater R, et al. Concurrent mapping of brain ontogeny and phylogeny within a common space: Standardized tractography and applications. Science advances 2022 21 Oct;8(42):eabq2022. http://dx.doi.org/10.1126/sciadv.abq2022.
78. Coizet V, Heilbronner SR, Carcenac C, Mailly P, Lehman JF, Savasta M, et al. Organization of the anterior limb of the internal capsule in the rat. The Journal of neuroscience: the official journal of the Society for Neuroscience 2017 8 Mar;37(10):2539–2554. http://dx.doi.org/10.1523/JNEUROSCI.3304-16.2017.
79. Martin RF, Bowden DM. A Stereotaxic Template Atlas of the Macaque Brain for Digital Imaging and Quantitative Neuroanatomy. NeuroImage 1996 10;4(2):119–150.
80. Lein ES, Hawrylycz MJ, Ao N, Ayres M, Bensinger A, Bernard A, et al. Genome-wide atlas of gene expression in the adult mouse brain. Nature 2007 11 Jan;445(7124):168–176. http://dx.doi.org/10.1038/nature05453.
81. Wang Q, Ding SL, Li Y, Royall J, Feng D, Lesnar P, et al. The Allen Mouse Brain Common Coordinate Framework: A 3D reference atlas. Cell 2020 14 May;181(4):936–953.e20. http://dx.doi.org/10.1016/j.cell.2020.04.007.
82. Zhang Y, Song T, Yang CY, Shen Y, Yang Y, Hu X, et al. Whole-brain reconstruction of fiber tracts based on cytoarchitectonic organization. Nat Methods 2025 Dec;22(12):2639–2648.
83. Hayot-Sasson V, Glatard T, Rokem A. The benefits of prefetching for large-scale cloud-based neuroimaging analysis workflows. In: 2021 IEEE Workshop on Workflows in Support of Large-Scale Science (WORKS); 2021. p. 42–49.
84. Trinkle S, Foxley S, Kasthuri N, La Rivière P. Synchrotron X-ray micro-CT as a validation dataset for diffusion MRI in whole mouse brain. Magnetic resonance in medicine 2021 1 Aug;86(2):1067–1076. http://dx.doi.org/10.1002/mrm.28776.
85. Foxley S, Sampathkumar V, De Andrade V, Trinkle S, Sorokina A, Norwood K, et al. Multi-modal imaging of a single mouse brain over five orders of magnitude of resolution. NeuroImage 2021 1 Sep;238(118250):118250. http://dx.doi.org/10.1016/j.neuroimage.2021.118250.
86. Shapson-Coe A, Januszewski M, Berger DR, Pope A, Wu Y, Blakely T, et al. A petavoxel fragment of human cerebral cortex reconstructed at nanoscale resolution. Science (New York, NY) 2024 10 May;384(6696):eadk4858. http://dx.doi.org/10.1126/science.adk4858.
87. Rheault F, Houde JC, Descoteaux M. Visualization, interaction and tractometry: Dealing with millions of streamlines from diffusion MRI tractography. Frontiers in neuroinformatics 2017 26 Jun;11:42. http://dx.doi.org/10.3389/fninf.2017.00042.
88. Gabusi I, Battocchio M, Bosticardo S, Schiavi S, Daducci A. Blurred streamlines: A novel representation to reduce redundancy in tractography. Med Image Anal 2024 Apr;93(103101):103101.
89. Presseau C, Jodoin PM, Houde JC, Descoteaux M. A new compression format for fiber tracking datasets. Neuroimage 2015 Sep;109(102893):160018.
90. Kruper J, Hagen MP, Rheault F, Crane I, Gilmore A, Narayan M, et al. Tractometry of the Human Connectome Project: resources and insights. Frontiers in neuroscience 2024 12 Jun;18:1389680. http://dx.doi.org/10.3389/fnins.2024.1389680.
91. Kraaijeveld B, Vilanova A, Chamberland M, Improved Shading and Performance using Density Volumes in Interactive Tractography Visualization. Kyoto, Japan; 2025. https://www.ismrm.org/workshops/2025/Diffusion40/, poster #181. ISMRM Workshop on 40 Years of Diffusion: Past, Present & Future Perspectives.
92. Rheault F, Hayot-Sasson V, Smith RE, Rorden C, Tournier JD, Garyfallidis E, et al. TRX: A Community-Oriented Tractography File Format. In: 28th Annual Meeting of The Organization for Human Brain Mapping (OHBM) Glasgow, Scotland; 2022. .
93. Legeay S. Mesoscopic mapping of the human structural connectome using high-performance global tractography. PhD thesis, Université Paris-Saclay; 2025.
94. Kruper J, Bisson M, Romero J, Fatica M, Rokem A. GPU-accelerated Diffusion MRI Tractography in DIPY. In: International Society for Magnetic Resonance in Medicine; 2025. https://archive.ismrm.org/2025/4785_bKkDvtQEA.html.
95. Hernandez-Fernandez M, Reguly I, Jbabdi S, Giles M, Smith S, Sotiropoulos SN. Using GPUs to accelerate computational diffusion MRI: From microstructure estimation to tractography and connectomes. Neuroimage 2019 Mar;188:598–615.
96. Abernathey RP, Augspurger T, Banihirwe A, Blackmon-Luca CC, Crone TJ, Gentemann CL, et al. Cloud-Native Repositories for Big Scientific Data. Computing in Science Engineering 2021 Mar;23(2):26–35.

97. Poulin P, Jörgens D, Jodoin PM, Descoteaux M. Tractography and machine learning: Current state and open challenges. Magnetic Resonance Imaging 2019 12;64:37–48. Artificial Intelligence in MRI.
98. Karimi D, Warfield SK. Diffusion MRI with machine learning. Imaging Neuroscience 2024 11;2:imag–2–00353.
99. Neher P, Poulin P, Jörgens D, Reisert M, Benou I, Maier-Hein K. Chapter 17 - Machine learning in tractography. In: Dell'Acqua F, Descoteaux M, Leemans A, editors. Handbook of Diffusion MR Tractography - Imaging Methods, Biophysical Models, Algorithms and Applications Academic Press; 2025.p. 315–345.
100. Côté MA, Girard G, Boré A, Garyfallidis E, Houde JC, Descoteaux M. Tractometer: Towards validation of tractography pipelines. Medical Image Analysis 2013 10;17(7):844–857. Special Issue on the 2012 Conference on Medical Image Computing and Computer Assisted Intervention.
101. Bai J, Lu F, Zhang K, et al., ONNX: Open Neural Network Exchange. GitHub; 2019. https://github.com/onnx/onnx.
102. Bizzi A, Yang JYM, Aliaga-Arias J, Dell'Acqua F, Lavrador JP, Vergani F. Chapter 31 - Neurosurgical applications of clinical tractography. In: Dell'Acqua F, Descoteaux M, Leemans A, editors. Handbook of Diffusion MR Tractography : Academic Press; 2025.p. 631–652.
103. Aylmore H, Young F, Aquilina K, Clark CA, Clayden JD. The use of intraoperative tractography in brain tumor and epilepsy surgery: a systematic review and meta-analysis. Frontiers in Neuroimaging 2025 06;4.
104. Kamagata K, Andica C, Uchida W, Takabayashi K, Saito Y, Lukies M, et al. Advancements in Diffusion MRI Tractography for Neurosurgery. Investigative Radiology 2024;59(1). https://journals.lww.com/investigativeradiology/fulltext/2024/01000/advancements_in_diffusion_mri_tractography_for.2.aspx.
105. Costabile JD, Alaswad E, D'Souza S, Thompson JA, Ormond DR. Current applications of diffusion tensor imaging and tractography in intracranial tumor resection. Frontiers in oncology 2019 29 May;9:426. http://dx.doi.org/10.3389/fonc.2019.00426.
106. Vanderweyen DC, Theaud G, Sidhu J, Rheault F, Sarubbo S, Descoteaux M, et al. The role of diffusion tractography in refining glial tumor resection. Brain structure & function 2020 May;225(4):1413–1436. http://dx.doi.org/10.1007/s00429-020-02056-z.
107. Kwon HG, Hong JH, Hong CP, Lee DH, Ahn SH, Jang SH. Dentatorubrothalamic tract in human brain: diffusion tensor tractography study. Neuroradiology 2011 Oct;53(10):787–791. http://dx.doi.org/10.1007/s00234-011-0878-7.
108. Lehman VT, Lee KH, Klassen BT, Blezek DJ, Goyal A, Shah BR, et al. MRI and tractography techniques to localize the ventral intermediate nucleus and dentatorubrothalamic tract for deep brain stimulation and MR-guided focused ultrasound: a narrative review and update. Neurosurgical focus 2020 1 Jul;49(1):E8. http://dx.doi.org/10.3171/2020.4.FOCUS20170.
109. Nowacki A, Debove I, Rossi F, Schlaeppi JA, Petermann K, Wiest R, et al. Targeting the posterior subthalamic area for essential tremor: proposal for MRI-based anatomical landmarks. Journal of neurosurgery 2019 Sep;131(3):820–827. http://dx.doi.org/10.3171/2018.4.JNS18373.
110. Jacquesson T, Frindel C, Kocevar G, Berhouma M, Jouanneau E, Attyé A, et al. Overcoming Challenges of Cranial Nerve Tractography: A Targeted Review. Neurosurgery 2019 02;84(2).
111. de Faria O Jr, Pivonkova H, Varga B, Timmler S, Evans KA, Káradóttir RT. Periods of synchronized myelin changes shape brain function and plasticity. Nat Neurosci 2021 Nov;24(11):1508–1521.
112. Knickmeyer RC, Gouttard S, Kang C, Evans D, Wilber K, Smith JK, et al. A structural MRI study of human brain development from birth to 2 years. J Neurosci 2008 Nov;28(47):12176–12182.
113. Shi F, Yap PT, Wu G, Jia H, Gilmore JH, Lin W, et al. Infant brain atlases from neonates to 1- and 2-year-olds. PLoS One 2011 Apr;6(4):e18746.
114. Sanchez CE, Richards JE, Almli CR. Neurodevelopmental MRI brain templates for children from 2 weeks to 4 years of age. Developmental psychobiology 2012;54(1):77–91.
115. Calixto C, Dorigatti Soldatelli M, Jaimes C, Pierotich L, Warfield SK, Gholipour A, et al. A detailed spatiotemporal atlas of the white matter tracts for the fetal brain. Proceedings of the National Academy of Sciences 2025 7 Jan;122(1):e2410341121. http://dx.doi.org/10.1073/pnas.2410341121.
116. Gilmore JH, Knickmeyer RC, Gao W. Imaging structural and functional brain development in early childhood. Nat Rev Neurosci 2018 Feb;19(3):123–137.
117. Grotheer M, Rosenke M, Wu H, Kular H, Querdasi FR, Natu VS, et al. White matter myelination during early infancy is linked to spatial gradients and myelin content at birth. Nature communications 2022;13(1):1–12.
118. Guerrero JM, Adluru N, Bendlin BB, Goldsmith HH, Schaefer SM, Davidson RJ, et al. Optimizing the intrinsic parallel diffusivity in NODDI: An extensive empirical evaluation. PLoS One 2019 Sep;14(9):e0217118.
119. Chang KH, Burke L, LaPiana N, Howlett B, Hunt D, Dezelar M, et al. Free water elimination tractometry for aging brains. bioRxiv 2024 Nov;p. 2024.11.10.622861.
120. Hollmann S, Frohme M, Endrullat C, Kremer A, D'Elia D, Regierer B, et al. Ten simple rules on how to write a standard operating procedure. PLoS computational biology 2020 3 Sep;16(9):e1008095. http://dx.doi.org/10.1371/journal.pcbi.1008095.
121. Shamir I, Assaf Y. Tutorial: a guide to diffusion MRI and structural connectomics. Nat Protoc 2025 Feb;20(2):317–335.
122. Tahedl M, Tournier JD, Smith RE. Structural connectome construction using constrained spherical deconvolution in multi-shell diffusion-weighted magnetic resonance imaging. Nat Protoc 2025 Feb;p. 1–33.
123. Layton KJ, Kroboth S, Jia F, Littin S, Yu H, Leupold J, et al. Pulseq: A rapid and hardware-independent pulse sequence prototyping framework. Magn Reson Med 2017 Apr;77(4):1544–1552.
124. Liu Q, Ning L, Shaik IA, Liao C, Gagoski B, Bilgic B, et al. Reduced cross-scanner variability using vendor-agnostic sequences for single-shell diffusion MRI. Magn Reson Med 2024 Jul;92(1):246–256.
125. Barker M, Chue Hong NP, Katz DS, Lamprecht AL, Martinez-Ortiz C, Psomopoulos F, et al. Introducing the FAIR Principles for research software. Sci Data 2022 Oct;9(1):622.
126. Ambatipudi S, Byna S. A comparison of HDF5, zarr, and netCDF4 in performing common I/O operations. arXiv [csDC] 2022 Jul;.
127. Vohra D. Apache Parquet. In: Practical Hadoop Ecosystem Berkeley, CA: Apress; 2016.p. 325–335.
128. Lopez-Gomez J, Blomer J. RNTuple performance: Status and Outlook. arXiv [physicsdata-an] 2022 Apr;.
129. Kruper J, Richie-Halford A, Qiao J, Gilmore A, Chang K, Grotheer M, et al. A software ecosystem for brain tractometry processing, analysis, and insight. PLoS Comput Biol 2025 Aug;21(8):e1013323.
130. Beare R, Alexander B, Warren A, Kean M, Seal M, Wray A, et al. Karawun: a software package for assisting evaluation of advances in multimodal imaging for neurosurgical planning and intraoperative neuronavigation. Int J Comput Assist Radiol Surg 2023 Jan;18(1):171–179.

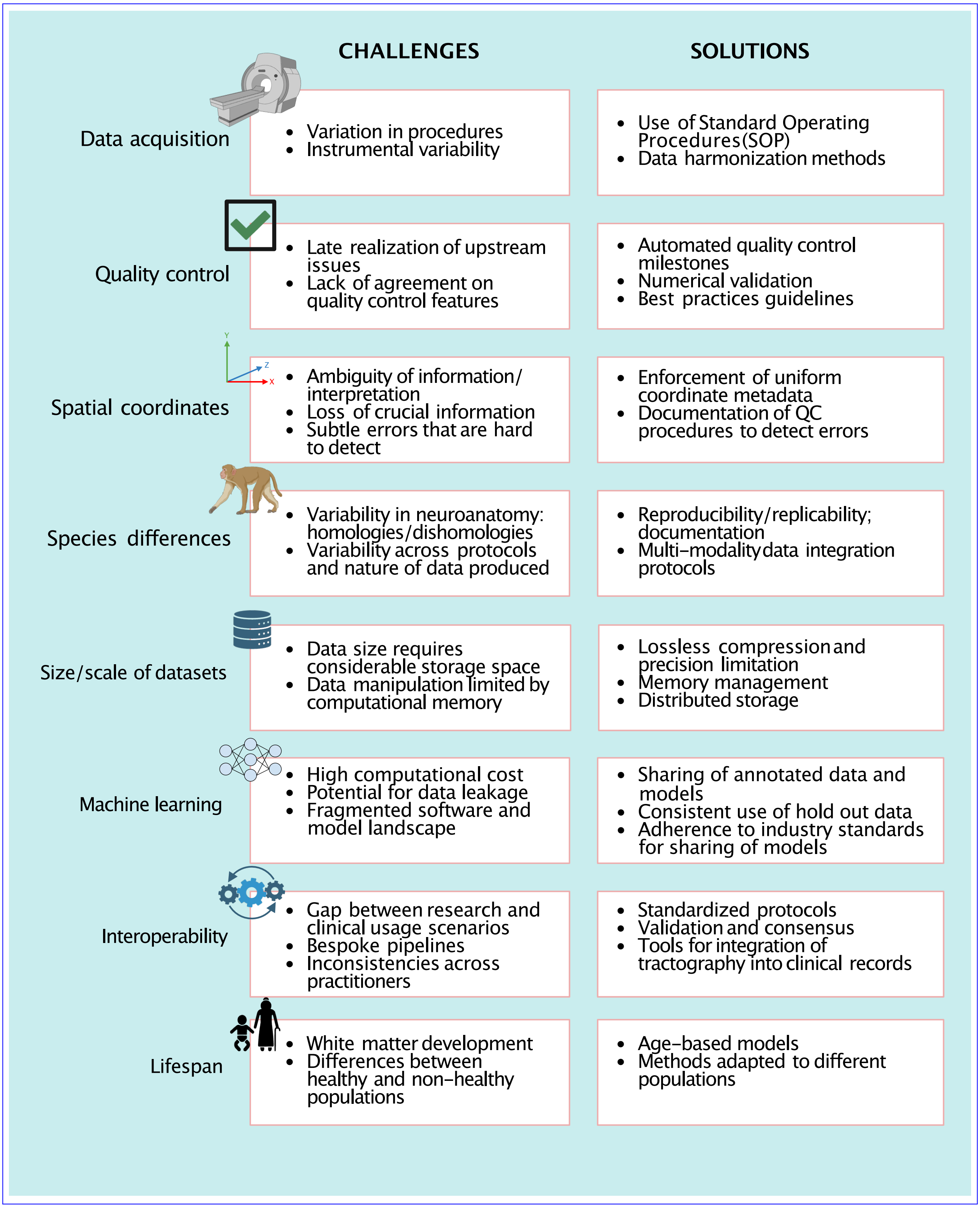

Figure 1. Summary of main challenges and suggested standardization solutions towards reliable, reproducible, and robust tractography.

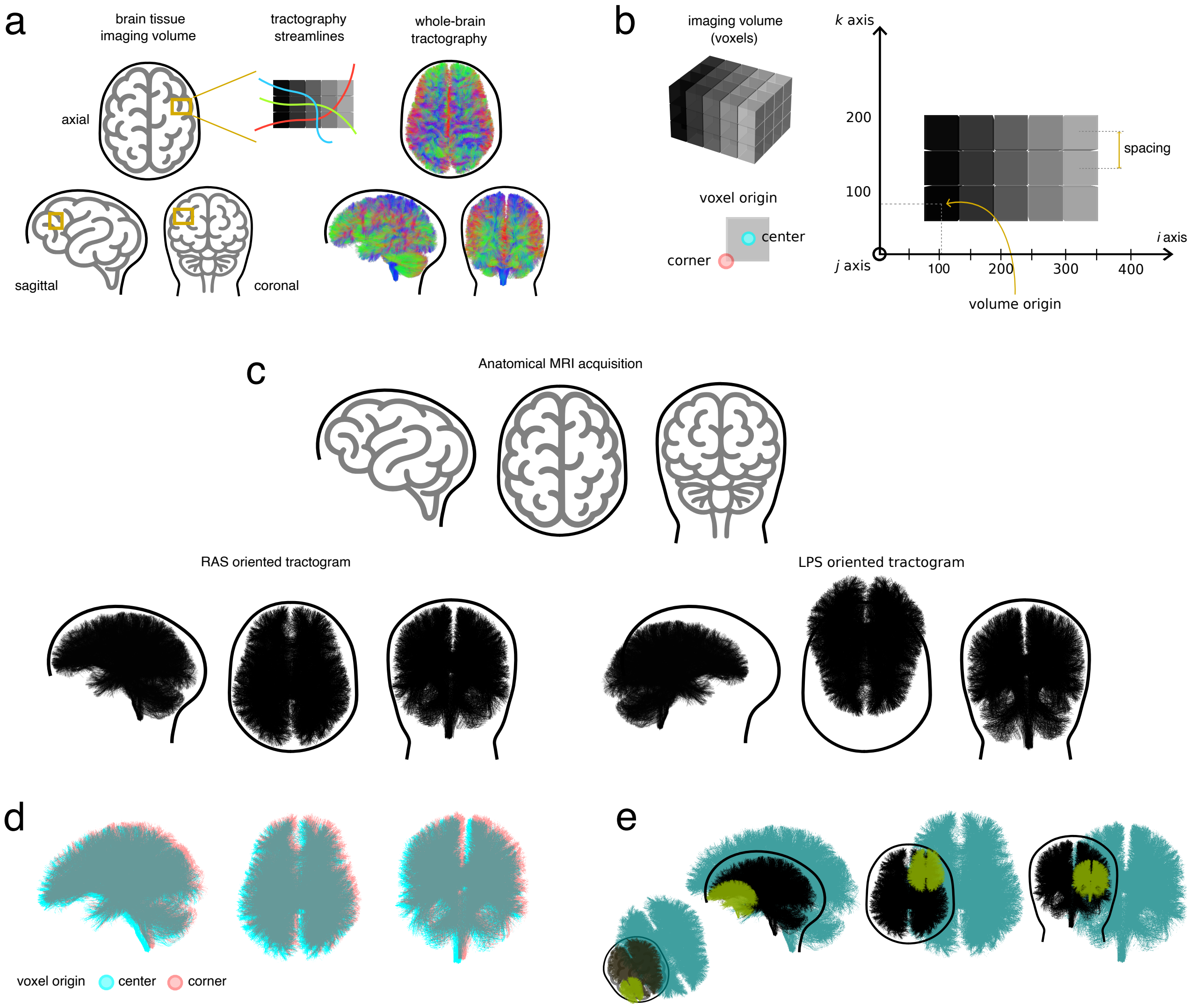

Figure 2. Tractography data representation issues. Panel a: schematic view of the process of generating tractography streamline data from an imaged brain tissue volume; Panel b: miscellaneous concepts related to tractography data; Panel c: illustration of the spatial mismatch between a tractography file natively serialized following the *LPS+* convention but the structural data (e.g., a T1-weighted MRI acquisition) being arranged following the *RAS+* convention; Panel d: effect of the voxel origin convention visualized as a half-voxel shift between the *corner* and *center* conventions on a whole-brain tractogram; Panel e: illustration of tractography data representation issues arising from mismatches between the spatial coordinate transformation matrix employed when reconstructing streamlines *vs.* the one used when serializing the tractography data, or from storing the data in voxel space *vs.* real-world space coordinates, or from inadvertently applying spatial transformations multiple times. In all cases, the tractogram in black is at the correct location, and the tractograms in green and turquoise do not lie at the correct anatomical location.

Personal Cover
Click here to access/download;Personal Cover;2025-Cover-GigaScience-IST-Standardization-Proposal.pdf

Ariel Rokem, PhD
Department of Psychology
Guthrie Hall 119 A
Seattle, WA 98105
Phone: +1 (510) 387-6264
E-mail: arokem@uw.edu
https://neuroinformatics.uw.edu

September 4th , 2025

Dear *GigaScience* Editors,

We are pleased to submit a proposal for a potential "Review" contribution to *GigaScience* titled "What needs to be standardized for reliable, reproducible, and robust tractography?"

Tractography is a cornerstone method for neuroanatomical studies, including many Open/Big Data initiatives, yet it remains plagued by ambiguity –ranging from fragmentation in spatial reference information, to the absence of unified analysis protocols. To address these challenges, the International Society for Tractography (https://tractography.io/) convened a panel of experts for a series of discussions focused on tractography standardization. This has resulted in a detailed white paper on the standards and policy related to these questions that we are now pleased to submit for your consideration.

The set of authors for this potential contribution is authoritative and very diverse, and is composed of 17 recognized leaders in the field from 21 different institutions in 6 different countries. The work is timely given the increasing reliance on tractography data for both clinical applications and large-scale neuroscience initiatives. The authors of the proposed contribution cover a broad range of expertise, including researchers that use many different methods and model systems in basic research, as well as clinicians who use tractography in their work.

We believe that this white paper could serve as the basis for a "Review" paper in *GigaScience*, as it provides a forward-looking and critical assessment of the scientific and technical challenges and opportunities that currently exist for standardization in the highly interdisciplinary field of tractography. In this work, we have also formulated a series of steps towards improvements of the state of the field in the future.

We confirm that all authors have approved the manuscript for submission, and that potential competing interests have been disclosed in the manuscript. The content of the manuscript has not been published, or submitted for publication elsewhere.

Thank you for your consideration of our proposal.

Sincerely,

Ariel Rokem, PhD
Research Associate Professor, Department of Psychology
Adjunct Associate Professor, Paul G. Allen School of Computer Science & Engineering
Senior Data Scientist, eScience Institute
University of Washington
