# Supplementary material for: What needs to be standardized for reliable, reproducible, and robust tractography?
Source: Gigascience. 2026 Mar 25;15:giag034. doi: 10.1093/gigascience/giag034 (PMC13137869; doi:10.1093/gigascience/giag034)
Supplement: giag034_GIGA-D-25-00365_Revision_2 [file giag034_giga-d-25-00365_revision_2.pdf]

GigaScience

What needs to be standardized for reliable, reproducible, and robust tractography?

--Manuscript Draft--

**Manuscript Number:**	GIGA-D-25-00365R2	
**Full Title:**	What needs to be standardized for reliable, reproducible, and robust tractography?	
**Article Type:**	Review	
**Funding Information:**	National Institute of Biomedical Imaging and Bioengineering (2R01EB027585-04A1)	PhD Wei Tang
	National Institute of Biomedical Imaging and Bioengineering (K01EB032898)	PhD Kurt Schilling
	National Institute of Mental Health (2R01MH112847)	PhD Matthew Cieslak
	National Institute of Mental Health (2R01MH120482)	PhD Matthew Cieslak
	National Institute of Mental Health (2R01MH113550)	PhD Matthew Cieslak
	HORIZON EUROPE European Research Council (101163214)	PhD Alberto De Luca
	Galen and Hilary Weston foundation	PhD Alberto De Luca
	Stichting Hanarth Fonds	PhD Alberto De Luca
	Division of Graduate Education (DGE-2140004)	MSc John Kruper
	Natural Sciences and Engineering Research Council of Canada	PhD Francois Rheault
	HORIZON EUROPE European Research Council (101000969)	Prof Stamatios N. Sotiropoulos
	Wellcome Trust (226486/Z/22/Z)	PhD Franco Pestilli
	National Institute of Neurological Disorders and Stroke (UM1NS132207)	PhD Franco Pestilli<br>PhD Sarah Heilbronner
	National Institute of Neurological Disorders and Stroke (U24NS140384)	PhD Franco Pestilli
	Royal Children's Hospital Foundation (RCHF 2022-1402, RCHF 2025-1621)	MD, PhD Joseph Yuan-Mou Yang
	Kids' Cancer Project	MD, PhD Joseph Yuan-Mou Yang
	Université de Sherbrooke (Research Chair in Neuroinformatics)	Prof Maxime Descoteaux
	National Institute of Mental Health (MH121868)	PhD Ariel Rokem
	National Institute of Mental Health (MH121867)	PhD Ariel Rokem
	National Institute of Mental Health (R25MH112480)	PhD Ariel Rokem
	National Institute on Aging (R01AG060942)	PhD Ariel Rokem
	National Institute on Aging (U19AG066567)	PhD Ariel Rokem
	National Institute of Biomedical Imaging and Bioengineering (R01EB027585)	PhD Ariel Rokem
	National Science Foundation (1934292)	PhD Ariel Rokem
	National Science Foundation	PhD Ariel Rokem

Powered by Editorial Manager® and ProduXion Manager® from Aries Systems Corporation

	(2334483)	
	Chan Zuckerberg Initiative	PhD Ariel Rokem

Abstract: Tractography is a key component of efforts to map brain connectivity. As a rapidly-evolving field of neuroscience, current tractography methods are diverse, often varying across research laboratories and different software pipelines. Therefore, it suffers from a lack of standardization leading to inconsistencies in results, which can limit reproducibility, and affect the robustness needed for research and clinical applications of these methods. Variability in data acquisition procedures, inconsistencies in spatial referencing schemes and implementations, and anatomical heterogeneity —at the individual level, across the lifespan, and across species— hinders comparative analyses. Additionally, the lack of consensus on best practices complicates the development of robust automated quality control pipelines and limits the clinical translation of tractography-based procedures. Establishing standardized protocols for acquisition, preprocessing, and tractography reconstruction are critical towards enabling reliable tract-specific analyses, facilitating cross-study harmonization, and supporting replicable large-scale population studies. The present article provides an overview of the current challenges in tractography standardization and identifies the key aspects that require standardization for reliable, reproducible, and robust tractography.

**Corresponding Author:**	Jon Haitz Legarreta<br>Brigham and Women's Hospital<br>Somerville, Massachusetts UNITED STATES
**Corresponding Author Secondary Information:**	
**Corresponding Author's Institution:**	Brigham and Women's Hospital
**Corresponding Author's Secondary Institution:**	
**First Author:**	Jon Haitz Legarreta
**First Author Secondary Information:**	
**Order of Authors:**	Jon Haitz Legarreta
	Simona Schiavi
	Wei Tang
	Garrett Banks
	Matthew Cieslak
	Kurt Schilling
	Alberto De Luca
	Jacques-Donald Tournier
	John Kruper
	Francois Rheault
	Stamatios N. Sotiropoulos
	Franco Pestilli
	Jelle Veraart
	Joseph Yuan-Mou Yang
	Maxime Descoteaux
	Sarah Heilbronner
	Ariel Rokem
**Order of Authors Secondary Information:**	

Powered by Editorial Manager® and ProduXion Manager® from Aries Systems Corporation

**Response to Reviewers:**	Letter to Editor<br>--------------------<br><br>Prof. Hongfang Zhang<br>Editor<br>GigaScience<br><br><br>Department of Radiology<br>Brigham and Women's Hospital<br>Harvard Medical School<br>02145 Somerville MA USA<br>jon.haitz.legarreta@gmail.com<br><br>March 13, 2026<br><br>Revision to manuscript GIGA-D-25-00365R1<br><br>The authors would like to thank the reviewers for their constructive comments and suggestions, which have helped improve the quality of this manuscript. We also thank the editor for handling the review process. The manuscript has been revised in accordance with the reviewers' comments. Please see below our point-by-point responses. For the reviewers' convenience, we have highlighted changes in the revised manuscript in strikethrough red/underlined blue.<br><br>The main changes to the manuscript are as follows:<br>- We have revised and clarified the definitions linked to the manuscript title.<br>- We have edited the "Integration of modern machine learning methods" section to clarify ambiguous statements and fully address the reviewers' comments.<br>- We have revised the "Translation of tractography methods to clinical applications" section to improve its logical flow and to provide a more accurate account of how advanced tractography models are currently used in clinical practice.<br><br>We look forward to your response.<br><br>Truly yours,<br>Jon Haitz Legarreta, PhD<br>On behalf of the co-authors<br><br><br>Response to Reviewers<br>--------------------------------<br><br>Reviewer 1<br>---------------<br><br>This revision represents an honest effort to address my original comments. I would like to share the following thoughts regarding the newly added material.<br><br><br>Reviewer Comment 1.1<br>-------------------------------<br><br>I do not find the proposed distinction between reproducibility ("ability to obtain equivalent results across sites") and robustness ("stability in the face of varying conditions (e.g., multi-site)") sufficiently clear.<br><br>Reply:<br><br>Thank you for pointing this out. It is essential that these terms be defined clearly, and we acknowledge that this was not sufficiently crisp in the revision. Therefore, we have significantly revised the first paragraph of the "Challenges and Solutions" section, where these are defined (L71-L105), which now reads:

Powered by Editorial Manager® and ProduXion Manager® from Aries Systems Corporation

Reliability, reproducibility, and robustness in tractography encompass multiple methodological dimensions. They manifest across the full data life-cycle of tractography, including acquisition, processing, and analysis. They also manifest in inferences applied across different species, different types of measurements, different ages, and across basic research and clinical application. There are many definitions of these constructs [29, 30, 31]. We define reliability as the stability of tractography algorithms in the face of varying conditions (e.g., noise perturbations in test-retest, multi-site or multi-vendor datasets, etc.). Reproducibility is defined as the ability to obtain the same results with the same data and same software used in the original study. Thus, reproducibility is mostly about the open and unencumbered availability of research products and importantly, the compliance of openly available products with conventions and standards. Replicability, a closely related term, is the ability of another research team to produce findings that are consistent without using the materials used by the original research team, but while trying to emulate the methods as closely as possible. This will depend to a large degree on adequate description of the methods used. This will also depend on the robustness of the findings, which we define as the ability to obtain the same expected anatomical observations using different data and/or different software or even methods that differ in their assumptions and implementation details. For example, the ability to delineate a certain brain structure in data obtained on different instruments, from different participants, and using different tractography algorithms. All three of these are necessary conditions for trustworthy, transparent research, and an ability for research efforts to efficiently build on previous work. As we will demonstrate in addressing a range of practical and conceptual challenges, standardization is essential to achieve these goals. Therefore, as we survey a range of challenges and proposed solutions, we will highlight key factors influencing these aspects and propose ways to increase them.

We have also introduced changes in other parts of the manuscript where these terms are used, to ensure that the definitions are consistently applied throughout.

Reviewer Comment 1.2

While I agree that ML-based tractography could benefit from increased data standardization and availability, I disagree with the suggestion that the heterogeneity in approaches that are currently being explored in related research implies an immediate need for standardization. I would argue that maintaining diversity in the approaches a scientific community explores is essential for innovation, and increases its chance of jointly arriving at a good solution.

Reply:

We agree that this is not cut and dry, and there is significant merit to these considerations. We have added the following text, based on the reviewer's comment (L581-589):

At the same time, it is important not to over-standardize too early. That is, early-stage research needs to maintain diversity in the approaches a scientific community explores, which is essential for innovation, and increases the opportunities for the community to discover good solutions. Taken together, these considerations suggest that standardization of ML/AI approaches merits caution and careful consideration, to support the goals of reliability, reproducibility, and robustness, even while not stifling innovation.

Reviewer Comment 1.3

I would also argue that the difficulty of reliable evaluation, in particular, the absence of universally accepted anatomical ground truth, is not a specific limitation for the evaluation of "AI-based" algorithms, but for the evaluation of tractography in general.

	Reply:<br><br>This is a good point, but we would still argue that AI/ML based methods are particularly hampered by these issues, because they are so oriented towards the incremental improvement of quantitative metrics. Therefore, we have added the following text (L520-L527):<br><br>The difficulty of reliable evaluation, and the absence of universally accepted anatomical ground truth in particular, is not a specific limitation for the evaluation of ML/AI algorithms, but for the evaluation of tractography in general. Nevertheless, while these issues are broadly applicable to tractography methods more generally, they are exacerbated in ML/AI methods, which are oriented towards quantitative metrics of performance for their consistent improvement.<br><br>Reviewer Comment 1.4<br>-------------------------------<br><br>I would suggest to replace the somewhat vague complaint of "Insufficient reporting of scanner or session biases that mask site or protocol-specific effects that may dominate learned representation" with a clear recommended guideline for reporting.<br><br>Reply:<br><br>We have reworded the passage and we believe that the recommendation is clearly stated now (L563-L571):<br><br>Additionally, representations and trends learned by ML/AI systems may be dominated by site- or protocol-specific effects. Systematic reporting of scanner characteristics, acquisition parameters, session-level metadata, and preprocessing choices is essential for replicability and robustness and for interpreting performance gains as genuine methodological advances<br><br>Reviewer Comment 1.5<br>-------------------------------<br><br>I understand that "fragmented research landscapes" come with challenges such as fair comparisons, but had the impression that this point was highlighted somewhat redundantly in similar words in two subsequent paragraphs.<br><br>Reply:<br><br>We agree that parts of the previous discussion were repetitive in how additional aspects of the issue were introduced. We have accordingly revised the text to eliminate redundancy and improve conceptual clarity across the two paragraphs (L590-L600):<br><br>Building on the heterogeneous methodological terrain described above, competition among large corporate entities (e.g., technology companies) is also driving further barriers to interoperability in this ecosystem, as different entities try to position their tools as dominant in the marketplace. As externally developed components are introduced into established processing pipelines, they promise incremental value but require careful accommodation within historically entrenched workflows.<br><br>Reviewer Comment 1.6<br>-------------------------------<br><br>The discussion on the translation of tractography to neurosurgery no longer accurately reflects the current state of the art. In particular, the manuscript repeats claims that have been made for more than a decade, i.e., that commercially available navigation platforms offer DTI as the only option, and that practical adoption of tractography is hindered by large false positive rates. The paper even concludes by stating that substantial efforts can be foreseen to translate more anatomically reliable tractography

methods into clinical practice. In fact, recent versions of the widely used navigation software by BrainLab already offer CSD-based reconstruction of complex fiber architectures, along with templates for a clean and automated reconstruction of many relevant tracts (see https://www.brainlab.com/surgery-products/overview-neurosurgery-products/elements-fibertracking/).

Reply:

We acknowledge that the contents were no longer coherent and have reworked the section to follow a more logical flow: starting with current uses, then discussing aspects of both prevalent and advanced models in clinical tractography, recognizing that advanced models are already in use, and finally outlining areas needing further development. We have adjusted the tone and incorporated additional references to reinforce the arguments presented.

The relevant passages of the section now read (L613-L636; L637-659; L675-L679):

Tractography is used clinically to aid in the planning and execution of neurosurgical procedures [102]. Tractography has proven useful during the resection of epileptic foci, or brain tumors [105, 106]. In this case, a surgeon might utilize a different surgical approach to the tumor to avoid certain white matter tracts, particularly those involved with motor, language and visual function. This is especially the case in slow growing tumors and pediatric developmental abnormalities, where the standard anatomical organization of white matter pathways can be significantly altered while remaining functional. To this end, tractography-guided brain tumor resections rely on functional brain mapping through direct brain electrical stimulation to confirm white matter tract positions and resection functional boundaries during awake surgery. In other instances, tractography is utilized for precision targeting in stereotactic procedures such as deep brain stimulation and focused ultrasound, such as when localizing the dentatorubrothalamic tract, a neuromodulation target for treatment of essential tremor and tremor-dominant Parkinson's disease [107, 108, 109].

Despite constituting a useful tool for improving neurosurgical outcomes and mitigating the likelihood of postoperative complications, its use remains limited [103, 110]. Diffusion tensor-based deterministic tractography remains the prevalent tool in neurosurgical preoperative planning, largely because it is supported by many commercially available navigation platforms [104]. Yet, limitations of the tensor model result in incomplete reconstruction and visualization of complex fiber architecture (e.g., crossing, fanning, and bending pathways) in clinical practice.

Tractography with advanced models is beginning to appear in commercial software, but adoption remains slow due to increased likelihood of spurious fibers, limited clinical validation, and inconsistent protocols and heterogeneous methodological frameworks [110, 111]. This is especially pronounced when examining fine-scaled structures, such as cranial nerves, and highlight the sensitivity of the tracking parameters with respect to the structures of interest [112]. Additionally, results provided by intraoperative tractography are constrained by the limited acquisition and processing time.

(...)

Thus, unlocking tractography's full potential in clinical routine necessitates further development along several critical dimensions. (...)

We have revised the related paragraph in our "Summary and recommendations" section to emphasize feasibility through the coordinated efforts required to translate basic tractography research into clinical practice (L828-L844):

Build the bridges between research and clinical tractography: Delivery of advanced methods into clinical practice can be facilitated by standardizing workflows, and by increasing the interoperability between different parts of the clinical informatics infrastructure. For example, via integration of visualization into surgery image-guided systems, into Picture Archiving and Communication Systems (PACS) used in clinical,

Powered by Editorial Manager® and ProduXion Manager® from Aries Systems Corporation

	and into electronic medical records [132]. This will also set the scene to improve the bench-to-bedside pipeline of new computational methods. Feasibility: while research and clinical requirements remain different, anatomically refined tractography methods can reach clinical practice through coordinated validation and collaboration among researchers, clinicians, and vendors.<br><br><br>Reviewer Comment 1.7<br>-------------------------------<br><br>I do not think [103] is suitable to support the claim that the use of tractography in the planning of neurosurgical procedures remains limited, given its focus on the specialized use case of intraoperative (as opposed to preoperative) tractography.<br><br>Reply:<br><br>We would like to emphasize that the introduction to the section mentions "planning and execution of neurosurgical procedures". However, we have added a very recent reference that covers a broader spectrum of tractography applications in neurosurgery, while clearly acknowledging the need for methodological improvements and standardization for routine clinical use. The reference added is:<br><br>Sarubbo S, Vergani F, Yang JYM. Tractography in brain tumor surgery: current clinical impact and future challenges. Brain Structure and Function 2025;230(6):93. https://doi.org/10.1007/s00429-025-02956-y.
**Additional Information:**	
**Question**	**Response**
Are you submitting this manuscript to a special series or article collection?	No
**Experimental design and statistics**<br><br><br>Full details of the experimental design and statistical methods used should be given in the Methods section, as detailed in our Minimum Standards Reporting Checklist. Information essential to interpreting the data presented should be made available in the figure legends.<br><br><br>Have you included all the information requested in your manuscript?	Yes
**Resources**<br><br><br>A description of all resources used, including antibodies, cell lines, animals and software tools, with enough information to allow them to be uniquely identified, should be included in the Methods section. Authors are strongly encouraged to cite Research Resource Identifiers (RRIDs) for antibodies, model	Yes

organisms and tools, where possible.<br><br>Have you included the information requested as detailed in our Minimum Standards Reporting Checklist?	
**Availability of data and materials**<br><br>All datasets and code on which the conclusions of the paper rely must be either included in your submission or deposited in publicly available repositories (where available and ethically appropriate), referencing such data using a unique identifier in the references and in the "Availability of Data and Materials" section of your manuscript.<br><br>Have you have met the above requirement as detailed in our Minimum Standards Reporting Checklist?	Yes
GigaScience has policies and guidelines in place for the use of generative AI-writing tools such as ChatGPT. If you have used such writing tools to assist with writing the manuscript this must be declared and cited in the text. Authors should not list AI-writing tools and other AI-assisted technologies as an author or co-author and should acknowledge that they are fully responsible for text generated or refined by AI-writing tools.<p><br><br>A summary of use (particularly in the introduction or among methods) needs to be included at the end of the paper, and the outputs should also be included as a supplementary file hosted in GigaDB or other open repositories. Please read our guidelines for more information. <p><br><br>By submitting to GigaScience, you are aware of the journal's AI-writing tools policy, and if you have declared use of such tools below, you have acknowledged this where appropriate in your manuscript and have made a summary of use and outputs available. <p><br><b>AI-assisted writing tools have been	No

used in the preparation of this manuscript?	

Powered by Editorial Manager® and ProduXion Manager® from Aries Systems Corporation

Click here to access/download;LaTeX - Main Document (TeX file);main.tex

```
This is pdfTeX, Version 3.141592653-2.6-1.40.26 (TeX Live 2024)
(preloaded format=pdflatex 2024.8.2)  23 MAR 2026 22:15
entering extended mode
 restricted \write18 enabled.
 %&-line parsing enabled.
**main.tex
(./main.tex
LaTeX2e <2024-06-01> patch level 2
L3 programming layer <2024-05-27>
(./oup-contemporary.cls
Document Class: oup-contemporary 2023/06/12, v1.2
(c:/texlive/2024/texmf-dist/tex/latex/base/article.cls
Document Class: article 2024/02/08 v1.4n Standard LaTeX document class
(c:/texlive/2024/texmf-dist/tex/latex/base/size10.clo
File: size10.clo 2024/02/08 v1.4n Standard LaTeX file (size option)
)
\c@part=\count194
\c@section=\count195
\c@subsection=\count196
\c@subsubsection=\count197
\c@paragraph=\count198
\c@subparagraph=\count199
\c@figure=\count266
\c@table=\count267
\abovecaptionskip=\skip49
\belowcaptionskip=\skip50
\bibindent=\dimen141
) (c:/texlive/2024/texmf-dist/tex/latex/base/inputenc.sty
Package: inputenc 2024/02/08 v1.3d Input encoding file
\inpenc@prehook=\toks17
\inpenc@posthook=\toks18
) (c:/texlive/2024/texmf-dist/tex/latex/base/fontenc.sty
Package: fontenc 2021/04/29 v2.0v Standard LaTeX package
) (c:/texlive/2024/texmf-dist/tex/generic/iftex/ifpdf.sty
Package: ifpdf 2019/10/25 v3.4 ifpdf legacy package. Use iftex instead.
(c:/texlive/2024/texmf-dist/tex/generic/iftex/iftex.sty
Package: iftex 2022/02/03 v1.0f TeX engine tests
)) (c:/texlive/2024/texmf-dist/tex/latex/microtype/microtype.sty
Package: microtype 2024/03/29 v3.1b Micro-typographical refinements (RS)
(c:/texlive/2024/texmf-dist/tex/latex/graphics/keyval.sty
Package: keyval 2022/05/29 v1.15 key=value parser (DPC)
\KV@toks@=\toks19
) (c:/texlive/2024/texmf-dist/tex/latex/etoolbox/etoolbox.sty
Package: etoolbox 2020/10/05 v2.5k e-TeX tools for LaTeX (JAW)
\etb@tempcnta=\count268
)
\MT@toks=\toks20
\MT@tempbox=\box52
\MT@count=\count269
LaTeX Info: Redefining \noprotrusionifhmode on input line 1061.
LaTeX Info: Redefining \leftprotrusion on input line 1062.
\MT@prot@toks=\toks21
LaTeX Info: Redefining \rightprotrusion on input line 1081.
LaTeX Info: Redefining \textls on input line 1392.
```

```
\MT@outer@kern=\dimen142
LaTeX Info: Redefining \textmicrotypecontext on input line 2013.
\MT@listname@count=\count270
(c:/texlive/2024/texmf-dist/tex/latex/microtype/microtype-pdftex.def
File: microtype-pdftex.def 2024/03/29 v3.1b Definitions specific to
pdftex (RS)

LaTeX Info: Redefining \lsstyle on input line 902.
LaTeX Info: Redefining \lslig on input line 902.
\MT@outer@space=\skip51
)
Package microtype Info: Loading configuration file microtype.cfg.
(c:/texlive/2024/texmf-dist/tex/latex/microtype/microtype.cfg
File: microtype.cfg 2024/03/29 v3.1b microtype main configuration file
(RS)
)) (c:/texlive/2024/texmf-dist/tex/latex/euler/euler.sty
Package: euler 1995/03/05 v2.5
Package: `euler' v2.5 <1995/03/05> (FJ and FMi)
LaTeX Font Info:    Redeclaring symbol font `letters' on input line 35.
LaTeX Font Info:    Encoding `OML' has changed to `U' for symbol font
(Font)              `letters' in the math version `normal' on input line
35.
LaTeX Font Info:    Overwriting symbol font `letters' in version `normal'
(Font)                  OML/cmm/m/it --> U/eur/m/n on input line 35.
LaTeX Font Info:    Encoding `OML' has changed to `U' for symbol font
(Font)              `letters' in the math version `bold' on input line
35.
LaTeX Font Info:    Overwriting symbol font `letters' in version `bold'
(Font)                  OML/cmm/b/it --> U/eur/m/n on input line 35.
LaTeX Font Info:    Overwriting symbol font `letters' in version `bold'
(Font)                  U/eur/m/n --> U/eur/b/n on input line 36.
LaTeX Font Info:    Redeclaring math symbol \Gamma on input line 47.
LaTeX Font Info:    Redeclaring math symbol \Delta on input line 48.
LaTeX Font Info:    Redeclaring math symbol \Theta on input line 49.
LaTeX Font Info:    Redeclaring math symbol \Lambda on input line 50.
LaTeX Font Info:    Redeclaring math symbol \Xi on input line 51.
LaTeX Font Info:    Redeclaring math symbol \Pi on input line 52.
LaTeX Font Info:    Redeclaring math symbol \Sigma on input line 53.
LaTeX Font Info:    Redeclaring math symbol \Upsilon on input line 54.
LaTeX Font Info:    Redeclaring math symbol \Phi on input line 55.
LaTeX Font Info:    Redeclaring math symbol \Psi on input line 56.
LaTeX Font Info:    Redeclaring math symbol \Omega on input line 57.
\symEulerFraktur=\mathgroup4
LaTeX Font Info:    Overwriting symbol font `EulerFraktur' in version
`bold'
(Font)                  U/euf/m/n --> U/euf/b/n on input line 63.
LaTeX Info: Redefining \oldstylenums on input line 85.
\symEulerScript=\mathgroup5
LaTeX Font Info:    Overwriting symbol font `EulerScript' in version
`bold'
(Font)                  U/eus/m/n --> U/eus/b/n on input line 93.
LaTeX Font Info:    Redeclaring math symbol \aleph on input line 97.
LaTeX Font Info:    Redeclaring math symbol \Re on input line 98.
LaTeX Font Info:    Redeclaring math symbol \Im on input line 99.
```

LaTeX Font Info: Redeclaring math delimiter \vert on input line 101.
LaTeX Font Info: Redeclaring math delimiter \backslash on input line
103.
LaTeX Font Info: Redeclaring math symbol \neg on input line 106.
LaTeX Font Info: Redeclaring math symbol \wedge on input line 108.
LaTeX Font Info: Redeclaring math symbol \vee on input line 110.
LaTeX Font Info: Redeclaring math symbol \setminus on input line 112.
LaTeX Font Info: Redeclaring math symbol \sim on input line 113.
LaTeX Font Info: Redeclaring math symbol \mid on input line 114.
LaTeX Font Info: Redeclaring math delimiter \arrowvert on input line
116.
LaTeX Font Info: Redeclaring math symbol \mathsection on input line
117.
\symEulerExtension=\mathgroup6
LaTeX Font Info: Redeclaring math symbol \coprod on input line 125.
LaTeX Font Info: Redeclaring math symbol \prod on input line 125.
LaTeX Font Info: Redeclaring math symbol \sum on input line 125.
LaTeX Font Info: Redeclaring math symbol \intop on input line 130.
LaTeX Font Info: Redeclaring math symbol \ointop on input line 131.
LaTeX Font Info: Redeclaring math symbol \braceld on input line 132.
LaTeX Font Info: Redeclaring math symbol \bracerd on input line 133.
LaTeX Font Info: Redeclaring math symbol \bracelu on input line 134.
LaTeX Font Info: Redeclaring math symbol \braceru on input line 135.
LaTeX Font Info: Redeclaring math symbol \infty on input line 136.
LaTeX Font Info: Redeclaring math symbol \nearrow on input line 153.
LaTeX Font Info: Redeclaring math symbol \searrow on input line 154.
LaTeX Font Info: Redeclaring math symbol \nwarrow on input line 155.
LaTeX Font Info: Redeclaring math symbol \swarrow on input line 156.
LaTeX Font Info: Redeclaring math symbol \Leftrightarrow on input line
157.
LaTeX Font Info: Redeclaring math symbol \Leftarrow on input line 158.
LaTeX Font Info: Redeclaring math symbol \Rightarrow on input line
159.
LaTeX Font Info: Redeclaring math symbol \leftrightarrow on input line
160.
LaTeX Font Info: Redeclaring math symbol \leftarrow on input line 161.
LaTeX Font Info: Redeclaring math symbol \rightarrow on input line
163.
LaTeX Font Info: Redeclaring math delimiter \uparrow on input line
166.
LaTeX Font Info: Redeclaring math delimiter \downarrow on input line
168.
LaTeX Font Info: Redeclaring math delimiter \updownarrow on input line
170.
LaTeX Font Info: Redeclaring math delimiter \Uparrow on input line
172.
LaTeX Font Info: Redeclaring math delimiter \Downarrow on input line
174.
LaTeX Font Info: Redeclaring math delimiter \Updownarrow on input line
176.
LaTeX Font Info: Redeclaring math symbol \leftharpoonup on input line
177.
LaTeX Font Info: Redeclaring math symbol \leftharpoondown on input
line 178.

```
LaTeX Font Info:    Redeclaring math symbol \rightharpoonup on input line
179.
LaTeX Font Info:    Redeclaring math symbol \rightharpoondown on input
line 180
.
LaTeX Font Info:    Redeclaring math delimiter \lbrace on input line 182.
LaTeX Font Info:    Redeclaring math delimiter \rbrace on input line 184.
\symcmmigroup=\mathgroup7
LaTeX Font Info:    Overwriting symbol font `cmmigroup' in version `bold'
(Font)                  OML/cmm/m/it --> OML/cmm/b/it on input line 200.
LaTeX Font Info:    Redeclaring math accent \vec on input line 201.
LaTeX Font Info:    Redeclaring math symbol \triangleleft on input line
202.
LaTeX Font Info:    Redeclaring math symbol \triangleright on input line
203.
LaTeX Font Info:    Redeclaring math symbol \star on input line 204.
LaTeX Font Info:    Redeclaring math symbol \lhook on input line 205.
LaTeX Font Info:    Redeclaring math symbol \rhook on input line 206.
LaTeX Font Info:    Redeclaring math symbol \flat on input line 207.
LaTeX Font Info:    Redeclaring math symbol \natural on input line 208.
LaTeX Font Info:    Redeclaring math symbol \sharp on input line 209.
LaTeX Font Info:    Redeclaring math symbol \smile on input line 210.
LaTeX Font Info:    Redeclaring math symbol \frown on input line 211.
LaTeX Font Info:    Redeclaring math accent \grave on input line 245.
LaTeX Font Info:    Redeclaring math accent \acute on input line 246.
LaTeX Font Info:    Redeclaring math accent \tilde on input line 247.
LaTeX Font Info:    Redeclaring math accent \ddot on input line 248.
LaTeX Font Info:    Redeclaring math accent \check on input line 249.
LaTeX Font Info:    Redeclaring math accent \breve on input line 250.
LaTeX Font Info:    Redeclaring math accent \bar on input line 251.
LaTeX Font Info:    Redeclaring math accent \dot on input line 252.
LaTeX Font Info:    Redeclaring math accent \hat on input line 254.
) (c:/texlive/2024/texmf-dist/tex/latex/merriweather/merriweather.sty
Package: merriweather 2022/09/20 (Bob Tennent) Supports
Merriweather(Sans) font
s for all LaTeX engines.
(c:/texlive/2024/texmf-dist/tex/generic/iftex/ifxetex.sty
Package: ifxetex 2019/10/25 v0.7 ifxetex legacy package. Use iftex
instead.
) (c:/texlive/2024/texmf-dist/tex/generic/iftex/ifluatex.sty
Package: ifluatex 2019/10/25 v1.5 ifluatex legacy package. Use iftex
instead.
) (c:/texlive/2024/texmf-dist/tex/latex/base/textcomp.sty
Package: textcomp 2024/04/24 v2.1b Standard LaTeX package
) (c:/texlive/2024/texmf-dist/tex/latex/xkeyval/xkeyval.sty
Package: xkeyval 2022/06/16 v2.9 package option processing (HA)
(c:/texlive/2024/texmf-dist/tex/generic/xkeyval/xkeyval.tex
(c:/texlive/2024/te
xmf-dist/tex/generic/xkeyval/xkvutils.tex
\XKV@toks=\toks22
\XKV@tempa@toks=\toks23
)
\XKV@depth=\count271
```

```
File: xkeyval.tex 2014/12/03 v2.7a key=value parser (HA)
)) (c:/texlive/2024/texmf-dist/tex/latex/base/fontenc.sty
Package: fontenc 2021/04/29 v2.0v Standard LaTeX package
) (c:/texlive/2024/texmf-dist/tex/latex/fontaxes/fontaxes.sty
Package: fontaxes 2020/07/21 v1.0e Font selection axes
LaTeX Info: Redefining \upshape on input line 29.
LaTeX Info: Redefining \itshape on input line 31.
LaTeX Info: Redefining \slshape on input line 33.
LaTeX Info: Redefining \swshape on input line 35.
LaTeX Info: Redefining \scshape on input line 37.
LaTeX Info: Redefining \sscshape on input line 39.
LaTeX Info: Redefining \ulcshape on input line 41.
LaTeX Info: Redefining \textsw on input line 47.
LaTeX Info: Redefining \textssc on input line 48.
LaTeX Info: Redefining \textulc on input line 49.
)) (c:/texlive/2024/texmf-dist/tex/latex/mathastext/mathastext.sty
Package: mathastext 2024/07/27 v1.4b Use the text font in math mode (JFB)

Package mathastext Info: Starting the math mode configuration.
\mst@exists@muskip=\muskip17
\mst@forall@muskip=\muskip18
\mst@prime@muskip=\muskip19
\mst@do@nonletters=\toks24
\mst@undo@nonletters=\toks25
\mst@do@easynonletters=\toks26
\mst@undo@easynonletters=\toks27
\symmtoperatorfont=\mathgroup8
\symmtletterfont=\mathgroup9
(   mathastext:   ) ! and ?
(   mathastext:   ) punctuation: , . : ; and \colon
LaTeX Info: Redefining \relbar on input line 1201.
LaTeX Info: Redefining \rightarrowfill on input line 1202.
LaTeX Info: Redefining \leftarrowfill on input line 1205.
(   mathastext:   ) + and =
LaTeX Info: Redefining \Relbar on input line 1298.
(   mathastext:   ) adding = ; and + to \nfss@catcodes
(   mathastext:   ) parentheses ( ) [ ] and slash /
(   mathastext:   ) alldelims: < > \backslash \setminus | \vert \mid \{
\}
LaTeX Font Info:    Redeclaring math symbol \setminus on input line 1364.
LaTeX Info: Redefining \models on input line 1383.
(   mathastext:   ) \# \mathdollar \% \&
(   mathastext:   ) \imath and \jmath
LaTeX Font Info:    Overwriting math alphabet `\Mathnormalbold' in
version `nor
mal'
(Font)                  T1/Merriwthr-OsF/b/it --> T1/Merriwthr-OsF/b/it
on inpu
t line 2863.
LaTeX Font Info:    Overwriting math alphabet `\Mathnormalbold' in
version `bol
d'
(Font)                  T1/Merriwthr-OsF/b/it --> T1/Merriwthr-OsF/b/it
on inpu
```

```
t line 2863.
LaTeX Font Info:    Overwriting symbol font `mtletterfont' in version
`normal'
(Font)                  T1/Merriwthr-OsF/m/it --> T1/Merriwthr-OsF/m/it
on inpu
t line 2863.
LaTeX Font Info:    Overwriting symbol font `mtletterfont' in version
`bold'
(Font)                  T1/Merriwthr-OsF/m/it --> T1/Merriwthr-OsF/b/it
on inpu
t line 2863.
LaTeX Font Info:    Overwriting symbol font `mtoperatorfont' in version
`normal
'
(Font)                  T1/Merriwthr-OsF/m/n --> T1/Merriwthr-OsF/m/n on
input
line 2863.
LaTeX Font Info:    Overwriting symbol font `mtoperatorfont' in version
`bold'
(Font)                  T1/Merriwthr-OsF/m/n --> T1/Merriwthr-OsF/b/n on
input
line 2863.
LaTeX Font Info:    Overwriting math alphabet `\Mathbf' in version
`normal'
(Font)                  T1/Merriwthr-OsF/b/n --> T1/Merriwthr-OsF/b/n on
input
line 2863.
LaTeX Font Info:    Overwriting math alphabet `\Mathbf' in version `bold'
(Font)                  T1/Merriwthr-OsF/b/n --> T1/Merriwthr-OsF/b/n on
input
line 2863.
LaTeX Font Info:    Overwriting math alphabet `\Mathit' in version
`normal'
(Font)                  T1/Merriwthr-OsF/m/it --> T1/Merriwthr-OsF/m/it
on inpu
t line 2863.
LaTeX Font Info:    Overwriting math alphabet `\Mathit' in version `bold'
(Font)                  T1/Merriwthr-OsF/m/it --> T1/Merriwthr-OsF/b/it
on inpu
t line 2863.
LaTeX Font Info:    Overwriting math alphabet `\Mathsf' in version
`normal'
(Font)                  T1/MerriwthrSans-OsF/m/n --> T1/MerriwthrSans-
OsF/m/n o
n input line 2863.
LaTeX Font Info:    Overwriting math alphabet `\Mathsf' in version `bold'
(Font)                  T1/MerriwthrSans-OsF/m/n --> T1/MerriwthrSans-
OsF/b/n o
n input line 2863.
LaTeX Font Info:    Overwriting math alphabet `\Mathtt' in version
`normal'
(Font)                  T1/lmtt/m/n --> T1/lmtt/m/n on input line 2863.
LaTeX Font Info:    Overwriting math alphabet `\Mathtt' in version `bold'
(Font)                  T1/lmtt/m/n --> T1/lmtt/b/n on input line 2863.
```

```
(   mathastext:   ) Latin letters in the `normal', resp. `bold',
(   mathastext:   ) math versions are now set up to use the fonts
(   mathastext:   ) T1/Merriwthr-OsF/m/it, resp. T1/Merriwthr-OsF/b/it.
(   mathastext:   ) Other characters (digits, ...) and \log-like names
will be
(   mathastext:   ) typeset with the n shape.
(   mathastext:   ) \hbar
(   mathastext:   ) minus as endash
(   mathastext:   ) The italic option is in effect.
(   mathastext:   ) \HUGE has been (re)-defined.
(   mathastext:   ) mathastext has declared larger sizes for subscripts.
(   mathastext:   ) To keep LaTeX defaults, use option
`defaultmathsizes'.

Package mathastext Info: Loading is complete.  You can now use
\Mathastext to
(mathastext)             modify the normal and bold math versions.  Use
it
(mathastext)             with optional argument or use \MTDeclareVersion
to
(mathastext)             declare additional math versions.
) (c:/texlive/2024/texmf-dist/tex/latex/relsize/relsize.sty
Package: relsize 2013/03/29 ver 4.1
) (c:/texlive/2024/texmf-dist/tex/latex/ragged2e/ragged2e.sty
Package: ragged2e 2023/06/22 v3.6 ragged2e Package
\CenteringLeftskip=\skip52
\RaggedLeftLeftskip=\skip53
\RaggedRightLeftskip=\skip54
\CenteringRightskip=\skip55
\RaggedLeftRightskip=\skip56
\RaggedRightRightskip=\skip57
\CenteringParfillskip=\skip58
\RaggedLeftParfillskip=\skip59
\RaggedRightParfillskip=\skip60
\JustifyingParfillskip=\skip61
\CenteringParindent=\skip62
\RaggedLeftParindent=\skip63
\RaggedRightParindent=\skip64
\JustifyingParindent=\skip65
) (c:/texlive/2024/texmf-dist/tex/latex/xcolor/xcolor.sty
Package: xcolor 2023/11/15 v3.01 LaTeX color extensions (UK)
(c:/texlive/2024/texmf-dist/tex/latex/graphics-cfg/color.cfg
File: color.cfg 2016/01/02 v1.6 sample color configuration
)
Package xcolor Info: Driver file: pdftex.def on input line 274.
(c:/texlive/2024/texmf-dist/tex/latex/graphics-def/pdftex.def
File: pdftex.def 2024/04/13 v1.2c Graphics/color driver for pdftex
) (c:/texlive/2024/texmf-dist/tex/latex/graphics/mathcolor.ltx)
Package xcolor Info: Model `cmy' substituted by `cmy0' on input line
1350.
Package xcolor Info: Model `hsb' substituted by `rgb' on input line 1354.
Package xcolor Info: Model `RGB' extended on input line 1366.
Package xcolor Info: Model `HTML' substituted by `rgb' on input line
1368.
```

```
Package xcolor Info: Model `Hsb' substituted by `hsb' on input line 1369.
Package xcolor Info: Model `tHsb' substituted by `hsb' on input line
1370.
Package xcolor Info: Model `HSB' substituted by `hsb' on input line 1371.
Package xcolor Info: Model `Gray' substituted by `gray' on input line
1372.
Package xcolor Info: Model `wave' substituted by `hsb' on input line
1373.
) (c:/texlive/2024/texmf-dist/tex/latex/colortbl/colortbl.sty
Package: colortbl 2024/07/06 v1.0i Color table columns (DPC)
(c:/texlive/2024/texmf-dist/tex/latex/tools/array.sty
Package: array 2024/06/14 v2.6d Tabular extension package (FMi)
\col@sep=\dimen143
\ar@mcellbox=\box53
\extrarowheight=\dimen144
\NC@list=\toks28
\extratabsurround=\skip66
\backup@length=\skip67
\ar@cellbox=\box54
)
\everycr=\toks29
\minrowclearance=\skip68
\rownum=\count272
) (c:/texlive/2024/texmf-dist/tex/latex/graphics/graphicx.sty
Package: graphicx 2021/09/16 v1.2d Enhanced LaTeX Graphics (DPC,SPQR)
(c:/texlive/2024/texmf-dist/tex/latex/graphics/graphics.sty
Package: graphics 2024/05/23 v1.4g Standard LaTeX Graphics (DPC,SPQR)
(c:/texlive/2024/texmf-dist/tex/latex/graphics/trig.sty
Package: trig 2023/12/02 v1.11 sin cos tan (DPC)
) (c:/texlive/2024/texmf-dist/tex/latex/graphics-cfg/graphics.cfg
File: graphics.cfg 2016/06/04 v1.11 sample graphics configuration
)
Package graphics Info: Driver file: pdftex.def on input line 106.
)
\Gin@req@height=\dimen145
\Gin@req@width=\dimen146
) (c:/texlive/2024/texmf-dist/tex/latex/xpatch/xpatch.sty
(c:/texlive/2024/texm
f-dist/tex/latex/l3kernel/expl3.sty
Package: expl3 2024-05-27 L3 programming layer (loader)
(c:/texlive/2024/texmf-dist/tex/latex/l3backend/l3backend-pdftex.def
File: l3backend-pdftex.def 2024-05-08 L3 backend support: PDF output
(pdfTeX)
\l__color_backend_stack_int=\count273
\l__pdf_internal_box=\box55
))
Package: xpatch 2020/03/25 v0.3a Extending etoolbox patching commands
(c:/texlive/2024/texmf-dist/tex/latex/l3packages/xparse/xparse.sty
Package: xparse 2024-05-08 L3 Experimental document command parser
)) (c:/texlive/2024/texmf-dist/tex/latex/environ/environ.sty
Package: environ 2014/05/04 v0.3 A new way to define environments
(c:/texlive/2024/texmf-dist/tex/latex/trimspaces/trimspaces.sty
Package: trimspaces 2009/09/17 v1.1 Trim spaces around a token list
)
```

```
\@envbody=\toks30
) (c:/texlive/2024/texmf-dist/tex/latex/lastpage/lastpage.sty
Package: lastpage 2024/07/07 v2.1c lastpage: 2.09 or 2e? (HMM)
(c:/texlive/2024/texmf-dist/tex/latex/lastpage/lastpage2e.sty
Package: lastpage2e 2024/07/07 v2.1c Decide which 2e lastpage version to
use (H
MM)
(c:/texlive/2024/texmf-dist/tex/latex/lastpage/lastpagemodern.sty
Package: lastpagemodern 2024-07-07 v2.1c Refers to last page's name (HMM;
JPG)
\c@lastpagecount=\count274
)
)) (c:/texlive/2024/texmf-dist/tex/latex/graphics/rotating.sty
Package: rotating 2016/08/11 v2.16d rotated objects in LaTeX
(c:/texlive/2024/texmf-dist/tex/latex/base/ifthen.sty
Package: ifthen 2024/03/16 v1.1e Standard LaTeX ifthen package (DPC)
)
\c@r@tfl@t=\count275
\rotFPtop=\skip69
\rotFPbot=\skip70
\rot@float@box=\box56
\rot@mess@toks=\toks31
) (c:/texlive/2024/texmf-dist/tex/latex/graphics/lscape.sty
Package: lscape 2020/05/28 v3.02 Landscape Pages (DPC)
) (c:/texlive/2024/texmf-dist/tex/latex/tools/afterpage.sty
Package: afterpage 2023/07/04 v1.08 After-Page Package (DPC)
\AP@output=\toks32
\AP@partial=\box57
\AP@footins=\box58
) (c:/texlive/2024/texmf-dist/tex/latex/textpos/textpos.sty
Package: textpos 2022/07/23 v1.10.1
Package textpos Info: choosing support for LaTeX3 on input line 60.
\TP@textbox=\box59
\TP@holdbox=\box60
\TPHorizModule=\dimen147
\TPVertModule=\dimen148
\TP@margin=\dimen149
\TP@absmargin=\dimen150
Grid set 16 x 16 = 37.34424pt x 52.81541pt
\TPboxrulesize=\dimen151
\TP@ox=\dimen152
\TP@oy=\dimen153
\TP@tbargs=\toks33
TextBlockOrigin set to 0pt x 0pt
) (c:/texlive/2024/texmf-dist/tex/latex/url/url.sty
\Urlmuskip=\muskip20
Package: url 2013/09/16  ver 3.4  Verb mode for urls, etc.
) (c:/texlive/2024/texmf-dist/tex/latex/newfloat/newfloat.sty
Package: newfloat 2023/10/01 v1.2 Defining new floating environments (AR)
Package newfloat Info: `rotating' package detected.
) (c:/texlive/2024/texmf-dist/tex/latex/mdframed/mdframed.sty
Package: mdframed 2013/07/01 1.9b: mdframed
(c:/texlive/2024/texmf-dist/tex/latex/kvoptions/kvoptions.sty
```

```
Package: kvoptions 2022-06-15 v3.15 Key value format for package options
(HO)
(c:/texlive/2024/texmf-dist/tex/generic/ltxcmds/ltxcmds.sty
Package: ltxcmds 2023-12-04 v1.26 LaTeX kernel commands for general use
(HO)
) (c:/texlive/2024/texmf-dist/tex/latex/kvsetkeys/kvsetkeys.sty
Package: kvsetkeys 2022-10-05 v1.19 Key value parser (HO)
)) (c:/texlive/2024/texmf-dist/tex/latex/zref/zref-abspage.sty
Package: zref-abspage 2023-09-14 v2.35 Module abspage for zref (HO)
(c:/texlive/2024/texmf-dist/tex/latex/zref/zref-base.sty
Package: zref-base 2023-09-14 v2.35 Module base for zref (HO)
(c:/texlive/2024/texmf-dist/tex/generic/infwarerr/infwarerr.sty
Package: infwarerr 2019/12/03 v1.5 Providing info/warning/error messages
(HO)
) (c:/texlive/2024/texmf-dist/tex/generic/kvdefinekeys/kvdefinekeys.sty
Package: kvdefinekeys 2019-12-19 v1.6 Define keys (HO)
) (c:/texlive/2024/texmf-dist/tex/generic/pdftexcmds/pdftexcmds.sty
Package: pdftexcmds 2020-06-27 v0.33 Utility functions of pdfTeX for
LuaTeX (HO
)
Package pdftexcmds Info: \pdf@primitive is available.
Package pdftexcmds Info: \pdf@ifprimitive is available.
Package pdftexcmds Info: \pdfdraftmode found.
) (c:/texlive/2024/texmf-dist/tex/generic/etexcmds/etexcmds.sty
Package: etexcmds 2019/12/15 v1.7 Avoid name clashes with e-TeX commands
(HO)
) (c:/texlive/2024/texmf-dist/tex/latex/auxhook/auxhook.sty
Package: auxhook 2019-12-17 v1.6 Hooks for auxiliary files (HO)
)
Package zref Info: New property list: main on input line 767.
Package zref Info: New property: default on input line 768.
Package zref Info: New property: page on input line 769.
)
\c@abspage=\count276
Package zref Info: New property: abspage on input line 67.
) (c:/texlive/2024/texmf-dist/tex/latex/needspace/needspace.sty
Package: needspace 2010/09/12 v1.3d reserve vertical space
)
\mdf@templength=\skip71
\c@mdf@globalstyle@cnt=\count277
\mdf@skipabove@length=\skip72
\mdf@skipbelow@length=\skip73
\mdf@leftmargin@length=\skip74
\mdf@rightmargin@length=\skip75
\mdf@innerleftmargin@length=\skip76
\mdf@innerrightmargin@length=\skip77
\mdf@innertopmargin@length=\skip78
\mdf@innerbottommargin@length=\skip79
\mdf@splittopskip@length=\skip80
\mdf@splitbottomskip@length=\skip81
\mdf@outermargin@length=\skip82
\mdf@innermargin@length=\skip83
\mdf@linewidth@length=\skip84
\mdf@innerlinewidth@length=\skip85
```

```
\mdf@middlelinewidth@length=\skip86
\mdf@outerlinewidth@length=\skip87
\mdf@roundcorner@length=\skip88
\mdf@footenotedistance@length=\skip89
\mdf@userdefinedwidth@length=\skip90
\mdf@needspace@length=\skip91
\mdf@frametitleaboveskip@length=\skip92
\mdf@frametitlebelowskip@length=\skip93
\mdf@frametitlerulewidth@length=\skip94
\mdf@frametitleleftmargin@length=\skip95
\mdf@frametitlerightmargin@length=\skip96
\mdf@shadowsize@length=\skip97
\mdf@extratopheight@length=\skip98
\mdf@subtitleabovelinewidth@length=\skip99
\mdf@subtitlebelowlinewidth@length=\skip100
\mdf@subtitleaboveskip@length=\skip101
\mdf@subtitlebelowskip@length=\skip102
\mdf@subtitleinneraboveskip@length=\skip103
\mdf@subtitleinnerbelowskip@length=\skip104
\mdf@subsubtitleabovelinewidth@length=\skip105
\mdf@subsubtitlebelowlinewidth@length=\skip106
\mdf@subsubtitleaboveskip@length=\skip107
\mdf@subsubtitlebelowskip@length=\skip108
\mdf@subsubtitleinneraboveskip@length=\skip109
\mdf@subsubtitleinnerbelowskip@length=\skip110
(c:/texlive/2024/texmf-dist/tex/latex/mdframed/md-frame-0.mdf
File: md-frame-0.mdf 2013/07/01\ 1.9b: md-frame-0
)
\mdf@frametitlebox=\box61
\mdf@footnotebox=\box62
\mdf@splitbox@one=\box63
\mdf@splitbox@two=\box64
\mdf@splitbox@save=\box65
\mdfsplitboxwidth=\skip111
\mdfsplitboxtotalwidth=\skip112
\mdfsplitboxheight=\skip113
\mdfsplitboxdepth=\skip114
\mdfsplitboxtotalheight=\skip115
\mdfframetitleboxwidth=\skip116
\mdfframetitleboxtotalwidth=\skip117
\mdfframetitleboxheight=\skip118
\mdfframetitleboxdepth=\skip119
\mdfframetitleboxtotalheight=\skip120
\mdffootnoteboxwidth=\skip121
\mdffootnoteboxtotalwidth=\skip122
\mdffootnoteboxheight=\skip123
\mdffootnoteboxdepth=\skip124
\mdffootnoteboxtotalheight=\skip125
\mdftotallinewidth=\skip126
\mdfboundingboxwidth=\skip127
\mdfboundingboxtotalwidth=\skip128
\mdfboundingboxheight=\skip129
\mdfboundingboxdepth=\skip130
\mdfboundingboxtotalheight=\skip131
```

```
\mdf@freevspace@length=\skip132
\mdf@horizontalwidthofbox@length=\skip133
\mdf@verticalmarginwhole@length=\skip134
\mdf@horizontalspaceofbox=\skip135
\mdfsubtitleheight=\skip136
\mdfsubsubtitleheight=\skip137
\c@mdfcountframes=\count278

****** mdframed patching \endmdf@trivlist

****** -- success******

\mdf@envdepth=\count279
\c@mdf@env@i=\count280
\c@mdf@env@ii=\count281
\c@mdf@zref@counter=\count282
Package zref Info: New property: mdf@pagevalue on input line 895.
) (c:/texlive/2024/texmf-dist/tex/latex/titlesec/titlesec.sty
Package: titlesec 2023/10/27 v2.16 Sectioning titles
\ttl@box=\box66
\beforetitleunit=\skip138
\aftertitleunit=\skip139
\ttl@plus=\dimen154
\ttl@minus=\dimen155
\ttl@toksa=\toks34
\titlewidth=\dimen156
\titlewidthlast=\dimen157
\titlewidthfirst=\dimen158
) (c:/texlive/2024/texmf-dist/tex/latex/koma-script/scrextend.sty
Package: scrextend 2023/07/07 v3.41 KOMA-Script package (extend other
classes w
ith features of KOMA-Script classes)
(c:/texlive/2024/texmf-dist/tex/latex/koma-script/scrkbase.sty
Package: scrkbase 2023/07/07 v3.41 KOMA-Script package (KOMA-Script-
dependent b
asics and keyval usage)
(c:/texlive/2024/texmf-dist/tex/latex/koma-script/scrbase.sty
Package: scrbase 2023/07/07 v3.41 KOMA-Script package (KOMA-Script-
independent
basics and keyval usage)
(c:/texlive/2024/texmf-dist/tex/latex/koma-script/scrlfile.sty
Package: scrlfile 2023/07/07 v3.41 KOMA-Script package (file load hooks)
(c:/texlive/2024/texmf-dist/tex/latex/koma-script/scrlfile-hook.sty
Package: scrlfile-hook 2023/07/07 v3.41 KOMA-Script package (using LaTeX
hooks)

(c:/texlive/2024/texmf-dist/tex/latex/koma-script/scrlogo.sty
Package: scrlogo 2023/07/07 v3.41 KOMA-Script package (logo)
)))
Applying: [2021/05/01] Usage of raw or classic option list on input line
252.
Already applied: [0000/00/00] Usage of raw or classic option list on
input line
 368.
```

```
))
Package scrextend Info: unexpected definition of `\@makefnmark'.
(scrextend)             Trying to patch it on input line 1762.
Package scrextend Info: patch seems to be successfull on input line 1762.
)

LaTeX Font Warning: Font shape `T1/cmr/m/n' in size <7.5> not available
(Font)              size <7> substituted on input line 69.

(c:/texlive/2024/texmf-dist/tex/latex/tools/calc.sty
Package: calc 2023/07/08 v4.3 Infix arithmetic (KKT,FJ)
\calc@Acount=\count283
\calc@Bcount=\count284
\calc@Adimen=\dimen159
\calc@Bdimen=\dimen160
\calc@Askip=\skip140
\calc@Bskip=\skip141
LaTeX Info: Redefining \setlength on input line 80.
LaTeX Info: Redefining \addtolength on input line 81.
\calc@Ccount=\count285
\calc@Cskip=\skip142
) (c:/texlive/2024/texmf-dist/tex/latex/geometry/geometry.sty
Package: geometry 2020/01/02 v5.9 Page Geometry
(c:/texlive/2024/texmf-dist/tex/generic/iftex/ifvtex.sty
Package: ifvtex 2019/10/25 v1.7 ifvtex legacy package. Use iftex instead.
)
\Gm@cnth=\count286
\Gm@cntv=\count287
\c@Gm@tempcnt=\count288
\Gm@bindingoffset=\dimen161
\Gm@wd@mp=\dimen162
\Gm@odd@mp=\dimen163
\Gm@even@mp=\dimen164
\Gm@layoutwidth=\dimen165
\Gm@layoutheight=\dimen166
\Gm@layouthoffset=\dimen167
\Gm@layoutvoffset=\dimen168
\Gm@dimlist=\toks35
) (c:/texlive/2024/texmf-dist/tex/latex/preprint/authblk.sty
Package: authblk 2001/02/27 1.3 (PWD)
\affilsep=\skip143
\@affilsep=\skip144
\c@Maxaffil=\count289
\c@authors=\count290
\c@affil=\count291
) (c:/texlive/2024/texmf-dist/tex/latex/footmisc/footmisc.sty
Package: footmisc 2023/07/05 v6.0f a miscellany of footnote facilities
\FN@temptoken=\toks36
\footnotemargin=\dimen169
\@outputbox@depth=\dimen170
Package footmisc Info: Declaring symbol style bringhurst on input line
696.
Package footmisc Info: Declaring symbol style chicago on input line 704.
Package footmisc Info: Declaring symbol style wiley on input line 713.
```

```
Package footmisc Info: Declaring symbol style lamport-robust on input
line 724.

Package footmisc Info: Declaring symbol style lamport* on input line 744.
Package footmisc Info: Declaring symbol style lamport*-robust on input
line 765
.
) (c:/texlive/2024/texmf-dist/tex/latex/fancyhdr/fancyhdr.sty
Package: fancyhdr 2024/07/23 v4.3.1 Extensive control of page headers and
foote
rs
\f@nch@headwidth=\skip145
\f@nch@O@elh=\skip146
\f@nch@O@erh=\skip147
\f@nch@O@olh=\skip148
\f@nch@O@orh=\skip149
\f@nch@O@elf=\skip150
\f@nch@O@erf=\skip151
\f@nch@O@olf=\skip152
\f@nch@O@orf=\skip153
) (c:/texlive/2024/texmf-dist/tex/generic/alphalph/alphalph.sty
Package: alphalph 2019/12/09 v2.6 Convert numbers to letters (HO)
(c:/texlive/2024/texmf-dist/tex/generic/intcalc/intcalc.sty
Package: intcalc 2019/12/15 v1.3 Expandable calculations with integers
(HO)
))
\c@authorfn=\count292
(c:/texlive/2024/texmf-dist/tex/latex/abstract/abstract.sty
Package: abstract 2009/06/08 v1.2a configurable abstracts
\abstitleskip=\skip154
\absleftindent=\skip155
\absrightindent=\skip156
\absparindent=\skip157
\absparsep=\skip158
)
Package newfloat Info: New float `keypoints' with options
`placement=t!,name=kp
t' on input line 291.
\c@keypoints=\count293
\newfloat@ftype=\count294
Package newfloat Info: float type `keypoints'=8 on input line 291.
(c:/texlive/2024/texmf-dist/tex/latex/enumitem/enumitem.sty
Package: enumitem 2019/06/20 v3.9 Customized lists
\labelindent=\skip159
\enit@outerparindent=\dimen171
\enit@toks=\toks37
\enit@inbox=\box67
\enit@count@id=\count295
\enitdp@description=\count296
) (c:/texlive/2024/texmf-dist/tex/latex/quoting/quoting.sty
Package: quoting 2014/01/28 v0.1c Consolidated environment for displayed
text
\quo@toppartop=\skip160
) (c:/texlive/2024/texmf-dist/tex/latex/sttools/stfloats.sty
```

```
Package: stfloats 2017/03/27 v3.3 Improve float mechanism and
baselineskip sett
ings
\@dblbotnum=\count297
\c@dblbotnumber=\count298
) (c:/texlive/2024/texmf-dist/tex/latex/booktabs/booktabs.sty
Package: booktabs 2020/01/12 v1.61803398 Publication quality tables
\heavyrulewidth=\dimen172
\lightrulewidth=\dimen173
\cmidrulewidth=\dimen174
\belowrulesep=\dimen175
\belowbottomsep=\dimen176
\aboverulesep=\dimen177
\abovetopsep=\dimen178
\cmidrulesep=\dimen179
\cmidrulekern=\dimen180
\defaultaddspace=\dimen181
\@cmidla=\count299
\@cmidlb=\count300
\@aboverulesep=\dimen182
\@belowrulesep=\dimen183
\@thisruleclass=\count301
\@lastruleclass=\count302
\@thisrulewidth=\dimen184
) (c:/texlive/2024/texmf-dist/tex/latex/tools/tabularx.sty
Package: tabularx 2023/12/11 v2.12a `tabularx' package (DPC)
\TX@col@width=\dimen185
\TX@old@table=\dimen186
\TX@old@col=\dimen187
\TX@target=\dimen188
\TX@delta=\dimen189
\TX@cols=\count303
\TX@ftn=\toks38
)
\enitdp@tablenotes=\count304
(c:/texlive/2024/texmf-dist/tex/latex/caption/caption.sty
Package: caption 2023/08/05 v3.6o Customizing captions (AR)
(c:/texlive/2024/texmf-dist/tex/latex/caption/caption3.sty
Package: caption3 2023/07/31 v2.4d caption3 kernel (AR)
\caption@tempdima=\dimen190
\captionmargin=\dimen191
\caption@leftmargin=\dimen192
\caption@rightmargin=\dimen193
\caption@width=\dimen194
\caption@indent=\dimen195
\caption@parindent=\dimen196
\caption@hangindent=\dimen197
Package caption Info: Standard document class detected.
)
\c@caption@flags=\count305
\c@continuedfloat=\count306
Package caption Info: rotating package is loaded.
Package caption Info: scrextend package is loaded.
\caption@addmargin@hsize=\dimen198
```

```
\caption@addmargin@linewidth=\dimen199
) (c:/texlive/2024/texmf-dist/tex/latex/natbib/natbib.sty
Package: natbib 2010/09/13 8.31b (PWD, AO)
\bibhang=\skip161
\bibsep=\skip162
LaTeX Info: Redefining \cite on input line 694.
\c@NAT@ctr=\count307
)) (c:/texlive/2024/texmf-dist/tex/latex/siunitx/siunitx.sty
Package: siunitx 2024-06-24 v3.3.19 A comprehensive (SI) units package
\l__siunitx_number_uncert_offset_int=\count308
\l__siunitx_number_exponent_fixed_int=\count309
\l__siunitx_number_min_decimal_int=\count310
\l__siunitx_number_min_integer_int=\count311
\l__siunitx_number_round_precision_int=\count312
\l__siunitx_number_lower_threshold_int=\count313
\l__siunitx_number_upper_threshold_int=\count314
\l__siunitx_number_group_first_int=\count315
\l__siunitx_number_group_size_int=\count316
\l__siunitx_number_group_minimum_int=\count317
\l__siunitx_angle_tmp_dim=\dimen256
\l__siunitx_angle_marker_box=\box68
\l__siunitx_angle_unit_box=\box69
\l__siunitx_compound_count_int=\count318
(c:/texlive/2024/texmf-dist/tex/latex/translations/translations.sty
Package: translations 2022/02/05 v1.12 internationalization of LaTeX2e
packages
 (CN)
) (c:/texlive/2024/texmf-dist/tex/latex/amsmath/amstext.sty
Package: amstext 2021/08/26 v2.01 AMS text
(c:/texlive/2024/texmf-dist/tex/latex/amsmath/amsgen.sty
File: amsgen.sty 1999/11/30 v2.0 generic functions
\@emptytoks=\toks39
\ex@=\dimen257
))
\l__siunitx_table_tmp_box=\box70
\l__siunitx_table_tmp_dim=\dimen258
\l__siunitx_table_column_width_dim=\dimen259
\l__siunitx_table_integer_box=\box71
\l__siunitx_table_decimal_box=\box72
\l__siunitx_table_uncert_box=\box73
\l__siunitx_table_before_box=\box74
\l__siunitx_table_after_box=\box75
\l__siunitx_table_before_dim=\dimen260
\l__siunitx_table_carry_dim=\dimen261
\l__siunitx_unit_tmp_int=\count319
\l__siunitx_unit_position_int=\count320
\l__siunitx_unit_total_int=\count321
) (c:/texlive/2024/texmf-dist/tex/latex/tools/xspace.sty
Package: xspace 2014/10/28 v1.13 Space after command names (DPC,MH)
) (c:/texlive/2024/texmf-dist/tex/latex/setspace/setspace.sty
Package: setspace 2022/12/04 v6.7b set line spacing
) (c:/texlive/2024/texmf-dist/tex/latex/lineno/lineno.sty
Package: lineno 2023/05/20 line numbers on paragraphs v5.3
\linenopenalty=\count322
```

```
\output=\toks40
\linenoprevgraf=\count323
\linenumbersep=\dimen262
\linenumberwidth=\dimen263
\c@linenumber=\count324
\c@pagewiselinenumber=\count325
\c@LN@truepage=\count326
\c@internallinenumber=\count327
\c@internallinenumbers=\count328
\quotelinenumbersep=\dimen264
\bframerule=\dimen265
\bframesep=\dimen266
\bframebox=\box76
LaTeX Info: Redefining \\ on input line 3180.
) (c:/texlive/2024/texmf-dist/tex/latex/hyperref/hyperref.sty
Package: hyperref 2024-07-10 v7.01j Hypertext links for LaTeX
(c:/texlive/2024/texmf-dist/tex/generic/pdfescape/pdfescape.sty
Package: pdfescape 2019/12/09 v1.15 Implements pdfTeX's escape features
(HO)
) (c:/texlive/2024/texmf-dist/tex/latex/hycolor/hycolor.sty
Package: hycolor 2020-01-27 v1.10 Color options for hyperref/bookmark
(HO)
) (c:/texlive/2024/texmf-dist/tex/latex/hyperref/nameref.sty
Package: nameref 2023-11-26 v2.56 Cross-referencing by name of section
(c:/texlive/2024/texmf-dist/tex/latex/refcount/refcount.sty
Package: refcount 2019/12/15 v3.6 Data extraction from label references
(HO)
) (c:/texlive/2024/texmf-
dist/tex/generic/gettitlestring/gettitlestring.sty
Package: gettitlestring 2019/12/15 v1.6 Cleanup title references (HO)
)
\c@section@level=\count329
) (c:/texlive/2024/texmf-dist/tex/generic/stringenc/stringenc.sty
Package: stringenc 2019/11/29 v1.12 Convert strings between diff.
encodings (HO
)
)
\@linkdim=\dimen267
\Hy@linkcounter=\count330
\Hy@pagecounter=\count331
(c:/texlive/2024/texmf-dist/tex/latex/hyperref/pd1enc.def
File: pd1enc.def 2024-07-10 v7.01j Hyperref: PDFDocEncoding definition
(HO)
Now handling font encoding PD1 ...
... no UTF-8 mapping file for font encoding PD1
)
\Hy@SavedSpaceFactor=\count332
(c:/texlive/2024/texmf-dist/tex/latex/hyperref/puenc.def
File: puenc.def 2024-07-10 v7.01j Hyperref: PDF Unicode definition (HO)
Now handling font encoding PU ...
... no UTF-8 mapping file for font encoding PU
)
Package hyperref Info: Option `colorlinks' set `true' on input line 4040.
Package hyperref Info: Hyper figures OFF on input line 4157.
```

```
Package hyperref Info: Link nesting OFF on input line 4162.
Package hyperref Info: Hyper index ON on input line 4165.
Package hyperref Info: Plain pages OFF on input line 4172.
Package hyperref Info: Backreferencing OFF on input line 4177.
Package hyperref Info: Implicit mode ON; LaTeX internals redefined.
Package hyperref Info: Bookmarks ON on input line 4424.
\c@Hy@tempcnt=\count333
LaTeX Info: Redefining \url on input line 4763.
\XeTeXLinkMargin=\dimen268
(c:/texlive/2024/texmf-dist/tex/generic/bitset/bitset.sty
Package: bitset 2019/12/09 v1.3 Handle bit-vector datatype (HO)
(c:/texlive/2024/texmf-dist/tex/generic/bigintcalc/bigintcalc.sty
Package: bigintcalc 2019/12/15 v1.5 Expandable calculations on big
integers (HO
)
))
\Fld@menulength=\count334
\Field@Width=\dimen269
\Fld@charsize=\dimen270
Package hyperref Info: Hyper figures OFF on input line 6042.
Package hyperref Info: Link nesting OFF on input line 6047.
Package hyperref Info: Hyper index ON on input line 6050.
Package hyperref Info: backreferencing OFF on input line 6057.
Package hyperref Info: Link coloring ON on input line 6060.
Package hyperref Info: Link coloring with OCG OFF on input line 6067.
Package hyperref Info: PDF/A mode OFF on input line 6072.
(c:/texlive/2024/texmf-dist/tex/latex/base/atbegshi-ltx.sty
Package: atbegshi-ltx 2021/01/10 v1.0c Emulation of the original atbegshi
package with kernel methods
)
\Hy@abspage=\count335
\c@Item=\count336
\c@Hfootnote=\count337
)
Package hyperref Info: Driver (autodetected): hpdftex.
(c:/texlive/2024/texmf-dist/tex/latex/hyperref/hpdftex.def
File: hpdftex.def 2024-07-10 v7.01j Hyperref driver for pdfTeX
(c:/texlive/2024/texmf-dist/tex/latex/base/atveryend-ltx.sty
Package: atveryend-ltx 2020/08/19 v1.0a Emulation of the original
atveryend pac
kage
with kernel methods
)
\HyAnn@Count=\count338
\Fld@listcount=\count339
\c@bookmark@seq@number=\count340
(c:/texlive/2024/texmf-dist/tex/latex/rerunfilecheck/rerunfilecheck.sty
Package: rerunfilecheck 2022-07-10 v1.10 Rerun checks for auxiliary files
(HO)
(c:/texlive/2024/texmf-dist/tex/generic/uniquecounter/uniquecounter.sty
Package: uniquecounter 2019/12/15 v1.4 Provide unlimited unique counter
(HO)
)
```

```
Package uniquecounter Info: New unique counter `rerunfilecheck' on input
line 2
85.
)
\Hy@SectionHShift=\skip163
)
Package translations Info: No language package found. I am going to use
`englis
h' as default language. on input line 92.
LaTeX Font Info:    Trying to load font information for T1+Merriwthr-OsF
on inp
ut line 92.
(c:/texlive/2024/texmf-dist/tex/latex/merriweather/T1Merriwthr-OsF.fd
File: T1Merriwthr-OsF.fd 2020/08/30 (autoinst) Font definitions for
T1/Merriwth
r-OsF.
)
LaTeX Font Info:    Font shape `T1/Merriwthr-OsF/m/n' will be
(Font)              scaled to size 7.5pt on input line 92.
(./main.aux)
\openout1 = `main.aux'.

LaTeX Font Info:    Checking defaults for OML/cmm/m/it on input line 92.
LaTeX Font Info:    ... okay on input line 92.
LaTeX Font Info:    Checking defaults for OMS/cmsy/m/n on input line 92.
LaTeX Font Info:    ... okay on input line 92.
LaTeX Font Info:    Checking defaults for OT1/cmr/m/n on input line 92.
LaTeX Font Info:    ... okay on input line 92.
LaTeX Font Info:    Checking defaults for T1/cmr/m/n on input line 92.
LaTeX Font Info:    ... okay on input line 92.
LaTeX Font Info:    Checking defaults for TS1/cmr/m/n on input line 92.
LaTeX Font Info:    ... okay on input line 92.
LaTeX Font Info:    Checking defaults for OMX/cmex/m/n on input line 92.
LaTeX Font Info:    ... okay on input line 92.
LaTeX Font Info:    Checking defaults for U/cmr/m/n on input line 92.
LaTeX Font Info:    ... okay on input line 92.
LaTeX Font Info:    Checking defaults for PD1/pdf/m/n on input line 92.
LaTeX Font Info:    ... okay on input line 92.
LaTeX Font Info:    Checking defaults for PU/pdf/m/n on input line 92.
LaTeX Font Info:    ... okay on input line 92.
LaTeX Info: Redefining \microtypecontext on input line 92.
Package microtype Info: Applying patch `item' on input line 92.
Package microtype Info: Applying patch `toc' on input line 92.
Package microtype Info: Applying patch `eqnum' on input line 92.
Package microtype Info: Applying patch `footnote' on input line 92.
Package microtype Info: Applying patch `verbatim' on input line 92.
Package microtype Info: Generating PDF output.
Package microtype Info: Character protrusion enabled (level 2).
Package microtype Info: Using default protrusion set `alltext'.
Package microtype Info: Automatic font expansion enabled (level 2),
(microtype)             stretch: 20, shrink: 20, step: 1, non-selected.
Package microtype Info: Using default expansion set `alltext-nott'.
LaTeX Info: Redefining \showhyphens on input line 92.
Package microtype Info: No adjustment of tracking.
```

```
Package microtype Info: No adjustment of interword spacing.
Package microtype Info: No adjustment of character kerning.
Package microtype Info: Loading generic protrusion settings for font
family
(microtype)             `Merriwthr-OsF' (encoding: T1).
(microtype)             For optimal results, create family-specific
settings.
(microtype)             See the microtype manual for details.
LaTeX Font Info:    Redeclaring symbol font `operators' on input line 92.
LaTeX Font Info:    Encoding `OT1' has changed to `T1' for symbol font
(Font)              `operators' in the math version `normal' on input
line 92.
LaTeX Font Info:    Overwriting symbol font `operators' in version
`normal'
(Font)                  OT1/cmr/m/n --> T1/Merriwthr-OsF/m/up on input
line 92.

LaTeX Font Info:    Encoding `OT1' has changed to `T1' for symbol font
(Font)              `operators' in the math version `bold' on input line
92.
LaTeX Font Info:    Overwriting symbol font `operators' in version `bold'
(Font)                  OT1/cmr/bx/n --> T1/Merriwthr-OsF/m/up on input
line 92
.
LaTeX Font Info:    Overwriting symbol font `operators' in version `bold'
(Font)                  T1/Merriwthr-OsF/m/up --> T1/Merriwthr-OsF/b/up
on inpu
t line 92.
LaTeX Font Info:    Redeclaring math alphabet \mathbf on input line 92.
LaTeX Font Info:    Overwriting math alphabet `\mathbf' in version
`normal'
(Font)                  OT1/cmr/bx/n --> T1/Merriwthr-OsF/b/up on input
line 92
.
LaTeX Font Info:    Overwriting math alphabet `\mathbf' in version `bold'
(Font)                  OT1/cmr/bx/n --> T1/Merriwthr-OsF/b/up on input
line 92
.
LaTeX Font Info:    Redeclaring math alphabet \mathsf on input line 92.
LaTeX Font Info:    Overwriting math alphabet `\mathsf' in version
`normal'
(Font)                  OT1/cmss/m/n --> T1/MerriwthrSans-OsF/m/up on
input lin
e 92.
LaTeX Font Info:    Overwriting math alphabet `\mathsf' in version `bold'
(Font)                  OT1/cmss/bx/n --> T1/MerriwthrSans-OsF/m/up on
input li
ne 92.
LaTeX Font Info:    Redeclaring math alphabet \mathit on input line 92.
LaTeX Font Info:    Overwriting math alphabet `\mathit' in version
`normal'
(Font)                  OT1/cmr/m/it --> T1/Merriwthr-OsF/m/it on input
line 92
.
```

```
LaTeX Font Info:    Overwriting math alphabet `\mathit' in version `bold'
(Font)                  OT1/cmr/bx/it --> T1/Merriwthr-OsF/m/it on input
line 9
2.
LaTeX Font Info:    Redeclaring math alphabet \mathtt on input line 92.
LaTeX Font Info:    Overwriting math alphabet `\mathtt' in version
`normal'
(Font)                  OT1/cmtt/m/n --> T1/lmtt/m/up on input line 92.
LaTeX Font Info:    Overwriting math alphabet `\mathtt' in version `bold'
(Font)                  OT1/cmtt/m/n --> T1/lmtt/m/up on input line 92.
LaTeX Font Info:    Overwriting math alphabet `\mathsf' in version `bold'
(Font)                  T1/MerriwthrSans-OsF/m/up --> T1/MerriwthrSans-
OsF/b/up
 on input line 92.
LaTeX Font Info:    Overwriting math alphabet `\mathit' in version `bold'
(Font)                  T1/Merriwthr-OsF/m/it --> T1/Merriwthr-OsF/b/it
on inpu
t line 92.
\c@mv@tabular=\count341
\c@mv@boldtabular=\count342
(c:/texlive/2024/texmf-dist/tex/context/base/mkii/supp-pdf.mkii
[Loading MPS to PDF converter (version 2006.09.02).]
\scratchcounter=\count343
\scratchdimen=\dimen271
\scratchbox=\box77
\nofMPsegments=\count344
\nofMParguments=\count345
\everyMPshowfont=\toks41
\MPscratchCnt=\count346
\MPscratchDim=\dimen272
\MPnumerator=\count347
\makeMPintoPDFobject=\count348
\everyMPtoPDFconversion=\toks42
) (c:/texlive/2024/texmf-dist/tex/latex/epstopdf-pkg/epstopdf-base.sty
Package: epstopdf-base 2020-01-24 v2.11 Base part for package epstopdf
Package epstopdf-base Info: Redefining graphics rule for `.eps' on input
line 4
85.
(c:/texlive/2024/texmf-dist/tex/latex/latexconfig/epstopdf-sys.cfg
File: epstopdf-sys.cfg 2010/07/13 v1.3 Configuration of (r)epstopdf for
TeX Liv
e
))
*geometry* driver: auto-detecting
*geometry* detected driver: pdftex
*geometry* verbose mode - [ preamble ] result:
* driver: pdftex
* paper: a4paper
* layout: <same size as paper>
* layoutoffset:(h,v)=(0.0pt,0.0pt)
* modes: includefoot twoside
* h-part:(L,W,R)=(54.64pt, 488.22787pt, 54.64pt)
* v-part:(T,H,B)=(66.0pt, 745.04684pt, 34.0pt)
* \paperwidth=597.50787pt
```

```
* \paperheight=845.04684pt
* \textwidth=488.22787pt
* \textheight=715.04684pt
* \oddsidemargin=-17.62999pt
* \evensidemargin=-17.62999pt
* \topmargin=-47.76999pt
* \headheight=17.5pt
* \headsep=24.0pt
* \topskip=10.0pt
* \footskip=30.0pt
* \marginparwidth=48.0pt
* \marginparsep=10.0pt
* \columnsep=18.0pt
* \skip\footins=22.0pt plus 2.0pt
* \hoffset=0.0pt
* \voffset=0.0pt
* \mag=1000
* \@twocolumntrue
* \@twosidetrue
* \@mparswitchtrue
* \@reversemarginfalse
* (1in=72.27pt=25.4mm, 1cm=28.453pt)

Package caption Info: Begin \AtBeginDocument code.
Package caption Info: hyperref package is loaded.
Package caption Info: End \AtBeginDocument code.

(c:/texlive/2024/texmf-dist/tex/latex/translations/translations-basic-
dictionar
y-english.trsl
File: translations-basic-dictionary-english.trsl (english translation
file `tra
nslations-basic-dictionary')
)
Package translations Info: loading dictionary `translations-basic-
dictionary' f
or `english'. on input line 92.
Package hyperref Info: Link coloring ON on input line 92.
(./main.out) (./main.out)
\@outlinefile=\write3
\openout3 = `main.out'.

\@gscitedetails=\box78
\@gscitedetailsheight=\skip164
\@gsheadbox=\box79
\@gsheadboxheight=\skip165
LaTeX Font Info:    Font shape `T1/Merriwthr-OsF/b/n' will be
(Font)              scaled to size 6.5pt on input line 92.
LaTeX Font Info:    Calculating math sizes for size <7.5> on input line
92.

LaTeX Font Warning: Font shape `T1/Merriwthr-OsF/m/up' undefined
(Font)              using `T1/Merriwthr-OsF/m/n' instead on input line
92.
```

```
LaTeX Font Info:    Font shape `T1/Merriwthr-OsF/m/up' will be
(Font)              scaled to size 6.24973pt on input line 92.
LaTeX Font Info:    Font shape `T1/Merriwthr-OsF/m/up' will be
(Font)              scaled to size 5.24997pt on input line 92.
LaTeX Font Info:    Trying to load font information for U+eur on input
line 92.

(c:/texlive/2024/texmf-dist/tex/latex/amsfonts/ueur.fd
File: ueur.fd 2013/01/14 v3.01 Euler Roman
) (c:/texlive/2024/texmf-dist/tex/latex/microtype/mt-eur.cfg
File: mt-eur.cfg 2006/07/31 v1.1 microtype config. file: AMS Euler Roman
(RS)
)

LaTeX Font Warning: Font shape `OMS/cmsy/m/n' in size <7.5> not available
(Font)              size <7> substituted on input line 92.

LaTeX Font Info:    External font `cmex10' loaded for size
(Font)              <7.5> on input line 92.
LaTeX Font Info:    External font `cmex10' loaded for size
(Font)              <6.24973> on input line 92.
LaTeX Font Info:    External font `cmex10' loaded for size
(Font)              <5.24997> on input line 92.
LaTeX Font Info:    Trying to load font information for U+euf on input
line 92.

(c:/texlive/2024/texmf-dist/tex/latex/amsfonts/ueuf.fd
File: ueuf.fd 2013/01/14 v3.01 Euler Fraktur
) (c:/texlive/2024/texmf-dist/tex/latex/microtype/mt-euf.cfg
File: mt-euf.cfg 2006/07/03 v1.1 microtype config. file: AMS Euler
Fraktur (RS)

)
LaTeX Font Info:    Trying to load font information for U+eus on input
line 92.

(c:/texlive/2024/texmf-dist/tex/latex/amsfonts/ueus.fd
File: ueus.fd 2013/01/14 v3.01 Euler Script
) (c:/texlive/2024/texmf-dist/tex/latex/microtype/mt-eus.cfg
File: mt-eus.cfg 2006/07/28 v1.2 microtype config. file: AMS Euler Script
(RS)
)
LaTeX Font Info:    Trying to load font information for U+euex on input
line 92
.
(c:/texlive/2024/texmf-dist/tex/latex/amsfonts/ueuex.fd
File: ueuex.fd 2013/01/14 v3.01 Euler extra symbols
)

LaTeX Font Warning: Font shape `OML/cmm/m/it' in size <7.5> not available
(Font)              size <7> substituted on input line 92.

LaTeX Font Info:    Font shape `T1/Merriwthr-OsF/m/n' will be
```

```
(Font)              scaled to size 6.24973pt on input line 92.
LaTeX Font Info:    Font shape `T1/Merriwthr-OsF/m/n' will be
(Font)              scaled to size 5.24997pt on input line 92.
LaTeX Font Info:    Font shape `T1/Merriwthr-OsF/m/it' will be
(Font)              scaled to size 7.5pt on input line 92.
LaTeX Font Info:    Font shape `T1/Merriwthr-OsF/m/it' will be
(Font)              scaled to size 6.24973pt on input line 92.
LaTeX Font Info:    Font shape `T1/Merriwthr-OsF/m/it' will be
(Font)              scaled to size 5.24997pt on input line 92.
LaTeX Font Info:    Font shape `T1/Merriwthr-OsF/m/n' will be
(Font)              scaled to size 8.0pt on input line 92.
LaTeX Font Info:    Font shape `T1/Merriwthr-OsF/m/it' will be
(Font)              scaled to size 8.0pt on input line 92.
LaTeX Font Info:    Font shape `T1/Merriwthr-OsF/b/it' will be
(Font)              scaled to size 8.0pt on input line 92.
TextBlockOrigin set to 4pc+6.64pt x 4pc+6pt
<oup.pdf, id=93, 597.50829pt x 845.0471pt>
File: oup.pdf Graphic file (type pdf)
<use oup.pdf>
Package pdftex.def Info: oup.pdf  used on input line 104.
(pdftex.def)             Requested size: 41.03665pt x 58.038pt.
<gigascience-logo.pdf, id=94, 99.37125pt x 33.12375pt>
File: gigascience-logo.pdf Graphic file (type pdf)
<use gigascience-logo.pdf>
Package pdftex.def Info: gigascience-logo.pdf  used on input line 104.
(pdftex.def)             Requested size: 126.00902pt x 42.0pt.

Overfull \hbox (54.64pt too wide) in paragraph at lines 104--104
[][]
 []

LaTeX Font Info:    Font shape `T1/Merriwthr-OsF/m/n' will be
(Font)              scaled to size 14.0pt on input line 104.
LaTeX Font Info:    Font shape `T1/Merriwthr-OsF/m/n' will be
(Font)              scaled to size 8.99997pt on input line 104.
LaTeX Font Info:    Calculating math sizes for size <14> on input line
104.
LaTeX Font Info:    Font shape `T1/Merriwthr-OsF/m/up' will be
(Font)              scaled to size 14.0pt on input line 104.
LaTeX Font Info:    Font shape `T1/Merriwthr-OsF/m/up' will be
(Font)              scaled to size 11.66617pt on input line 104.
LaTeX Font Info:    Font shape `T1/Merriwthr-OsF/m/up' will be
(Font)              scaled to size 9.79996pt on input line 104.
LaTeX Font Info:    External font `cmex10' loaded for size
(Font)              <14> on input line 104.
LaTeX Font Info:    External font `cmex10' loaded for size
(Font)              <11.66617> on input line 104.
LaTeX Font Info:    External font `cmex10' loaded for size
(Font)              <9.79996> on input line 104.
LaTeX Font Info:    Font shape `T1/Merriwthr-OsF/m/n' will be
(Font)              scaled to size 11.66617pt on input line 104.
LaTeX Font Info:    Font shape `T1/Merriwthr-OsF/m/n' will be
(Font)              scaled to size 9.79996pt on input line 104.
LaTeX Font Info:    Font shape `T1/Merriwthr-OsF/m/it' will be
```

```
(Font)              scaled to size 14.0pt on input line 104.
LaTeX Font Info:    Font shape `T1/Merriwthr-OsF/m/it' will be
(Font)              scaled to size 11.66617pt on input line 104.
LaTeX Font Info:    Font shape `T1/Merriwthr-OsF/m/it' will be
(Font)              scaled to size 9.79996pt on input line 104.
LaTeX Font Info:    Font shape `T1/Merriwthr-OsF/b/n' will be
(Font)              scaled to size 18.0pt on input line 104.
LaTeX Font Info:    Font shape `T1/Merriwthr-OsF/m/n' will be
(Font)              scaled to size 13.0pt on input line 104.
LaTeX Font Info:    Calculating math sizes for size <13> on input line
104.
LaTeX Font Info:    Font shape `T1/Merriwthr-OsF/m/up' will be
(Font)              scaled to size 13.0pt on input line 104.
LaTeX Font Info:    Font shape `T1/Merriwthr-OsF/m/up' will be
(Font)              scaled to size 10.83287pt on input line 104.
LaTeX Font Info:    Font shape `T1/Merriwthr-OsF/m/up' will be
(Font)              scaled to size 9.09996pt on input line 104.

LaTeX Font Warning: Font shape `OMS/cmsy/m/n' in size <13> not available
(Font)              size <12> substituted on input line 104.

LaTeX Font Info:    External font `cmex10' loaded for size
(Font)              <13> on input line 104.
LaTeX Font Info:    External font `cmex10' loaded for size
(Font)              <10.83287> on input line 104.
LaTeX Font Info:    External font `cmex10' loaded for size
(Font)              <9.09996> on input line 104.

LaTeX Font Warning: Font shape `OML/cmm/m/it' in size <13> not available
(Font)              size <12> substituted on input line 104.

LaTeX Font Info:    Font shape `T1/Merriwthr-OsF/m/n' will be
(Font)              scaled to size 10.83287pt on input line 104.
LaTeX Font Info:    Font shape `T1/Merriwthr-OsF/m/n' will be
(Font)              scaled to size 9.09996pt on input line 104.
LaTeX Font Info:    Font shape `T1/Merriwthr-OsF/m/it' will be
(Font)              scaled to size 13.0pt on input line 104.
LaTeX Font Info:    Font shape `T1/Merriwthr-OsF/m/it' will be
(Font)              scaled to size 10.83287pt on input line 104.
LaTeX Font Info:    Font shape `T1/Merriwthr-OsF/m/it' will be
(Font)              scaled to size 9.09996pt on input line 104.
LaTeX Font Info:    Trying to load font information for TS1+Merriwthr-OsF
on in
put line 104.
(c:/texlive/2024/texmf-dist/tex/latex/merriweather/TS1Merriwthr-OsF.fd
File: TS1Merriwthr-OsF.fd 2020/08/30 (autoinst) Font definitions for
TS1/Merriw
thr-OsF.
)
LaTeX Font Info:    Font shape `TS1/Merriwthr-OsF/m/n' will be
(Font)              scaled to size 10.83287pt on input line 104.
Package microtype Info: Loading generic protrusion settings for font
family
(microtype)             `Merriwthr-OsF' (encoding: TS1).
```

```
(microtype)             For optimal results, create family-specific
settings.
(microtype)             See the microtype manual for details.
LaTeX Font Info:    Font shape `T1/Merriwthr-OsF/m/n' will be
(Font)              scaled to size 9.0pt on input line 104.
LaTeX Font Info:    Font shape `T1/Merriwthr-OsF/m/up' will be
(Font)              scaled to size 9.0pt on input line 104.
LaTeX Font Info:    Font shape `T1/Merriwthr-OsF/m/up' will be
(Font)              scaled to size 7.0pt on input line 104.
LaTeX Font Info:    Font shape `T1/Merriwthr-OsF/m/up' will be
(Font)              scaled to size 5.0pt on input line 104.
LaTeX Font Info:    External font `cmex10' loaded for size
(Font)              <9> on input line 104.
LaTeX Font Info:    External font `cmex10' loaded for size
(Font)              <7> on input line 104.
LaTeX Font Info:    External font `cmex10' loaded for size
(Font)              <5> on input line 104.
LaTeX Font Info:    Font shape `T1/Merriwthr-OsF/m/n' will be
(Font)              scaled to size 7.0pt on input line 104.
LaTeX Font Info:    Font shape `T1/Merriwthr-OsF/m/n' will be
(Font)              scaled to size 5.0pt on input line 104.
LaTeX Font Info:    Font shape `T1/Merriwthr-OsF/m/it' will be
(Font)              scaled to size 9.0pt on input line 104.
LaTeX Font Info:    Font shape `T1/Merriwthr-OsF/m/it' will be
(Font)              scaled to size 7.0pt on input line 104.
LaTeX Font Info:    Font shape `T1/Merriwthr-OsF/m/it' will be
(Font)              scaled to size 5.0pt on input line 104.
LaTeX Font Info:    Font shape `T1/Merriwthr-OsF/m/n' will be
(Font)              scaled to size 6.5pt on input line 104.
LaTeX Font Info:    Calculating math sizes for size <6.5> on input line
104.
LaTeX Font Info:    Font shape `T1/Merriwthr-OsF/m/up' will be
(Font)              scaled to size 6.5pt on input line 104.
LaTeX Font Info:    Font shape `T1/Merriwthr-OsF/m/up' will be
(Font)              scaled to size 5.41643pt on input line 104.
LaTeX Font Info:    Font shape `T1/Merriwthr-OsF/m/up' will be
(Font)              scaled to size 4.54997pt on input line 104.

LaTeX Font Warning: Font shape `OMS/cmsy/m/n' in size <6.5> not available
(Font)              size <6> substituted on input line 104.


LaTeX Font Warning: Font shape `OMS/cmsy/m/n' in size <5.41643> not
available
(Font)              size <5> substituted on input line 104.


LaTeX Font Warning: Font shape `OMS/cmsy/m/n' in size <4.54997> not
available
(Font)              size <5> substituted on input line 104.

LaTeX Font Info:    External font `cmex10' loaded for size
(Font)              <6.5> on input line 104.
LaTeX Font Info:    External font `cmex10' loaded for size
```

```
(Font)              <5.41643> on input line 104.
LaTeX Font Info:    External font `cmex10' loaded for size
(Font)              <4.54997> on input line 104.

LaTeX Font Warning: Font shape `OML/cmm/m/it' in size <6.5> not available
(Font)              size <6> substituted on input line 104.


LaTeX Font Warning: Font shape `OML/cmm/m/it' in size <5.41643> not
available
(Font)              size <5> substituted on input line 104.


LaTeX Font Warning: Font shape `OML/cmm/m/it' in size <4.54997> not
available
(Font)              size <5> substituted on input line 104.

LaTeX Font Info:    Font shape `T1/Merriwthr-OsF/m/n' will be
(Font)              scaled to size 5.41643pt on input line 104.
LaTeX Font Info:    Font shape `T1/Merriwthr-OsF/m/n' will be
(Font)              scaled to size 4.54997pt on input line 104.
LaTeX Font Info:    Font shape `T1/Merriwthr-OsF/m/it' will be
(Font)              scaled to size 6.5pt on input line 104.
LaTeX Font Info:    Font shape `T1/Merriwthr-OsF/m/it' will be
(Font)              scaled to size 5.41643pt on input line 104.
LaTeX Font Info:    Font shape `T1/Merriwthr-OsF/m/it' will be
(Font)              scaled to size 4.54997pt on input line 104.
LaTeX Font Info:    Font shape `TS1/Merriwthr-OsF/m/n' will be
(Font)              scaled to size 5.41643pt on input line 104.

Overfull \hbox (54.64pt too wide) in paragraph at lines 104--104
[][][]
 []

LaTeX Font Info:    Font shape `T1/Merriwthr-OsF/b/n' will be
(Font)              scaled to size 10.0pt on input line 104.
LaTeX Font Info:    Font shape `T1/Merriwthr-OsF/b/n' will be
(Font)              scaled to size 8.0pt on input line 104.

Overfull \hbox (54.64pt too wide) in paragraph at lines 104--104
[][][]
 []


LaTeX Warning: Optional argument of \twocolumn too tall on page 1.


Overfull \vbox (27.50037pt too high) has occurred while \output is active
[]




LaTeX Warning: Text page 1 contains only floats.
```

```
Overfull \vbox (27.50037pt too high) has occurred while \output is active
[]



LaTeX Font Info:    Font shape `T1/Merriwthr-OsF/m/n' will be
(Font)              scaled to size 7.8pt on input line 104.
LaTeX Font Info:    Font shape `T1/Merriwthr-OsF/b/n' will be
(Font)              scaled to size 7.8pt on input line 104.
[1{c:/texlive/2024/texmf-
var/fonts/map/pdftex/updmap/pdftex.map}{c:/texlive/202
4/texmf-
dist/fonts/enc/dvips/merriweather/merriwthr_posqbl.enc}{c:/texlive/2024
/texmf-dist/fonts/enc/dvips/merriweather/merriwthr_owzwzj.enc}


 <./oup.pdf> <./gigascience-logo.pdf>]
LaTeX Font Info:    Font shape `T1/Merriwthr-OsF/m/n' will be
(Font)              scaled to size 10.0pt on input line 106.
LaTeX Font Info:    Font shape `T1/Merriwthr-OsF/m/n' will be
(Font)              scaled to size 3.75pt on input line 106.
LaTeX Font Info:    Trying to load font information for T1+MerriwthrSans-
OsF on
 input line 106.
(c:/texlive/2024/texmf-dist/tex/latex/merriweather/T1MerriwthrSans-OsF.fd
File: T1MerriwthrSans-OsF.fd 2020/08/30 (autoinst) Font definitions for
T1/Merr
iwthrSans-OsF.
)
LaTeX Font Info:    Font shape `T1/MerriwthrSans-OsF/m/n' will be
(Font)              scaled to size 3.75pt on input line 106.
Package microtype Info: Loading generic protrusion settings for font
family
(microtype)             `MerriwthrSans-OsF' (encoding: T1).
(microtype)             For optimal results, create family-specific
settings.
(microtype)             See the microtype manual for details.
LaTeX Font Info:    Font shape `T1/Merriwthr-OsF/b/n' will be
(Font)              scaled to size 7.5pt on input line 109.

Package natbib Warning: Citation `De_Schotten2022-bl' on page 2 undefined
on in
put line 109.


Package natbib Warning: Citation `Fields2008-ui' on page 2 undefined on
input l
ine 109.


Package natbib Warning: Citation `Passingham2002-zu' on page 2 undefined
on inp
```

ut line 109.

Package natbib Warning: Citation `Wandell2016-gh' on page 2 undefined on input
line 109.

Package natbib Warning: Citation `Jbabdi2015-hz' on page 2 undefined on input l
ine 109.

Package natbib Warning: Citation `Schmahmann2006-gh' on page 2 undefined on inp
ut line 109.

Package natbib Warning: Citation `Axer2016-np' on page 2 undefined on input lin
e 109.

Package natbib Warning: Citation `Lefebvre2018-pb' on page 2 undefined on input
 line 109.

Package natbib Warning: Citation `Menzel2023-tm' on page 2 undefined on input l
ine 109.

Package natbib Warning: Citation `Mollink2017-uz' on page 2 undefined on input
line 109.

Package natbib Warning: Citation `Schurr2021-fx' on page 2 undefined on input l
ine 109.

Package natbib Warning: Citation `Wang2015-so' on page 2 undefined on input lin
e 109.

Package natbib Warning: Citation `Xu2021-yx' on page 2 undefined on input line
109.

```
Package natbib Warning: Citation `Menzel2023-tm' on page 2 undefined on
input l
ine 109.


Package natbib Warning: Citation `Kjer2025-pz' on page 2 undefined on
input lin
e 109.


Package natbib Warning: Citation `Girard2023-fn' on page 2 undefined on
input l
ine 109.


Package natbib Warning: Citation `Maffei2022-ya' on page 2 undefined on
input l
ine 109.


Package natbib Warning: Citation `Maier-Hein2017-zf' on page 2 undefined
on inp
ut line 109.


Package natbib Warning: Citation `Schilling2022-rm' on page 2 undefined
on inpu
t line 109.


Package natbib Warning: Citation `Rheault2020-im' on page 2 undefined on
input
line 109.


Package natbib Warning: Citation `Schilling2021-ww' on page 2 undefined
on inpu
t line 109.


Package natbib Warning: Citation `Casey2018-oo' on page 2 undefined on
input li
ne 111.


Package natbib Warning: Citation `Cieslak2025-og' on page 2 undefined on
input
line 111.


Package natbib Warning: Citation `Alexander2017-ex' on page 2 undefined
on inpu
t line 111.
```

Package natbib Warning: Citation `Richie-Halford2022-wk' on page 2
undefined on
 input line 111.

Package natbib Warning: Citation `Alfaro-Almagro2018-vd' on page 2
undefined on
 input line 111.

Package natbib Warning: Citation `Wilkinson2016-gw' on page 2 undefined
on inpu
t line 113.

Package natbib Warning: Citation `ist' on page 2 undefined on input line
113.

Package natbib Warning: Citation `Jelescu2022-ta' on page 2 undefined on
input
line 113.

Package natbib Warning: Citation `Schilling2022-rm' on page 2 undefined
on inpu
t line 113.

Package natbib Warning: Citation `Schilling2024-qj' on page 2 undefined
on inpu
t line 113.

Package natbib Warning: Citation `dsg' on page 2 undefined on input line
113.

Package natbib Warning: Citation `ismrm' on page 2 undefined on input
line 113.

Package natbib Warning: Citation `Barba2018-up' on page 2 undefined on
input li
ne 121.

Package natbib Warning: Citation `Kruper2021-mj' on page 2 undefined on
input l
ine 121.

```
Package natbib Warning: Citation `turingway' on page 2 undefined on input
line
121.


LaTeX Warning: File `figures/figure_1_schematic_vertical_revised_v2.pdf'
not fo
und on input line 126.


! Package pdftex.def Error: File
`figures/figure_1_schematic_vertical_revised_v
2.pdf' not found: using draft setting.

See the pdftex.def package documentation for explanation.
Type  H <return>  for immediate help.
 ...

l.126 ...gure_1_schematic_vertical_revised_v2.pdf}

Try typing  <return>  to proceed.
If that doesn't work, type  X <return>  to quit.

LaTeX Font Info:    Trying to load font information for T1+lmtt on input
line 1
26.
(c:/texlive/2024/texmf-dist/tex/latex/lm/t1lmtt.fd
File: t1lmtt.fd 2015/05/01 v1.6.1 Font defs for Latin Modern
)
Package microtype Info: Loading generic protrusion settings for font
family
(microtype)             `lmtt' (encoding: T1).
(microtype)             For optimal results, create family-specific
settings.
(microtype)             See the microtype manual for details.
LaTeX Font Info:    Font shape `T1/Merriwthr-OsF/m/n' will be
(Font)              scaled to size 6.0pt on input line 127.
LaTeX Font Info:    Font shape `T1/Merriwthr-OsF/b/n' will be
(Font)              scaled to size 6.0pt on input line 127.
LaTeX Font Info:    Font shape `T1/Merriwthr-OsF/b/n' will be
(Font)              scaled to size 8.5pt on input line 131.
LaTeX Font Info:    Font shape `T1/Merriwthr-OsF/m/n' will be
(Font)              scaled to size 8.5pt on input line 131.

Package natbib Warning: Citation `Mustra2008-ko' on page 2 undefined on
input l
ine 134.


Package natbib Warning: Citation `bids' on page 2 undefined on input line
134.
```

```
Package natbib Warning: Citation `Gorgolewski2016-dw' on page 2 undefined
on in
put line 134.


Package natbib Warning: Citation `Gholam2021-ru' on page 2 undefined on
input l
ine 134.


Package natbib Warning: Citation `Pestilli2021' on page 2 undefined on
input li
ne 134.


Package natbib Warning: Citation `Cai2021-yf' on page 2 undefined on
input line
 136.


Package natbib Warning: Citation `Gajwani2023-dv' on page 2 undefined on
input
line 136.


Package natbib Warning: Citation `Schilling2021-vx' on page 2 undefined
on inpu
t line 136.


Package natbib Warning: Citation `Tong2019-kr' on page 2 undefined on
input lin
e 136.


Package natbib Warning: Citation `Warrington2025-lq' on page 2 undefined
on inp
ut line 136.



LaTeX Font Info:    Font shape `T1/Merriwthr-OsF/m/it' will be
(Font)              scaled to size 7.8pt on input line 137.
[2{c:/texlive/2024/texmf-
dist/fonts/enc/dvips/merriweather/merriwthr_ags7qn.enc
}]

Package natbib Warning: Citation `Pinto2020-yt' on page 3 undefined on
input li
ne 138.
```

```
Package natbib Warning: Citation `Moyer2020-yi' on page 3 undefined on
input li
ne 138.


Package natbib Warning: Citation `Ning2019-fp' on page 3 undefined on
input lin
e 138.


Package natbib Warning: Citation `Tax2019-pk' on page 3 undefined on
input line
 138.


Package natbib Warning: Citation `Warrington2023-gi' on page 3 undefined
on inp
ut line 138.


Package natbib Warning: Citation `Magdoom:Neuroimage:2023' on page 3
undefined
on input line 140.


Package natbib Warning: Citation `Yang:arxiv:2025' on page 3 undefined on
input
 line 142.


Package natbib Warning: Citation `hcp' on page 3 undefined on input line
144.


Package natbib Warning: Citation `abcd' on page 3 undefined on input line
144.


Package natbib Warning: Citation `hbcd' on page 3 undefined on input line
144.


Package natbib Warning: Citation `hcph' on page 3 undefined on input line
144.


Underfull \vbox (badness 2726) has occurred while \output is active []



Package natbib Warning: Citation `Esteban2017-xy' on page 3 undefined on
input
line 150.
```

```
Package natbib Warning: Citation `Bastiani2019-nr' on page 3 undefined on
input
 line 150.


Package natbib Warning: Citation `Hagen2024-tx' on page 3 undefined on
input li
ne 150.


Package natbib Warning: Citation `Cieslak2021-iu' on page 3 undefined on
input
line 150.


Package natbib Warning: Citation `Cirstian2024-zq' on page 3 undefined on
input
 line 150.


Package natbib Warning: Citation `Richie-Halford2022-wk' on page 3
undefined on
 input line 150.


Package natbib Warning: Citation `Rheault2020-im' on page 3 undefined on
input
line 150.


Package natbib Warning: Citation `Schilling2021-ww' on page 3 undefined
on inpu
t line 150.


Package natbib Warning: Citation `Smith2013-zk' on page 3 undefined on
input li
ne 152.


Package natbib Warning: Citation `Smith2015-ok' on page 3 undefined on
input li
ne 152.


Package natbib Warning: Citation `Daducci2015-fj' on page 3 undefined on
input
line 152.


Package natbib Warning: Citation `Schiavi2020-uz' on page 3 undefined on
input
```

```
line 152.


Package natbib Warning: Citation `Caiafa2017-pi' on page 3 undefined on
input l
ine 152.


Package natbib Warning: Citation `Pestilli2014-kx' on page 3 undefined on
input
 line 152.


Package natbib Warning: Citation `Astolfi2023-jt' on page 3 undefined on
input
line 152.


Package natbib Warning: Citation `Legarreta2021-pg' on page 3 undefined
on inpu
t line 152.


Package natbib Warning: Citation `Sarwar2021-tj' on page 3 undefined on
input l
ine 152.


Package natbib Warning: Citation `Sarwar2023-ur' on page 3 undefined on
input l
ine 152.


Package natbib Warning: Citation `Zalesky2020-fk' on page 3 undefined on
input
line 152.


Package natbib Warning: Citation `Laamoumi2025-pn' on page 3 undefined on
input
 line 154.


Package natbib Warning: Citation `openneuro' on page 3 undefined on input
line
154.


Package natbib Warning: Citation `zenodo' on page 3 undefined on input
line 154
.
```

```
Package natbib Warning: Citation `Fillard:Neuroimage:2011' on page 3
undefined
on input line 154.


Package natbib Warning: Citation `Maier-Hein2017-zf' on page 3 undefined
on inp
ut line 154.


Package natbib Warning: Citation `Girard:Neuroimage:2023' on page 3
undefined o
n input line 154.


Package natbib Warning: Citation `Maffei2022-ya' on page 3 undefined on
input l
ine 154.


Package natbib Warning: Citation `Glen2020-cb' on page 3 undefined on
input lin
e 159.



[3]
LaTeX Font Info:    Font shape `T1/Merriwthr-OsF/m/up' will be
(Font)              scaled to size 7.5pt on input line 163.

LaTeX Warning: File
`figures/ist_su_position_paper_manuscript_tractography_layo
ut2_planes_lowercase_panels.pdf' not found on input line 171.


! Package pdftex.def Error: File
`figures/ist_su_position_paper_manuscript_trac
tography_layout2_planes_lowercase_panels.pdf' not found: using draft
setting.

See the pdftex.def package documentation for explanation.
Type  H <return>  for immediate help.
 ...

l.171 ...aphy_layout2_planes_lowercase_panels.pdf}

Try typing  <return>  to proceed.
If that doesn't work, type  X <return>  to quit.

LaTeX Font Info:    Font shape `T1/Merriwthr-OsF/m/it' will be
(Font)              scaled to size 6.0pt on input line 172.

Package natbib Warning: Citation `bids_coordinates' on page 4 undefined
on inpu
```

```
t line 176.


Package natbib Warning: Citation `Lanciego2020-ek' on page 4 undefined on
input
 line 182.

Package natbib Warning: Citation `linc' on page 4 undefined on input line
182.

Package natbib Warning: Citation `cmc' on page 4 undefined on input line
182.

Package natbib Warning: Citation `Milham2018-hf' on page 4 undefined on
input l
ine 182.

Package natbib Warning: Citation `Hata2023-ia' on page 4 undefined on
input lin
e 182.

Package natbib Warning: Citation `Paxinos:AP:1999' on page 4 undefined on
input
 line 184.

Package natbib Warning: Citation `Desikan2006-gr' on page 4 undefined on
input
line 184.

Package natbib Warning: Citation `Mai2023-vl' on page 4 undefined on
input line
 184.

Package natbib Warning: Citation `Petrides2012-tk' on page 4 undefined on
input
 line 184.

Package natbib Warning: Citation `Lu:SciBulletin:2024' on page 4
undefined on i
nput line 184.
```

```
Package natbib Warning: Citation `Zhang:ISMRM:2025' on page 4 undefined
on inpu
t line 184.


Package natbib Warning: Citation `Warrington2022-sx' on page 4 undefined
on inp
ut line 184.


Package natbib Warning: Citation `Coizet2017-nh' on page 4 undefined on
input l
ine 186.


Package natbib Warning: Citation `Martin:Neuroimage:1996' on page 4
undefined o
n input line 186.


Package natbib Warning: Citation `Paxinos:AP:1999' on page 4 undefined on
input
 line 186.


Package natbib Warning: Citation `ccf' on page 4 undefined on input line
186.


Package natbib Warning: Citation `Lein2007-se' on page 4 undefined on
input lin
e 186.


Package natbib Warning: Citation `Wang2020-qk' on page 4 undefined on
input lin
e 186.



[4]

Package natbib Warning: Citation `Kremer1996-iy' on page 5 undefined on
input l
ine 188.


Package natbib Warning: Citation `Zhang2025-st' on page 5 undefined on
input li
ne 190.


Package natbib Warning: Citation `Hayot-Sasson2021-fn' on page 5
undefined on i
```

```
nput line 195.


Package natbib Warning: Citation `Axer2016-np' on page 5 undefined on
input lin
e 195.


Package natbib Warning: Citation `Lefebvre2018-pb' on page 5 undefined on
input
 line 195.


Package natbib Warning: Citation `Mollink2017-uz' on page 5 undefined on
input
line 195.


Package natbib Warning: Citation `Wang2015-so' on page 5 undefined on
input lin
e 195.


Package natbib Warning: Citation `Trinkle2021-fj' on page 5 undefined on
input
line 195.


Package natbib Warning: Citation `Menzel2023-tm' on page 5 undefined on
input l
ine 195.


Package natbib Warning: Citation `Kjer2025-pz' on page 5 undefined on
input lin
e 195.


Package natbib Warning: Citation `Foxley2021-if' on page 5 undefined on
input l
ine 195.


Package natbib Warning: Citation `Shapson-Coe2024-oz' on page 5 undefined
on in
put line 195.


Package natbib Warning: Citation `Presseau2015-aw' on page 5 undefined on
input
 line 195.
```

Package natbib Warning: Citation `Rheault2017-qn' on page 5 undefined on input
line 195.

Package natbib Warning: Citation `Gabusi2024-ek' on page 5 undefined on input l
ine 195.

Package natbib Warning: Citation `Kruper2024-ew' on page 5 undefined on input l
ine 195.

Package natbib Warning: Citation `Kraaijeveld:DSG:2025' on page 5 undefined on
input line 195.

Package natbib Warning: Citation `Rheault2022-ze' on page 5 undefined on input
line 195.

Package natbib Warning: Citation `legeay2025mesoscopic' on page 5 undefined on
input line 197.

Package natbib Warning: Citation `Kruper2025gpu' on page 5 undefined on input l
ine 197.

Package natbib Warning: Citation `Hernandez-Fernandez2019-um' on page 5 undefin
ed on input line 197.

Package natbib Warning: Citation `Abernathey2021-zb' on page 5 undefined on inp
ut line 197.

Package natbib Warning: Citation `Poulin:MRI:2019' on page 5 undefined on input
line 202.

Package natbib Warning: Citation `Poulin:MRI:2019' on page 5 undefined on input

```
 line 204.


Package natbib Warning: Citation `Karimi:ImagingNeurosc:2024' on page 5 undefin
ed on input line 204.


Package natbib Warning: Citation `Neher:AP:2025' on page 5 undefined on input l
ine 204.



[5]

Package natbib Warning: Citation `Maier-Hein2017-zf' on page 6 undefined on inp
ut line 206.


Package natbib Warning: Citation `Cote:MIA:2013' on page 6 undefined on input l
ine 206.


Package natbib Warning: Citation `bai2019' on page 6 undefined on input line 21
0.


Underfull \vbox (badness 6461) has occurred while \output is active []




Package natbib Warning: Citation `BIZZI2025631' on page 6 undefined on input li
ne 215.


Package natbib Warning: Citation `Costabile2019-it' on page 6 undefined on inpu
t line 215.


Package natbib Warning: Citation `Vanderweyen2020-hd' on page 6 undefined on in
put line 215.


Package natbib Warning: Citation `Kwon2011-ms' on page 6 undefined on input lin
e 215.
```

```
Package natbib Warning: Citation `Lehman2020-pp' on page 6 undefined on
input l
ine 215.


Package natbib Warning: Citation `Nowacki2019-zm' on page 6 undefined on
input
line 215.


Package natbib Warning: Citation `Aylmore:FrontiersNeuroim:2025' on page
6 unde
fined on input line 217.


Package natbib Warning: Citation `Sarubbo2025' on page 6 undefined on
input lin
e 217.


Package natbib Warning: Citation `Kamagata:InvRadiol:2024' on page 6
undefined
on input line 217.


Package natbib Warning: Citation `Sarubbo2025' on page 6 undefined on
input lin
e 219.


Package natbib Warning: Citation `Beyh:Springer:2025' on page 6 undefined
on in
put line 219.


Package natbib Warning: Citation `Jacquesson:Neurosurg:2019' on page 6
undefine
d on input line 219.



[6]

Package natbib Warning: Citation `De_Faria2021-px' on page 7 undefined on
input
 line 228.


Package natbib Warning: Citation `Knickmeyer2008-jn' on page 7 undefined
on inp
ut line 228.
```

```
Package natbib Warning: Citation `Shi2011-db' on page 7 undefined on
input line
 228.


Package natbib Warning: Citation `sanchez2012neurodevelopmental' on page
7 unde
fined on input line 228.


Package natbib Warning: Citation `Calixto2025-ax' on page 7 undefined on
input
line 228.


Package natbib Warning: Citation `Gilmore2018-gr' on page 7 undefined on
input
line 228.


Package natbib Warning: Citation `grotheer2022white' on page 7 undefined
on inp
ut line 228.


Package natbib Warning: Citation `Guerrero2019-tq' on page 7 undefined on
input
 line 228.


Package natbib Warning: Citation `Chang2024-ab' on page 7 undefined on
input li
ne 228.


Package natbib Warning: Citation `Hollmann2020-od' on page 7 undefined on
input
 line 239.


Package natbib Warning: Citation `Shamir2025-ak' on page 7 undefined on
input l
ine 239.


Package natbib Warning: Citation `Hagen2024-tx' on page 7 undefined on
input li
ne 239.


Package natbib Warning: Citation `Tahedl2025-vw' on page 7 undefined on
input l
ine 239.
```

Package natbib Warning: Citation `Layton2017-ay' on page 7 undefined on input l
ine 241.

Package natbib Warning: Citation `Liu2024-ll' on page 7 undefined on input line
 241.

Package natbib Warning: Citation `Barker2022-jw' on page 7 undefined on input l
ine 247.

Package natbib Warning: Citation `Ambatipudi2022-su' on page 7 undefined on inp
ut line 249.

Package natbib Warning: Citation `Vohra2016-ny' on page 7 undefined on input li
ne 249.

Package natbib Warning: Citation `Lopez-Gomez2022-xp' on page 7 undefined on in
put line 249.

Package natbib Warning: Citation `Kruper2024-ew' on page 7 undefined on input l
ine 253.

Package natbib Warning: Citation `Kruper2025-uf' on page 7 undefined on input l
ine 253.

Underfull \vbox (badness 10000) has occurred while \output is active []

[7]

Package natbib Warning: Citation `Beare2023-gi' on page 8 undefined on input li
ne 257.

```
Underfull \hbox (badness 10000) in paragraph at lines 267--273
\T1/Merriwthr-OsF/m/up/7.5 (+20) Project name: Standardization-Position-
Paper;
Project
 []


Underfull \hbox (badness 10000) in paragraph at lines 267--273
\T1/Merriwthr-OsF/m/up/7.5 (+20) home-page:
https://github.com/International-So
ciety-for-
 []



No file main.bbl.

Package natbib Warning: There were undefined citations.



[8] [9{c:/texlive/2024/texmf-dist/fonts/enc/dvips/lm/lm-ec.enc}] [10]
enddocument/afterlastpage: lastpage setting LastPage.
(./main.aux)
 ***********
LaTeX2e <2024-06-01> patch level 2
L3 programming layer <2020/03/25>
 ***********

LaTeX Font Warning: Size substitutions with differences
(Font)              up to 1.0pt have occurred.


LaTeX Font Warning: Some font shapes were not available, defaults
substituted.

Package rerunfilecheck Info: File `main.out' has not changed.
(rerunfilecheck)             Checksum:
9C26A954866616CAE322C2D5112C7F9C;3997.
 )
Here is how much of TeX's memory you used:
 24691 strings out of 473583
 485908 string characters out of 5732343
 1994908 words of memory out of 5000000
 46539 multiletter control sequences out of 15000+600000
 1845061 words of font info for 543 fonts, out of 8000000 for 9000
 1141 hyphenation exceptions out of 8191
 123i,12n,131p,3240b,1142s stack positions out of
10000i,1000n,20000p,200000b,200000s
<c:/texlive/2024/texmf-dist/fonts/type1/sorkin/merriweather/Merriwthr-
Bold.pf
b><c:/texlive/2024/texmf-dist/fonts/type1/sorkin/merriweather/Merriwthr-
BoldIta
```

```
lic.pfb><c:/texlive/2024/texmf-
dist/fonts/type1/sorkin/merriweather/Merriwthr-I
talic.pfb><c:/texlive/2024/texmf-
dist/fonts/type1/sorkin/merriweather/Merriwthr
-Regular.pfb><c:/texlive/2024/texmf-
dist/fonts/type1/sorkin/merriweather/Merriw
thrSans-Regular.pfb><c:/texlive/2024/texmf-
dist/fonts/type1/public/lm/lmtt8.pfb
>
Output written on main.pdf (10 pages, 504374 bytes).
PDF statistics:
 262 PDF objects out of 1000 (max. 8388607)
 221 compressed objects within 3 object streams
 46 named destinations out of 1000 (max. 500000)
 193211 words of extra memory for PDF output out of 221844 (max.
10000000)
```

figure_2

Click here to access/download;LaTeX - Figure (eps, ps, etc.);tractography_layout2_planes_lowercase_panels.pdf

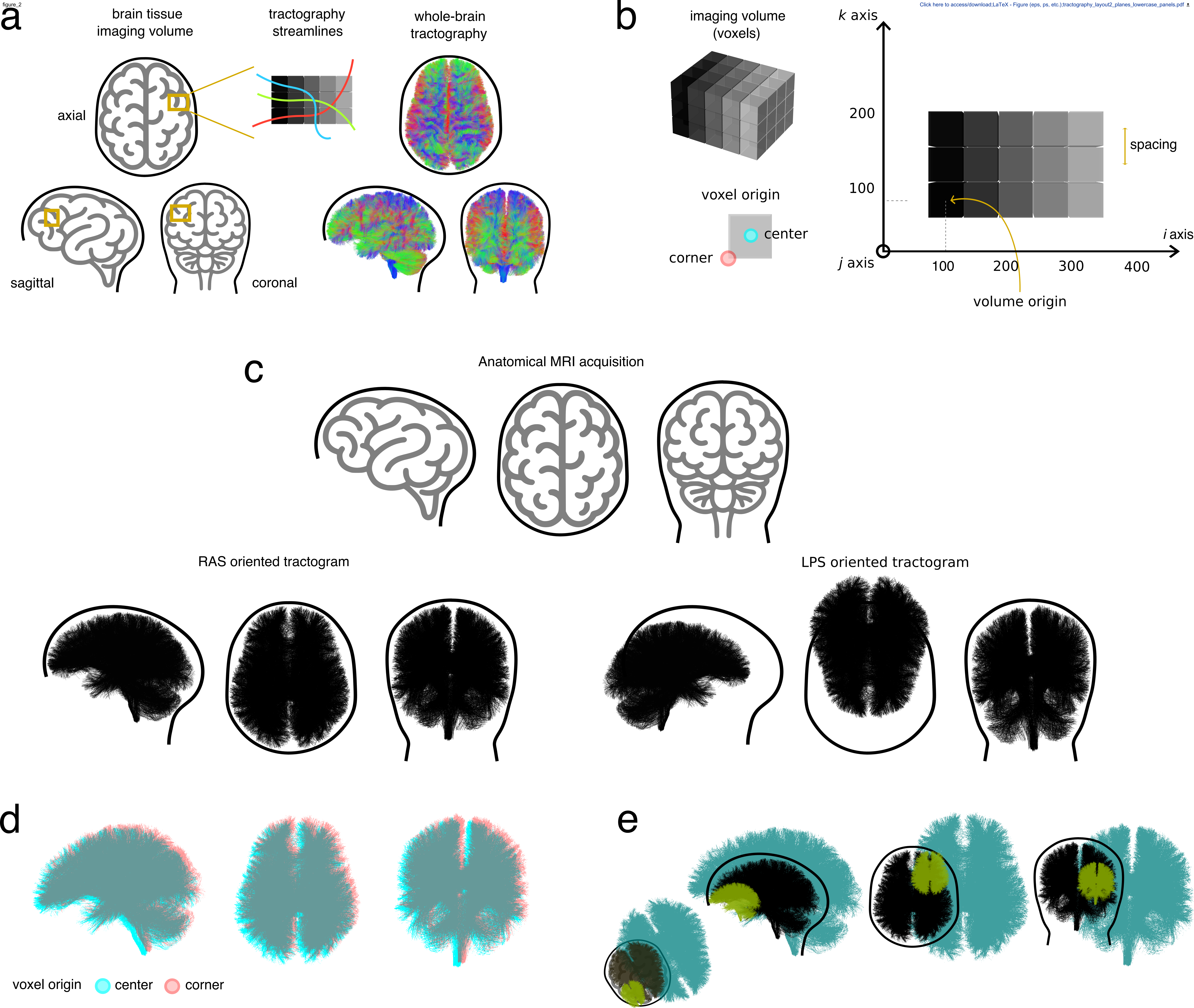

figure_1

Click here to access/download;LaTeX - Figure (eps, ps, etc.);figure_1_schematic_vertical_revised_v2.pdf

	CHALLENGES	SOLUTIONS
Data acquisition	• Variation in procedures<br>• Instrumental variability	• Use of Standard Operating Procedures (SOP)<br>• Data harmonization methods
Quality control	• Late realization of upstream issues<br>• Lack of agreement on quality control features	• Automated quality control milestones<br>• Numerical validation<br>• Best practices guidelines
Spatial coordinates	• Ambiguity of information/ interpretation<br>• Loss of crucial information<br>• Subtle errors that are hard to detect	• Enforcement of uniform coordinate metadata<br>• Documentation of QC procedures to detect errors
Species differences	• Variability in neuroanatomy: homologies/dishomologies<br>• Variability across protocols and nature of data produced	• Reproducibility/replicability; documentation<br>• Multi–modality data integration protocols
Size/scale of datasets	• Data size requires considerable storage space<br>• Data manipulation limited by computational memory	• Lossless compression and precision limitation<br>• Memory management<br>• Distributed storage
Machine learning	• High computational cost<br>• Potential for data leakage<br>• Fragmented software and model landscape	• Sharing of annotated data and models<br>• Consistent use of hold out data<br>• Adherence to industry standards for sharing of models
Interoperability	• Gap between research and clinical usage scenarios<br>• Bespoke pipelines<br>• Inconsistencies across practitioners	• Standardized protocols<br>• Validation and consensus<br>• Tools for integration of tractography into clinical records
Lifespan	• White matter development<br>• Differences between healthy and non–healthy populations	• Age–based models<br>• Methods adapted to different populations

Manuscript

Click here to access/download;Manuscript;ist_su_what_needs_to_be_stand

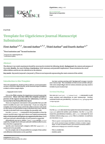

GigaScience, 2023, 1–15

doi: xx.xxxx/xxxx
Manuscript in Preparation
Review

REVIEW

What needs to be standardized for reliable, reproducible, and robust tractography?

Jon Haitz Legarreta[1,2*], Simona Schiavi[3,4], Wei Tang[5], Garrett Banks[6], Matthew Cieslak[7,8,9], Kurt Schilling[10], Alberto De Luca[11], Jacques-Donald Tournier[12,13], John Kruper[14], Francois Rheault[15], Stamatios N. Sotiropoulos[16,17], Franco Pestilli[18,19], Jelle Veraart[20], Joseph Yuan-Mou Yang[21,22,23], Maxime Descoteaux[15], Sarah Heilbronner[6] and Ariel Rokem[14,24*]

[1]Department of Radiology, Brigham and Women's Hospital, Mass General Brigham, Boston, MA, USA and [2]Harvard Medical School, Boston, MA, USA and [3]ASG Superconductors S.p.A., Genoa, Italy and [4]Department of Computer Science, University of Verona, Verona, Italy and [5]Department of Psychological and Brain Sciences, Indiana University, Bloomington, IN, USA and [6]Department of Neurosurgery, Baylor College of Medicine, Houston, TX, USA and [7]Lifespan Informatics and Neuroimaging Center (PennLINC), Department of Psychiatry, Perelman School of Medicine, University of Pennsylvania, Philadelphia, PA, USA and [8]Penn/CHOP Lifespan Brain Institute, Perelman School of Medicine, Children's Hospital of Philadelphia Research Institute, Philadelphia, PA, USA and [9]Department of Psychiatry, Perelman School of Medicine, University of Pennsylvania, Philadelphia, PA, USA and [10]Department of Radiology, Vanderbilt University Medical Center, Nashville, TN, USA and [11]Image Sciences Institute, University Medical Center Utrecht, Utrecht, The Netherlands and [12]Department of Biomedical Engineering, School of Biomedical Engineering and Imaging Sciences, King's College London, King's Health Partners, St. Thomas' Hospital, London, UK and [13]Centre for the Developing Brain, School of Biomedical Engineering and Imaging Sciences, King's College London, King's Health Partners, St. Thomas' Hospital, London, UK and [14]Department of Psychology, University of Washington, Seattle, WA, USA and [15]Sherbrooke Connectivity Imaging Lab (SCIL), Department of Computer Science, Université de Sherbrooke, Sherbrooke, Québec, Canada and [16]Sir Peter Mansfield Imaging Centre, School of Medicine, University of Nottingham, Nottingham, UK and [17]NIHR Nottingham Biomedical Research Centre, School of Medicine, Queen's Medical Centre, Nottingham, UK and [18]Department of Psychology, The University of Texas at Austin, Austin, TX, USA and [19]Department of Neuroscience, The University of Texas at Austin, Austin, TX, USA and [20]School of Medicine, New York University, New York, NY, USA and [21]Department of Neurosurgery, Neuroscience Advanced Clinical Imaging Service (NACIS), Royal Children's Hospital, Melbourne, Victoria, Australia and [22]Neuroscience Research, Murdoch Children's Research Institute, Melbourne, Victoria, Australia and [23]Department of Paediatrics, University of Melbourne, Melbourne, Victoria, Australia and [24]The University of Washington eScience Institute, University of Washington, Seattle, WA, USA

*jhlegarreta@bwh.harvard.edu; arokem@uw.edu

Abstract

Tractography is a key component of efforts to map brain connectivity. As a rapidly-evolving field of neuroscience, current tractography methods are diverse, often varying across research laboratories and different software pipelines. Therefore, it suffers from a lack of standardization leading to inconsistencies in results, which can limit reproducibility, and affect the robustness needed for research and clinical applications of these methods. Variability in data acquisition procedures, inconsistencies in spatial referencing schemes and implementations, and anatomical heterogeneity —at the individual level, across the lifespan, and across species— hinders comparative analyses. Additionally, the lack of consensus on best practices complicates the development of robust automated quality control pipelines and limits the clinical translation of tractography-based procedures. Establishing standardized protocols for acquisition, preprocessing, and tractography reconstruction are critical towards enabling reliable tract-specific analyses, facilitating cross-study harmonization, and supporting replicable large-scale population studies. The present article provides an overview of the current challenges in tractography standardization and identifies the key aspects that require standardization for reliable, reproducible, and robust tractography.

Key words: Neuroanatomy; standardization; tractography; brain connectivity; white matter; computational neuroimaging

Background

Understanding human brain anatomy across different organizational levels is a central goal of contemporary neuroscience. There is increasing evidence that structural connectivity across the white matter underlies many of the capacities of the living brain and that their physical properties are linked to human brain health [1, 2, 3, 4]. This understanding has grown with the development of methods to measure brain connections and it has also fueled a new generation of data collection and data analysis methods. Magnetic resonance imaging (MRI) measurements are non-invasive and can be used to study brain connectivity *in vivo* [5]. These data are complemented by other invasive techniques that highlight brain connections by using specific chemical tracers, histological stains and molecular markers, by dissecting the tissue, and by direct optical observation [6, 7, 8, 9, 10, 11, 12, 13], or using X-ray imaging methods [9, 14]. *Computational tractography* assesses the location, direction and pattern of brain connections estimating their trajectories (with an individual trajectory often referred to as a *streamline*). However, as the methods evolve and the promises from their application grow, it is important to take stock of challenges that hinder leveraging their full potential. Other works have examined challenges related to accurate delineation of brain connections [15, 16, 17, 18] or challenges related to definitions of certain anatomical concepts in such data [19, 20]. The present paper focuses specifically on the challenges related to standardizing representations of tractography-derived brain connections in digital formats.

These challenges have become increasingly pressing as the field has transitioned in recent years towards larger and larger datasets. This increase in data volume and complexity arises from large-scale data acquisition projects, with thousands of subjects, on the one hand. Examples of these include the Adolescent Brain Cognitive Development (ABCD) [21], Healthy Brain and Child Development (HBCD) [22], and Healthy Brain Network [23, 24] studies, as well as the UK Biobank [25], and many others. On the other, it arises from the increase in the resolution as well as coverage of measurements. These larger data have led to the application of data-driven discovery methods, where consistent and comprehensive standards are necessary.

Establishing standards and best practices supports transparent, reproducible and robust research through application of the FAIR (Findable, Accessible, Interoperable, Reusable) principles [26]. In addition, establishing usable standards can enable research that is otherwise difficult, making things that are hard easier, and making things that are otherwise impossible practical. With the recent establishment of the International Society for Tractography (IST) [27] and its standardization unit (members of which are authors of this paper), together with several consensus efforts in diffusion MRI (dMRI) acquisition and processing [28, 18, 29] led by the Diffusion Study Group [30] of the International Society for Magnetic Resonance in Medicine (ISMRM) [31], we see an opportunity to advance broadly-applicable and widely-accepted community standards that will pave the way towards future research of brain connectivity.

This work introduces some of the current challenges that arise from gaps in standardization for tractography and offers some potential solutions to them. We provide a set of recommendations for the broader research community to pursue the solutions that we identify. The recommendations presented herein will improve the rigor and impact of work that uses brain tractography and will enable better understanding of brain connectivity. The paper is organized by the different stages of the data life-cycle and a range of domains in which standardization poses challenges (Figure 1), concluding with a set of recommendations (section Summary and recommendations).

Challenges and Solutions

Reliability, reproducibility, and robustness in tractography encompass multiple methodological dimensions. They manifest across the full data life-cycle of tractography, including acquisition, processing, and analysis. They also manifest in inferences applied across different species, different types of measurements, different ages, and across basic research and clinical application. There are many definitions of these constructs [32, 33, 34]. We define *reliability* as the stability of tractography algorithms in the face of varying conditions (e.g., noise perturbations in test-retest, multi-site or multi-vendor datasets, etc.). *Reproducibility* is defined as the ability to obtain the same results with the same data and same software used in the original study. Thus, reproducibility is mostly about the open and unencumbered availability of research products and importantly, the compliance of openly available products with conventions and standards. *Replicability*, a closely related term, is the ability of another research team to produce findings that are consistent without using the materials used by the original research team, but while trying to emulate the methods as closely as possible. This will depend to a large degree on adequate description of the methods used. This will also depend on the *robustness* of the findings, which we define as the ability to obtain the same expected anatomical observations using different data and/or different software or even methods that differ in their assumptions and implementation details. For example, the ability to delineate a certain brain structure in data obtained on different instruments, from different participants, and using different tractography algorithms. All three of these are necessary conditions for trustworthy, transparent research, and an ability for research efforts to efficiently build on previous work. As we will demonstrate in addressing a range of practical and conceptual challenges, standardization is essential to achieve these goals. Therefore, as we survey a range of challenges and proposed solutions, we will highlight key factors influencing these aspects and propose ways to increase them.

Data acquisition

The utility of standards starts with the moment that data are created. Different experimental methods produce tractography data, and considerations for standardization may be different for each one of these. Furthermore, the creation of some data is already governed by numerous existing standards. For example, in a large number of cases, MRI data is acquired following the Digital Imaging and Communications in Medicine (DICOM) standard [35]. Similarly, modern storage and sharing of reconstructed (raw or processed) research MRI data is standardized by the Brain Imaging Data Structure (BIDS) [36] specification [37], including several extensions for dMRI and brain connectivity (e.g., [38, 39]). These standards facilitate not only the structured storage of the data itself, but also prescribe necessary metadata. In principle, this should facilitate the standard processing in subsequent steps, but undocumented and poorly standardized procedures are typical of many different experimental techniques and a source of unwanted variability.

Differences across datasets can also arise due to a range of non-biological variables, such as variation in acquisition protocols, scanner hardware, reconstruction pipelines, and tractography workflows, that are hard to reconcile even given full description of the data acquisition. These sources of variability can have significant impact on downstream tractography results, particularly in multi-site studies and large-scale datasets. For example, acquisition resolution, diffusion sampling schemes, and vendor-specific differences introduce variability in the spatial geometry and microstruc-

Compiled on: March 19, 2026.
Draft manuscript prepared by the author.

tural characteristics of reconstructed brain tracts that compromise reproducibility and robustness [40, 41, 42, 43, 44].

Statistical methods for *harmonization*, which aim to eliminate such differences at the level of the raw data [45], provide promising solutions to some of these challenges, but the intricacy of downstream tractography analysis workflows poses further challenges. These workflows involve subjective parameter choices, user-defined constraints, and possibly different priors or anatomical reference definitions that influence reproducibility and anatomical fidelity. Cross-scanner and cross-vendor effects propagate through these pipelines, leading to inconsistencies in reconstructed geometry, volume, and quantification of tract properties. Recent efforts to standardize processing pipelines and implement robust quality control protocols across sites have shown promise in reducing these inconsistencies, thus improving robustness [46, 47, 48, 49].

Tractography is rarely the sole purpose of acquiring dMRI data. Quantitative characterization of tissue microstructure is often a concurrent goal, which imposes additional and occasionally conflicting constraints stemming from distinct acquisition requirements. As the sequences for probing the tissue microstructure get more sophisticated (e.g., using tensor-valued encoding), more variability can be expected to be present in the dMRI data, and more nuanced tractography reconstructions can be expected [50]. Thus, additional methodological development is needed to align such multidimensional acquisition protocols across sites, to manage scan time constraints while jointly supporting tissue microstructure modeling and tractography.

Despite the progress that has been achieved, further work is needed to provide an adequate basis for tractography. Using BIDS for neuroimaging data sharing offers a high-level of consistency; however, its adoption across pipelines is not yet all-pervading, and its implementation is not uniform. Furthermore, although the diffusion extension proposal has been submitted for inclusion in the standard, it only covers scalar derivatives and tensors. The tractography extension has not been submitted yet for inclusion. Similarly, vendor-agnostic, standardized sequences are yet to be implemented across the variety of scanning hardware and protocols, and be validated extensively. Limited support and interest for regulatory clearance entail additional hindrance to their wide adoption [51]. Finally, harmonization is still a topic under active research, or present limitations (e.g., fixed, well populated site numbers, homogeneous populations) that have yet to be overcome. Thus, significant methodological gaps remain in diffusion MRI data acquisition that limit the development of automated and robust tractography downstream.

Standard operating procedures (SOPs) are necessary to remove variability at data collection and provide some level of reliability in tractography reconstruction from a given study. SOPs have been employed to describe the imaging and processing protocols for large-scale studies (e.g., the Human Connectome Project (HCP) [52], the ABCD study [53], etc.); more recent studies (e.g., the HBCD study [54], or the Human Connectome PHantom Study (HCPh) [55]) have adopted contemporary web technologies to modernize data presentation. Smaller-scale, single-site studies would likewise benefit from this practice, contributing to improved transparency in reporting and reproducibility. Similarly, standardized data and metadata records and organization (e.g., through BIDS) are required to ensure reproducibility. This includes developing the extensions to derivatives (e.g., fiber orientation distributions) that are employed to generate tractography results. Finally, harmonization solutions (within and across scanners and sites, across acquisition upgrade packages, etc.) are required to resolve data source variability. Combined with the adoption of unambiguous, self-reporting data formats (see Spatial coordinates), these measures would mitigate practical barriers and contribute to reducing overhead in multi-center studies with disparate settings.

Quality control

There are many well-established pipelines for quality control (QC) of raw MRI data [56], including dMRI data [57, 58], as well as data that has undergone initial pre-processing (i.e., denoising, correction of motion and eddy current artifacts, and removal of other artifacts) [59, 60, 24]. Presumably, QC procedures that are applied at these early stages should catch many of the issues that would impact subsequent analysis steps. However, QC can and should be done at multiple different stages of the analysis, because errors can occur at each one: e.g., registration between different imaging modalities, separation of the image into different tissue types and regions of interest for tractography initiation, etc. Often times, many of these issues only become apparent when computational tractography is conducted. This means that QC of tractography results is still necessary. In practice, QC of computational tractography pipelines is often done through visual examination of whole-brain tractograms (the collection of all streamlines estimated in a single brain), or by their ability to identify the locations and trajectories of large, well-known tracts. However, this approach is challenged by high inter-rater variability and an apparent lack of consensus on the structure of these tracts, even in the same set of streamlines [19, 20].

Another approach for QC of tractography results is through numerical validation. In this approach, individual streamlines and sets of streamlines are subjected to statistical evaluation with respect to the measured data. In these methods [61, 62, 63, 64, 65, 66] individual streamlines or sets of streamlines are given objective scores, based on how well their trajectory conforms to the data that was used to generate the streamlines (e.g., in contrast to smoothness constraints, anatomical constraints, or randomness that is introduced in the process of tractography). Other methods use machine learning and deep learning techniques to filter streamlines based on their geometric properties or a representation of them [67, 68]. The benefit of these approaches is that they provide objective numerical values that can then be used as benchmarks for QC procedures. Overall, the field would benefit from more studies that demonstrate the utility and sensitivity of different QC procedures in relevant scientific inferences [69, 70], including validation by reference to known anatomical structure, and via the detection of individual differences related to development, aging, or clinical conditions [71].

Taken together, progress has been limited in tractography QC standardization practice and adoption levels. Although there are many advanced tractography visualization tools and paradigms [72], and despite some pipelines offering automated reporting, their use for QC purposes has not been thoroughly studied. Similarly, validating numerical tractography QC methods is still impacted by the inability to have gold standards on real brain data, and the lack of anatomical measures defining the success of a tractography method. As a result, best practices for excluding low-quality results based on either visual or numerical reports remain insufficiently established. Reliable tractography would benefit from clearly defined, shared criteria determining the quality of results, including standardized reporting. Additionally, universally shared phantom, and synthetic data, as well as anonymized *in vivo* and *ex vivo* data from both healthy and diseased participants are required to guarantee a common ground for quality control of a given tractography pipeline. This includes sharing data in software-agnostic repositories (e.g., OpenNeuro [73] or Zenodo [74]). Note that this extends beyond datasets used across international challenges that quantify tractography results only within a particular dataset (see, for example, [75, 17, 76, 16]).

Spatial coordinates

Representing spatial coordinates in an unequivocal frame is a known challenge in many fields of science that deal with spatial information. This is a well-recognized issue in anatomical studies of the human brain, where different types of information (e.g., structural, functional, physiological, etc.) need to be integrated. Given the variety of information sources that are involved, each acquisition modality having particular spatial properties, the potential for errors is high and the consequences detrimental to the study of brain connectivity [77].

Tractography algorithms typically output data as a set of three-dimensional (3D) coordinates that represent the trajectory of particular brain white matter tracts (see Figure 2a,b). In reconstructing and interpreting these structures, several coordinate frames need to be considered. One coordinate frame is the one that refers only to the individual participant's brain, allowing to locate a structure relative to a particular anatomical landmark. Another coordinate frame is the position of the individual's brain within the scanner, i.e., relative to the origin of the scanner's fixed reference frame (e.g., the iso-center of the MRI's bore). Another coordinate frame to consider is that of the grid of *voxels* that are included in the measurement itself.

Volumetric imaging data follows particular anatomical conventions that facilitate relative positioning and orientation. These conventions split the space according to three orthogonal planes, namely *axial*, *coronal*, and *sagittal* planes (Figure 2a). When storing real-world spatial data into discrete imaging data, the information can be arranged according to a particular basis. Two typical conventions are *RAS+* (Right, Anterior, Superior, the + sign indicating that coordinates increase from left to right, posterior to anterior, and inferior to superior) and *LPS+* (Left, Posterior, Superior; the same principle applying to the + sign). If no metadata is accessible, applications reading tractography results may assume the incorrect convention (Figure 2c).

At times, the conventions implied by a particular format are not made apparent in the corresponding documentation. A typical example of this issue is the definition of the origin of a voxel: some tractography formats consider the origin being at the center of the voxel, whereas others consider that it lies at the corner. This may arise in the correspondence between streamline coordinates and other coordinate-based (e.g., volume-based) data: unless this is carefully considered, a half-voxel shift will exist between the streamlines relative to their intended world coordinates. Visual inspection is not always sufficient to notice this (Figure 2d), and such small systematic errors can have significant consequences in downstream applications. Some tractography data processing tools may fail to read streamlines that, due to not accounting for this shift, lie outside the volume bounding box. In connectivity analyses, these errors may lead to mismatches between surfaces or label maps reconstructed from T1-weighted data and the tractography space, resulting in incorrect streamline assignments and spurious inter- or intra-hemispheric connectivity patterns.

Finally, streamlines generated by tractography algorithms may have their own positions relative to each of the other coordinate frames, and may be tied to an additional piece of data (e.g., another data file). The units used to store their position may also differ from the units used to refer to the measurement (discrete voxels, or continuous mm). Incongruent coordinate frames and units can lead to errors such as the ones shown in Figure 2e.

Overall, despite a well-established understanding of the coordinate frameworks involved in tractography reconstruction, current pipelines continue to rely on implicit or insufficiently documented conventions. Clear and unambiguous guidance for debugging, validating, and converting data across coordinate spaces and formats remains lacking. Consequently, meaningful progress in this area has remained limited. A tractography data standard that explicitly and unambiguously documents its spatial coordinate framework (e.g., according to the BIDS convention [78]), and version-controlled, general resources (i.e., not tied to a particular processing package) are required. This includes testing data stored in software-agnostic repositories, and guidelines towards conformance, error checking and conversion of retrospective data.

Translation across species and methods

Anatomical tract-tracing methods involve injecting a dye or tracer into a living brain, waiting a set period of time, then extracting the brain for histology and microscopy [79]. Thus, these methods provide information that is not accessible with MRI but, for obvious reasons, cannot be performed in humans. Thus, tract-tracing is performed in brains from non-human animals, such as primates and rodents. Combining these methods with dMRI in non-human models provides a unique opportunity to verify dMRI-based tractography against anatomical gold standards, and to iteratively improve these methods. Several large-scale efforts aim to provide high-quality dMRI along with microscopy and tracers in the same non-human primate brains (e.g., the Large-scale Imaging of Neural Circuits (LINC) BRAIN CONNECTS center [80], or the Center for mesoscale connectomics (CMC [81])). Openly accessible resources from these consortia and others (such as PRIME-DE [82], or the BRAIN/MINDS portal [83] will be invaluable in optimizing, standardizing, and developing the next generation of tractography approaches. Using unified methods that combine these multi-modal and multi-scale data would ultimately enable informed judgment about cross-species homologies/dishomologies. However, this process is complicated by several factors. Non-human brains —even in our closest primate relatives— are not simply smaller, but also have different morphological and biophysical properties.

This poses foremost a challenge to the validity of inferences between species. Integration between human and non-human primate data remains conceptually challenging, because non-human anatomical knowledge relies heavily on atlases based on cytoarchitectonics (e.g., [84]). These do not directly align with the common atlases used in human MRI studies (e.g., [85]). While cytoarchitectonics-based human brain atlases have been developed [86], some even with a particular stress on cross-species homology [87], these have not been integrated with widely used neuroimaging tools. At the same time, common coordinate frameworks for non-human primates are beginning to emerge (see, for example, [88]), in some cases with the explicit goal of establishing a direct relationship with the developing human connectome [89]. Tractography fingerprinting [90], i.e., using white matter bundles as latent landmarks to assess similarities and divergences across the two species, is geometry-agnostic and can provide additional solutions to the challenge of integrating cross-species coordinate frameworks.

Integration with studies in rodents are further complicated by the extent of species dishomology. Their white matter bundles are organized very differently from primates' [91], and their lissencephalic brains mean that many of the MRI-based tractography methods that work in primate brains do not translate. On the other hand, there are a host of genetic, molecular, and imaging tools readily available for circuit characterization and manipulation in mice that are not feasible in humans or non-human primates. Thus, merging dMRI tractography with tract-tracing, spatial transcriptomics, and imaging data in the mouse model could be highly valuable. However, while anatomical atlases in primate models have a long history (e.g., [92, 84]), just two decades ago, there were virtually no standard frameworks (atlases, metadata, structural ontologies, etc.) for the mouse brain. However, with major investments in generating large-scale datasets of mouse brain transcriptomics, cell typing, connectivity, and function, the need for a standard framework became overwhelming. The Allen Mouse Brain Common Coordinate Framework (CCF) [93, 94] (current version

CCFv3 [95]) has emerged as a standard framework for organizing data about mouse brain connectivity. The enormous success of this framework illustrates the value of standardization. An extensive informatics pipeline at the Allen Institute for Brain Science allows users to download everything from raw images to structural summaries of connectivity, and tools that use the CCF can integrate data from multiple sources. However, these tools are not currently interoperable with MRI data, aside from at the crudest (region-to-region connectivity summaries) level.

The methods employed to map connectivity across species also differs in profound ways due to the nature of the acquired data; while axonal pathways in the rodent brain are reconstructed from optical data (e.g., electron microscopy), human brain pathway reconstruction using tractography employs dMRI data. Thus, the data representation structures employed are different: in rodent data, anatomical structures (gray/white matter boundaries, nuclei volumes, cells, fibers, etc.) are represented in standard anatomical reconstruction software (e.g., Neurolucida) that interface with microscopes as points, contours and surfaces. Downstream processing software such as IMOD [96] inherits the file formats that organize these structures. This is conceptually different from the MRI file conventions, which use voxels as their primary representational unit. The nature of the contrast in the acquired data necessitates the development of distinct methods (e.g., segmentation and stitching as opposed to streamline propagation), introducing a supplemental layer for divergence. As a consequence, the progress to bridge the gap in tractography data representation and software across species and imaging modalities has been limited. Addressing these challenges requires a comprehensive approach that includes standardized multi-modality and multi-resolution data acquisition, advancements in hardware for efficient *in vivo* scanning, essential validation studies, and methodological innovations, such as automated tools for establishing a common framework of correspondence.

While the challenges to validity and generalization are evident, these issues also raise challenges to robustness. This is because the differences in measurement tools and in analysis methods need to be thoroughly adapted and validated when they are used in species other than the ones on which they have been developed. Examples in which tractography tools that were developed for use in human dMRI data were adapted to be used in non-human data [97] suggest that there is moderate progress on this challenge.

The scale of data

Like many other datasets of spatial biological information, tractography data can take up large amounts of storage. A typical dMRI tractography file occupies several gigabytes (GB) of data. The size depends on the spatial and angular resolution of the diffusion data, the sampling density employed to reconstruct streamlines, the number of streamlines that were generated, the data associated with each point or each streamline (e.g., scalars that represent tissue properties along their length etc.), as well as the precision employed to store the data. Additionally, the size of the tractography output may also depend on the resolution of the acquisition. For example, with sub-millimeter resolution MRI acquisitions, the size of tractograms can easily reach the terabyte-scale (TB) [98]. With other modalities that provide even higher image resolution in *ex vivo* tissue samples, such as polarization microscopy [7, 8, 10, 12], X-ray microcomputed tomography [99], X-ray scattering [9], or synchrotron technology [14], data is orders of magnitude larger (see, for example, [100, 101]). Manipulating such data volumes pose a challenge in terms of the required hardware and computational power. As imaging acquisition hardware evolves, and data storage capacity and physical memory limits increase, current analysis tools may buckle under the strain. This means that some approaches, which work well with smaller datasets, can become infeasible when translated into larger data, threatening robustness. A few technical and standardization developments may ease this strain. First, tractography data lends itself to compression, with *linearization*, where collinear points are discarded (with some tolerance threshold), being the fastest and most efficient approach [102, 103, 104]. Additional compression is provided by using lower precision numerical representations (i.e., 16-bit floating point precision, instead of the extended 64), which can provide significant space saving, without much loss of anatomical information [105]. Similarly, some visualization techniques such as level of detail, occlusion culling or visibility-based rendering optimization aim to save computational resources by avoiding rendering data that is occluded by other objects or by rendering it at lower resolutions (see [106] for a recent method). Second, new file formats and distributed computing paradigms offer opportunities to scale compute to very large datasets. As an example, a newly-proposed file format, *TRactography eXchange* (TRX) [107], was designed to make it easy to create large tractograms with minimal random access memory requirements. This enables memory mapping, providing a convenient and efficient way to access data directly from disk.

Software and data-format enhancements have so far enabled only limited progress, due to heterogeneous precision adjustment across pipelines and partial adoption of the TRX file format. Data storage and visualization remain major bottlenecks, and significant advances have relied primarily on improvements in computational power, with efficient mapping of brain connectivity at scale being possible on supercomputing hardware [108] or using Graphics Processing Units (GPUs) [109, 110]. Distributed data storage (e.g., with zarr [111]) could offer an attractive possibility for work with very large datasets. Effective visualization of memory-intensive tractography data will require implementations in low-level programming languages, such as C++ or Rust, and/or compatibility with GPU hardware acceleration.

Integration of modern machine learning methods

The increasing adoption of machine learning and artificial intelligence (ML/AI) methods in dMRI and tractography has raised new challenges and exposed limitations in current standardization practices. Modern ML/AI methods require large, diverse, and well-curated datasets to achieve robust generalization. These requirements compound many of the challenges described. For example, in the absence of reliable and automated QC tools, labor-intensive quality control procedures are required. Spatial coordinate inconsistencies within large heterogeneous datasets may cause deep learning models to fail to converge. Similarly, poor data organization and metadata standardization —due to both poor compliance with BIDS and the preliminary status of the related extensions— hinder reproducibility and compromise downstream analyses, making cross-study comparisons more challenging. Inadequate handling of data splits can lead to train–test leakage [112]. Such leakage risks inflating reported performance and obscure the true generalizability of ML/AI-based tractography methods. Though there are some annotated datasets that can be used as the basis for training ML/AI, the scarcity of large, well-annotated, and openly shared tractography datasets —particularly multi-site and longitudinal datasets that include non-healthy individuals for clinical relevance — constrains the development of robust and reliable tractography. The difficulty of reliable evaluation, and the absence of universally accepted anatomical ground truth in particular, is not a specific limitation for the evaluation of ML/AI algorithms, but for the evaluation of tractography in general. Nevertheless, while these issues are broadly applicable to tractography methods more generally, they are exacerbated in ML/AI methods, which are oriented towards quantitative metrics of performance for their consistent improvement.

Furthermore, there is need for standardization within the ML/AI

methods themselves. ML/AI-based tractography techniques exhibit substantial heterogeneity in model architecture, input representation, and learning objectives, often making direct comparisons difficult (see [112, 113, 114] for relevant review works). Existing approaches range from voxel-wise classifiers and streamline propagation networks to graph-based and geometric learning formulations. As such, the input to the model may be the raw diffusion signal, the local orientation information represented as spherical harmonics coefficients or fiber peaks, etc. Additionally, the contextual information to regularize the optimization process is also heterogeneous across methods, and includes tissue maps, neighboring voxel information, etc. Other approaches avoid reliance on the modality that sensitizes axonal pathway architecture (e.g., dMRI or other), and estimate tractography without explicit orientation data. These factors result in differing dataset requirements for training various models, often leading to substantial increases in computational requirements, both in terms of storage and processing resources. The high computational cost —including the need for specialized hardware, such as GPUs— makes regular testing and evaluation of methods expensive and resource-intensive.

This heterogeneity is compounded by the absence of universally accepted anatomical ground truth for white-matter pathways, a limitation that challenges the current tractography landscape. The evaluation of AI-based tractography algorithms frequently relies on indirect metrics, simulated phantoms, or expert-defined references, each with inherent limitations. In this context, the absence of standardized reporting conventions is particularly problematic for the evaluation of AI models. Although volume- and streamline-oriented measures are well-established since landmark studies [17, 115], evaluation tools remain poorly maintained, with most pipelines relying on bespoke implementations. Additionally, the assessment of application-specific derivatives —such as tract-specific metrics, connectivity measures, or quantitative microstructural parameters— is often inconsistent or omitted entirely. Additionally, representations and trends learned by ML/AI systems may be dominated by site- or protocol-specific effects. Systematic reporting of scanner characteristics, acquisition parameters, session-level metadata, and preprocessing choices is essential for replicability and robustness and for interpreting performance gains as genuine methodological advances.

The result is a fragmented landscape in which performance claims are highly dependent on specific datasets, tasks, and evaluation choices, increasing the difficulty of reliable evaluation. Together, this underscores the necessity of standardization for AI-based tractography, encompassing access to well-annotated and fully described datasets, common validation frameworks with standardized benchmarks, metrics and reporting procedures. Such standardization would support integration with established neuroimaging pipelines, improve reproducibility, robustness, and maintainability. At the same time, it is important not to over-standardize too early. That is, early-stage research needs to maintain diversity in the approaches a scientific community explores, which is essential for innovation, and increases the opportunities for the community to discover good solutions. Taken together, these considerations suggest that standardization of ML/AI approaches merits caution and careful consideration, to support the goals of reliability, reproducibility, and robustness, even while not stifling innovation.

Building on the heterogeneous methodological terrain described above, competition among large corporate entities (e.g., technology companies) is also driving further barriers to interoperability in this ecosystem, as different entities try to position their tools as dominant in the marketplace. As externally developed components are introduced into established processing pipelines, they promise incremental value but require careful accommodation within historically entrenched workflows. In cases where models are shared not only as software, but also in the form of parameter values (or "weights"), the standardization of the format of model parameters, and of the metadata associated with these parameters becomes important. This is where industry standards such as the Open Neural Network Exchange (ONNX) standard [116] could play an important role. For these use-cases, there is much to benefit from the adoption of already-existing standards that apply broadly, rather than reinventing these standards for narrow applications.

Translation of tractography methods to clinical applications

Tractography is used clinically to aid in the planning and execution of neurosurgical procedures [117]. Tractography has proven useful during the resection of epileptic foci, or brain tumors [118, 119]. In this case, a surgeon might utilize a different surgical approach to the tumor to avoid certain white matter tracts, particularly those involved with motor, language and visual function. This is especially the case in slow growing tumors and pediatric developmental abnormalities, where the standard anatomical organization of white matter pathways can be significantly altered while remaining functional. To this end, tractography-guided brain tumor resections rely on functional brain mapping through direct brain electrical stimulation to confirm white matter tract positions and resection functional boundaries during awake surgery. In other instances, tractography is utilized for precision targeting in stereotactic procedures such as deep brain stimulation and focused ultrasound, such as when localizing the dentatorubrothalamic tract, a neuromodulation target for treatment of essential tremor and tremor-dominant Parkinson's disease [120, 121, 122].

Despite constituting a useful tool for improving neurosurgical outcomes and mitigating the likelihood of postoperative complications, its use remains limited [123, 124]. Diffusion tensor-based deterministic tractography remains the prevalent tool in neurosurgical preoperative planning, largely because it is supported by many commercially available navigation platforms [125]. Yet, limitations of the tensor model result in incomplete reconstruction and visualization of complex fiber architecture (e.g., crossing, fanning, and bending pathways) in clinical practice.

Tractography with advanced models is beginning to appear in commercial software, but adoption remains slow due to increased likelihood of spurious fibers, limited clinical validation, and inconsistent protocols and heterogeneous methodological frameworks [124, 126]. This is especially pronounced when examining fine-scaled structures, such as cranial nerves, and highlight the sensitivity of the tracking parameters with respect to the structures of interest [127]. Additionally, results provided by intraoperative tractography are constrained by the limited acquisition and processing time.

Standardization of clinical workflows is complicated, because these applications require thorough validation and consensus among experts. This requires processing software to remain very stable over time, and makes updates to the software very difficult in these settings. Variable imaging protocols also make consensus more difficult to reach (see section Data acquisition). Benchmarks and standard validations methods are very challenging to formulate, because clinical use-cases are diverse, and there are concerns that methods may be affected by the presence of pathologies. Furthermore, clinicians (e.g., radiologists or neurosurgeons) may use bespoke pipelines developed in-house to perform tractography, making translation across settings difficult. Finally, depending on the clinical setting and operative urgency, some processing pipelines that are common in research settings may be too time consuming and/or computationally intensive.

Thus, unlocking tractography's full potential in clinical routine necessitates further development along several critical dimensions. Consistent acquisition and processing protocols are required to produce tractography results that can be translated across clinical settings. In addition, methodological innovations are needed for

time-constrained clinical acquisitions —such as optimized pulse sequences, undersampling strategies, and super-resolution techniques— that enable robust tractography results, alongside tractography methods that remain reliable when employed on lower-quality data. Additional measures to support clinical adoption include systematic reporting of reconstruction uncertainty across varying data quality conditions (e.g., noise levels) and reconstruction settings (e.g., seeding strategies). Finally, establishing reliable proxies for clinical outcomes is essential to validate the reliability of fiber reconstruction techniques.

Standardization of methods throughout the lifespan

The brain changes significantly throughout the lifespan, and there is a wealth of evidence that lifespan development of brain connections is linked to health outcomes (see [128] for a review). The changes in the properties of the brain also pose challenges for standardization of methods across different epochs of life. For example, studying early life brain development is challenged by the simple fact that the brain is substantially smaller at birth, with total brain volume more than doubling during the first 12 months of life [129]. This poses a particular challenge to the use of standard atlases that localize certain structures, as these atlases are usually constructed based on healthy young brains. To address this challenge, researchers have been developing detailed and time-resolved atlases in early life [130, 131] and even during gestation [132]. Furthermore, while the major brain tracts are already established at birth [133], it is not simply the case that the infant brain is a scaled-down version of the adult brain. There is some evidence that the curvature of some structures is different in this early phase relative to later development [134]. In addition, the tissue properties of brain connections in early life are quite different than those of a more mature brain. This can pose a challenge to the use of standard tractography methods, which sometimes rely on assumptions about the biophysics of the tissue, which may not hold [135]. Similar changes apply in aging, as tissue properties of the white matter change again with age in a manner that can impact standard tractography methods [136]. The challenge of studying the developing and aging brain often intersects with challenges related to harmonization (mentioned in section Data acquisition) because it is difficult to obtain a large sample that covers all ages within a single study, necessitating integration of data across studies and differing acquisitions.

Progress in offering authoritative, centralized and standardized resources for brain connectivity mapping across the lifespan has been limited; as an example, the extension of the BIDS standard to describe atlases does not, at the moment, cover tractography templates. Standardizing tractography across the lifespan will require creating such templates, generating normative derivatives linking structural connectivity to tissue microstructure (tractometry), providing uniform method implementations, and guidelines for calibrating processing parameters.

Summary and recommendations

Despite the above challenges, if a series of recommendations are followed, tractography reconstruction and interpretation can be highly consistent and robust. Below we provide a set of recommendations towards building standardized, reliable and robust tractography data reconstruction and sharing procedures. We also discuss the feasibility of the suggested measures in terms of the effort (minimal, moderate, substantial) required to implement them.

i. Implement standard operating procedures [137] during data acquisition, i.e., written sets of instructions that specify the processes that take place during the measurement. Feasibility: a moderate effort is required to implement centralized SOPs towards tractography standardization. That said, some examples of SOPs for tractography data acquisition [138], quality assurance [58] and analysis [139] already exist (albeit, with a focus on dMRI), and could easily be adopted by the community.

ii. Adopt vendor-agnostic, open-source pulse sequence design and reconstruction frameworks (e.g., Pulseq [140, 141]) to facilitate more similar results across the instruments of different vendors. Feasibility: a substantial effort is required to make vendor-agnostic pulse sequences extensive within the data acquisition hardware, as it involves vendor support and validation towards regulatory clearance.

iii. Advance BIDS-compliant standards for tractography inputs and outputs. Akin to a wide range of neuroimaging use-cases, including human electrophysiology and microscopy, where BIDS has demonstrated its utility, a tractography-specific extension is needed towards reliable, reproducible, and robust tractography data representation and sharing. Note that the particular adoption of the BIDS specification for tractography should not prevent software from processing isolated tractography files. Feasibility: As of writing of this article, the BIDS tractography extension is being drafted, and a small effort is needed to complete it.

iv. Quality control. Standardized quality control procedures need to be fostered including automated procedures at every stage of processing. Further research on the impact of different decisions in QC is needed, to ultimately develop guidelines for best practices. Feasibility: due to the challenges inherent to tractography validation, a moderate effort will be required to define consensus measures and procedures towards implementing standardized QC protocols and pipelines.

v. Make analysis tools that are flexible to be used across different data acquisition methods, different species and different settings. FAIR-Software principles can be applied, to make sure that methods are transparent, rigorously designed and available to the community [142]. Feasibility: a substantial effort in validation is required to generalize tools across methods, species and settings. This may include generating publicly shared, reproducible records of the datasets and procedures employed.

vi. Scalability. Software and platforms for sharing and computing on brain connectivity data need to be built with the large datasets of the future in mind, and with the ability to scale to much larger data than is currently available, in anticipation of the inevitable deluge of data that is expected to occur. Infrastructure for distributed and cloud computing includes evolving standards for representation of large array-based [143], tabular [144], and even trajectory data [145] in these kinds of systems, and formats for representation of tractography data could be adapted to capitalize on these developments. Feasibility: a moderate effort can be expected to design frameworks capable of handling large volumes of data. This effort is related to the development of data representation standards inherent to tractography, and the reliance on the development of general purpose, advanced scientific software.

vii. Create and share annotated datasets for machine learning training. For improved interoperability and reproducibility, use industry standards for machine learning methods, such as ONNX and widely used sharing platforms for ML/AI models and tools, such as Hugging Face. Feasibility: These both require minor efforts on the side of scientists and researchers who develop these methods.

viii. Future-proof standardized file formats. The community needs to advance new file formats that address the needs for consistent explicit tractography data spatial representation, while also supporting the needs for new large-scale datasets. This includes standardized, traceable, openly available, and well-documented specifications, including community-sustained conversion information and tools. We identify the nascent TRX file-format as a format that has the potential to address many of the issues raised here, specifically because it was developed taking

these aspects into consideration. Feasibility: a minor effort remains to be done in terms of documentation of the TRX file format. A few initial studies already provide evidence for the suitability of TRX for reliable, and robust tractography data representation [105, 146]. A moderate effort will be required in order to incorporate the format as the output of existing software pipelines, which would make it more widely used.

ix. Prioritize the articulation of clearly specified validation frameworks with common benchmarks and metrics, alongside the development of centralized, consensus evaluation tools. Importantly, these will enable unambiguous assessment of relevant aspects like heterogeneous scanners, acquisition protocols, and imaging sources, critical to assess reliability and robustness of tractography. Feasibility: a substantial effort will be required to specify the resources —including data, metrics, and technological requirements— and to operationalize and sustain the resulting infrastructure.

x. Build the bridges between research and clinical tractography: Delivery of advanced methods into clinical practice can be facilitated by standardizing workflows, and by increasing the interoperability between different parts of the clinical informatics infrastructure. For example, via integration of visualization into surgery image-guided systems, into Picture Archiving and Communication Systems (PACS) used in clinical, and into electronic medical records [147]. This will also set the scene to improve the bench-to-bedside pipeline of new computational methods. Feasibility: while research and clinical requirements remain different, anatomically refined tractography methods can reach clinical practice through rigorous, coordinated validation and collaboration among researchers, clinicians, and vendors.

Data availability

Not applicable.

Availability of Source Code and Requirements

Project name: Standardization-Position-Paper; Project homepage: https://github.com/International-Society-for-Tractography/Standardization-Position-Paper; Operating system: Not applicable; Programming language: Python; Other requirements: The data used to generate these plots are publicly available at https://osf.io/qcm7a/overview; License: Apache-2.0

Declarations

List of abbreviations

3D: three-dimensional; AI: artificial intelligence; BIDS: Brain Imaging Data Structure; CCF: Common Coordinate Framework; DICOM: Digital Imaging and Communications in Medicine; dMRI: diffusion magnetic resonance imaging; FAIR: Findable, Accessible, Interoperable, Reusable; GB: gigabyte; GPU: Graphics Processing Unit; ISMRM: International Society for Magnetic Resonance in Medicine; IST: International Society for Tractography; LPS: Left, Posterior, Superior; ML: machine learning; MRI: magnetic resonance imaging; PACS: Picture Archiving and Communication Systems; QC: quality control; RAS: Right, Anterior, Superior; SOP: standard operating procedures; TB: terabyte; TRX: TRactography eXchange

Ethical Approval

Not applicable.

Consent for publication

Not applicable.

Competing Interests

S.S. is an employee of ASG Superconductors; M.D. is shareholder at Imeka Solutions Inc.

Funding

W.T. is supported by the National Institute of Biomedical Imaging and Bioengineering (NIBIB) grant 2R01EB027585-04A1; K.S. is supported by grant NIH K01EB032898; M.C. is supported through NIH grants 2R01MH112847, 2R01MH120482, and 2R01MH113550; A.D.L. is supported by a Starting Grant from the European Research Council (agreement 101163214), the Galen and Hilary Weston foundation, and Stichting Hanarth Fonds; J.K. is supported by NSF Graduate Research Fellowship DGE-2140004; F.R. acknowledges support of the Natural Sciences and Engineering Research Council of Canada (NSERC); S.N.S is supported by a European Research Council Consolidator Grant (101000969); F.P. is supported by Wellcome Trust (grant no. 226486/Z/22/Z, Principal Investigator F. Pestilli), NINDS UM1NS132207, BRAIN CONNECTS: Center for Mesoscale Connectomics (Principal Investigator K. Ugurbil), and NINDS U24NS140384, BRAIN CONNECTS: The Axonal Projectome EXchange (APEX) (Principal Investigator F. Pestilli); J.Y.-M.Y acknowledges position funding support from the Royal Children's Hospital Foundation (RCHF 2022-1402 and RCHF 2025-1621), and support from The Kids' Cancer Project (TKCP) Col Reynolds Fellowship; M.D. is supported by the Université de Sherbrooke research chair in Neuroinformatics; S.H. is supported by grant NIH UM1NS132207; A.R.'s work is funded by National Institutes of Health grants MH121868, MH121867, R25MH112480, R01AG060942, and U19AG066567, and R01EB027585, as well as by National Science Foundation grants 1934292 and 2334483, and by the Chan Zuckerberg Initiative's Essential Open Source Software for Science program.

Author's Contributions

J.H.L., F.R., and A.R. developed the code that served as the basis for the plots in Panels a, c, d and e in Figure 2. All authors discussed the content, reviewed and wrote the paper.

Acknowledgements

Not applicable.

ORCiD information

Jon Haitz Legarreta: 0000-0002-9661-1396; Simona Schiavi: 0000-0003-1641-186X; Wei Tang: 0000-0003-3550-4076; Garrett Banks: 0000-0001-6292-977X; Matthew Cieslak: 0000-0002-1931-4734; Kurt Schilling: 0000-0003-3686-7645; Alberto De Luca: 0000-0002-2553-7299; Jacques-Donald Tournier: 0000-0001-5591-7383; John Kruper: 0000-0003-0081-391X; Francois Rheault: 0000-0002-0097-8004; Stamatios N. Sotiropoulos: 0000-0003-4735-5776; Franco Pestilli: 0000-0002-2469-0494; Jelle Veraart: 0000-0003-0781-0420; Joseph Yuan-Mou Yang: 0000-0003-4081-7157; Maxime Descoteaux: 0000-0002-8191-2129; Sarah Heilbronner: 0000-0003-0893-5364; Ariel Rokem: 0000-0003-0679-1985

References

1. Thiebaut de Schotten M, Forkel SJ. The emergent properties of the connected brain. Science (New York, NY) 2022;378(6619):505–510. http://dx.doi.org/10.1126/science.abq2591.
2. Fields RD. White matter in learning, cognition and psychiatric disorders. Trends in neurosciences 2008;31(7):361–370. http://dx.doi.org/10.1016/j.tins.2008.04.001.
3. Passingham RE, Stephan KE, Kötter R. The anatomical basis of functional localization in the cortex. Nature reviews Neuroscience 2002;3(8):606–616. http://dx.doi.org/10.1038/nrn893.
4. Wandell BA. Clarifying Human White Matter. Annual review of neuroscience 2016 1 Apr;39:103–128. http://dx.doi.org/10.1146/annurev-neuro-070815-013815.
5. Jbabdi S, Sotiropoulos SN, Haber SN, Van Essen DC, Behrens TE. Measuring macroscopic brain connections in vivo. Nat Neurosci 2015 Nov;18(11):1546–1555.
6. Schmahmann JD, Petrides M, Pandya DN. Fiber pathways of the brain. New York, NY: Oxford University Press; 2006.
7. Axer M, Strohmer S, Gräßel D, Bücker O, Dohmen M, Reckfort J, et al. Estimating fiber orientation distribution functions in 3D-Polarized Light Imaging. Frontiers in neuroanatomy 2016 19 Apr;10:40. http://dx.doi.org/10.3389/fnana.2016.00040.
8. Lefebvre J, Delafontaine-Martel P, Pouliot P, Girouard H, Descoteaux M, Lesage F. Fully automated dual-resolution serial optical coherence tomography aimed at diffusion MRI validation in whole mouse brains. Neurophotonics 2018 Oct;5(4):045004. http://dx.doi.org/10.1117/1.NPh.5.4.045004.
9. Menzel M, Gräßel D, Rajkovic I, Zeineh MM, Georgiadis M. Using light and X-ray scattering to untangle complex neuronal orientations and validate diffusion MRI. eLife 2023 11 May;12. http://dx.doi.org/10.7554/eLife.84024.
10. Mollink J, Kleinnijenhuis M, van Cappellen van Walsum AM, Sotiropoulos SN, Cottaar M, Mirfin C, et al. Evaluating fibre orientation dispersion in white matter: Comparison of diffusion MRI, histology and polarized light imaging. NeuroImage 2017 15 Aug;157:561–574. http://dx.doi.org/10.1016/j.neuroimage.2017.06.001.
11. Schurr R, Mezer AA. The glial framework reveals white matter fiber architecture in human and primate brains. Science (New York, NY) 2021 5 Nov;374(6568):762–767. http://dx.doi.org/10.1126/science.abj7960.
12. Wang H, Lenglet C, Akkin T. Structure tensor analysis of serial optical coherence scanner images for mapping fiber orientations and tractography in the brain. Journal of biomedical optics 2015 Mar;20(3):036003. http://dx.doi.org/10.1117/1.JBO.20.3.036003.
13. Xu F, Shen Y, Ding L, Yang CY, Tan H, Wang H, et al. High-throughput mapping of a whole rhesus monkey brain at micrometer resolution. Nature biotechnology 2021 26 Dec;39(12):1521–1528. http://dx.doi.org/10.1038/s41587-021-00986-5.
14. Kjer HM, Andersson M, He Y, Pacureanu A, Daducci A, Pizzolato M, et al. Bridging the 3D geometrical organisation of white matter pathways across anatomical length scales and species. eLife 2025 28 Feb;13. http://dx.doi.org/10.7554/eLife.94917.
15. Girard G, Rafael-Patiño J, Truffet R, Aydogan DB, Adluru N, Nair VA, et al. Tractography passes the test: Results from the diffusion-simulated connectivity (disco) challenge. NeuroImage 2023 15 Aug;277(120231):120231. http://dx.doi.org/10.1016/j.neuroimage.2023.120231.
16. Maffei C, Girard G, Schilling KG, Aydogan DB, Adluru N, Zhylka A, et al. Insights from the IronTract challenge: Optimal methods for mapping brain pathways from multi-shell diffusion MRI. NeuroImage 2022 15 Aug;257(119327):119327. http://dx.doi.org/10.1016/j.neuroimage.2022.119327.
17. Maier-Hein KH, Neher PF, Houde JC, Côté MA, Garyfallidis E, Zhong J, et al. The challenge of mapping the human connectome based on diffusion tractography. Nature communications 2017 7 Nov;8(1):1349. http://dx.doi.org/10.1038/s41467-017-01285-x.
18. Schilling KG, Grussu F, Ianus A, Hansen B, Howard AFD, Barrett RLC, et al., Considerations and recommendations from the ISMRM Diffusion Study Group for preclinical diffusion MRI: Part 2 – Ex vivo imaging: added value and acquisition; 2022. http://arxiv.org/abs/2209.13371.
19. Rheault F, De Benedictis A, Daducci A, Maffei C, Tax CMW, Romascano D, et al. Tractostorm: The what, why, and how of tractography dissection reproducibility. Human brain mapping 2020;41(7):1859–1874. http://dx.doi.org/10.1002/hbm.24917.
20. Schilling KG, Rheault F, Petit L, Hansen CB, Nath V, Yeh FC, et al. Tractography dissection variability: What happens when 42 groups dissect 14 white matter bundles on the same dataset? NeuroImage 2021 1 Nov;243(118502):118502. http://dx.doi.org/10.1016/j.neuroimage.2021.118502.
21. Casey BJ, Cannonier T, Conley MI, Cohen AO, Barch DM, Heitzeg MM, et al. The Adolescent Brain Cognitive Development (ABCD) study: Imaging acquisition across 21 sites. Dev Cogn Neurosci 2018 Aug;32:43–54.
22. Cieslak M, Irfanoglu MO, Meisler SL, Salo T, Raikes A, Cook PA, et al. Diffusion MRI processing in the HEALthy Brain and child development study: Innovations and applications. bioRxiv 2025 Nov;p. 2025.11.10.687672.
23. Alexander LM, Escalera J, Ai L, Andreotti C, Febre K, Mangone A, et al. An open resource for transdiagnostic research in pediatric mental health and learning disorders. Sci Data 2017 Dec;4:170181.
24. Richie-Halford A, Cieslak M, Ai L, Caffarra S, Covitz S, Franco AR, et al. An analysis-ready and quality controlled resource for pediatric brain white-matter research. Scientific data 2022 12 Oct;9(1):616. http://dx.doi.org/10.1038/s41597-022-01695-7.
25. Alfaro-Almagro F, Jenkinson M, Bangerter NK, Andersson JLR, Griffanti L, Douaud G, et al. Image processing and Quality Control for the first 10,000 brain imaging datasets from UK Biobank. Neuroimage 2018 Feb;166:400–424.
26. Wilkinson MD, Dumontier M, Aalbersberg IJJ, Appleton G, Axton M, Baak A, et al. The FAIR Guiding Principles for scientific data management and stewardship. Scientific data 2016 15 Mar;3:160018. http://dx.doi.org/10.1038/sdata.2016.18.
27. International Society for Tractography (IST);. https://tractography.io/, accessed: 2026-03-19.
28. Jelescu IO, Grussu F, Ianus A, Hansen B, Barrett RLC, Aggarwal M, et al., Considerations and Recommendations from the ISMRM Diffusion Study Group for preclinical diffusion MRI: Part 1 – In vivo small-animal imaging; 2022. http://arxiv.org/abs/2209.12994.
29. Schilling KG, Howard AFD, Grussu F, Ianus A, Hansen B, Barrett RLC, et al., Considerations and recommendations from the ISMRM Diffusion Study Group for preclinical diffusion MRI: Part 3 – Ex vivo imaging: data processing, comparisons with microscopy, and tractography; 2024. http://arxiv.org/abs/2411.05021.
30. ISMRM Diffusion Study Group;. https://groups.ismrm.org/diffusion/, accessed: 2026-03-19.
31. International Society for Magnetic Resonance in Medicine (ISMRM);. https://www.ismrm.org/, accessed: 2026-03-19.
32. Barba LA. Terminologies for Reproducible Research. arXiv [csDL] 2018 Feb;.
33. Kruper J, Yeatman JD, Richie-Halford A, Bloom D, Grotheer M, Caffarra S, et al. Evaluating the Reliability of Human Brain

White Matter Tractometry. Apert Neuro 2021 Nov;1(1).

34. The Turing Way;. Accessed: 2026-3-3. https://book.the-turing-way.org/reproducible-research/overview/overview-definitions/.

35. Mustra M, Delac K, Grgic M. Overview of the DICOM standard. In: 2008 50th International Symposium ELMAR, vol. 1 Zadar, Croatia: IEEE; 2008. p. 39–44. https://ieeexplore.ieee.org/abstract/document/4747434/.

36. Brain Imaging Data Structure (BIDS);. https://bids.neuroimaging.io/, accessed: 2026-03-19.

37. Gorgolewski KJ, Auer T, Calhoun VD, Cameron Craddock R, Das S, Duff EP, et al. The brain imaging data structure, a format for organizing and describing outputs of neuroimaging experiments. Scientific data 2016 21 Jun;3(1):1–9.

38. Gholam J, Szczepankiewicz F, Tax CMW, Mueller L, Kopanoglu E, Nilsson M, et al., aDWI-BIDS: an extension to the brain imaging data structure for advanced diffusion weighted imaging; 2021. http://arxiv.org/abs/2103.14485.

39. Pestilli F, Poldrack R, Rokem A, Satterthwaite T, Feingold F, Duff E, et al. A community-driven development of the brain imaging data standard (BIDS) to describe macroscopic brain connections 2021;.

40. Cai LY, Yang Q, Kanakaraj P, Nath V, Newton AT, Edmonson HA, et al. MASiVar: Multisite, multiscanner, and multisubject acquisitions for studying variability in diffusion weighted MRI. Magnetic resonance in medicine 2021 1 Dec;86(6):3304–3320. http://dx.doi.org/10.1002/mrm.28926.

41. Gajwani M, Oldham S, Pang JC, Arnatkevičiūtė A, Tiego J, Bellgrove MA, et al. Can hubs of the human connectome be identified consistently with diffusion MRI? Network neuroscience (Cambridge, Mass) 2023 22 Dec;7(4):1326–1350. http://dx.doi.org/10.1162/netn_a_00324.

42. Schilling KG, Tax CMW, Rheault F, Hansen C, Yang Q, Yeh FC, et al. Fiber tractography bundle segmentation depends on scanner effects, vendor effects, acquisition resolution, diffusion sampling scheme, diffusion sensitization, and bundle segmentation workflow. NeuroImage 2021 15 Nov;242:118451. http://dx.doi.org/10.1016/j.neuroimage.2021.118451.

43. Tong Q, He H, Gong T, Li C, Liang P, Qian T, et al. Reproducibility of multi-shell diffusion tractography on traveling subjects: A multicenter study prospective. Magnetic resonance imaging 2019 1 Jun;59:1–9. http://dx.doi.org/10.1016/j.mri.2019.02.011.

44. Warrington S, Torchi A, Mougin O, Campbell J, Ntata A, Craig M, et al. A multi-site, multi-modal travelling-heads resource for brain MRI harmonisation. Sci Data 2025 Apr;12(1):609.

45. Pinto MS, Paolella R, Billiet T, Van Dyck P, Guns PJ, Jeurissen B, et al. Harmonization of Brain Diffusion MRI: Concepts and Methods. Front Neurosci 2020 May;14:396.

46. Moyer D, Ver Steeg G, Tax CMW, Thompson PM. Scanner invariant representations for diffusion MRI harmonization. Magnetic resonance in medicine 2020 1 Oct;84(4):2174–2189. http://dx.doi.org/10.1002/mrm.28243.

47. Ning L, Bonet-Carne E, Grussu F, Sepehrband F, Kaden E, Veraart J, et al. Muti-shell diffusion MRI harmonisation and enhancement challenge (MUSHAC): Progress and results. In: Computational Diffusion MRI Mathematics and visualization, Cham: Springer International Publishing; 2019.p. 217–224.

48. Tax CM, Grussu F, Kaden E, Ning L, Rudrapatna U, John Evans C, et al. Cross-scanner and cross-protocol diffusion MRI data harmonisation: A benchmark database and evaluation of algorithms. NeuroImage 2019 15 Jul;195:285–299. http://dx.doi.org/10.1016/j.neuroimage.2019.01.077.

49. Warrington S, Ntata A, Mougin O, Campbell J, Torchi A, Craig M, et al. A resource for development and comparison of multimodal brain 3T MRI harmonisation approaches. Imaging Neurosci (Camb) 2023 Nov;1:1–27.

50. Magdoom KN, Avram AV, Sarlls JE, Dario G, Basser PJ. A novel framework for in-vivo diffusion tensor distribution MRI of the human brain. NeuroImage 2023 05;271:120003.

51. Yang Q, Shomal-Zadeh F, Gholipour A, Harmonization in Magnetic Resonance Imaging: A Survey of Acquisition, Image-level, and Feature-level Methods; 2025. https://arxiv.org/abs/2507.16962.

52. Human Connectome Project (HCP);. https://www.humanconnectome.org/, accessed: 2026-03-19.

53. Adolescent Brain Cognitive Development (ABCD) study;. https://abcdstudy.org/, accessed: 2026-03-19.

54. HEALthy Brain and Child Development (HBCD) Study;. https://hbcdstudy.org/, accessed: 2026-03-19.

55. The Human Connectome PHantom (HCPh) study, Standard Operating Procedures;. https://www.axonlab.org/hcph-sops/, accessed: 2026-03-19.

56. Esteban O, Birman D, Schaer M, Koyejo OO, Poldrack RA, Gorgolewski KJ. MRIQC: Advancing the automatic prediction of image quality in MRI from unseen sites. PloS one 2017 25 Sep;12(9):e0184661. http://dx.doi.org/10.1371/journal.pone.0184661.

57. Bastiani M, Cottaar M, Fitzgibbon SP, Suri S, Alfaro-Almagro F, Sotiropoulos SN, et al. Automated quality control for within and between studies diffusion MRI data using a non-parametric framework for movement and distortion correction. NeuroImage 2019 1 Jan;184:801–812. http://dx.doi.org/10.1016/j.neuroimage.2018.09.073.

58. Hagen MP, Provins C, MacNicol E, Li J, Gomez T, Garcia M, et al., Quality assessment and control of unprocessed anatomical, functional, and diffusion MRI of the human brain using MRIQC; 2024.

59. Cieslak M, Cook PA, He X, Yeh FC, Dhollander T, Adebimpe A, et al. QSIPrep: an integrative platform for preprocessing and reconstructing diffusion MRI data. Nature methods 2021;18(7):775–778. http://dx.doi.org/10.1038/s41592-021-01185-5.

60. Cirstian R, Forde NJ, Andersson JLR, Sotiropoulos SN, Beckmann CF, Marquand AF. Objective QC for diffusion MRI data: Artefact detection using normative modelling. Imaging Neuroscience 2024 26 Apr;2:1–14. https://dx.doi.org/10.1162/imag_a_00144.

61. Smith RE, Tournier JD, Calamante F, Connelly A. SIFT: Spherical-deconvolution informed filtering of tractograms. NeuroImage 2013 15 Feb;67:298–312. http://dx.doi.org/10.1016/j.neuroimage.2012.11.049.

62. Smith RE, Tournier JD, Calamante F, Connelly A. SIFT2: Enabling dense quantitative assessment of brain white matter connectivity using streamlines tractography. NeuroImage 2015 1 Oct;119:338–351. http://dx.doi.org/10.1016/j.neuroimage.2015.06.092.

63. Daducci A, Palù AD, Lemkaddem A, Thiran J. COMMIT: Convex optimization modeling for microstructure informed tractography. IEEE transactions on medical imaging 2015 1 Jan;34:246–257. http://dx.doi.org/10.1109/TMI.2014.2352414.

64. Schiavi S, Ocampo-Pineda M, Barakovic M, Petit L, Descoteaux M, Thiran JP, et al. A new method for accurate in vivo mapping of human brain connections using microstructural and anatomical information. Science Advances 2020 31 Jul;6(31). http://dx.doi.org/10.1126/sciadv.aba8245.

65. Caiafa CF, Pestilli F. Multidimensional encoding of brain connectomes. Scientific reports 2017 13 Sep;7(1):11491. http://dx.doi.org/10.1038/s41598-017-09250-w.

66. Pestilli F, Yeatman JD, Rokem A, Kay KN, Wandell BA. Evaluation and statistical inference for human connectomes. Nature methods 2014 Oct;11(10):1058–1063. http://dx.doi.org/10.1038/nmeth.3098.

67. Astolfi P, Verhagen R, Petit L, Olivetti E, Sarubbo S, Masci J, et al. Supervised tractogram filtering using Geometric Deep Learning. Medical image analysis 2023

1 Dec;90(102893):102893. http://dx.doi.org/10.1016/j.media.2023.102893.

68. Legarreta JH, Petit L, Rheault F, Theaud G, Lemaire C, Descoteaux M, et al. Filtering in Tractography using Autoencoders (FINTA). Medical image analysis 2021 7 Jun;p. 102126. http://dx.doi.org/10.1016/j.media.2021.102126.
69. Sarwar T, Ramamohanarao K, Zalesky A. A critical review of connectome validation studies. NMR in biomedicine 2021 1 Dec;34(12):e4605. http://dx.doi.org/10.1002/nbm.4605.
70. Sarwar T, Ramamohanarao K, Daducci A, Schiavi S, Smith RE, Zalesky A. Evaluation of tractogram filtering methods using human-like connectome phantoms. NeuroImage 2023 1 Nov;281(120376):120376. http://dx.doi.org/10.1016/j.neuroimage.2023.120376.
71. Zalesky A, Sarwar T, Ramamohanarao K. A cautionary note on the use of SIFT in pathological connectomes. Magnetic resonance in medicine 2020 1 Mar;83(3):791–794. http://dx.doi.org/10.1002/mrm.28037.
72. Laamoumi M, Hendriks T, Chamberland M. A taxonomic guide to diffusion MRI tractography visualization tools. NMR in Biomedicine 2025 7 Jan;38(1):e5267. http://dx.doi.org/10.1002/nbm.5267.
73. OpenNeuro;. https://openneuro.org/, accessed: 2026-03-19.
74. Zenodo;. https://zenodo.org/, accessed: 2026-03-19.
75. Fillard P, Descoteaux M, Goh A, Gouttard S, Jeurissen B, Malcolm J, et al. Quantitative evaluation of 10 tractography algorithms on a realistic diffusion MR phantom. NeuroImage 2011 05;56(1):220–234.
76. Girard G, no JRP, Truffet R, Aydogan DB, Adluru N, Nair VA, et al. Tractography passes the test: Results from the diffusion-simulated connectivity (disco) challenge. NeuroImage 2023 08;277:120231. https://www.sciencedirect.com/science/article/pii/S1053811923003828.
77. Glen DR, Taylor PA, Buchsbaum BR, Cox RW, Reynolds RC. Beware (surprisingly common) left-right flips in your MRI data: An efficient and robust method to check MRI dataset consistency using AFNI. Frontiers in neuroinformatics 2020 25 May;14:18. http://dx.doi.org/10.3389/fninf.2020.00018.
78. Brain Imaging Data Structure (BIDS) specification, Coordinate systems;. https://bids-specification.readthedocs.io/en/stable/appendices/coordinate-systems.html, accessed: 2026-03-19.
79. Lanciego JL, Wouterlood FG. Neuroanatomical tract-tracing techniques that did go viral. Brain Struct Funct 2020 May;225(4):1193–1224.
80. Large-scale Imaging of Neural Circuits (LINC), BRAIN CONNECTS center;. https://connects.mgh.harvard.edu/, accessed: 2026-03-19.
81. Center for mesoscale connectomics (CMC);. https://www.mesoscale-connectivity.org, accessed: 2026-03-19.
82. Milham MP, Ai L, Koo B, Xu T, Amiez C, Balezeau F, et al. An open resource for non-human primate imaging. Neuron 2018 10 Oct;100(1):61–74.e2. http://dx.doi.org/10.1016/j.neuron.2018.08.039.
83. Hata J, Nakae K, Tsukada H, Woodward A, Haga Y, Iida M, et al. Multi-modal brain magnetic resonance imaging database covering marmosets with a wide age range. Scientific data 2023 27 Apr;10(1):221. http://dx.doi.org/10.1038/s41597-023-02121-2.
84. Paxinos G, Huang XF, Toga AW. The Rhesus Monkey Brain in Stereotaxic Coordinates. 1 ed. San Diego, CA: Academic Press; 1999. ISBN: 9780128158524.
85. Desikan RS, Ségonne F, Fischl B, Quinn BT, Dickerson BC, Blacker D, et al. An automated labeling system for subdividing the human cerebral cortex on MRI scans into gyral based regions of interest. NeuroImage 2006 1 Jul;31(3):968–980. http://dx.doi.org/10.1016/j.neuroimage.2006.01.021.
86. Mai JK, Majtanik M. Myeloarchitectonic maps of the human cerebral cortex registered to surface and sections of a standard atlas brain. Translational neuroscience 2023 1 Jan;14(1):20220325. http://dx.doi.org/10.1515/tnsci-2022-0325.
87. Petrides M, Tomaiuolo F, Yeterian EH, Pandya DN. The prefrontal cortex: comparative architectonic organization in the human and the macaque monkey brains. Cortex; a journal devoted to the study of the nervous system and behavior 2012 1 Jan;48(1):46–57. http://dx.doi.org/10.1016/j.cortex.2011.07.002.
88. Lu Y, Cui Y, Cao L, Dong Z, Cheng L, Wu W, et al. Macaque Brainnetome Atlas: A multifaceted brain map with parcellation, connection, and histology. Science Bulletin 2024;69(14):2241–2259. 07.
89. Zhang Z, Zhu T, Guo J, Ouyang M, Sousa A, Levine J, et al. Common coordinate framework of developmental macaque brain from birth to early childhood based on ultra-high-resolution diffusion MRI. In: 34th Annual Conference & Exhibition of the International Society for Magnetic Resonance in Medicine (ISMRM) Honolulu, HI USA: International Society for Magnetic Resonance in Medicine (ISMRM); 2025. Program #3654.
90. Warrington S, Thompson E, Bastiani M, Dubois J, Baxter L, Slater R, et al. Concurrent mapping of brain ontogeny and phylogeny within a common space: Standardized tractography and applications. Science advances 2022 21 Oct;8(42):eabq2022. http://dx.doi.org/10.1126/sciadv.abq2022.
91. Coizet V, Heilbronner SR, Carcenac C, Mailly P, Lehman JF, Savasta M, et al. Organization of the anterior limb of the internal capsule in the rat. The Journal of neuroscience: the official journal of the Society for Neuroscience 2017 8 Mar;37(10):2539–2554. http://dx.doi.org/10.1523/JNEUROSCI.3304-16.2017.
92. Martin RF, Bowden DM. A Stereotaxic Template Atlas of the Macaque Brain for Digital Imaging and Quantitative Neuroanatomy. NeuroImage 1996 10;4(2):119–150.
93. The Allen Brain Institute, Allen Brain Atlas: Allen Mouse Brain Common Coordinate Framework (CCF);. https://atlas.brain-map.org/, accessed: 2026-03-19.
94. Lein ES, Hawrylycz MJ, Ao N, Ayres M, Bensinger A, Bernard A, et al. Genome-wide atlas of gene expression in the adult mouse brain. Nature 2007 11 Jan;445(7124):168–176. http://dx.doi.org/10.1038/nature05453.
95. Wang Q, Ding SL, Li Y, Royall J, Feng D, Lesnar P, et al. The Allen Mouse Brain Common Coordinate Framework: A 3D reference atlas. Cell 2020 14 May;181(4):936–953.e20. http://dx.doi.org/10.1016/j.cell.2020.04.007.
96. Kremer JR, Mastronarde DN, McIntosh JR. Computer visualization of three-dimensional image data using IMOD. Journal of structural biology 1996 1 Jan;116(1):71–76. http://dx.doi.org/10.1006/jsbi.1996.0013.
97. Zhang Y, Song T, Yang CY, Shen Y, Yang Y, Hu X, et al. Whole-brain reconstruction of fiber tracts based on cytoarchitectonic organization. Nat Methods 2025 Dec;22(12):2639–2648.
98. Hayot-Sasson V, Glatard T, Rokem A. The benefits of prefetching for large-scale cloud-based neuroimaging analysis workflows. In: 2021 IEEE Workshop on Workflows in Support of Large-Scale Science (WORKS); 2021. p. 42–49.
99. Trinkle S, Foxley S, Kasthuri N, La Rivière P. Synchrotron X-ray micro-CT as a validation dataset for diffusion MRI in whole mouse brain. Magnetic resonance in medicine 2021 1 Aug;86(2):1067–1076. http://dx.doi.org/10.1002/mrm.28776.
100. Foxley S, Sampathkumar V, De Andrade V, Trinkle S, Sorokina A, Norwood K, et al. Multi-modal imaging of a single mouse brain over five orders of magnitude of resolution. NeuroImage 2021 1 Sep;238(118250):118250. http://dx.doi.org/10.1016/

j.neuroimage.2021.118250.

101. Shapson-Coe A, Januszewski M, Berger DR, Pope A, Wu Y, Blakely T, et al. A petavoxel fragment of human cerebral cortex reconstructed at nanoscale resolution. Science (New York, NY) 2024 10 May;384(6696):eadk4858. http://dx.doi.org/10.1126/science.adk4858.
102. Presseau C, Jodoin PM, Houde JC, Descoteaux M. A new compression format for fiber tracking datasets. Neuroimage 2015 Sep;109(102893):160018.
103. Rheault F, Houde JC, Descoteaux M. Visualization, interaction and tractometry: Dealing with millions of streamlines from diffusion MRI tractography. Frontiers in neuroinformatics 2017 26 Jun;11:42. http://dx.doi.org/10.3389/fninf.2017.00042.
104. Gabusi I, Battocchio M, Bosticardo S, Schiavi S, Daducci A. Blurred streamlines: A novel representation to reduce redundancy in tractography. Med Image Anal 2024 Apr;93(103101):103101.
105. Kruper J, Hagen MP, Rheault F, Crane I, Gilmore A, Narayan M, et al. Tractometry of the Human Connectome Project: resources and insights. Frontiers in neuroscience 2024 12 Jun;18:1389680. http://dx.doi.org/10.3389/fnins.2024.1389680.
106. Kraaijeveld B, Vilanova A, Chamberland M, Improved Shading and Performance using Density Volumes in Interactive Tractography Visualization. Kyoto, Japan; 2025. https://www.ismrm.org/workshops/2025/Diffusion40/, poster #181 ISMRM Workshop on 40 Years of Diffusion: Past, Present & Future Perspectives.
107. Rheault F, Hayot-Sasson V, Smith RE, Rorden C, Tournier JD, Garyfallidis E, et al. TRX: A Community-Oriented Tractography File Format. In: 28th Annual Meeting of The Organization for Human Brain Mapping (OHBM) Glasgow, Scotland; 2022.
108. Legeay S. Mesoscopic mapping of the human structural connectome using high-performance global tractography. PhD thesis, Université Paris-Saclay; 2025.
109. Kruper J, Bisson M, Romero J, Fatica M, Rokem A. GPU-accelerated Diffusion MRI Tractography in DIPY. In: International Society for Magnetic Resonance in Medicine; 2025. https://archive.ismrm.org/2025/4785_bKkDvtQEA.html.
110. Hernandez-Fernandez M, Reguly I, Jbabdi S, Giles M, Smith S, Sotiropoulos SN. Using GPUs to accelerate computational diffusion MRI: From microstructure estimation to tractography and connectomes. Neuroimage 2019 Mar;188:598–615.
111. Abernathey RP, Augspurger T, Banihirwe A, Blackmon-Luca CC, Crone TJ, Gentemann CL, et al. Cloud-Native Repositories for Big Scientific Data. Computing in Science Engineering 2021 Mar;23(2):26–35.
112. Poulin P, Jörgens D, Jodoin PM, Descoteaux M. Tractography and machine learning: Current state and open challenges. Magnetic Resonance Imaging 2019 12;64:37–48. Artificial Intelligence in MRI.
113. Karimi D, Warfield SK. Diffusion MRI with machine learning. Imaging Neuroscience 2024 11;2:imag–2–00353.
114. Neher P, Poulin P, Jörgens D, Reisert M, Benou I, Maier-Hein K. Chapter 17 - Machine learning in tractography. In: Dell'Acqua F, Descoteaux M, Leemans A, editors. Handbook of Diffusion MR Tractography - Imaging Methods, Biophysical Models, Algorithms and Applications Academic Press; 2025.p. 315–345.
115. Côté MA, Girard G, Boré A, Garyfallidis E, Houde JC, Descoteaux M. Tractometer: Towards validation of tractography pipelines. Medical Image Analysis 2013 10;17(7):844–857. Special Issue on the 2012 Conference on Medical Image Computing and Computer Assisted Intervention.
116. Bai J, Lu F, Zhang K, et al., ONNX: Open Neural Network Exchange. GitHub; 2019. https://github.com/onnx/onnx.
117. Bizzi A, Yang JYM, Aliaga-Arias J, Dell'Acqua F, Lavrador JP, Vergani F. Chapter 31 - Neurosurgical applications of clinical tractography. In: Dell'Acqua F, Descoteaux M, Leemans A, editors. Handbook of Diffusion MR Tractography : Academic Press; 2025.p. 631–652.
118. Costabile JD, Alaswad E, D'Souza S, Thompson JA, Ormond DR. Current applications of diffusion tensor imaging and tractography in intracranial tumor resection. Frontiers in oncology 2019 29 May;9:426. http://dx.doi.org/10.3389/fonc.2019.00426.
119. Vanderweyen DC, Theaud G, Sidhu J, Rheault F, Sarubbo S, Descoteaux M, et al. The role of diffusion tractography in refining glial tumor resection. Brain structure & function 2020 May;225(4):1413–1436. http://dx.doi.org/10.1007/s00429-020-02056-z.
120. Kwon HG, Hong JH, Hong CP, Lee DH, Ahn SH, Jang SH. Dentatorubrothalamic tract in human brain: diffusion tensor tractography study. Neuroradiology 2011 Oct;53(10):787–791. http://dx.doi.org/10.1007/s00234-011-0878-7.
121. Lehman VT, Lee KH, Klassen BT, Blezek DJ, Goyal A, Shah BR, et al. MRI and tractography techniques to localize the ventral intermediate nucleus and dentatorubrothalamic tract for deep brain stimulation and MR-guided focused ultrasound: a narrative review and update. Neurosurgical focus 2020 1 Jul;49(1):E8. http://dx.doi.org/10.3171/2020.4.FOCUS20170.
122. Nowacki A, Debove I, Rossi F, Schlaeppi JA, Petermann K, Wiest R, et al. Targeting the posterior subthalamic area for essential tremor: proposal for MRI-based anatomical landmarks. Journal of neurosurgery 2019 Sep;131(3):820–827. http://dx.doi.org/10.3171/2018.4.JNS18373.
123. Aylmore H, Young F, Aquilina K, Clark CA, Clayden JD. The use of intraoperative tractography in brain tumor and epilepsy surgery: a systematic review and meta-analysis. Frontiers in Neuroimaging 2025 06;4.
124. Sarubbo S, Vergani F, Yang JYM. Tractography in brain tumor surgery: current clinical impact and future challenges. Brain Structure and Function 2025;230(6):93. https://doi.org/10.1007/s00429-025-02956-y.
125. Kamagata K, Andica C, Uchida W, Takabayashi K, Saito Y, Lukies M, et al. Advancements in Diffusion MRI Tractography for Neurosurgery. Investigative Radiology 2024;59(1). https://journals.lww.com/investigativeradiology/fulltext/2024/01000/advancements_in_diffusion_mri_tractography_for.2.aspx.
126. Beyh A, Ohlerth AK, Forkel SJ. Harnessing Advanced Tractography in Neurosurgical Practice. In: Krieg SM, Picht T, editors. Navigated Transcranial Magnetic Stimulation in Neurosurgery Cham, Switzerland: Springer; 2025.p. 385–411. https://doi.org/10.1007/978-3-031-97155-6_21.
127. Jacquesson T, Frindel C, Kocevar G, Berhouma M, Jouanneau E, Attyé A, et al. Overcoming Challenges of Cranial Nerve Tractography: A Targeted Review. Neurosurgery 2019 02;84(2).
128. de Faria O Jr, Pivonkova H, Varga B, Timmler S, Evans KA, Káradóttir RT. Periods of synchronized myelin changes shape brain function and plasticity. Nat Neurosci 2021 Nov;24(11):1508–1521.
129. Knickmeyer RC, Gouttard S, Kang C, Evans D, Wilber K, Smith JK, et al. A structural MRI study of human brain development from birth to 2 years. J Neurosci 2008 Nov;28(47):12176–12182.
130. Shi F, Yap PT, Wu G, Jia H, Gilmore JH, Lin W, et al. Infant brain atlases from neonates to 1- and 2-year-olds. PLoS One 2011 Apr;6(4):e18746.
131. Sanchez CE, Richards JE, Almli CR. Neurodevelopmental MRI brain templates for children from 2 weeks to 4 years of age. Developmental psychobiology 2012;54(1):77–91.
132. Calixto C, Dorigatti Soldatelli M, Jaimes C, Pierotich L, Warfield SK, Gholipour A, et al. A detailed spatiotemporal atlas of the white matter tracts for the fetal brain. Proceedings of the National Academy of Sciences 2025 7 Jan;122(1):e2410341121.

http://dx.doi.org/10.1073/pnas.2410341121.
133. Gilmore JH, Knickmeyer RC, Gao W. Imaging structural and functional brain development in early childhood. Nat Rev Neurosci 2018 Feb;19(3):123–137.
134. Grotheer M, Rosenke M, Wu H, Kular H, Querdasi FR, Natu VS, et al. White matter myelination during early infancy is linked to spatial gradients and myelin content at birth. Nature communications 2022;13(1):1–12.
135. Guerrero JM, Adluru N, Bendlin BB, Goldsmith HH, Schaefer SM, Davidson RJ, et al. Optimizing the intrinsic parallel diffusivity in NODDI: An extensive empirical evaluation. PLoS One 2019 Sep;14(9):e0217118.
136. Chang KH, Burke L, LaPiana N, Howlett B, Hunt D, Dezelar M, et al. Free water elimination tractometry for aging brains. bioRxiv 2024 Nov;p. 2024.11.10.622861.
137. Hollmann S, Frohme M, Endrullat C, Kremer A, D'Elia D, Regierer B, et al. Ten simple rules on how to write a standard operating procedure. PLoS computational biology 2020 3 Sep;16(9):e1008095. http://dx.doi.org/10.1371/journal.pcbi.1008095.
138. Shamir I, Assaf Y. Tutorial: a guide to diffusion MRI and structural connectomics. Nat Protoc 2025 Feb;20(2):317–335.
139. Tahedl M, Tournier JD, Smith RE. Structural connectome construction using constrained spherical deconvolution in multi-shell diffusion-weighted magnetic resonance imaging. Nat Protoc 2025 Feb;p. 1–33.
140. Layton KJ, Kroboth S, Jia F, Littin S, Yu H, Leupold J, et al. Pulseq: A rapid and hardware-independent pulse sequence prototyping framework. Magn Reson Med 2017 Apr;77(4):1544–1552.
141. Liu Q, Ning L, Shaik IA, Liao C, Gagoski B, Bilgic B, et al. Reduced cross-scanner variability using vendor-agnostic sequences for single-shell diffusion MRI. Magn Reson Med 2024 Jul;92(1):246–256.
142. Barker M, Chue Hong NP, Katz DS, Lamprecht AL, Martinez-Ortiz C, Psomopoulos F, et al. Introducing the FAIR Principles for research software. Sci Data 2022 Oct;9(1):622.
143. Ambatipudi S, Byna S. A comparison of HDF5, zarr, and netCDF4 in performing common I/O operations. arXiv [csDC] 2022 Jul;.
144. Vohra D. Apache Parquet. In: Practical Hadoop Ecosystem Berkeley, CA: Apress; 2016.p. 325–335.
145. Lopez-Gomez J, Blomer J. RNTuple performance: Status and Outlook. arXiv [physicsdata-an] 2022 Apr;.
146. Kruper J, Richie-Halford A, Qiao J, Gilmore A, Chang K, Grotheer M, et al. A software ecosystem for brain tractometry processing, analysis, and insight. PLoS Comput Biol 2025 Aug;21(8):e1013323.
147. Beare R, Alexander B, Warren A, Kean M, Seal M, Wray A, et al. Karawun: a software package for assisting evaluation of advances in multimodal imaging for neurosurgical planning and intraoperative neuronavigation. Int J Comput Assist Radiol Surg 2023 Jan;18(1):171–179.

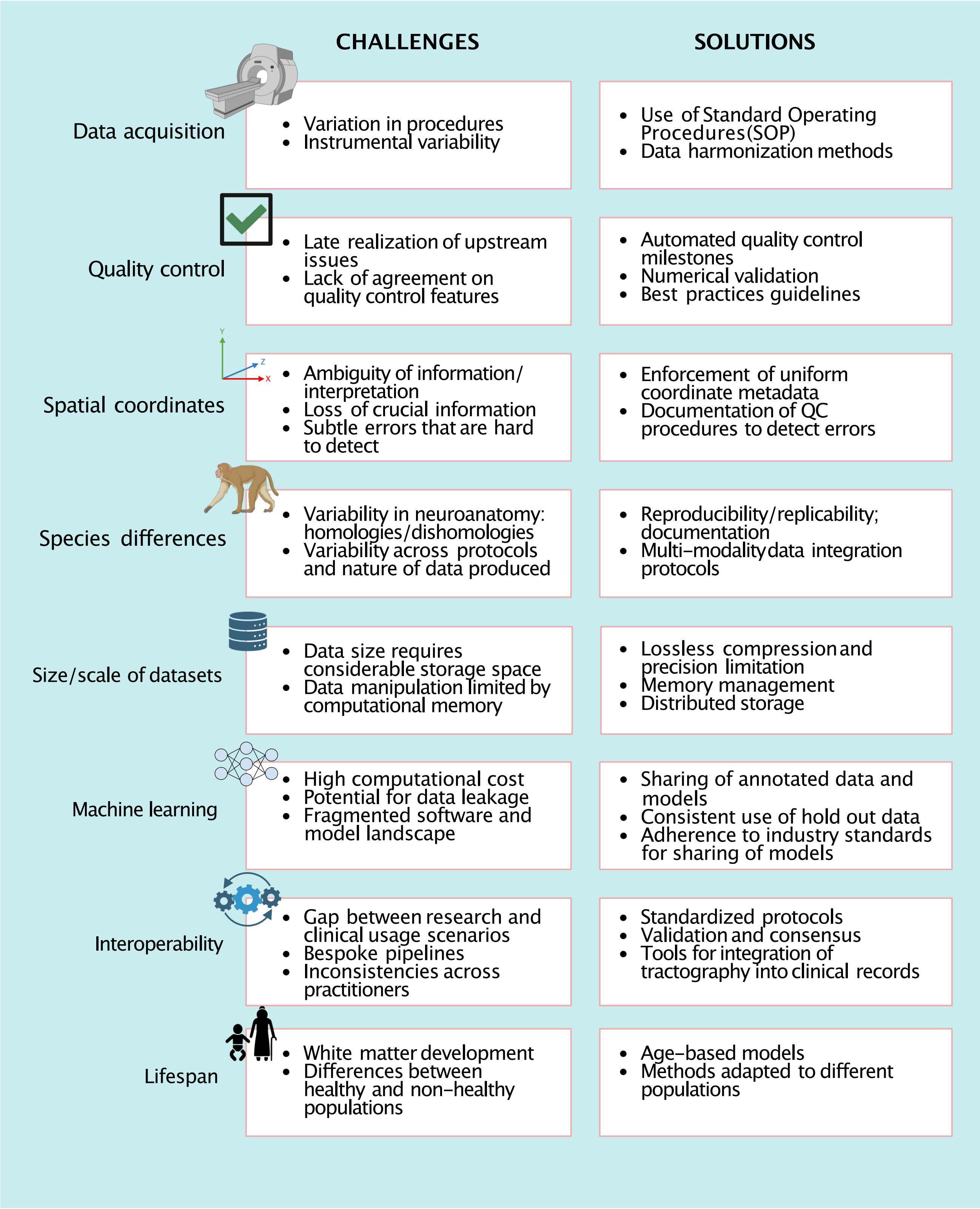

Figure 1. Summary of main challenges and suggested standardization solutions towards reliable, reproducible, and robust tractography.

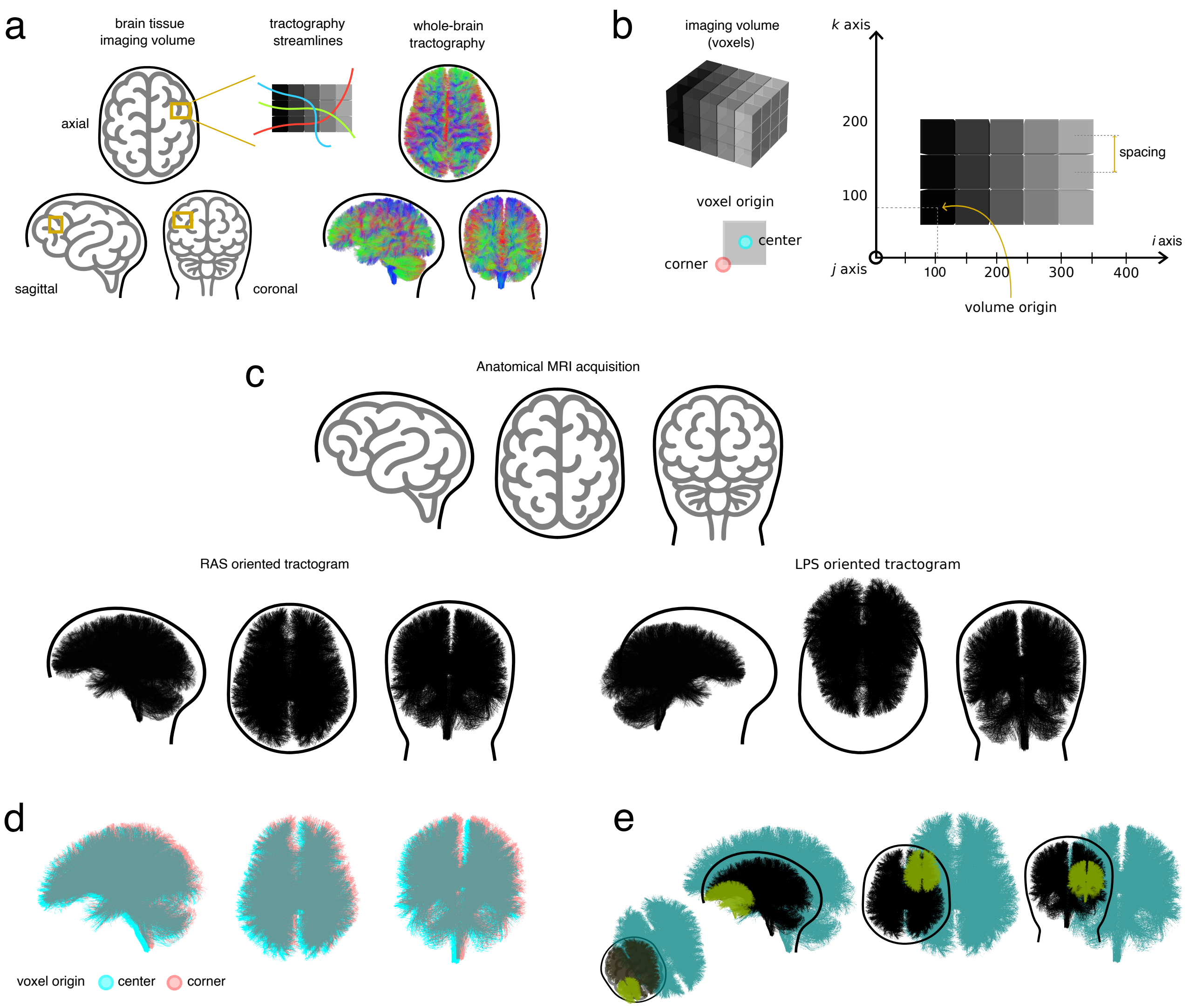

Figure 2. Tractography data representation issues. Panel a: schematic view of the process of generating tractography streamline data from an imaged brain tissue volume; Panel b: miscellaneous concepts related to tractography data; Panel c: illustration of the spatial mismatch between a tractography file natively serialized following the *LPS+* convention but the structural data (e.g., a T1-weighted MRI acquisition) being arranged following the *RAS+* convention; Panel d: effect of the voxel origin convention visualized as a half-voxel shift between the *corner* and *center* conventions on a whole-brain tractogram; Panel e: illustration of tractography data representation issues arising from mismatches between the spatial coordinate transformation matrix employed when reconstructing streamlines *vs.* the one used when serializing the tractography data, or from storing the data in voxel space *vs.* real-world space coordinates, or from inadvertently applying spatial transformations multiple times. In all cases, the tractogram in black is at the correct location, and the tractograms in green and turquoise do not lie at the correct anatomical location.
