# Supplementary material for: What needs to be standardized for reliable, reproducible, and robust tractography?
Source: Gigascience. 2026 Mar 25;15:giag034. doi: 10.1093/gigascience/giag034 (PMC13137869; doi:10.1093/gigascience/giag034)
Supplement: giag034_Authors_Response_To_Reviewer_Comments_original_submission [file giag034_authors_response_to_reviewer_comments_original_submission.pdf]

Author's Response To Reviewer Comments

Letter to Editor

Prof. Hongfang Zhang
Editor
GigaScience

Department of Radiology
Brigham and Women's Hospital
Harvard Medical School
02145 Somerville MA USA
jhlegarreta@bwh.harvard.edu

February 3, 2026
Revision to manuscript GIGA-D-25-00365

The authors would like to thank the editor and reviewers for their constructive comments and suggestions that have helped improve the quality of this manuscript. The manuscript has undergone a thorough revision according to the editor and reviewers' comments. Please see below our point-by-point responses. For the reviewers' convenience, we have highlighted changes in the revised manuscript in strikethrough red/underlined blue.

The main changes to the manuscript are as follows:
- We have extensively revised the manuscript, providing further clarifications to the points raised by the reviewers.
- We discuss additional points raised by the reviewers that contribute to a more comprehensive view of the need for tractography standardization.
- We detail actionable measures needed to support standardization throughout the manuscript, including the effort required for each of the recommendations.
- We have corrected the typos and added suggested references.

We look forward to your response.

Truly yours,
Jon Haitz Legarreta, PhD
On behalf of the co-authors

Response to Reviewers

Reviewer 1

This aim of article is threefold: introduce some of the problems related to lack of standardization for tractography, offers potential solutions, and provide recommendations for the research community relating to the solutions. The three contexts identified in the abstract are: (1) enabling reliable tract-specific analyses, (2) facilitating cross-study harmonization, and (3) supporting replicable large-scale population studies.

Tractography is a widely used MRI analysis technique, applied to both research and clinical decision-making. Owing to its complexity, results are hard to quantify and interpret, and this lack of reliability is an

important limitation.

Reviewer Comment 1.1

The aim of the article is clear. It seems to align with the journal's criteria for reviews: "critical assessments of emerging technologies [. . .] white papers on standards and policy." However, the focus is not specifically aimed at "research areas relating to the utilisation of large scale research data".

Reply:

Tractography is one of the many research fields that have seen a transition from smaller bespoke datasets to very large consortium led data acquisition efforts. At the same time, data acquisition methods are generating data at ever-higher resolution. This means that TB- and even PB-scale datasets are more prevalent. These large-scale datasets are an important motivation for the present review, as with these datasets standardization becomes not only more convenient, but mission critical.

To emphasize this issue, we added text in the "Background" section (L27-38):

These challenges have become increasingly pressing as the field has transitioned in recent years towards larger and larger datasets. This increase in data volume and complexity arises from large-scale data acquisition projects, with thousands of subjects, on the one hand. Examples of these include the Adolescent Brain Cognitive Development (ABCD) [21], Healthy Brain and Child Development (HBCD) [22], and Healthy Brain Network [23, 24] studies, as well as the UK Biobank [25], and many others. On the other, it arises from the increase in the resolution as well as coverage of measurements.
These larger data have led to the application of data-driven discovery methods, where consistent and comprehensive standards are necessary.

Reviewer Comment 1.2

There is a disconnect between the title of the review and the contents. Specifically, while the terms "reliable", "replicable", and "robust" are used in the title, they are not defined in the text and are not used prominently to organize the discussion. It would have been helpful for the authors to translate these concepts into operational terms to make the usefulness of their recommendations clearer.

Reply:

To clarify the theoretical underpinnings of our work, and to make these terms more concrete, we have provided a definition of these terms (L65-88) and in each of our sections, we describe how the standardization challenges and solutions that are proposed relate to these terms (or where they are less relevant to these terms):

Reliability, reproducibility, and robustness in tractography encompass multiple methodological dimensions. They manifest across the full data life-cycle of tractography, including acquisition, processing, and analysis. They also manifest in inferences applied across different species, different types of measurements, different ages, and across basic research and clinical application. In this context, we define reliability as the consistency of the reconstruction under fixed conditions (e.g., repeated measurements on the same participant, repeated computations of the same reconstruction method, and repeated use of the same tractography algorithm). Reproducibility (sometimes also referred to as "replicability ", see [29]) is the ability to obtain equivalent results across sites, operators, and implementations, while preserving the expected anatomical observations (e.g., consistency across software packages, versions, and hardware platforms, consistent results obtained from the same shared data, etc.). Finally, robustness characterizes the stability of tractography algorithms in the face of varying conditions (e.g., noise perturbations, multi-site or multi-vendor datasets, etc.). As we will demonstrate in addressing a range of practical and conceptual challenges, standardization is essential to achieve these goals. Therefore, as we survey a range of challenges and proposed solutions, we will highlight key factors influencing these aspects and propose ways to increase them.

Reviewer Comment 1.3

I note that in the metadata accompanying the manuscript, the authors reply “yes” to the question “Have you included all the information requested [regarding design and statistics] in your manuscript?” However, given that the format is effectively that of a narrative review the more correct answer would be “not applicable”.

Reply:

We agree with the reviewer’s comment, and have changed our answer to “Not applicable”.

Reviewer Comment 1.4

I would say many of the problems discussed are already well-known (for example, spatial coordinates). Implementation of the solutions can be difficult. It might have been useful to classify the degree of progress regarding each of the problems mentioned. Some seem easy to achieve (harmonization of data formats) while others are only a distant dream. For example, people were discussing vendor-agnostic sequences in the 1990s, but the documented progress has been limited and the limitations are not only technical but also institutional (commercial).

Reply:

We have edited each section so that they end with a paragraph that clearly describes the current state of the challenges described.

Data acquisition (L143-183):

Despite the progress that has been achieved, further work is needed to provide an adequate basis for tractography. Using BIDS for neuroimaging data sharing offers a high-level of consistency; however, its adoption across pipelines is not yet all-pervading, and its implementation is not uniform. Furthermore, although the diffusion extension proposal has been submitted for inclusion in the standard, it only covers scalar derivatives and tensors. The tractography extension has not been submitted yet for inclusion. Similarly, vendor-agnostic, standardized sequences are yet to be implemented across the variety of scanning hardware and protocols, and be validated extensively. Limited support and interest for regulatory clearance entail additional hindrance to their wide adoption [45]. Finally, harmonization is still a topic under active research, or present limitations (e.g., fixed, well populated site numbers, homogeneous populations) that have yet to be overcome. Thus, significant methodological gaps remain in diffusion MRI data acquisition that limit the development of automated and robust tractography downstream.

Standard operating procedures (SOPs) are necessary to remove variability at data collection and provide some level of reliability in tractography reconstruction from a given study. SOPs have been employed to describe the imaging and processing protocols for large-scale studies (e.g., the Human Connectome Project (HCP), the ABCD study, etc.); more recent studies (e.g., the HBCD study, or the Human Connectome PHantom Study (HCPh)) have adopted contemporary web technologies to modernize data presentation. Smaller-scale, single-site studies would likewise benefit from this practice, contributing to improved transparency in reporting and reproducibility. Similarly, standardized data and metadata records and organization (e.g., through BIDS) are required to ensure reproducibility. This includes developing the extensions to derivatives (e.g., fiber orientation distributions) that are employed to generate tractography results. Finally, harmonization solutions (within and across scanners and sites, across acquisition upgrade packages, etc.) are required to resolve data source variability. Combined with the adoption of unambiguous, self-reporting data formats (see Spatial coordinates), these measures would mitigate practical barriers and contribute to reducing overhead in multi-center studies with disparate settings.

Quality control (L230-250):

Taken together, progress has been limited in tractography QC standardization practice and adoption levels. Although there are many advanced tractography visualization tools and paradigms [51], and de- spite some pipelines offering automated reporting, their use for QC purposes has not been thoroughly studied. Similarly, validating numerical tractography QC methods is still impacted by the inability to have gold standards on real brain data, and the lack of anatomical measures defining the success of a tractography method. As a result, best practices for excluding low-quality results based on either visual or numerical reports remain insufficiently established. Reliable tractography would benefit from clearly defined, shared criteria determining the quality of results, including standardized reporting. Additionally, universally shared phantom, and synthetic data, as well as anonymized in vivo and ex vivo data from both healthy and diseased participants are required to guarantee a common ground for quality control of a given tractography pipeline. This includes sharing data in software-agnostic repositories (e.g., OpenNeuro or Zenodo). Note that this extends beyond datasets used across international challenges that quantify tractography results only within a particular dataset (see, for example, [63,17, 64, 16]).

Spatial coordinates (L310-323):

Overall, despite a well-established understanding of the coordinate frameworks involved in tractography reconstruction, current pipelines continue to rely on implicit or insufficiently documented conventions. Clear and unambiguous guidance for debugging, validating, and converting data across coordinate spaces and formats remains lacking. Consequently, meaningful progress in this area has remained limited. A tractography data standard that explicitly and unambiguously documents its spatial coordinate framework (e.g., according to the BIDS convention), and version-controlled, general resources (i.e., not tied to a particular processing package) are required. This includes testing data stored in software-agnostic repositories, and guidelines towards conformance, error checking and conversion of retrospective data.

Translation across species and methods (L421-437):

(...). As a consequence, the progress to bridge the gap in tractography data representation and software across species and imaging modalities has been limited. Addressing these challenges requires a comprehensive approach that includes standardized multi-modality and multi-resolution data acquisition, advancements in hardware for efficient in vivo scanning, essential validation studies, and methodological innovations, such as automated tools for establishing a common framework of correspondence.

While the challenges to validity and generalization are evident, these issues also raise challenges to robustness. This is because the differences in measurement tools and in analysis methods need to be thoroughly adapted and validated when they are used in species other than the ones on which they have been developed. Examples in which tractography tools that were developed for use in human dMRI data were adapted to be used in non-human data [82] suggest that there is moderate progress on this challenge.

The scale of data (L482-494):

Software and data-format enhancements have so far enabled only limited progress, due to heterogeneous precision adjustment across pipelines and partial adoption of the TRX file format. Data storage and visualization remain major bottlenecks, and significant advances have relied primarily on improvements in computational power, with efficient mapping of brain connectivity at scale being possible on supercomputing hardware [93] or using Graphics Processing Units (GPUs) [94, 95]. Distributed data storage (e.g., with zarr [96]) could offer an attractive possibility for work with very large datasets. Effective visualization of memory-intensive tractography data will require implementations in low-level programming languages, such as C++ or Rust, and/or compatibility with GPU hardware acceleration.

Integration of modern machine learning methods (L571-584):

More broadly, the development and application of machine learning methods are facing a fragmented landscape, where different software is used in different use-cases. Competition among large corporate entities (e.g., technology companies) is also driving this fragmentation, as different entities try to position

their tools as leading in the marketplace. In cases where models are shared not only as software, but also in the form of parameter values (or “weights”), the standardization of the format of model parameters, and of the metadata associated with these parameters becomes important. This is where industry standards such as the Open Neural Network Exchange (ONNX) standard [101] could play an important role. For these use-cases, there is much to benefit from the adoption of already-existing standards that apply broadly, rather than reinventing these standards for narrow applications.

Translation of tractography methods to clinical applications (L639-655):

Thus, limited progress has been made to create reference procedures to bridge basic research in tractography and clinical work. Consistent acquisition and processing protocols are required to produce tractography results that can be translated across clinical settings. In addition, methodological innovations are needed for time-constrained clinical acquisitions —such as optimized pulse sequences, undersampling strategies, and super-resolution techniques— that enable robust tractography results, alongside tractography methods that remain reliable when employed on lower-quality data. Additional measures to support clinical adoption include systematic reporting of reconstruction uncertainty across varying data quality conditions (e.g., noise levels) and reconstruction settings (e.g., seeding strategies). Finally, establishing reliable proxies for clinical outcomes is essential to validate the reliability of fiber reconstruction techniques.

Standardization of methods throughout the lifespan (L687-696):

Progress in offering authoritative, centralized and standardized resources for brain connectivity mapping across the lifespan has been limited; as an example, the extension of the BIDS standard to describe atlases does not, at the moment, cover tractography templates. Standardizing tractography across the lifespan will require creating such templates, generating normative derivatives linking structural connectivity to tissue microstructure (tractometry), providing uniform method implementations, and guidelines for calibrating processing parameters.

Additionally, we have provided some qualitative assessment related to the degree of progress of each aspect, and have a adopted a three-tier scale from limited to substantial, moderate being between them (see response to Comment 1.11).

Reviewer Comment 1.5

For a review article, the manuscript is rather brief. It is more of a summary of previous literature than a comprehensive synthesis. Likewise, the solutions offered are extremely concise. They could not be used, for example, to guide the design of a workflow aimed at acquiring and analysing multicentre DWI for tractography. It might have been useful to take one or two concrete scenarios and make specific recommendations related to these. To take an example more-or-less at random, the 2019 review of Jacquesson et al. on cranial nerve tractography synthesized previous studies and offered an opinion on emergent best practice. Although the authors may be aiming at a higher-level synthesis in the current work, their stated aim encompasses three distinct scenarios (see Scope and Rationale) and it is not clear how the recommendations might differ for each of them.

Reply:

In addressing the comments made by the reviewers, the manuscript has indeed grown in volume. Indeed, the article provides some summary of the previous literature, in addition to recommendations that appear in the section titled “Summary and recommendations”. We agree that we have not gone into the level of detail provided in the Jacquesson et al. article, regarding specific protocols. The goal of the present article is a bit broader, and we also hope to reach and interest the much broader audience of GigaScience (which includes non-neuroscientists). As you can see in the article’s byline, the authors represent a very broad range of institutions. They also come from a wide range of disciplinary backgrounds and expertise, including computer scientists, computational neuroscientists, neuroanatomists, clinicians, and

psychologists. This means that writing this article entailed seeking a broad consensus that cuts across all of these groups and disciplines. Nevertheless, despite this broad group of authors, we have managed to find common ground on many issues, and we have taken a strong stance on some practices that we consider to be important for progress on the issues that we identify.

Reviewer Comment 1.6

There is no doubt that the problems identified are real ones and this is supported by evidence in the literature. It is not made clear which are the most important in practice, either in terms of the widespread adoption of suboptimal protocols or in terms of the degree of loss in reliability or robustness. Not all of the sections appear to be equally relevant; as an example, how does “translation across species” relate to reliability, reproducibility or robustness? It seems that the main link is the framework developed by the Allen Mouse Brain Common Coordinate Framework, but this works only with mouse data. The problem of comparing concepts across species is more related to validity than reliability, and as such does not fit neatly into the topic of the manuscript.

Reply:

With the new section that defines these terms, we hope that it is now more clear how the cross-species comparisons relate to these terms. To assist the reader in making the connection, we have added the following text (L430-437):

While the challenges to validity and generalization are evident, these issues also raise challenges to robustness. This is because the differences in measurement tools and in analysis methods need to be thoroughly adapted and validated when they are used in species other than the ones on which they have been developed. Examples in which tractography tools that were developed for use in human dMRI data were adapted to be used in non-human data [82] suggest that there is moderate progress on this challenge.

We have also added standard coordinate frames that support work in non-human primate species, which should lend a broader scope to this section. The new text in the manuscript is as follows (L357-375):

This poses foremost a challenge to the validity of inferences between species. Integration between human and non-human primate data remains conceptually challenging, because non-human anatomical knowledge relies heavily on atlases based on cytoarchitectonics (e.g., [71]). These do not directly align with the common atlases used in human MRI studies (e.g., [72]). While cytoarchitectonics-based human brain atlases have been developed [73], some even with a particular stress on cross-species homology [74], these have not been integrated with widely used neuroimaging tools. At the same time, common coordinate frameworks for non-human primates are beginning to emerge (see, for example, [75]), in some cases with the explicit goal of establishing a direct relationship with the developing human connectome [76]. Tractography fingerprinting [77], i.e., using white matter bundles as latent landmarks to assess similarities and divergences across the two species, is geometry-agnostic and can provide additional solutions to the challenge of integrating cross-species coordinate frameworks.

Reviewer Comment 1.7

As stated, the solutions are plausible but some are much more readily available than others. It would have been helpful if the authors had compared the feasibility of the different solutions in terms of methods and technology available today. Sometimes, it seems as though suggestions are contradictory or at least are not uniformly applicable. On the one hand, standardization of file formats is implicit in recommendations i and iv, while on the other “the community needs to advance new file formats” (recommendation viii) that address the limitations of currently-existing formats. To follow this recommendation literally, researchers would have to delay studies until widely agreed-upon future-proofed formats were available. As another example, building bridges between research and clinical tractography (recommendation viii) sounds like a

good idea but the requirements of research and clinical scanning are different. For clinical use, MR scanners must be approved by the FDA, EU, or similar authority. Would vendor-agnostic sequences (recommendation ii) be authorised for such use?

Reply:

We have added comments on the perceived feasibility of the suggested measures (see response to Comment 1.11). In particular, we point out that only a minor effort would be needed to use future-proof file formats, and we identify that already-existing TRX file format as a good option for scientists to already use now.

A goal of the current paper is to identify the relatively wide agreement that already exists on this topic, and to encourage scientists not to wait any further.

We have remained agnostic to the data or file format chosen when recommending SOPs. Similarly, we suggest the QC protocols be developed around the idea of what aspects of tractography need to be checked rather than what file format these protocols will read.

We do not see why vendor-agnostic sequences should not get regulatory clearance if their benefits can be demonstrated and the relevant validation experiments are presented.

Reviewer Comment 1.8

The work is a useful summary of previous literature but does not, in my view offer a new understanding, or real practical guidance, to practitioners in the field. A statement like “To help bridge basic research and clinical work, acquisition and processing protocols need to be made more consistent across settings” is doubtless true, but the potential reader of the work might have hoped for more concrete guidance as to where to focus their efforts, or simply examples of cases where this has been done successfully.

Reply:

We have added explicit examples of concrete solutions required to advance tractography toward standardization, reproducibility, and robustness. We believe these additions address the reviewer’s concern regarding the lack of practical guidance in the original manuscript.

Reviewer Comment 1.9

At a basic level the work is logically organised, but it is not clear that all the sections address the problem that is nominally the object of the work. Apart from examples mentioned above, the section on “the scale of data” is highly relevant to the focus of the journal but less well-aligned with the reliability of tractography - feasibility possibly, but the authors do not make a case that acquisition or processing pipelines are not currently robust due to a data management problem.

Reply:

As mentioned above, we have added pointers throughout to the definitions of reliability, reproducibility and robustness. This includes an explanation of the robustness challenges that occur when procedures that work on data at a small scale fail to translate to large-scale data. This argument is now made on lines L455-463:

Manipulating such data volumes pose a challenge in terms of the required hardware and computational power. As imaging acquisition hardware evolves, and data storage capacity and physical memory limits increase, current analysis tools may buckle under the strain. This means that some approaches, which work well with smaller datasets, can become infeasible when translated into larger data, threatening robustness. A few technical and standardization developments may ease this strain. First, (...)

Reviewer Comment 1.10

Figure 2 may be useful for those unfamiliar with MRI acquisition but does not add much value for experienced practitioners.

Reply:

We thank the reviewer for the comment. We believe that the mentioned figure illustrates in an explicit and graphical way data formatting problems that are relevant to modern tractography research. Crucially, these issues (i) are present and pervasive across current tractography practice, researchers investing significant amounts of time dealing with them; and (ii) are only apparent at late stages (e.g., when computing tractography derivatives). The variety of software packages and versions, the atlases that are freely available in a multiplicity of formats, make these issues surface continuously, especially when (i) sharing data across research groups, or (ii) employing mixed software and pipelines that naturally work with different formats. Thus, we believe that the figure is appropriate to highlight these. We advocate for early (ideally, automated) checks to avoid these problems, avoiding to waste computational resources through early identification, and thus, allowing faster scientific throughput and discovery.

Reviewer Comment 1.11

[Are any take-home messages clear and useful for researchers and practitioners?] No, this work aims more at raising awareness about likely problems than in providing solutions for specific scenarios.

Reply:

We have incorporated this feedback and now emphasize the feasibility of each of the recommendations in the last section of the article (L706-810):

1. Implement standard operating procedures [120] during data acquisition, i.e., written sets of instructions that specify the processes that take place during the measurement. Feasibility: a moderate effort is required to implement centralized SOPs towards tractography standardization. That said, some examples of SOPs for tractography data acquisition [121], quality assurance [48] and analysis [122] already exist (albeit, with a focus on dMRI), and could easily be adopted by the community.

2. Adopt vendor-agnostic, open-source pulse sequence design and reconstruction frameworks (e.g., Pulseq [123, 124]) to facilitate more similar results across the instruments of different vendors. Feasibility: a substantial effort is required to make vendor-agnostic pulse sequences extensive within the data acquisition hardware, as it involves vendor support and validation towards regulatory clearance.

3. Advance BIDS-compliant standards for tractography inputs and outputs. Akin to a wide range of neuroimaging use-cases, including human electrophysiology and microscopy, where BIDS has demonstrated its utility, a tractography-specific extension is needed towards reliable, reproducible, and robust tractography data representation and sharing. Note that the particular adoption of the BIDS specification for tractography should not prevent software from processing isolated tractography files. Feasibility: As of writing of this article, the BIDS tractography extension is being drafted, and a small effort is needed to complete it.

4. Quality control. Standardized quality control procedures need to be fostered including automated procedures at every stage of processing. Further research on the impact of different decisions in QC is needed, to ultimately develop guidelines for best practices. Feasibility: due to the challenges inherent to tractography validation, a moderate effort will be required to define consensus measures and procedures towards implementing standardized QC protocols and pipelines.

5. Make analysis tools that are flexible to be used across different data acquisition methods, different species and different settings. FAIR-Software principles can be applied, to make sure that methods are

transparent, rigorously designed and available to the community [125]. Feasibility: a substantial effort in validation is required to generalize tools across methods, species and settings. This may include generating publicly shared, reproducible records of the datasets and procedures employed.

6. Scalability. Software and platforms for sharing and computing on brain connectivity data need to be built with the large datasets of the future in mind, and with the ability to scale to much larger data than is currently available, in anticipation of the inevitable deluge of data that is expected to occur. Infrastructure for distributed and cloud computing includes evolving standards for representation of large array-based [126], tabular [127], and even trajectory data [128] in these kinds of systems, and formats for representation of tractography data could be adapted to capitalize on these developments. Feasibility: a moderate effort can be expected to design frameworks capable of handling large volumes of data. This effort is related to the development of data representation standards inherent to tractography, and the reliance on the development of general purpose, advanced scientific software.

7. Create and share annotated datasets for machine learning training. For improved interoperability and reproducibility, use industry standards for machine learning methods, such as ONNX and widely used sharing platforms for ML/AI models and tools, such as Hugging Face. Feasibility: These both require minor efforts on the side of scientists and researchers who develop these methods.

8. Future-proof standardized file formats. The community needs to advance new file formats that address the needs for consistent explicit tractography data spatial representation, while also supporting the needs for new large-scale datasets. This includes standardized, traceable, openly available, and well-documented specifications, including community-sustained conversion information and tools. We identify the nascent TRX file-format as a format that has the potential to address many of the issues raised here, specifically because it was developed taking these aspects into consideration. Feasibility: a minor effort remains to be done in terms of documentation of the TRX file format. A few initial studies already provide evidence for the suitability of TRX for reliable, and robust tractography data representation [90, 129]. A moderate effort will be required in order to incorporate the format as the output of existing software pipelines, which would make it more widely used.

9. Prioritize the articulation of clearly specified validation frameworks with common benchmarks and metrics, alongside the development of centralized, consensus evaluation tools. Importantly, these will enable unambiguous assessment of relevant aspects like heterogeneous scanners, acquisition protocols, and imaging sources, critical to assess reliability and robustness of tractography. Feasibility: a substantial effort will be required to specify the resources —including data, metrics, and technological requirements— and to operationalize and sustain the resulting infrastructure.

10. Build the bridges between research and clinical tractography: Delivery of advanced methods into clinical practice can be facilitated by standardizing workflows, and by increasing the interoperability between different parts of the clinical informatics infrastructure. For example, via integration of visualization into surgery image-guided systems, into Picture Archiving and Communication Systems (PACS) used in clinical, and into electronic medical records [130]. This will also set the scene to improve the bench-to-bedside pipeline of new computational methods. Feasibility: while research and clinical requirements remain different, the usefulness of increasingly more anatomically reliable tractography methods needs to be translated into clinical practice. Substantial efforts in terms of validation and collaboration with vendors can be foreseen.

Reviewer Comment 1.12
----

p. 4. “Additional compressions is provided”.

Reply:

Thank you for having spot the grammar error. We have fixed it in the manuscript.

Reviewer Comment 1.13

----

p. 5. “this approach is challenged by low inter-rater variability”. Presumably the authors mean high inter-rater variability. Low variability would be a good thing.

Reply:

Correct. We have fixed the oversight in the revised manuscript.

Reviewer 2

Tractography is an important technique for mapping brain connectivity in vivo, with many clinical and scientific applications and well-documented benefits. At the same time, many prior works have reported remaining challenges that hamper clinical adoption and might question the reproducibility and validity of some scientific conclusions that have been based on tractography. These challenges include a high degree of variation in tractography results which are, among other factors, due to the use of different acquisition protocols, processing pipelines, the dependency on subjective parameter settings, and differences in white matter tract definitions, which are difficult to reconcile due to a lack of a definite gold standard in most cases; high false positive rates of many widely used approaches; a resulting need for manual intervention, fine-tuning, and filtering; and issues of trustworthiness and generalization when attempting to resolve some of those challenges via machine learning.

Therefore, bringing together the perspectives of a broad range of experts in this field to try and resolve challenges with respect to the reliability, reproducibility, and robustness of tractography through standardization, which is the goal of the standardization unit of the recently founded International Society for Tractography, is a meaningful and valuable endeavor. Given the scale of these long-standing challenges, it is clear that they cannot be resolved all at once. Therefore, the current manuscript represents an initial step of this process, which is to define a specific list of points that require standardization, and to provide a review of what has so far been achieved with respect to each of them, and what remains to be done.

Reply:

Thank you for identifying the contribution of the article. We completely agree that this is just the first step in a long road. In order to make this point even more apparent, and in response to suggestions made by Reviewer 1, we have added more explicit language about the feasibility of the next steps in each of the areas in which progress needs to be made.

At this point, I see the following opportunities for further strengthening this review:

Reviewer Comment 2.1

In my opinion, the current weight on issues related to spatial coordinates is disproportionally large, considering the relatively large amount of text devoted to this topic and, even more importantly, the fact that this is the only challenge that is illustrated with a separate and quite large figure. The problems illustrated here are certainly important in practice, but they are also well-known and not specific to tractography. I would find this kind of material more appropriate for an introductory tutorial than for a position paper that aims to shape the future of the field. I see the fact that this section contains only a single citation as further evidence that this topic is somewhat out of scope for a review.

Reply:

We agree with the reviewer that this is a technical issue where there is not necessarily scientific articles to reference. Nevertheless, there are two reasons that we would like to retain the emphasis on this point, as

well as the graphical representation of this issue. The first is that, given the broad scientific purview of GigaScience, the intended target audience for this article may include researchers from other fields, and we feel that it is important to explain and demonstrate how this simple-seeming issue is a source of significant error and significant technical difficulty. For these readers, it would be important to present a more extensive treatment of this issue, as we have done here. The second reason is specifically because there is not currently an article that we can refer to, which explains this issue with clarity. Therefore, we find that the lack of references in this section represents precisely the fact that the field needs a standard reference for these issues. This point is particularly important given how wide-spread these issues are (based on the experience of the authors of this paper). We believe our review will be a valuable resource in this respect.

Reviewer Comment 2.2

At the same time, I believe that issues related to the use of machine learning in tractography are currently underrepresented. Deep learning is briefly mentioned as a potential strategy that might support quality control, but it could in fact be used at almost all stages of the pipeline (image reconstruction, harmonization, tracking as such) and has the potential to address some of the fundamental challenges that the field has long been struggling with, in particular, ambiguous correspondences between the diffusion MRI signal and tract geometry (as discussed in [17]). However, learning based approaches come with their own set of challenges, such as generalization across acquisition devices / protocols and patient populations, reliable uncertainty quantification, or explainability, that would in my opinion merit a separate discussion.

Reply:

We acknowledge that tractography methods based on artificial intelligence techniques pose challenges across all stages of a tractography pipeline, including its methodological foundations (i.e., generating local orientation fields to propagate streamlines). We have added a section named "Integration of modern machine learning methods" (L496-584) (which we reproduce below) to explicitly highlight the challenges that modern artificial intelligence-based method represent towards tractography standardization:

The increasing adoption of machine learning and artificial intelligence (ML/AI) methods in dMRI and tractography has raised new challenges and exposed limitations in current standardization practices. Modern ML/AI methods require large, diverse, and well-curated datasets to achieve robust generalization. These requirements compound many of the challenges described. For example, in the absence of reliable and automated QC tools, labor-intensive quality control procedures are required. Spatial coordinate inconsistencies within large heterogeneous datasets may cause deep learning models to fail to converge. Similarly, poor data organization and metadata standardization —due to both poor compliance with BIDS and the preliminary status of the related extensions— hinder reproducibility and compromise downstream analyses, complicating cross-study comparisons. Inadequate handling of data splits can lead to train–test leakage [97]. Such leakage risks inflating reported performance and obscure the true generalizability of ML/AI-based tractography methods. Though there are some annotated datasets that can be used as the basis for training ML/AI , the scarcity of large, well- annotated, and openly shared tractography datasets — particularly multi-site and longitudinal datasets that include non-healthy individuals for clinical relevance — constrains the development of robust and reliable tractography.

Furthermore, there is need for standardization within the ML/AI methods themselves. ML/AI-based tractography techniques exhibit substantial heterogeneity in model architecture, input representation, and learning objectives, often making direct comparisons difficult (see [97, 98, 99] for relevant review works). Existing approaches range from voxel-wise classifiers and streamline propagation networks to graph-based and geometric learning formulations. As such, the input to the model may be the raw diffusion signal, the local orientation information represented as spherical harmonics coefficients or fiber peaks, etc. Additionally, the contextual information to regularize the optimization process is also heterogeneous across methods, and includes tissue maps, neighboring voxel information, etc. Other approaches avoid reliance on the modality that sensitizes axonal pathway architecture (e.g., dMRI or other), and estimate tractography without explicit orientation data. These factors result in differing dataset requirements for training various models, often leading to substantial increases in computational requirements, both in terms of storage and processing resources. The high computational cost —including the need for specialized hardware, such as

GPUs— makes regular testing and evaluation of methods expensive and resource-intensive.

This heterogeneity is compounded by the absence of universally accepted anatomical ground truth for white-matter pathways, a limitation that challenges the current tractography landscape. The evaluation of AI-based tractography algorithms frequently relies on indirect metrics, simulated phantoms, or expert-defined references, each with inherent limitations. In this context, the absence of standardized reporting conventions is particularly problematic for the evaluation of AI models. Although volume- and streamline-oriented measures are well-established since landmark studies [17, 100], evaluation tools remain poorly maintained, with most pipelines relying on bespoke implementations. Additionally, the assessment of application-specific derivatives —such as tract-specific metrics, connectivity measures, or quantitative microstructural parameters— is often inconsistent or omitted entirely. Insufficient reporting of scanner or session biases can also mask site- or protocol-specific effects that may dominate learned representations, resulting in performance gains that are often difficult to attribute to genuine methodological advances.

The result is a fragmented landscape in which performance claims are highly dependent on specific datasets, tasks, and evaluation choices, increasing the difficulty of reliable evaluation. Together, this underscores the necessity of standardization for AI-based tractography, encompassing access to well-annotated and fully described datasets, common validation frameworks with standardized benchmarks, metrics and reporting procedures. Such standardization would support integration with established neuroimaging pipelines, improve reproducibility, robustness, and maintainability.

More broadly, the development and application of machine learning methods are facing a fragmented landscape, where different software is used in different use-cases. Competition among large corporate entities (e.g., technology companies) is also driving this fragmentation, as different entities try to position their tools as leading in the marketplace. In cases where models are shared not only as software, but also in the form of parameter values (or “weights”), the standardization of the format of model parameters, and of the metadata associated with these parameters becomes important. This is where industry standards such as the Open Neural Network Exchange (ONNX) standard [101] could play an important role. For these use-cases, there is much to benefit from the adoption of already-existing standards that apply broadly, rather than reinventing these standards for narrow applications.

Reviewer Comment 2.3

Another aspect that I would like to see discussed in some more detail is at the data acquisition stage: Tractography is rarely the sole purpose of acquiring diffusion MRI data. Often, quantifying tissue microstructure is another important goal, and introduces additional, sometimes conflicting requirements from a diverse set of respective methods. I expect that, in practice, attempts to standardize data acquisition for tractography will not succeed if they do not account for this.

Reply:

We thank the reviewer for highlighting this important observation. We have edited the manuscript to discuss the challenges that quantitative tissue microstructure characterization represents in tractography standardization in the “Data acquisition” section (L131-142):

Tractography is rarely the sole purpose of acquiring dMRI data. Quantitative characterization of tissue microstructure is often a concurrent goal, which imposes additional and occasionally conflicting constraints stemming from distinct acquisition requirements. As the sequences for probing the tissue microstructure get more sophisticated (e.g., using tensor-valued encoding), more variability can be expected to be present in the dMRI data, and more nuanced tractography reconstructions can be expected [44]. Thus, additional methodological development is needed to align such multi-dimensional acquisition protocols across sites, to manage scan time constraints while jointly supporting tissue microstructure modeling and tractography.

Reviewer Comment 2.4

Finally, I would find it helpful if the "summary and recommendations" section were more clearly structured according to a separate discussion of a) consensus recommendations of best practices and b) open issues that require further work.

Reply:

Instead of restructuring the section entirely, and also in response to comments from Reviewer 1 (see responses to Comment 1.4 and Comment 1.11), we have now interspersed these open issues and added assessments of their feasibility into each point in the discussion, following the sections used in the body of the article.

em Editorial Manager®

Cookie Preference Center

We use cookies which are necessary to make our site work. We may also use additional cookies to analyze, improve and personalize our content and your digital experience. For more information, see our Cookie Policy.

You may choose not to allow some types of cookies. However, blocking some types may impact your experience of our site and the services we are able to offer. See the different category headings below to find out more or change your settings.

Allow all

Manage Consent Preferences

Strictly Necessary Cookies

Always active

These cookies are necessary for the website to function and cannot be switched off in our systems. They are usually only set in response to actions made by you which amount to a request for services, such as setting your privacy preferences, logging in or filling in forms. You can set your browser to block or alert you about these cookies, but some parts of the site will not then work.

Functional Cookies

These cookies enable the website to provide enhanced functionality and personalisation. They may be set by us or by third party providers whose services we have added to our pages. If you do not allow these cookies then some or all of these services may not function properly.

Performance Cookies

These cookies allow us to count visits and traffic sources so we can measure and improve the performance of our site. They help us to know which pages are the most and least popular and see how visitors move around the site.

Targeting Cookies

These cookies may be set through our site by our advertising partners. They may be used by those companies to build a profile of your interests and show you relevant adverts on other sites. If you do not allow these cookies, you will experience less targeted advertising.

Cookie List

Clear

Apply Cancel

Consent Leg.Interest

Confirm my choices

Power
