# Supplementary material for: What needs to be standardized for reliable, reproducible, and robust tractography?
Source: Gigascience. 2026 Mar 25;15:giag034. doi: 10.1093/gigascience/giag034 (PMC13137869; doi:10.1093/gigascience/giag034)
Supplement: giag034_Reviewer_1_Report_original_submission — Reviewer 1 -- 10/9/2025 [file giag034_reviewer_1_report_original_submission.pdf]

Reviewer Report

Title: What needs to be standardized for reliable, reproducible, and robust tractography?

Version: Original Submission **Date:** 10/9/2025

Reviewer name: Reviewer 1

Reviewer Comments to Author:

* Title
What needs to be standardized for reliable, reproducible, and robust tractography?
* Scope and rationale
This aim of article is threefold: introduce some of the problems related to lack of standardization for tractography, offers potential solutions, and provide recommendations for the research community relating to the solutions. The three contexts identified in the abstract are: (1) enabling reliable tract-specific analyses, (2) facilitating cross-study harmonization, and (3) supporting replicable large-scale population studies.
** Clarity
The aim of the article is clear. It seems to align with the journal's criteria for reviews: "critical assessments of emerging technologies […] white papers on standards and policy." However, the focus is not specifically aimed at "research areas relating to the utilisation of large scale research data".
** Importance and justification
Tractography is a widely used MRI analysis technique, applied to both research and clinical decision-making. Owing to its complexity, results are hard to quantify and interpret, and this lack of reliability is an important limitation.
** Does the manuscript adequately motivate the review?
There is a disconnect between the title of the review and the contents. Specifically, while the terms "reliable", "replicable", and "robust" are used in the title, they are not defined in the text and are not used prominently to organize the discussion. It would have been helpful for the authors to translate these concepts into operational terms to make the usefulness of their recommendations clearer.
** Is the structure of the manuscript logical and effective for the topic?
There are no problems of note regarding the structure of the manuscript. I note that in the metadata accompanying the manuscript, the authors reply "yes" to the question "Have you included all the information requested [regarding design and statistics] in your manuscript?" However, given that the format is effectively that of a narrative review the more correct answer would be "not applicable".
* Coverage and balance of the literature
** Does the manuscript cover the key literature?
Given the variety of problems identified the potentially relevant literature is vast. The review covers many important citations in each of the areas discussed.
** Are references up-to-date and balanced (drawn from a variety of sources when available)?
The references are up-to-date.
** Are opposing viewpoints or limitations in the literature adequately acknowledged?

I would say many of the problems discussed are already well-known (for example, spatial coordinates). Implementation of the solutions can be difficult. It might have been useful to classify the degree of progress regarding each of the problems mentioned. Some seem easy to achieve (harmonization of data formats) while others are only a distant dream. For example, people were discussing vendor-agnostic sequences in the 1990s, but the documented progress has been limited and the limitations are not only technical but also institutional (commercial).
* Critical analysis and insight
** Does the manuscript move beyond summarising to offer genuine synthesis or critique?
For a review article, the manuscript is rather brief. It is more of a summary of previous literature than a comprehensive synthesis. Likewise, the solutions offered are extremely concise. They could not be used, for example, to guide the design of a workflow aimed at acquiring and analysing multicentre DWI for tractography. It might have been useful to take one or two concrete scenarios and make specific recommendations related to these. To take an example more-or-less at random, the 2019 review of Jacquesson et al. on cranial nerve tractography synthesized previous studies and offered an opinion on emergent best practice. Although the authors may be aiming at a higher-level synthesis in the current work, their stated aim encompasses three distinct scenarios (see Scope and Rationale) and it is not clear how the recommendations might differ for each of them.
** Are the identified problems well supported by evidence, examples, or citations?
There is no doubt that the problems identified are real ones and this is supported by evidence in the literature. It is not made clear which are the most important in practice, either in terms of the widespread adoption of suboptimal protocols or in terms of the degree of loss in reliability or robustness. Not all of the sections appear to be equally relevant; as an example, how does "translation across species" relate to reliability, reproducibility or robustness? It seems that the main link is the framework developed by the Allen Mouse Brain Common Coordinate Framework, but this works only with mouse data. The problem of comparing concepts across species is more related to validity than reliability, and as such does not fit neatly into the topic of the manuscript.
** Are the proposed solutions plausible, well-reasoned, and linked to the discussed issues?
As stated, the solutions are plausible but some are much more readily available than others. It would have been helpful if the authors had compared the feasibility of the different solutions in terms of methods and technology available today. Sometimes, it seems as though suggestions are contradictory or at least are not uniformly applicable. On the one hand, standardization of file formats is implicit in recommendations i and iv, while on the other "the community needs to advance new file formats" (recommendation viii) that address the limitations of currently-existing formats. To follow this recommendation literally, researchers would have to delay studies until widely agreed-upon future-proofed formats were available. As another example, building bridges between research and clinical tractography (recommendation viii) sounds like a good idea but the requirements of research and clinical scanning are different. For clinical use, MR scanners must be approved by the FDA, EU, or similar authority. Would vendor-agnostic sequences (recommendation ii) be authorised for such use?
** Does the work contribute new conceptual clarity or practical guidance to the field?
The work is a useful summary of previous literature but does not, in my view offer a new understanding, or real practical guidance, to practitioners in the field. A statement like "To help bridge basic research and clinical work, acquisition and processing protocols need to be made more consistent across

settings" is doubtless true, but the potential reader of the work might have hoped for more concrete guidance as to where to focus their efforts, or simply examples of cases where this has been done successfully.
* Organization and coherence
** Is the work logically organised?
At a basic level, yes, but it is not clear that all the sections address the problem that is nominally the object of the work. Apart from examples mentioned above, the section on "the scale of data" is highly relevant to the focus of the journal but less well-aligned with the reliability of tractography - feasibility possibly, but the authors do not make a case that acquisition or processing pipelines are not currently robust due to a data management problem.
** Are tables, figures, or diagrams used effectively?
Figure 2 may be useful for those unfamiliar with MRI acquisition but does not add much value for experienced practitioners.
* Writing, style, tone
** Is the writing scholarly (well-reasoned and not unduly opinionated)
There are no problems of note.
** Are there problems with the use of the English language?
There are no problems of note.
* Implications and conclusions
** Do any conclusions follow logically from the discussion?
** Are any take-home messages clear and useful for researchers and practitioners?
No, this work aims more at raising awareness about likely problems than in providing solutions for specific scenarios.
** Are areas for future research or development identified?
Several areas are identified.
* Minor points
p. 4. "Additional compressions is provided".
p. 5. "this approach is challenged by low inter-rater variability". Presumably the authors mean high inter-rater variability. Low variability would be a good thing.
* Bibliography
Jacquesson T, Frindel C, Kocevar G, Berhouma M, Jouanneau E, AttyÃ© A, Cotton F. Overcoming Challenges of Cranial Nerve Tractography: A Targeted Review. Neurosurgery. 2019 Feb 1;84(2):313-325. doi: 10.1093/neuros/nyy229. PMID: 30010992.

Methods

Are the methods appropriate to the aims of the study, are they well described, and are necessary controls included? Choose an item.

Conclusions

Are the conclusions adequately supported by the data shown? Choose an item.

Reporting Standards

Does the manuscript adhere to the journal's guidelines on minimum standards of reporting? Choose an item.

Choose an item.

Statistics

Are you able to assess all statistics in the manuscript, including the appropriateness of statistical tests used? Choose an item.

Quality of Written English

Please indicate the quality of language in the manuscript: Choose an item.

Declaration of Competing Interests

Please complete a declaration of competing interests, considering the following questions:

- Have you in the past five years received reimbursements, fees, funding, or salary from an organisation that may in any way gain or lose financially from the publication of this manuscript, either now or in the future?
- Do you hold any stocks or shares in an organisation that may in any way gain or lose financially from the publication of this manuscript, either now or in the future?
- Do you hold or are you currently applying for any patents relating to the content of the manuscript?
- Have you received reimbursements, fees, funding, or salary from an organization that holds or has applied for patents relating to the content of the manuscript?
- Do you have any other financial competing interests?
- Do you have any non-financial competing interests in relation to this paper?

If you can answer no to all of the above, write 'I declare that I have no competing interests' below. If your reply is yes to any, please give details below.

No.

I agree to the open peer review policy of the journal. I understand that my name will be included on my report to the authors and, if the manuscript is accepted for publication, my named report including any attachments I upload will be posted on the website along with the authors' responses. I agree for my report to be made available under an Open Access Creative Commons CC-BY license (http://creativecommons.org/licenses/by/4.0/). I understand that any comments which I do not wish to be included in my named report can be included as confidential comments to the editors, which will not be published.

Choose an item.

To further support our reviewers, we have joined with Publons, where you can gain additional credit to further highlight your hard work (see: https://publons.com/journal/530/gigascience). On publication of this paper, your review will be automatically added to Publons, you can then choose whether or not to claim your Publons credit. I understand this statement.

Yes Choose an item.
