# Supplementary material for: What needs to be standardized for reliable, reproducible, and robust tractography?
Source: Gigascience. 2026 Mar 25;15:giag034. doi: 10.1093/gigascience/giag034 (PMC13137869; doi:10.1093/gigascience/giag034)
Supplement: giag034_Reviewer_2_Report_original_submission — Reviewer 2 -- 10/31/2025 [file giag034_reviewer_2_report_original_submission.pdf]

Reviewer Report

Title: What needs to be standardized for reliable, reproducible, and robust tractography?

Version: Original Submission **Date:** 10/31/2025

Reviewer name: Reviewer 2

Reviewer Comments to Author:

Tractography is an important technique for mapping brain connectivity in vivo, with many clinical and scientific applications and well-documented benefits. At the same time, many prior works have reported remaining challenges that hamper clinical adoption and might question the reproducibility and validity of some scientific conclusions that have been based on tractography. These challenges include a high degree of variation in tractography results which are, among other factors, due to the use of different acquisition protocols, processing pipelines, the dependency on subjective parameter settings, and differences in white matter tract definitions, which are difficult to reconcile due to a lack of a definite gold standard in most cases; high false positive rates of many widely used approaches; a resulting need for manual intervention, fine-tuning, and filtering; and issues of trustworthiness and generalization when attempting to resolve some of those challenges via machine learning.

Therefore, bringing together the perspectives of a broad range of experts in this field to try and resolve challenges with respect to the reliability, reproducibility, and robustness of tractography through standardization, which is the goal of the standardization unit of the recently founded International Society for Tractography, is a meaningful and valuable endeavor. Given the scale of these long-standing challenges, it is clear that they cannot be resolved all at once. Therefore, the current manuscript represents an initial step of this process, which is to define a specific list of points that require standardization, and to provide a review of what has so far been achieved with respect to each of them, and what remains to be done.

At this point, I see the following opportunities for further strengthening this review:

In my opinion, the current weight on issues related to spatial coordinates is disproportionally large, considering the relatively large amount of text devoted to this topic and, even more importantly, the fact that this is the only challenge that is illustrated with a separate and quite large figure. The problems illustrated here are certainly important in practice, but they are also well-known and not specific to tractography. I would find this kind of material more appropriate for an introductory tutorial than for a position paper that aims to shape the future of the field. I see the fact that this section contains only a single citation as further evidence that this topic is somewhat out of scope for a review.

At the same time, I believe that issues related to the use of machine learning in tractography are currently underrepresented. Deep learning is briefly mentioned as a potential strategy that might support quality control, but it could in fact be used at almost all stages of the pipeline (image reconstruction, harmonization, tracking as such) and has the potential to address some of the fundamental challenges that the field has long been struggling with, in particular, ambiguous correspondences between the diffusion MRI signal and tract geometry (as discussed in [17]). However, learning based approaches come with their own set of challenges, such as generalization across

acquisition devices / protocols and patient populations, reliable uncertainty quantification, or explainability, that would in my opinion merit a separate discussion.
Another aspect that I would like to see discussed in some more detail is at the data acquisition stage: Tractography is rarely the sole purpose of acquiring diffusion MRI data. Often, quantifying tissue microstructure is another important goal, and introduces additional, sometimes conflicting requirements from a diverse set of respective methods. I expect that, in practice, attempts to standardize data acquisition for tractography will not succeed if they do not account for this.
Finally, I would find it helpful if the "summary and recommendations" section were more clearly structured according to a separate discussion of a) consensus recommendations of best practices and b) open issues that require further work.

Methods

Are the methods appropriate to the aims of the study, are they well described, and are necessary controls included? Choose an item.

Conclusions

Are the conclusions adequately supported by the data shown? Choose an item.

Reporting Standards

Does the manuscript adhere to the journal's guidelines on minimum standards of reporting? Choose an item.

Choose an item.

Statistics

Are you able to assess all statistics in the manuscript, including the appropriateness of statistical tests used? Choose an item.

Quality of Written English

Please indicate the quality of language in the manuscript: Choose an item.

Declaration of Competing Interests

Please complete a declaration of competing interests, considering the following questions:

- Have you in the past five years received reimbursements, fees, funding, or salary from an organisation that may in any way gain or lose financially from the publication of this manuscript, either now or in the future?
- Do you hold any stocks or shares in an organisation that may in any way gain or lose financially from the publication of this manuscript, either now or in the future?

- Do you hold or are you currently applying for any patents relating to the content of the manuscript?
- Have you received reimbursements, fees, funding, or salary from an organization that holds or has applied for patents relating to the content of the manuscript?
- Do you have any other financial competing interests?
- Do you have any non-financial competing interests in relation to this paper?

If you can answer no to all of the above, write 'I declare that I have no competing interests' below. If your reply is yes to any, please give details below.

I declare that I have no competing interests

I agree to the open peer review policy of the journal. I understand that my name will be included on my report to the authors and, if the manuscript is accepted for publication, my named report including any attachments I upload will be posted on the website along with the authors' responses. I agree for my report to be made available under an Open Access Creative Commons CC-BY license (http://creativecommons.org/licenses/by/4.0/). I understand that any comments which I do not wish to be included in my named report can be included as confidential comments to the editors, which will not be published.

Choose an item.

To further support our reviewers, we have joined with Publons, where you can gain additional credit to further highlight your hard work (see: https://publons.com/journal/530/gigascience). On publication of this paper, your review will be automatically added to Publons, you can then choose whether or not to claim your Publons credit. I understand this statement.

Yes Choose an item.
