# Supplementary material for: What needs to be standardized for reliable, reproducible, and robust tractography?
Source: Gigascience. 2026 Mar 25;15:giag034. doi: 10.1093/gigascience/giag034 (PMC13137869; doi:10.1093/gigascience/giag034)
Supplement: giag034_Reviewer_2_Report_revision_1 — Reviewer 2 -- 2/20/2026 [file giag034_reviewer_2_report_revision_1.pdf]

Reviewer Report

Title: What needs to be standardized for reliable, reproducible, and robust tractography?

Version: Revision 1 **Date:** 2/20/2026

Reviewer name: Reviewer 2

Reviewer Comments to Author:

This revision represents an honest effort to address my original comments. I would like to share the following thoughts regarding the newly added material:

I do not find the proposed distinction between reproducibility ("ability to obtain equivalent results across sites") and robustness ("stability in the face of varying conditions (e.g., multi-site)") sufficiently clear.

While I agree that ML-based tractography could benefit from increased data standardization and availability, I disagree with the suggestion that the heterogeneity in approaches that are currently being explored in related research implies an immediate need for standardization. I would argue that maintaining diversity in the approaches a scientific community explores is essential for innovation, and increases its chance of jointly arriving at a good solution.

I would also argue that the difficulty of reliable evaluation, in particular, the absence of universally accepted anatomical ground truth, is not a specific limitation for the evaluation of "AI-based" algorithms, but for the evaluation of tractography in general.

I would suggest to replace the somewhat vague complaint of "Insufficient reporting of scanner or session biases that mask site or protocol-specific effects that may dominate learned representation" with a clear recommended guideline for reporting.

I understand that "fragmented research landscapes" come with challenges such as fair comparisons, but had the impression that this point was highlighted somewhat redundantly in similar words in two subsequent paragraphs.

The discussion on the translation of tractography to neurosurgery no longer accurately reflects the current state of the art. In particular, the manuscript repeats claims that have been made for more than a decade, i.e., that commercially available navigation platforms offer DTI as the only option, and that practical adoption of tractography is hindered by large false positive rates. The paper even concludes by stating that substantial efforts can be foreseen to translate more anatomically reliable tractography methods into clinical practice. In fact, recent versions of the widely used navigation software by BrainLab already offer CSD-based reconstruction of complex fiber architectures, along with templates for a clean and automated reconstruction of many relevant tracts (see https://www.brainlab.com/surgery-products/overview-neurosurgery-products/elements-fibertracking/).

I do not think [103] is suitable to support the claim that the use of tractography in the planning of neurosurgical procedures remains limited, given its focus on the specialized use case of intraoperative (as opposed to preoperative) tractography.

Methods

Are the methods appropriate to the aims of the study, are they well described, and are necessary controls included? Choose an item.

Conclusions

Are the conclusions adequately supported by the data shown? Choose an item.

Reporting Standards

Does the manuscript adhere to the journal's guidelines on minimum standards of reporting? Choose an item.

Choose an item.

Statistics

Are you able to assess all statistics in the manuscript, including the appropriateness of statistical tests used? Choose an item.

Quality of Written English

Please indicate the quality of language in the manuscript: Choose an item.

Declaration of Competing Interests

Please complete a declaration of competing interests, considering the following questions:

- Have you in the past five years received reimbursements, fees, funding, or salary from an organisation that may in any way gain or lose financially from the publication of this manuscript, either now or in the future?
- Do you hold any stocks or shares in an organisation that may in any way gain or lose financially from the publication of this manuscript, either now or in the future?
- Do you hold or are you currently applying for any patents relating to the content of the manuscript?
- Have you received reimbursements, fees, funding, or salary from an organization that holds or has applied for patents relating to the content of the manuscript?
- Do you have any other financial competing interests?
- Do you have any non-financial competing interests in relation to this paper?

If you can answer no to all of the above, write 'I declare that I have no competing interests' below. If your reply is yes to any, please give details below.

I declare that I have no competing interests

I agree to the open peer review policy of the journal. I understand that my name will be included on my report to the authors and, if the manuscript is accepted for publication, my named report including any attachments I upload will be posted on the website along with the authors' responses. I agree for my report to be made available under an Open Access Creative Commons CC-BY license (http://creativecommons.org/licenses/by/4.0/). I understand that any comments which I do not wish to be included in my named report can be included as confidential comments to the editors, which will not be published.

I agree to the open peer review policy of the journal

To further support our reviewers, we have joined with Publons, where you can gain additional credit to further highlight your hard work (see: https://publons.com/journal/530/gigascience). On publication of this paper, your review will be automatically added to Publons, you can then choose whether or not to claim your Publons credit. I understand this statement.

Yes Choose an item.
